# Efficient Entropy‐Driven Inhibition of Dipeptidyl Peptidase III by Hydroxyethylene Transition‐State Peptidomimetics

**DOI:** 10.1002/chem.202102204

**Published:** 2021-08-31

**Authors:** Jakov Ivkovic, Shalinee Jha, Christian Lembacher‐Fadum, Johannes Puschnig, Prashant Kumar, Viktoria Reithofer, Karl Gruber, Peter Macheroux, Rolf Breinbauer

**Affiliations:** ^1^ Institute of Organic Chemistry Graz University of Technology Stremayrgasse 9 8010 Graz Austria; ^2^ Institute of Biochemistry Graz University of Technology Petersgasse 12/2 8010 Graz Austria; ^3^ Institute of Molecular Biosciences University of Graz Humboldtstr. 50 8010 Graz Austria

**Keywords:** drug design, inhibitor, medicinal chemistry, metalloprotein, peptidomimetics

## Abstract

Dipeptidyl peptidase III (DPP3) is a ubiquitously expressed Zn‐dependent protease, which plays an important role in regulating endogenous peptide hormones, such as enkephalins or angiotensins. In previous biophysical studies, it could be shown that substrate binding is driven by a large entropic contribution due to the release of water molecules from the closing binding cleft. Here, the design, synthesis and biophysical characterization of peptidomimetic inhibitors is reported, using for the first time an hydroxyethylene transition‐state mimetic for a metalloprotease. Efficient routes for the synthesis of both stereoisomers of the pseudopeptide core were developed, which allowed the synthesis of peptidomimetic inhibitors mimicking the VVYPW‐motif of tynorphin. The best inhibitors inhibit DPP3 in the low μM range. Biophysical characterization by means of ITC measurement and X‐ray crystallography confirm the unusual entropy‐driven mode of binding. Stability assays demonstrated the desired stability of these inhibitors, which efficiently inhibited DPP3 in mouse brain homogenate.

## Graphical abstract

A transition‐state‐based inhibitor has been developed which efficiently inhibits the metalloprotease dipeptidylpeptidase 3 (DPP3) by releasing structured water molecules from the binding cleft.

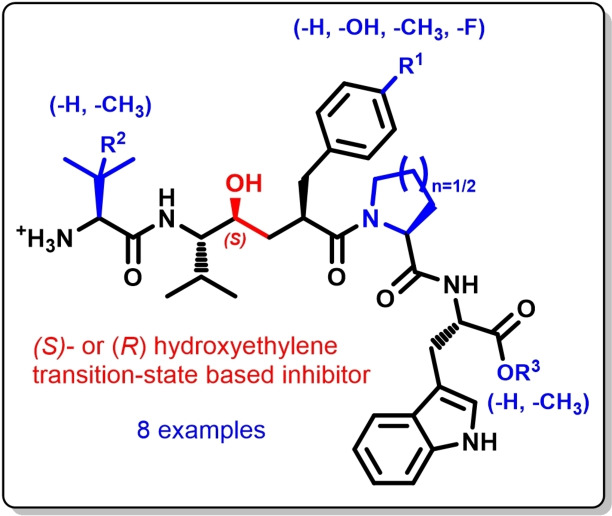

## Introduction

Dipeptidyl peptidase III (DPP3), also known as enkephalinase B, is a Zn‐dependent metalloprotease, which is ubiquitously expressed by human, bovine, porcine, monkey, rat, insect, yeast and other organisms. DPP3 cleaves the first two amino acids at the N‐terminus of biologically important oligopeptides ranging from 3–10 amino acids.

Over the last decades, several reports have been published characterizing the biological functions of DPP3. DPP3 was found to have a very high affinity towards angiotensins and enkephalins, suggesting a role in regulating enkephalin and angiotensin signaling.[^1^, ^2^] In fact, patients suffering pain show a lower activity of DPP3 in cerebrospinal fluid (CSF) compared to patients without pain.^[3]^ Literature reports an unusually high concentration of DPP3 in cancer cells, such as squamous cell lung carcinoma,^[4]^ glioblastoma cells,^[5]^ and ovarian malignant tissue.^[6]^ It has been shown that DPP3 is able to block the ubiquitination of NRF2 (nuclear factor erythroid 2‐related factor 2) by competing to interact with KEAP1 (kelch‐like ECH‐associated protein 1) ubiquitin ligase.^[7]^ Recently, Deniau et al. could show that circulating DPP3 (cDPP3) was elevated in cardiogenic shock patients and that high levels of cDPP3 were associated with altered hemodynamics and poor outcomes.^[8]^ Elevated levels of cDPP3 were found in a cohort study of critically ill sepsis patients with the concentrations corresponding to the severity of the disease. Septic shock patients showed significantly higher levels of cDPP3 compared to patients with severe sepsis.^[9]^ In an experimental model of sepsis it could be shown, that the inhibitory cDPP3‐antibody Procizumab restored altered cardiac function during sepsis in rats.^[10]^

These biological observations indicate that DPP3 plays an important role in several pathophysiological disease states. In order to study its chemical biology and validate it as a drug target a specific small molecule inhibitor would be highly desirable.^[11]^ Mammalian DPP3 is significantly inhibited by different small molecules that are potent covalently binding cysteine peptidase inhibitors, such as various organomercury compounds,[^12^, ^13^] and multiple covalently binding serine peptidase inhibitors like phenylmethanesulfonylfluoride (PMSF), diisopropylfluoro‐phosphate (DFP), 3,4‐dichloroisocoumarin (DCI) or tosyl‐phenylalanyl chloromethyl ketone (TPCK).[^13^, ^14^, ^15^] The naphthoquinone natural products fluostatins A and B isolated from *Streptomyces sp. TA‐3391* are very potent competitive inhibitors of placental DPP3. With arginyl‐arginine‐2‐naphthyl‐amide (H‐Arg‐Arg‐βNA) as a synthetic substrate, the naphthoquinone natural products fluostatins A and B exhibited IC_50_ values of 1.44 and 74.0 μM, respectively.^[16]^ However the quinone moiety is considered as a “pan‐assay‐interference compounds” (PAINS) fragment.^[17]^ To date, the strongest described “inhibitors” of DPP3 are oligopeptides. Yamamoto et al.^[18]^ and Nishimura et al.^[19]^ reported the finding and isolation of very potent and selective “inhibitors”, spinorphin (LVVYPWT) and its truncated form tynorphin (VVYPW), respectively. Tynorphin, which exhibited selectivity for enkephalin‐degrading enzymes, is slowly hydrolyzed by the enzyme, resulting in complete degradation of tynorphin in human serum at 37 °C within 4 h.^[18]^ Therefore, these peptides should be better regarded as slowly‐converted (poor) substrates instead of true inhibitors. Twelve synthesized amidino benzimidazole compounds were tested as inhibitors of DPP3, solely on the basis of bioisosterism of amidino groups to arginine guanidine residues from the DPP3 test substrate H‐Arg‐Arg‐βNA and were found to inactivate the enzyme by an irreversible inhibition mechanism.^[20]^ In summary, none of the above‐described substances, fulfills the criteria expected from a chemical probe,^[21]^ which would allow to validate DPP3 as a drug target.

Herein, we report how we used available information of preferred peptide substrates and from crystal structures of the open and substrate‐bound form of DPP3, produced in our laboratory, to design a transition‐state based inhibitor of DPP3, which should overcome limitations of previous attempts of DPP3 inhibitors as described above.

## Results and Discussion

**Figure 1:**
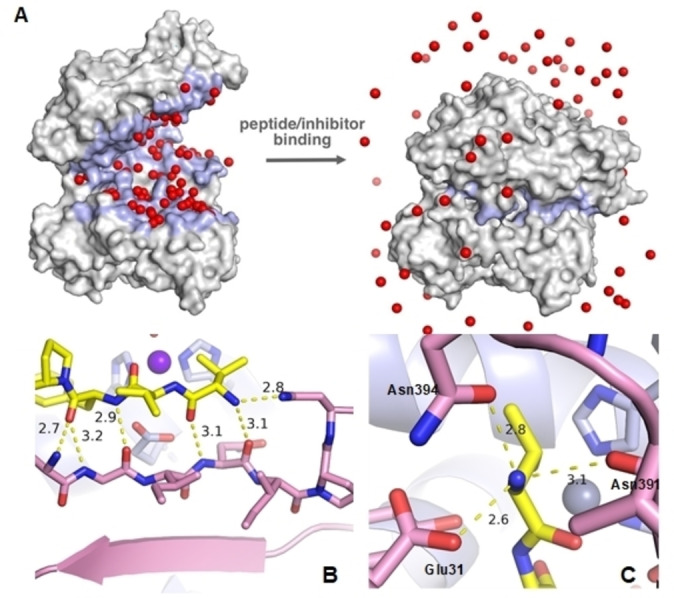
(A) X‐ray structure of hDPP3 without a ligand (PDB: 3FVY) (left). Upon substrate binding (PDB: 3T6B) (right) large conformational changes occur and structured H_2_O molecules (red balls) are released. (B) Binding mode of tynorphin showing an extended β‐sheet. The Zn‐ion and the catalytic Glu451 residue were added and force field optimized with molecular modelling software MOLOC.^[23]^ (C) Amino‐terminal ammonium group of the ligand is bound very tightly via three hydrogen bonds and a salt bridge to Glu316.

Previously, we had solved the structure of an inactive E451A‐variant of hDPP3 in complex with tynorphin (PDB code: 3T6B), which reveals how the peptide substrate binds to this enzyme (Figure 1).^[22]^ DPP3 has two big lobe‐like domains mostly composed of α‐helices with a smaller β‐sheet portion in the lower domain. Upon ligand binding, the lobes close encapsulating the substrate completely. This process is accompanied by a large loss of solvent‐accessible area of approximately 3,500 Å^2^, thereby releasing approximately 60 structured water molecules, which represents the entropic driving force of binding overcompensating the unfavourable, i. e. positive, binding enthalpy of the peptide substrate (Figure 1A).^[22]^

The first three amino‐terminal amino acid units of the peptide substrate bind in the form of an extended β‐sheet hydrogen bond network. Importantly, valorphin, tynorphin, tynorphin‐like pentapeptides, and angiotensin III share a structural motif in their first three amino acid residues. The first amino acid is variable, but the second and the third are the same (*X*VY). This common structure‐activity relationship feature could be an indication of evolutionary training of DPP3 to recognize such sequences with higher selectivity.

The N‐terminus of the peptide substrate is charged and forms a salt bridge to the side chain of Glu316 of the enzyme (Figure 1C). It also forms two more hydrogen bonds to the side chains of Asn394 and Asn391. This very tight interaction most probably represents an ammonium cation recognition site. The *C*‐terminal tryptophan residue, along with two hydrogen bonds, adds also a pincer‐like cation‐π interaction with Lys670 and Arg669 residues within the enzyme. The cation‐π interaction is well characterized as one of the strongest noncovalent interactions in protein environments.^[24]^ This additional localized set of tight interactions in the tynorphin‐DPP3 complex is the probable cause of the higher affinity of binding and the ability to act as an inhibitor, compared to the other peptide substrates of DPP3 (e. g. enkephalins, which have Leu or Met as a *C*‐terminal residue, or endomorphins, which are shorter by one amino acid unit and thus can hardly interact with this site).^[25]^

The second peptide bond (between P1 and P1’ according to the Schechter‐Berger‐nomenclature) is positioned for cleavage by nucleophilic attack in the wild type enzyme. It is surrounded by the catalytic apparatus, consisting of four ligands complexing the Zn‐ion (a molecule of water, Glu508, His450 and His 455) and the Glu451, as well as two additional residues (Tyr318 and His568) involved in precise substrate positioning and stabilization of the transition state. Tyr318 has been reported as an important, conserved residue in the family of DPP3 enzymes. Replacement of this residue leads to a decrease of the *k_cat_*‐value by two orders of magnitude.[^22^, ^26^] Tyr318 forms hydrogen bonds to the first amide bond of the peptide ligand and to Glu508, thus bringing together the catalytic apparatus and the peptide substrate backbone. His568 apparently has the major role in stabilization of the transition state. From the X‐ray structure of the complex, it can be predicted to be within hydrogen bonding distance to the carbonyl oxygen of the cleavable peptide bond, but the interaction is weak, because lone electron pair orbitals are positioned orthogonally to the N−H donor of the His568.

**Scheme 1:**
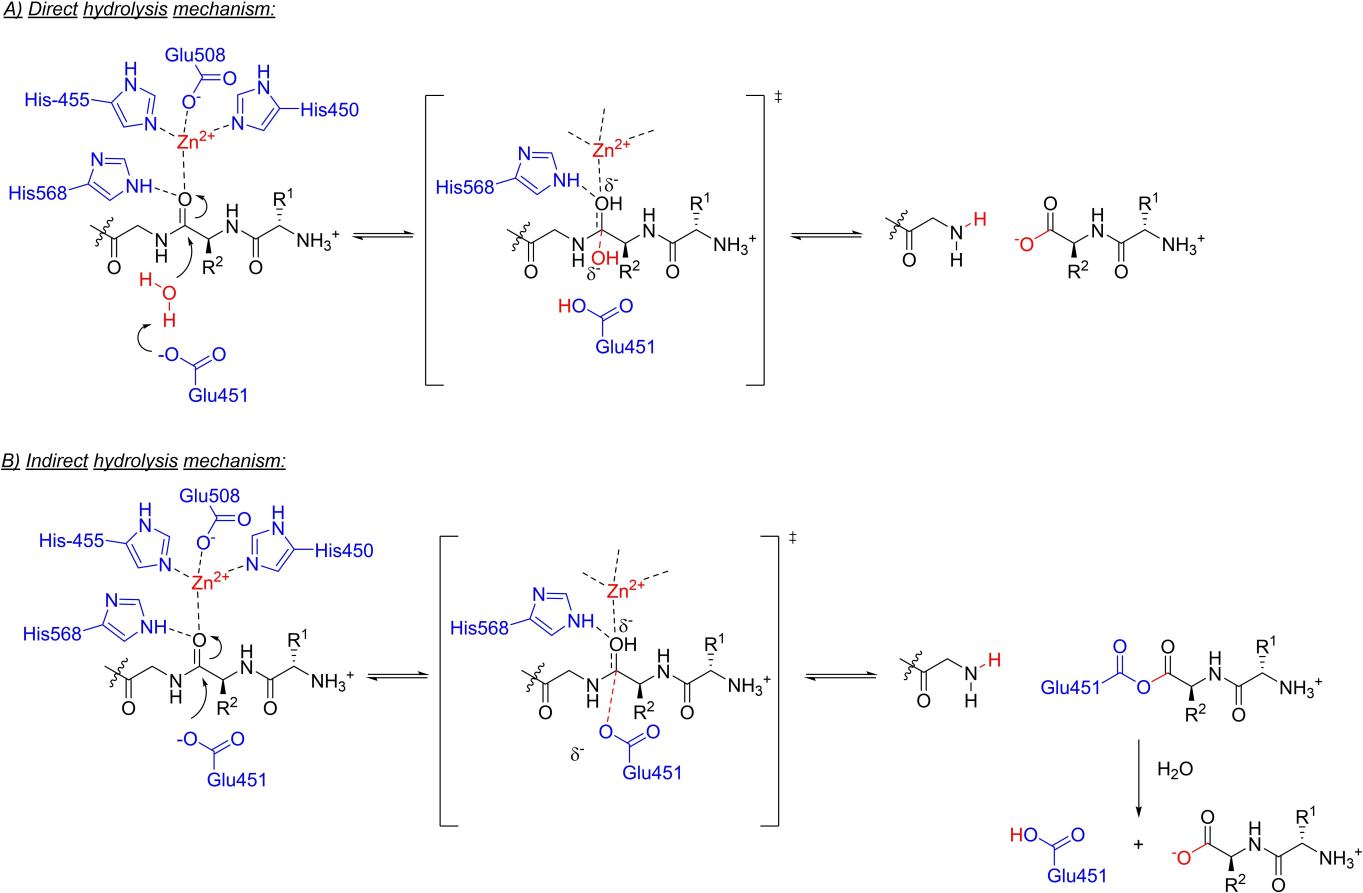
Hydrolysis mechanism of DPP3.

The binding mode of substrates to DPP3 occurs through backbone interactions of the peptide via hydrogen bonds, which is a common binding mode in proteases and enables a broad substrate specificity. The domain motion is responsible for positioning the scissile amide bond correctly to the catalytic centre. The zinc ion of hDPP3 is tetrahedrally coordinated by His450, His455 (both from HELLGH motif), Glu508 (from EECRAE motif) and a water molecule. A glutamate from HELLGH motif (Glu451) has been proposed to deprotonate a water molecule, which attacks the peptide bond coordinated to the zinc ion and His568. After a tetrahedral transition state, the peptide bond is cleaved (Scheme 1).[^22^, ^27^]

In a recent study, we analyzed the structures of complexes of hDPP3 with enkephalin (Leu/Met), angiotensin II, endomorphin‐2 as well as IVYPW via X‐ray crystallography and observed a difference in coordination of the carbonyl group of the scissile bond. In the complexes with Leu‐ and Met‐enkephalin, the zinc ion does not interact directly with the carbonyl group; instead, a water molecule is coordinated to it. However, in the complex with IVYPW, the water molecule is missing, but Glu451 has a smaller distance to the scissile peptide bond. Therefore, Glu451 may also act directly to the bond as a nucleophile forming an acyl‐enzyme‐like intermediate (Scheme 1).^[28]^ These two distinct mechanisms may be the explanation for the open question, why some peptides are good substrates whereas other peptides act as “inhibitors”.^[28]^

From the analysis of our crystallographic structures and biophysical characterization of the binding of peptide substrates, we framed the following tenets for inhibitor design: 1) the inhibitor should be structurally very similar to the best substrates in order to snuggly fit into the binding pockets inducing the release of bound water molecules to support entropically driven binding; 2) in order to take advantage of the enthalpic contribution of backbone binding, the inhibitor should be peptide based, 3) to be a true inhibitor the scissile bond between the second and third amino acid should be replaced by a stable entity, 4) in order to promote specificity, typical Zn binding motifs known to bind to metalloproteases such as hydroxamic acids, sulfonamides, etc. should be avoided,^[29]^ and 5) a transition‐state mimetic might increase the binding affinity.^[30]^ While transition‐state mimetics have been widely and very successfully used for the development of Ser‐, Cys‐ and Asp‐proteases,^[31]^ they have so far only been applied to thermolysin, as the sole representative of a metalloprotease.^[32]^

**Figure 2:**
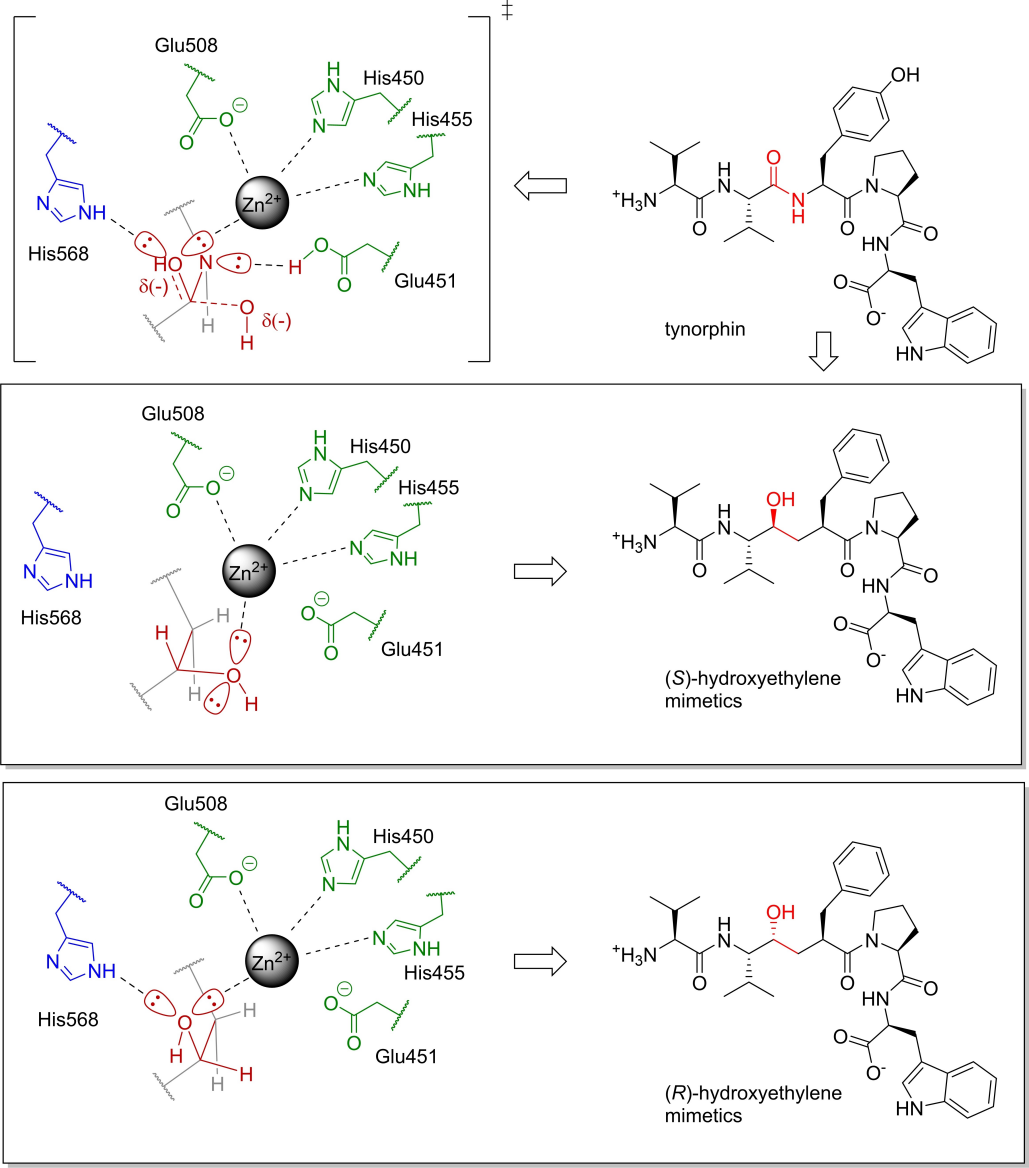
Proposed hydroxyethylene transition state mimetics as tynorphin derived inhibitors of hDPP3.

Not discouraged by the lack of precedence, we proposed for our DPP3 inhibitor design to incorporate a hydroxyethylene moiety instead of the cleavable peptide bond, as a noncleavable isostere resembling the transition state in peptide bond hydrolysis.^[33b]^ Hydroxyethylene has a tetrahedral geometry, equivalent to the geometry of the transition state. Moreover, it has a stable chiral configuration, and it can be obtained in two different configurations, both viable for synthesis (Figure 2).

It could be predicted from the representations of both inhibitors in the binding site, that the *(S)*‐hydroxyethylene could coordinate to the zinc ion with a lone electron pair from the hydroxyl substituent. On the other hand, *(R)*‐hydroxyethylene was expected to form both a coordinative bond with the zinc ion and a hydrogen bond to His568, very similar to the transition state configuration occurring during peptide hydrolysis. With one additional major noncovalent interaction, *(R)‐*hydroxyethylene was expected to have a more favorable enthalpy of binding and thus a stronger inhibitory effect.

It could be predicted from the representations of both inhibitors in the binding site, that the *(S)*‐hydroxyethylene could coordinate to the zinc ion with a lone electron pair from the hydroxyl substituent. On the other hand, *(R)*‐hydroxyethylene was expected to form both a coordinative bond with the zinc ion and a hydrogen bond to His568, very similar to the transition state configuration occurring during peptide hydrolysis. With one additional major noncovalent interaction, *(R)‐*hydroxyethylene was expected to have a more favorable enthalpy of binding and thus a stronger inhibitory effect.

We set out to produce both *(S)‐* and *(R)‐*epimers of hydroxyethylene transition state mimetics of tynorphin. To facilitate our initial synthetic efforts, we left out the hydroxy residue of the tyrosine side chain and decided to produce the molecules containing a pseudo‐phenylalanine instead (Figure 2). Using the main tynorphin scaffold was expected to provide selectivity over other enkephalinases, as demonstrated in the research on endogenous peptides inhibiting DPP3.

**Scheme 2:**
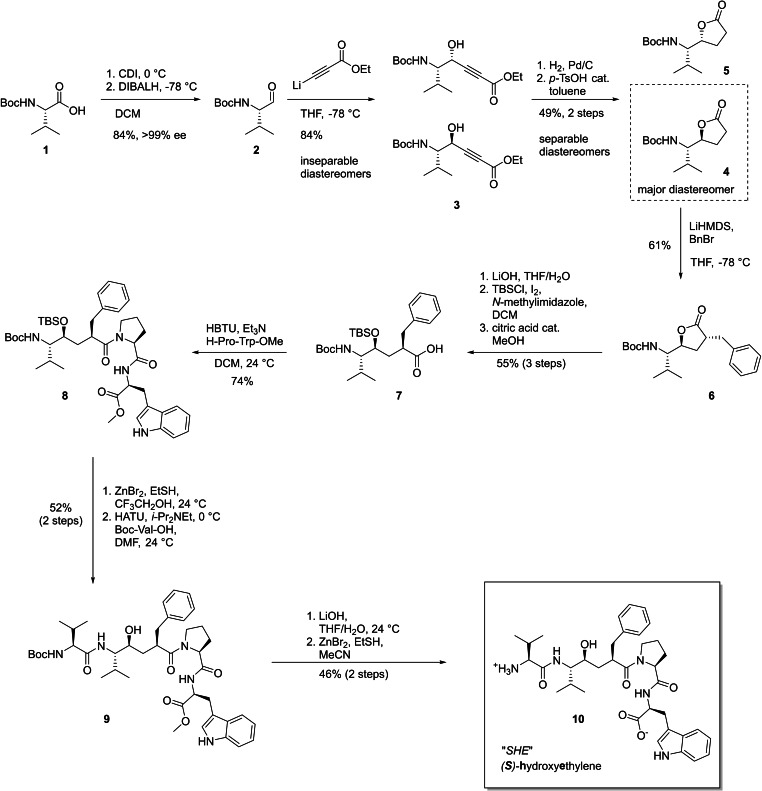
Synthesis of the *(S)‐*hydroxyethylene transition state mimetic “SHE” (**10**).

The synthesis of the central pseudopeptide building block (Scheme 2) followed a homoenolate strategy, which had been established by Ghosh for a slightly different substrate leading to a SARS protease inhibitor.^[34]^ *N*‐*tert*‐Butyloxycarbonyl (Boc) protected valine (**1**) was converted to the corresponding chiral aldehyde **2** using our recently developed convenient one‐pot two‐step methodology in which the amino acid is activated with Staab's reagent (1,1’‐carbonyldiimidazole, CDI),^[35]^ and the resulting intermediate imidazolide is selectively reduced to the aldehyde using diisobutylaluminium hydride (DIBAL−H).^[36]^ The aldehyde was isolated in 84 % yield and >99 % ee by an extractive workup, requiring no further purification. The amino aldehyde **2** was reacted with lithiated ethyl propiolate to obtain a mixture of two diastereomers of propargylic alcohols **3**, which were inseparable at this stage.

The alcohols **3** were catalytically hydrogenated with Pd/C, and the saturated intermediates were lactonized with catalytic amounts of *p*‐toluenesulfonic acid. The resulting lactones **4** and **5** were readily separated by flash chromatography. The major diastereomer was lactone **4** with the desired *(S)‐*configuration at the stereogenic centre at the lactone. **4** was enolized by deprotonation with lithium hexamethyldisilazide (LiHMDS) at −78 °C and alkylated with benzyl bromide. As the approach to the *Si*‐face of the ring of the enolate lactone is hindered by the bulky substituent, the electrophile attacked preferentially from the *Re*‐face. Accordingly, the benzylated lactone **6** was isolated in 61% yield without observation of significant amounts of the diastereomer that would be formed from the attack of the opposite face.

With the stereoselectively alkylated lactone **6** in hand, the pseudodipeptide could be produced by lactone opening for which several experimental issues had to be very carefully addressed. Although the lactone was completely opened by stirring for 1 h with at least 4.0 equiv. of LiOH in tetrahydrofuran/water (THF/H_2_O), the 4‐hydroxyacid intermediate appears to be very prone to spontaneous lactonization. The degree of unwanted lactonization was found to be highly dependent on the temperature and the acidity of the workup. The acidification had to be performed carefully at 0 °C using 25 % aqueous citric acid to adjust the pH value to 4. Also, it was found that if the temperature of the water bath used for the evaporation of solvents was >30 °C, the majority of the intermediate lactonized to the starting material. Further problems in this reaction were faced due to known issues with the *tert*‐butyldimethylsilyl (TBS) protection of sterically hindered secondary alcohols like the 4‐hydroxyacid intermediate. Initially, our efforts to utilize the standard TBSCl/imidazole method^[37]^ were unsuccessful. When using a large excess of reagents according to the protocol of Ghosh,[^34^, ^38^] the reactions were extremely slow, very low yielding, and irreproducible. Fortunately, a silylation protocol developed by Stawinski^[39]^ for sterically demanding substrates using the combination of TBSCl/*N*‐methylimidazole/iodine worked successfully for our substrate. In contrast to literature precedence,[^34^, ^38^] we observed that the simultaneously formed TBS‐ester could not be readily deprotected by short methanolysis. This could be solved by performing the methanolysis reaction in the presence of a catalytic amount of citric acid. The selective cleavage was completed within 6 h without harming the TBS‐ether and furnished protected pseudopeptide building block **7** in 55 % yield.

In order to complete the synthesis, the protected intermediate **7** was coupled with the H‐Pro‐Trp‐OMe dipeptide fragment using (2‐(1*H*‐benzotriazol‐1‐yl)‐1,1,3,3‐tetramethyluronium hexafluorophosphate (HBTU) as a coupling reagent delivering pseudotetrapeptide **8** in 74 % isolated yield. As the hydroxyethylene acid cannot form an oxazolone intermediate, no racemization was observed in this coupling step.^[40]^ In order to complete the required pentapeptide‐like scaffold, *N*‐Boc deprotection of the peptide and coupling with Boc‐L‐valine were necessary. While the peptide coupling was straightforward the orchestration of the deprotection strategy turned out to be a delicate matter. Treatment of **8** with TFA/ethanethiol resulted in neat and selective *N*‐Boc deprotection, whereas anhydrous HCl in the protic solvent resulted in rapid dual deprotection of both the Boc and TBS function, causing also a thermodynamically favored, acid‐catalyzed lactone cyclization “backbite” in the peptide, which has already been observed by Rich in earlier studies.^[33b]^ Ultimately, we could solve this issue by the use of ZnBr_2_ in 2,2,2‐trifluoroethanol^[41]^ with EtSH as an additive, which led to dual deprotection of both Boc as well as TBS without generating byproducts.

Taking advantage of the higher nucleophilicity of amines compared to alcohol nucleophiles,^[42]^ the *N,O*‐deprotected pseudotetrapeptide was coupled with Boc‐Val‐OH to afford **9** in 52 % isolated yield (2 steps) with no observed ester coupling byproduct. The target molecule **10** was easily obtained after saponification of the *C*‐terminal methyl ester with LiOH and *N*‐Boc deprotection with ZnBr_2_ and EtSH. Since the *(**S**)‐* **h**ydroxy**e**thylene transition state isostere is contained in **10** we assigned an arbitrary abbreviation “SHE” to this final molecule.

**Scheme 3:**
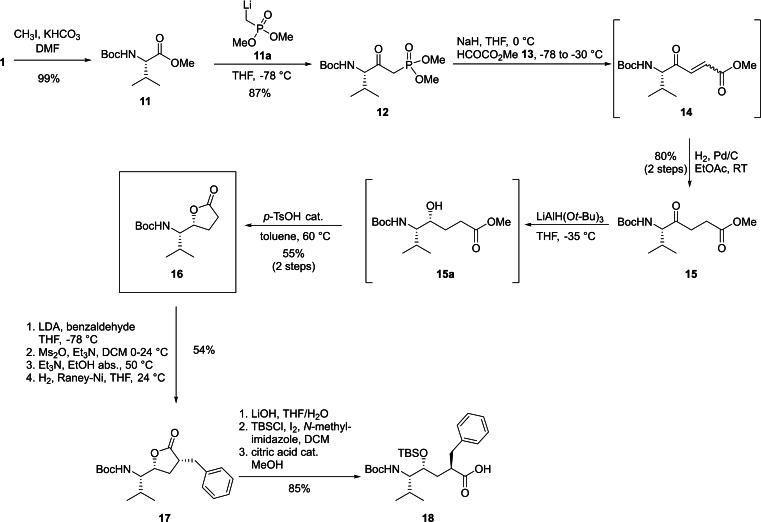
Synthesis of *(R)‐*hydroxyethylene pseudopeptide **18**.

The synthesis route for the opposite hydroxyethylene diastereomer (Scheme 3) used methodology developed by Rich,^[33a]^ who reported the synthesis of a Gln‐Phe hydroxyethylene dipeptide isostere as a precursor for proposed BoNT metalloprotease inhibitors. Our synthetic route started from *N*‐Boc‐protected L‐valine (**1**), which was converted into methyl ester **11** by using methyl iodide and potassium bicarbonate in *N*,*N*‐dimethylformamide (DMF) in 99 % yield and excellent purity without requiring further purification.[^43^, ^44^] In the next step, dimethyl methylphosphonate was lithiated with *n*‐butyllithium to generate lithiated compound **11 a**, which was allowed to react with **11** to β‐keto phosphonate **12** at ‐78 °C.^[45]^ Deprotonation of **12** was achieved with NaH and underwent a HWE reaction with freshly prepared methyl glyoxylate (**13**)[^33a^, ^46^, ^47^] at −30 °C. The crude mixture of *cis*‐ and *trans*‐isomers **14** was then hydrogenated with Pd/C to produce keto ester **15** in 80 % yield over two steps. Stereoselective reduction of **15** with lithium tri‐*tert*‐butoxyaluminum hydride at a temperature optimum between −40 °C to −30 °C to minimize formation of the unwanted *(S,S)‐*diastereomer and required 20 h for completion. The resulting amino alcohol intermediate **15 a** could be easily lactonized by addition of a catalytic amount of *p*‐toluenesulfonic acid in toluene and stirring at 60 °C for 12 h. The major part of the desired *(R,S)‐*γ‐lactone **16** could be obtained in a very pure form just by precipitation induced by addition of *n*‐hexane onto the crude oil. An additionally performed flash chromatography of the concentrated filtrate increased the yield of **16** to an acceptable 55 %. The absolute configuration of the stereogenic centers could be unambiguously assigned via X‐ray crystallography.

With γ‐lactone **16** in hand the *O*‐protected pseudodipeptide core molecule was synthesized by a multi‐step sequence leading to the α‐alkylation product, followed by opening of the lactone and immediate protection of the hydroxy moiety. For the stereoselective alkylation a four‐step sequence according to Nadin was used.^[48]^ In the first step, γ‐lactone **16** was enolized by deprotonation with lithium diisopropylamide (LDA) at ‐78 °C in THF and allowed to react with freshly distilled benzaldehyde to form the corresponding β‐hydroxy lactone **16 a** as a complex mixture of diastereomers in 71 % yield. Subsequent treatment with methane sulfonic anhydride, followed by a Et_3_N‐induced elimination resulted in an α,β‐unsaturated lactone **16 b**. The crude **16 b** was then hydrogenated with Raney‐Nickel as hydrogenation in previous attempts with palladium on charcoal had failed. Adsorption of the lactone substrate to the metal catalyst occurs with the less hindered face explaining the observed high stereoselectivity for the production of **17**.^[33a]^ For the delicate ring opening of lactone **17**, we could build on the experiences made in the synthesis of SHE (**10**) in order to avoid degradative lactonization. After opening of the lactone with LiOH in THF/H_2_O=2 : 3 (v/v) at rt, the reaction mixture was cooled to 0 °C and acidified to pH=4 by the careful addition of 25 % citric acid. After aqueous workup the solvents were removed carefully at a temperature below 30 °C to avoid degradative lactonization. Immediately after drying of the crude product the hydroxy moiety was TBS‐protected with TBSCl/I_2_/*N*‐methylimidazole.^[39]^ Methanolysis with a catalytic amount of citric acid converted the resulting TBS‐ester^[34]^ into the desired pseudodipeptide core molecule **18** in 85 % yield over 3 steps.

**Scheme 4:**
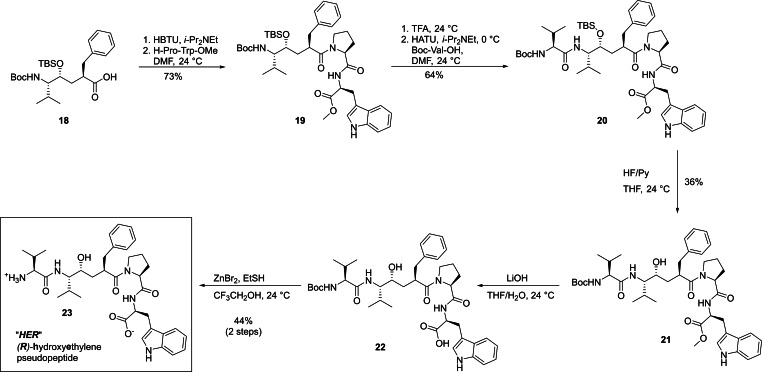
Synthesis of *(R)‐*hydroxyethylene inhibitor “HER” (**23**).

Now the core structure was ready to be elongated at both the C‐ and N‐termini using similar steps as developed for the synthesis of SHE. As shown in Scheme 4, synthesis started with compound **18**, which was coupled with dipeptide H‐Pro‐Trp‐OMe with HBTU/*i*‐Pr_2_NEt (DIPEA) as coupling agent. After purification of the reaction mixture via flash chromatography, a significant amount of tetramethylurea from peptide coupling remained, which could be removed by extensively washing with H_2_O. Pseudopentapeptide **19** was isolated as a white solid in 73 % yield in sufficient quantities, which gave us the opportunity to split the bulk for the synthesis of additional inhibitors. For the next peptide coupling the Boc‐group of **19** was first removed with TFA and then Boc‐Val‐OH was attached with *O*‐(7‐azabenzotriazol‐1‐yl)‐*N*,*N*,*N*’,*N*’‐tetramethyluronium hexafluorophosphate (HATU) as a coupling reagent to avoid the risk of epimerization. Again, tetramethylurea had to be removed after flash chromatography by several H_2_O washing steps delivering **20** in 64 % yield. Unfortunately, the final deprotection steps turned out to be more challenging than expected. The outcome of the first attempt, saponification of the methyl ester and subsequent simultaneous deprotection of both the Boc and TBS group with TFA, resulted in acid catalyzed γ‐lactone “backbite” and cleavage of the molecule.^[33b]^ Hence, an alternative deprotection strategy had to be found. HF/pyridine[^49^, ^50^, ^51^] was used as a milder deprotecting agent, which afforded the selective cleavage of TBS‐ether in a first step to obtain **21** in 36 % yield after flash chromatography. Despite careful reaction control, partial cleavage of the molecule due to the formation of the lactone could not be avoided. Saponification of **21** with lithium hydroxide and subsequent treatment of crude **22** with zinc bromide and ethanethiol in 2,2,2‐trifluoroethanol (TFE) provided the final target molecule “HER” (**23**) in 44 % yield (2 steps) after preparative HPLC.

**Figure 3:**
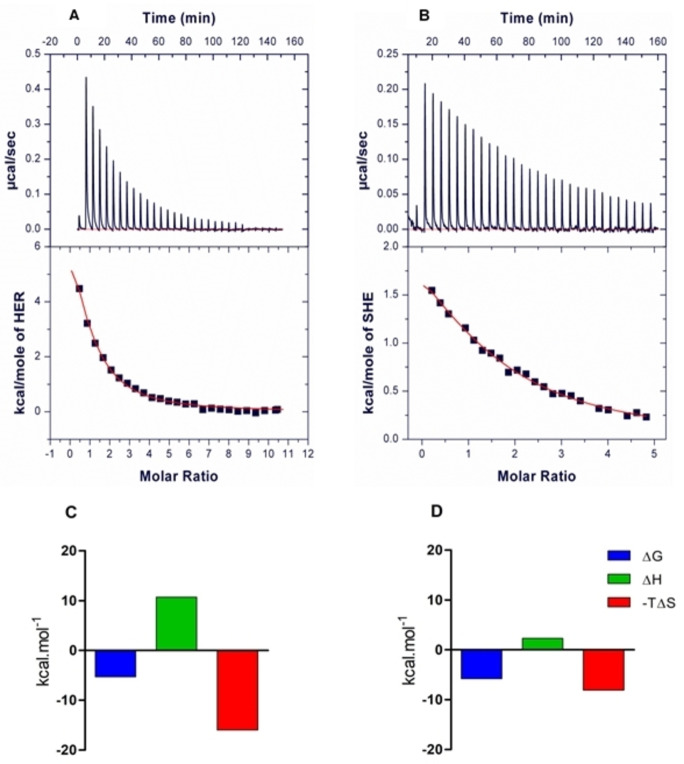
ITC measurement curves of DPP3 with (A) HER with *K*_d_ = 11±1 μM and (B) SHE with *K*_d_ = 23±4 μM. The bottom panel shows the thermodynamic signatures for (C) HER and (D) SHE derived from the ITC experiment. The mean Δ*G*, Δ*H*, ‐*T*Δ*S* values for HER are −5.3, 10.7 and −16.0 kcal/mol respectively, whereas the Δ*G*, Δ*H*, ‐*T*Δ*S* values for SHE are −5.8, 2.3 and −8.1 kcal/mol respectively. The data represents mean of three independent measurements.

With the two epimers of our target structures in hand, we could set out to characterize their potential as inhibitors of DPP3. Inhibition potencies of both SHE (**10**) and HER (**23**) were investigated via fluorescence‐based competitive inhibition assay of degradation of the H‐Arg‐Arg‐βNA substrate. IC_50_ values were calculated based on the resulting dose response curves. Both transition state mimetics inhibited hDPP3. SHE inhibited the enzyme with an IC_50_ of 98.5 μM, while inhibition with HER resulted in an IC_50_ of 13.8 μM, making it 7‐fold more potent than SHE. Importantly, HER could also efficiently inhibit DPP3 in mouse brain homogenate without losing much of its potency (Figure SI1). The inhibitory capacities of these compounds were corroborated by ITC measurements, where an endothermic binding was observed for both SHE and HER, similar to tynorphin. While SHE could bind to hDPP3 in vitro with an affinity of *K*_d_=23 μM, the binding of HER to hDPP3 was stronger with a *K*_d_=11 μM (Figure 3). The thermodynamic signatures depicted in Figure 3 show that both SHE and HER are compounds, which show that the unfavorable enthalpy of binding is overcompensated by strongly entropy‐driven binding.[^52^, ^53^] Such behavior is very rarely observed, but has been reported previously for the thermodynamic signatures of the HIV‐protease inhibitors Indinavir and Nelfinavir.^[54]^

**Figure 4:**
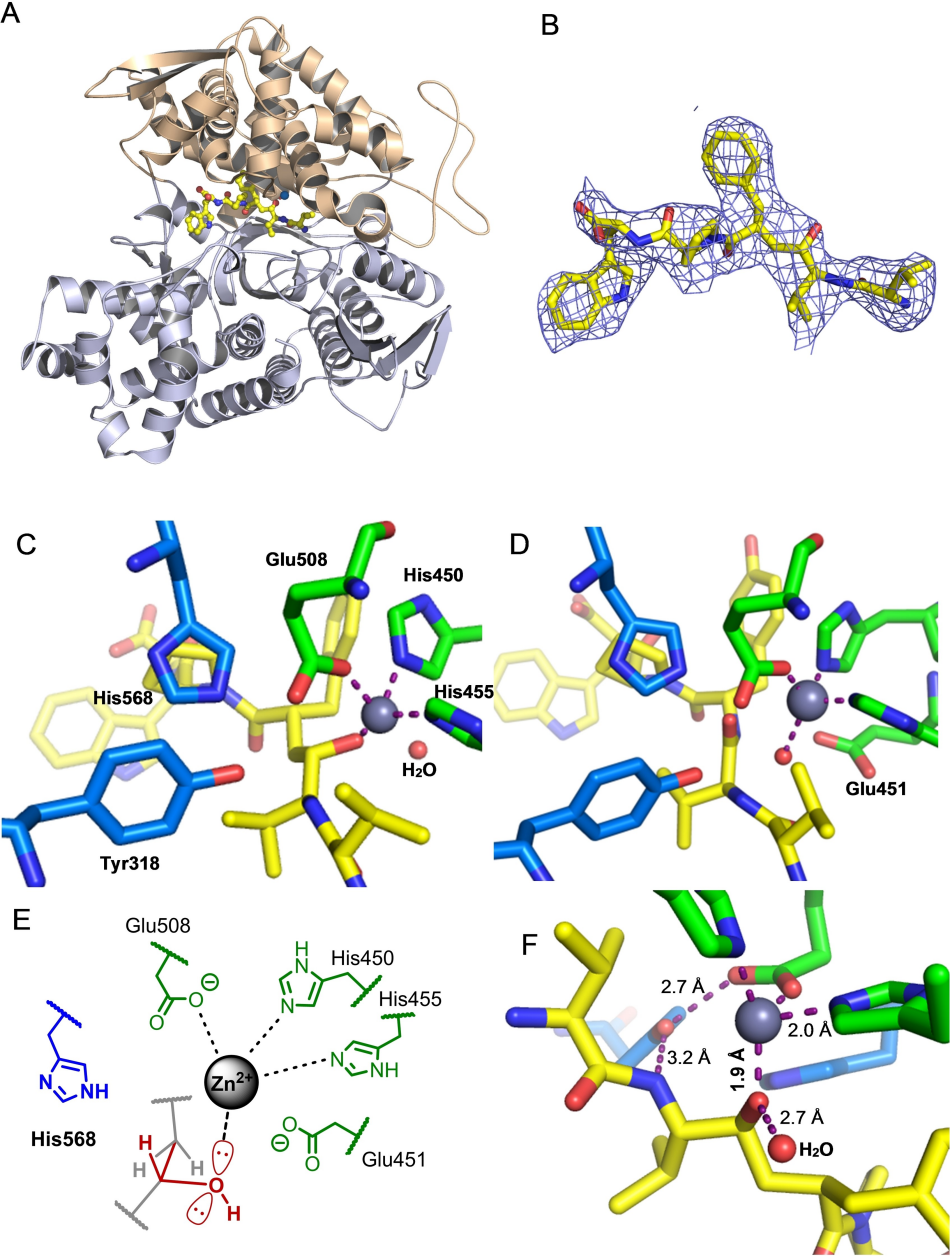
(A) Overall structure of the complex of human DPP3 (E451A‐variant) with the *(S)‐*hydroxyethylene‐peptidomimetic SHE. The two lobes of the enzyme are shown in light blue and brown, the bound ligand is depicted in a balls‐and‐sticks representation with carbon atoms colored yellow. The zinc ion in the active site is highlighted as a blue sphere. (B) Polder omit‐map^[56]^ around the bound inhibitor contoured at 3σ. The ligand is shown as a sticks‐representation and the electron density map is depicted a blue mesh. (C) Binding mode of SHE in the binding site of the inactive E451A mutant of hDPP3. (D) Binding mode of tynorphin in the binding site of the inactive E451A mutant of hDPP3.^[22]^ Glu451, Zn‐ion and the water molecule, missing out from the tynorphin‐hDPP3 structure, were computationally added and force field‐optimized using MOLOC software.^[23]^ (E) Initially proposed scheme of binding mode of SHE to the active site of the wild type hDPP3, having the catalytic Glu451 residue. (F) Measured hydrogen bonding and zinc coordinating interactions around the hydroxyethylene moiety in the SHE‐hDPP3 complex.

We were fortunate to crystallize a SHE/hDPP3 (E451A‐variant) complex and determine its structure with X‐ray crystallography (Figure 4A and 4B). The binding mode of SHE can be almost perfectly aligned with the binding mode of tynorphin in the previously obtained structure of tynorphin‐hDPP3 complex, the main differences being around the catalytic zinc‐complex. The *C*‐terminal part of the ligand is bound in a cation‐π complex of the indole moiety of SHE with Lys670 and Arg669 of hDPP3. Arg669 also forms a salt bridge to the carboxylate group of SHE. The equivalence to the tynorphin‐hDPP3 complex is also apparent at the N‐terminus of SHE, which is also tightly bound by the same three hydrogen bonds (Glu316, Asn394, Asn391) and the salt bridge to the Glu316.

In contrast to the binding mode of tynorphin, the hydroxyethylene group in SHE does not interact with His568, which most probably presents a significant penalty to the enthalpy of binding (Figure 4C and 4D). On the other hand, based on the initial rough model of binding of *(S)‐*hydroxyethylene type of inhibitor (Figure 4E), the hydroxyl substituent complexes as expected to the zinc ion, which was confirmed by measurement of the bond length from the crystallographic data. The Zn−O bond was found to be 1.9 Å (Figure 4F), which falls into the range of 1.9–2.4 Å for values typically observed for the length of Zn−O coordinating bonds in literature.^[55]^ Another interesting feature is a water molecule (Figure 4C and 4F) hydrogen bonded to the hydroxyethylene, which occupies the space where normally the carboxylate from Glu451 would be positioned within the active hDPP3 (Figure 4D). Unfortunately, we could not yet achieve an X‐ray structure of the HER/DPP3 complex, as we were not able to obtain crystals of this complex. However, from the good concordance of our models with the experimental structures of the complex with SHE, we are quite confident that also the proposed structure of the complex with HER in Figure 2 explains the observed higher affinity of this ligand to DPP3.

**Figure 5:**
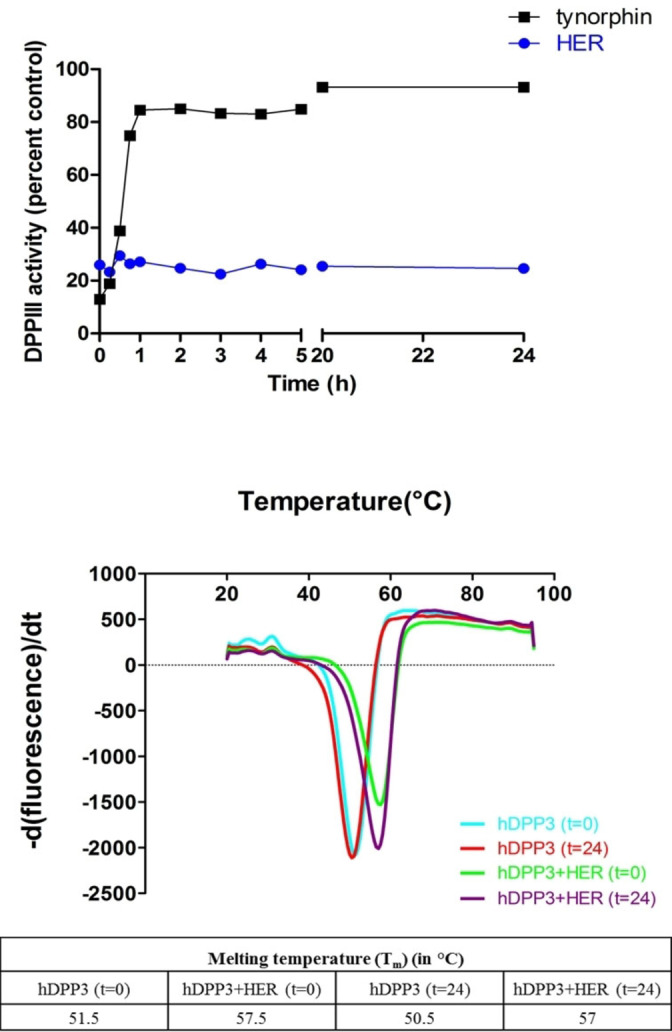
Top: Time‐dependent inhibition of DPP3 by tynorphin (black rectangles) and HER (blue circles). The x‐axis represents incubation time in hours and the y‐axis represents the percentage of DPP3 activity compared to control. Bottom: Thermal transitions (upper panel) and melting temperatures of hDPP3 in the presence and absence of HER (taken at two time points: 0 and 24 h of incubation) determined by Thermofluor™.

In order to address the question, that the hydroxyethylene isostere also leads to a stable inhibitor of DPP3 we performed a comparison of time‐dependent inhibition of hDPP3 by HER and tynorphin, which revealed that HER is not degraded by the peptidase and therefore can efficiently inhibit the activity over a period of 24 h. In contrast, tynorphin being a slowly converted substrate was purportedly cleaved by hDPP3 and thereby lost its efficacy within the first hour (Figure 5 top). This was also confirmed by a thermal stability assay where protein stabilization due to binding of HER, indicated by an increase in the protein melting temperature, was retained even after 24 h (Figure 5 bottom).

**Scheme 5:**
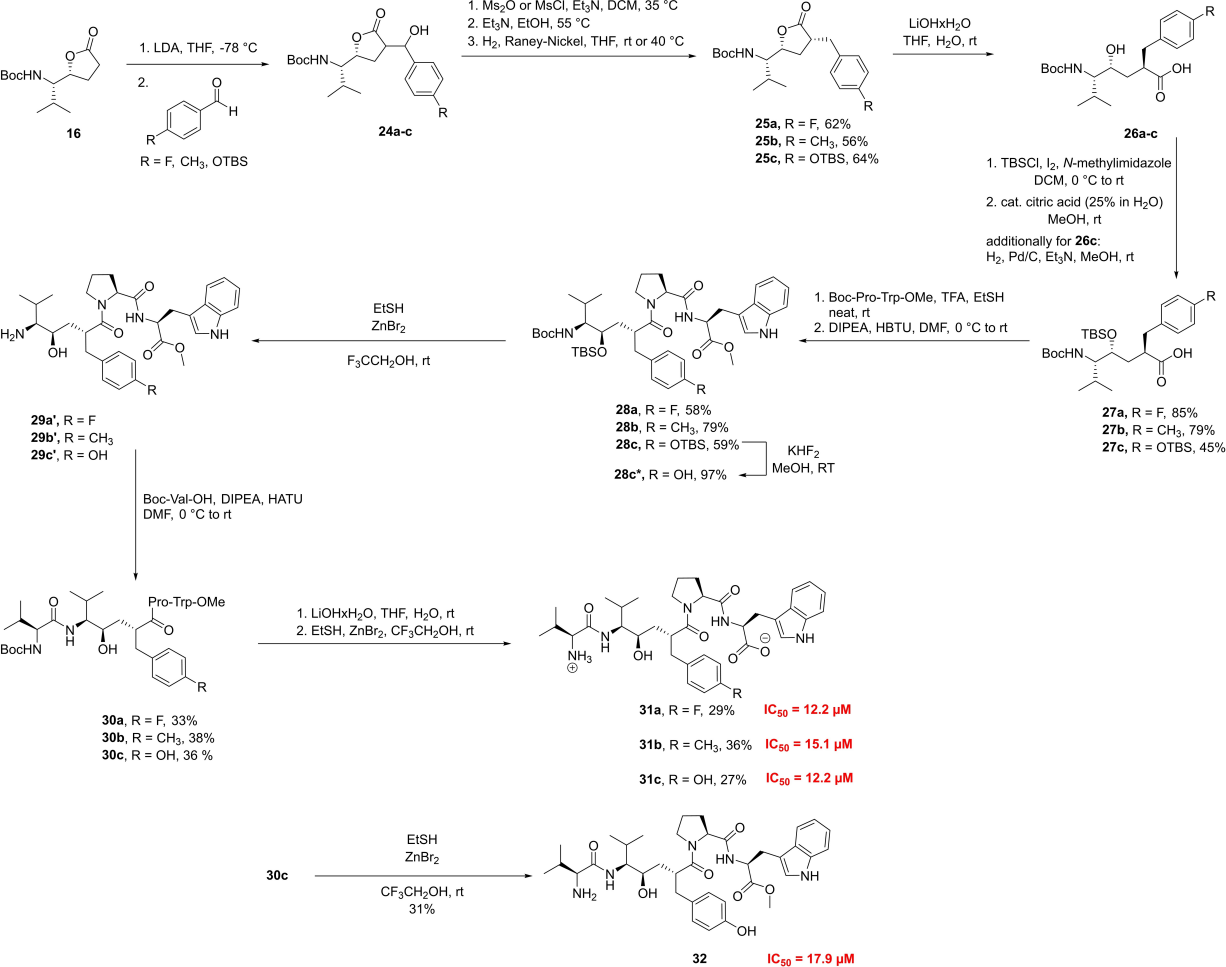
Synthesis of various pseudopeptide analogs and their IC_50_ values.

With these experiments we have shown that HER (**23**) is a suitable inhibitor of DPP3, which fulfills the criteria of selectivity and stability nicely confirming our design rationale. In a next step we sought out to use the underlying *(R)‐*hydroxyethylene scaffold for the synthesis of additional variants in which we varied the Phe‐analog of the pseudopeptide core to include the Tyr‐motif of the tynorphin lead structure, as well as F‐ and CH_3_‐substitutents in para‐position of the Phe‐group. The synthesis followed the route established for HER (**30**) starting with the aldol condensation of γ‐lactone **11** with the respective benzaldehydes (Scheme 5). While performing the TBS‐protection of compound **26 c**, an unexpected side reaction was observed using the silylation procedure of Stawinski.^[39]^ The presence of iodine during the protection procedure allowed the iodination of the aromatic ring leading to an almost 1 : 1 mixture of **26 c** and diiodinated isomer 26 c*, which could not be separated by flash chromatography. Fortunately, when the mixture of compounds 26 c* and **26 c** was treated with H_2_, Pd/C in the presence of triethylamine in methanol, the desired compound **27 c** was isolated in acceptable 45 % yield after an ultimate four‐step sequence (Scheme 5).

With all the pseudodipeptide fragments in hand, the core structures were now ready for elongation at the *C*‐terminus with the corresponding dipeptides under standard coupling conditions leading to the tetrapeptides **28 a**–**c**. The subsequent N‐terminal elongation with the characteristic Val residue started with the Boc‐deprotection of the tetrapeptide using TFA, followed by coupling of the Val residue under standard conditions with HATU as coupling additive and DIPEA as base in DMF leading to pentapeptides **30 a**‐**b**. In the case of the Tyr‐pseudotetrapeptide **28 c**, the aryl silyl ether had first to be cleaved off. Following the protocol for selective cleavage of phenol silylethers of Lakshman^[57]^ deprotection of **28 c** with KHF_2_ in methanol led to Tyr‐analog 28 c* in excellent yield. The already established treatment with ZnBr_2_/EtSH in TFE led to the successful tandem deprotection to compound **29 c**. Subsequent coupling with Boc‐Val‐OH provided desired compound **30 c** in 36 % overall yield. Finally, using the established twofold deprotection strategy saponification and *N*‐Boc deprotection delivered the target compounds **31 a–c**, which showed similar activity in the DPP3‐inhibition assay (IC_50_ values of 12–15 μM) as has been observed for HER, confirming the structural significance of the *(R)‐*hydroxyethylene subunit, while the para‐phenyl substituent in this moiety plays only a minor role. In addition to the already desired compounds, the methyl ester **32** was synthesized, in which only the amino group of **30 c** was deprotected under already established conditions. **32** still showed remarkable inhibition activity of DPP3 (IC_50_=17.9 μM), offering opportunities for improving bioavailability for this less polar peptide.

**Scheme 6:**
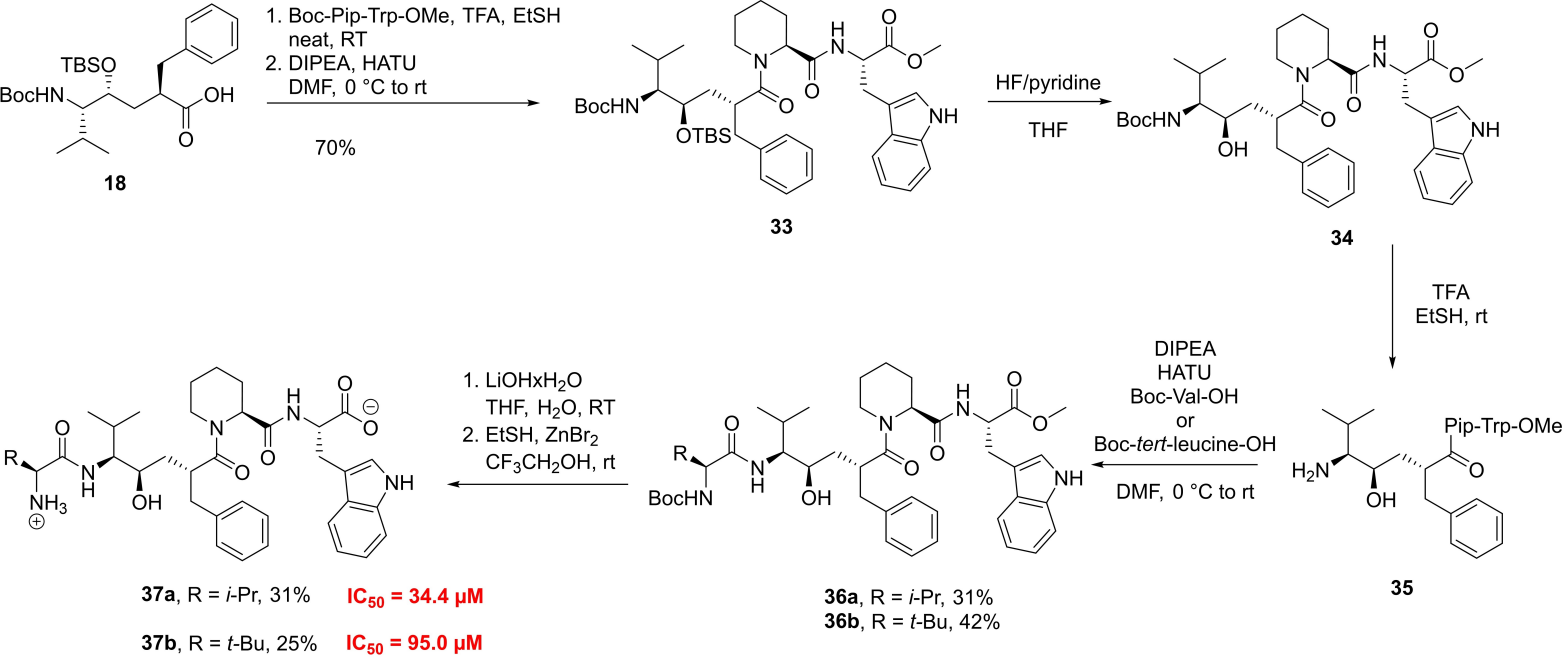
Synthesis of *(R)‐*hydroxyethylene inhibitor with pipecolic acid and its IC_50_ values.

In order to study the influence of the Pro on the conformation and activity of the peptide inhibitors, we synthesized a set of compounds using the larger pipecolic acid instead of Pro (Scheme 6). For the coupling of **18** with the Pip‐Trp‐OMe dipeptide fragment, the more powerful coupling agent HATU^[58]^ had to be used instead of HBTU, as the pipecolic acid with its piperidine moiety is less nucleophilic than proline.^[59]^ This, what could be believed to be only a minor change in structure, also made adaptations in the deprotection strategy necessary, as the conditions established for the Pro‐containing peptide (Scheme 5) resulted in amide cleavage for the pipecolic containing peptide. The observed degradative lactonization only led to the isolation of the lactone and the dipeptide fragment H‐Pip‐Trp‐OMe. In the need of a new deprotection strategy, we tried different additives for selective deprotection. Interestingly, HF/pyridine in THF, which showed being not successful for the silyl ether cleavage of the Pro‐containing compounds **26 a** and **26 b**, emerged for the pipecolic substrate **33** as the reagent of choice and selective and quick deprotection of the hydroxyl moiety of **33** could be observed after 1 h, according to NMR. *N*‐Boc‐deprotection of **34** was achieved by using TFA in ethanethiol and subsequent coupling with either Boc‐Val‐OH or Boc‐*tert*‐leucine‐OH provided compounds **36 a** and **36 b** in 31 % and 42 % overall yield respectively. Saponification of the methyl ester with LiOHxH_2_O and removal of the *N*‐Boc group with ZnBr_2_/EtSH in TFE produced the pipecolic acid derivatives **37 a‐b** (Scheme 6). In the DPP3 inhibition assay, compound **37 a**, which can be considered as a Pip‐analog of **HER**, showed a strongly increased IC_50_ of 34.4 μM. Replacing the N‐terminal Val with the bulkier tert‐butyl leucine resulted in an even more diminished inhibitory activity (IC_50_=95.0 μM). Together these results suggest that the original design of HER which was inspired by the amino acid sequence of tynorphin represents already a good starting point for further optimization which would profit from a high resolution crystal structure of the HER/DPP3 complex, where new interactions for improving the enthalpy of binding could be found or where conformational restrictions could be introduced which would increase the favorable entropic contribution of binding^[60]^ even further.

## Conclusions

Starting from the pentapeptide tynorphin (VVYPW), which is a substrate “inhibitor” slowly converted by DPP3, we designed pseudopeptide inhibitors of DPP3, directed by observations of preferred non‐covalent interactions in the crystal structure of the tynorphin‐hDPP3 complex. In order to convert tynorphin from being a slowly converted substrate to a true inhibitor of DPP3, a non‐cleavable hydroxyethylene isostere was used to replace the scissile peptide bond. We could show that among the two possible epimers the *(R)‐*hydroxyethylene pseudopeptide showed significantly better inhibition activity supporting our model about the active site interaction. Kinetic and thermodynamic characterization of the resulting inhibitors confirmed their inhibitory properties on hDPP3 and revealed that these inhibitors exhibit a strongly entropy driven binding behavior compensating for a positive binding enthalpy. Such a thermodynamic signature of binding has only very rarely been observed before, most notable for the HIV‐protease inhibitors Indinavir and Nelfinavir.

We could also demonstrate the long‐term stability of these peptidomimetics in assays in contrast to peptide “inhibitors” of DPP3, such as tynorphin, which are degraded by this enzyme within a short time. These are the first hydroxyethylene transition state mimetic inhibitors that demonstrably inhibit a metalloprotease and might provide a guidance that this type of transition state mimetics should be considered for the future design of inhibitors of other metalloproteases and ‐hydrolases.

The insights gained by the characterization of this new class of inhibitors will provide a starting point for the design of molecular tools specific for inhibiting hDPP3 and pave the way to exploit this enzyme as a potential drug target for pain intervention strategies, control of blood pressure, and treatment of septic shock.

## Experimental Section

Experimental details describing the synthesis and spectroscopic characterization of the compounds and details about the biophysical characterization are described in the Supporting Information. All animal experiments were approved by the Austrian Federal Ministry for Science, Research, and Economy (protocol number BMWF‐66.007/7‐ll/3b/), the ethics committee of the University of Graz, and conducted in compliance with the Council of Europe Convention (ETS 123).

Deposition Number(s) 2098559 (for **16**) contain(s) the supplementary crystallographic data for this paper. These data are provided free of charge by the joint Cambridge Crystallographic Data Centre and Fachinformationszentrum Karlsruhe Access Structures service.

## Conflict of interest

The authors declare no conflict of interest.

## Supporting information

As a service to our authors and readers, this journal provides supporting information supplied by the authors. Such materials are peer reviewed and may be re‐organized for online delivery, but are not copy‐edited or typeset. Technical support issues arising from supporting information (other than missing files) should be addressed to the authors.

Supporting Information
