# Supplementary material for: Efficient Entropy‐Driven Inhibition of Dipeptidyl Peptidase III by Hydroxyethylene Transition‐State Peptidomimetics
Source: Chemistry. 2021 Aug 31;27(56):14108–20. doi: 10.1002/chem.202102204 (PMC8518066; doi:10.1002/chem.202102204)

Chemistry–A European Journal

Supporting Information

Efficient Entropy-Driven Inhibition of Dipeptidyl Peptidase III by Hydroxyethylene Transition-State Peptidomimetics

Jakov Ivkovic, Shalinee Jha, Christian Lembacher-Fadum, Johannes Puschnig, Prashant Kumar, Viktoria Reithofer, Karl Gruber, Peter Macheroux,* and Rolf Breinbauer*

1 Supplementary Figures

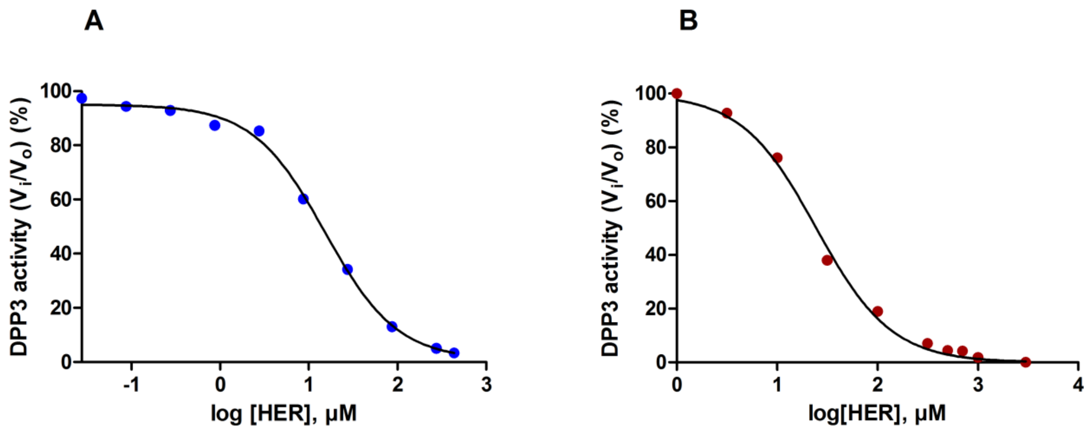

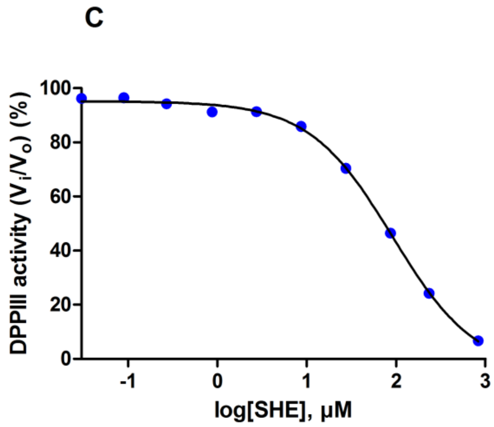

IC_{50} values (µM ± SD)	
hDPP3 (HER)	13.8 ± 1.4
Brain homogenate (HER)	26.7 ± 3.2
hDPP3 (SHE)	98.5 ± 6.5

Figure SI1 (A) In vitro and (B) ex vivo dose-response curves demonstrating DPP3 inhibition by HER. (C) Dose-response curve demonstrating DPP3 inhibition by SHE. Data is represented as mean values obtained from a minimum of three independent experiments (SD: standard deviation).

2 Experimental section

2.1 General methods of work

All reactions with moisture sensitive reagents were carried out under inert atmosphere with standard Schlenk techniques and dry solvents. Glassware for the experiments was dried using an oil pump vacuum (10^{-2} to 10^{-3} mbar) and heating with a heat gun. After cooling to room temperature, the glassware was purged with nitrogen or argon to obtain oxygen- and moisture-free conditions. Solvents and reagents were added under argon or nitrogen counter-stream. HPLC, GC and TLC samples of the reaction mixture were taken using a glass pipette under argon or nitrogen counter-stream. Temperatures were measured externally unless otherwise stated. For each reaction a Teflon® coated magnetic stirring bar was used for stirring.

Safety for the workup of hydrogenation reactions

Hydrogenation catalysts were carefully removed under argon atmosphere by filtration through a Schlenk-frit containing a plug of Celite®. The plug was first rinsed with the solvent of the reaction, then with a water-miscible solvent and finally with H_2O. The remaining slurry was stored under water and disposed as hazardous waste.

Chemicals and reagents

All commercially available chemicals and reagents were obtained from the following companies: Acros Organics, Aldrich, Alfa Aesar, Fisher Scientific, Fluka, Merck, Novabiochem, Roth, Sigma-Aldrich and VWR. Reagents were used without further purification, unless otherwise stated.

Benzaldehyde: Benzaldehyde was obtained from Fluka and purified by vacuum distillation. The freshly prepared aldehyde was stored in a Schlenk flask under argon at 5 °C.

***n*-Butyllithium:** *n*-BuLi was obtained from Aldrich as a 2.5 M solution in hexanes. The exact concentration was determined by titration using the procedure of Kofron and Baclawski.[1] 90.0 mg (424 µmol) diphenylacetic acid were dissolved in 1 mL abs. THF in a dry 8 mL Schlenk tube with magnetic stirring bar under argon. To the stirred colorless solution, *n*-BuLi solution was added with a syringe through a septum until the color changed to yellow. The titration was repeated three times and the concentration was calculated using the mean value of the consumed *n*-BuLi solution.

1,1’-Carbonyldiimidazole: CDI was obtained from Aldrich and recrystallized from abs. THF using the procedure published by Staab and Wendel.[2] A dry 250 mL three-necked round bottom flask with a Schlenk adapter, a reflux condenser, a gas bubbler and a magnetic stirring bar (evacuated, heated, N_2-purged) was charged with a suspension of 25.0 g (154 mmol) CDI

in 40 mL abs. THF and stirred. The suspension was heated to reflux (oil bath) and 20 mL abs. THF were added to enable full dissolution. Stirring was stopped and the pale yellow solution was allowed to cool down to RT for 60 min. Crystallization was completed by cooling to 0 °C (ice bath) for additional 30 min. The white crystals were collected by filtration through a glass frit under nitrogen atmosphere, washed with ice-cold abs. THF (15 mL) and dried *in vacuo*. 19.9 g (123 mmol, 80 %) of the recovered material was stored in a Schlenk flask under argon at –30 °C.

Molecular sieves: 3Å and 4Å molecular sieves (Sigma Aldrich, beads 8–12 mesh) were activated by heating them in a round bottom flask with a heat mantle (level 1) under high vacuum for 24 h. Activated molecular sieves were stored at RT under argon atmosphere.

Solvents

All solvents were purchased from the companies mentioned above and were used without further purification unless otherwise stated. For reactions where moisture and oxygen were excluded, absolute solvents were used. For that purpose the purchased solvents were dried by using the following methods and stored in brown 1 L Schlenk bottles under argon and over activated molecular sieves. For analytical applications solvents with analytical grade were purchased.

Chloroform: For purposes where complete dryness was not necessary, $CHCl_3$ was distilled using a rotary evaporator and stored in a brown glass bottle.

Dichloromethane: DCM (stabilized with EtOH) was first heated under reflux over P_4O_{10} for 12 h, then over CaH_2 for 2 d and distilled under argon atmosphere into a brown 1 L Schlenk bottle with activated 4Å molecular sieves.

Diethyl ether: Et_2O (for purposes where complete dryness was not necessary) was distilled using a rotary evaporator and stored in a brown bottle over KOH.

***N,N*-Dimethylformamide:** DMF was purchased in extra dry quality from Alfa Aesar and transferred into a brown 1 L Schlenk bottle with activated 3Å molecular sieves and stored under argon.

Ethanol: EtOH was purchased from Merck (99 %, containing 1 % methylethyl ketone) and heated under reflux together with sodium and diethyl phthalate in an inert distillation apparatus under argon for 2 h. Subsequently, the dry ethanol was distilled and stored over activated 3Å molecular sieves in a brown 1 L Schlenk bottle under argon.

Methanol: MeOH was heated under reflux over magnesium turnings and then distilled under argon atmosphere into a dry 1 L Schlenk bottle with activated 3Å molecular sieves.

Tetrahydrofuran: THF was heated under reflux over sodium for 21 h until the added benzophenone indicated dryness by turning color from green to blue. The dried THF was distilled into a 1 L Schlenk flask with activated 4Å molecular sieves and stored under argon.

Water: If water was used as a solvent in a reaction or for workup, deionized water from an ion exchanger was used.

2.2 Analytical methods

Thin-layer chromatography

Thin-layer chromatography was performed by using TLC-plates from Merck (TLC aluminium foil, silica gel 60, F_{254}). For detection of the spots a UV lamp with λ = 254 nm (fluorescence quenching) and/or staining with a reagent and subsequent development by heating with a heat gun was used. The following staining-reagents were used:

$KMnO_4$ (primarily used): 3.0 g $KMnO_4$ and 20 g K_2CO_3 were dissolved in 300 mL H_2O and 5 mL of 5 % aqueous NaOH were added.

CAM-solution: 2.0 g cerium(IV) sulfate, 50.0 g ammonium molybdate and 50 mL conc. H_2SO_4 in 400 mL water.

All used solvent mixtures, staining reagents and R_f values are stated in the experimental procedures.

Flash chromatography

Purification via flash chromatography was performed by using silica gel 60 from Acros Organics with a particle size of 35–70 µm. The mass of silica gel, depending on the separation problem, was a 30- to 100-fold amount (w/w) of the crude product. The length of the column was selected to get a pad of silica gel between 10 and 30 cm. All crude products were dissolved in a small amount of the mobile phase and applied in the dissolved form on the column. Solvent mixtures were chosen to adjust the R_f value of the product between R_f = 0.15 and R_f = 0.40. Solvents used for flash chromatography were purchased from Fisher Scientific as analytical grade solvents.

Gas chromatography

GC-MS

Analytical gas chromatography with mass detector was performed on an “Agilent Technologies 7890A GC System” with mass selective detector (EI, 70 eV; Agilent Technologies 5975C inert MSD with Triple-Axis Detector). Samples were injected by an autosampler (Agilent

Technologies 7683B Series Autosampler) in split mode (20/1; inlet temperature 280 °C). Attached capillary column was a polar HP-5ms column (30 m × 0.25 mm, layer thickness 0.25 µm) with helium 5.0 as carrier gas. Following methods were used:

JI_M300_S: 50 °C 2 min, ramp 40 °C/min linear to 300 °C, 5 min

JI_DMFcut_S: 50 °C 2 min, ramp 40 °C/min linear to 300 °C, 5 min

For the calculation of conversion, the integrated peak areas were used. Since no internal or external standard was used, these values are not exact.

GC-FID

Analytical gas chromatography with flame ionization detector (FID) for the separation of enantiomers was performed on an "Agilent Technologies 6890N GC System" with a CP-Chiralsil Dex CB capillary column (25 m × 0.32 mm, layer thickness 0.25 µm) with nitrogen 5.0 as carrier gas. Samples were injected by an autosampler (CTC Analytics CombiPAL) in split mode (5/1; inlet temperature 200 °C). The following method was used:

AMAL_GCPAL.M: 80 °C 5 min, ramp 10 °C/min linear to 150 °C, 18 min

The integrated peak areas were used to determine the ee of chiral compounds.

High-performance liquid chromatography

Analytical HPLC-MS

Analytical high-performance liquid chromatography was performed on an "Agilent Technologies 1200 Series" HPLC system with 1260 HiP Degasser G4225A, binary pump SL G1312, autosampler HiP-ALS SL G1367C, thermostated column compartment TCC SL G1316B, multiple wavelength detector G1365C MWD SL with deuterium lamp (λ = 190–400 nm) and subsequent connected mass detector (Agilent Technologies 6120 Quadrupole LC/MS) with an electrospray ionization (ESI) source. The components were separated on a RP Agilent Poroshell 120 SB-C18 column (3.0 × 100 mm, 2.7 µm) with a Merck LiChroCART® 4-4 pre-column. Signals were detected at 210 nm or 280 nm. As mobile phase acetonitrile (VWR HiPerSolv, HPLC-MS grade) and water (deionized and filtered through a 0.2 µm cellulose nitrate membrane filter) with 0.01 % formic acid were used. Following methods were used:

fast_Poroshell120_001HCOOH_10to95: 0-2.00 min: MeCN:H_2O = 10:90 (v/v), 2.00-10.00 min: linear increase to MeCN:H_2O = 95:5 (v/v), 10.00-16.00 min: holding of MeCN:H_2O = 95:5 (v/v), oven temperature: 40 °C, solvent flow: 0.700 mL/min.

FAST_POROSHELL120_001HCOOH_8MINGRADIENT.M: 40 °C, flow rate 0.7 mL/min; 0.0–2.0 min MeCN/H_2O = 10:90 (v/v), 2.0–10.0 min linear increase to MeCN/H_2O = 95:5 (v/v), 10.0–16.0 min hold MeCN/H_2O = 95:5 (v/v).

LONG_POROSHELL120_001HCOOH_40PCISOCRAT: 40 °C, flow rate 0.7 mL/min; 0.0–2.0 min MeCN/H_2O = 10:90 (v/v), 2.0–6.0 min linear increase to MeCN/H_2O = 40:60 (v/v), 6.0–12.0 min hold MeCN/H_2O 40:60 (v/v), 12.0–16.0 min linear increase to MeCN/H_2O = 95:5 (v/v), 16.0–22.0 min hold MeCN/H_2O = 95:5 (v/v).

Integrated peak areas were used for the calculation of conversion. Since no internal or external standard was used, these values are not exact.

Preparative HPLC

Isolation of polar compounds were performed on a "Thermo Scientific Dionex UltiMate 3000" system with UltiMate 3000 pump, UltiMate 3000 autosampler, UltiMate 3000 column compartment, UltiMate 3000 diode array detector (deuterium lamp, λ = 190–380 nm) and a UltiMate 3000 automatic fraction collector. The components were separated on a RP Machery-Nagel 125/21 Nucleodur® 100-5 C18ec column (21 × 125 mm, 5.0 µm). Signals were detected at 210 nm and 280 nm. As mobile phase acetonitrile (VWR HiPerSolv, HPLC grade) and water (deionized and filtered through a 0.2 µm cellulose nitrate membrane filter) with 0.01 % formic acid or 0.01 % trifluoroacetic acid were used. The following methods were used:

JKV_NucleodurC18_001HCOOH_10to85: 24 °C, flow rate 15 mL/min; 0.0–3.0 min MeCN/H_2O = 10:90 (v/v), 3.0–11.0 min linear increase to MeCN/H_2O = 85:15 (v/v), 11.0–13.0 min hold MeCN/H_2O = 85:15 (v/v).

CHRISTIAN_NucleodurC18_001CF3COOH: 24 °C, flow rate 15 mL/min; 0.0–3.0 min MeCN/H_2O = 10:90 (v/v), 3.0–11.0 min linear increase to MeCN/H_2O = 85:15 (v/v), 11.0–13.0 min hold MeCN/H_2O = 85:15 (v/v).

CLF_NucleodurC18_001HCOOH_5to90hold60: 24 °C, flow rate 15 mL/min; 0.0–5.0 min MeCN/H_2O = 5:95 (v/v), 5.0–13.0 min linear increase to MeCN/H_2O = 60:30 (v/v), 13.0–14.0 min hold MeCN/H_2O = 60:30 (v/v), 14.0–18.0 min linear increase to MeCN/H_2O = 90:10 (v/v), 18.0–20.0 min hold MeCN/H_2O = 90:10 (v/v).

Nuclear magnetic resonance spectroscopy

All NMR spectra were recorded with a Bruker AVANCE III spectrometer with autosampler at 300.36 MHz (^{1}H) and 75.53 MHz (^{13}C) or a Varian Inova NB high resolution spectrometer at 499.88 MHz (^{1}H) and 125.70 MHz (^{13}C). Chemical shifts δ are referenced to residual protonated solvent signals as internal standard. Signal multiplicities *J* are abbreviated as s (singlet), bs (broad singlet), d (doublet), dd (doublet of doublet), t (triplet) and m (multiplet). When it was necessary, additional 1D and 2D techniques (APT, COSY, HSQC, HMBC, NOESY, TOCSY, ^{19}F, ^{31}P) were recorded to verify the structure.

Deuterated solvents for NMR spectroscopy were purchased from Euriso-top®.

Melting points

Melting points were measured on a MEL-TEMP® apparatus with integrated microscopical support from Electrothermal in open capillary tubes. Reported values are uncorrected.

Optical rotation

A Perkin Elmer Polarimeter 341 was used for the determination of the specific rotation at λ = 589 nm (sodium D-line). Measurements were recorded at 23 or 24 °C and repeated at least three times.

High-resolution mass spectroscopy

High-resolution mass spectra were recorded using MALDI-TOF on a Micromass® MALDI micro MX™ spectrometer. Dithranol (1,8-dihydroxy-9,10-dihydroanthracen-9-one) or α-cyano-4-hydroxycinnamic acid served as matrix and PEG as internal standard. The stated values are m/z.

X-Ray crystallography of small molecule

X-Ray crystallography was performed by Prof. Roland Fischer (Institute of Anorganic Chemistry, Graz University of Technology), using the following procedure:

All crystals suitable for single crystal X-ray diffractometry were removed from a vial and immediately covered with a layer of silicone oil. A single crystal was selected, mounted on a glass rod on a copper pin, and placed in the cold N_2 stream provided by an Oxford Cryosystems cryostream. XRD data collection was performed on a Bruker APEX II diffractometer with use of Mo-K_α radiation (Incoatec Microsource *Iµ50*, λ = 0.71073 Å) and a CCD area detector. Empirical absorption corrections were applied using SADABS.[3,4] The structures were solved with use of direct methods in SHELXS and refined by the full-matrix least-squares procedures in SHELXL.[5] The space group assignments and structural solutions were evaluated using PLATON.[6]

Crystal structure determination of SHE/DPP3 ligand/protein complex

Crystals of the complex of hDPP3 with the inhibitor "SHE" were obtained using the sitting-drop vapour diffusion method employing the following crystallization conditions: 0.056 M sodium phosphate monobasic monohydrate, 1.344 M potassium phosphate dibasic, pH 8.2. The protein concentration was 8 mg mL^{-1} and the protein:ligand ratio was approximately 1:30. These crystals were allowed to grow for a month before being flash-cooled in liquid nitrogen without any additional cryoprotectant. Diffraction data were collected at beamline BM14 at the European Synchrotron Radiation Facility (ESRF, Grenoble/France). The data were processed

using the program XDS[7] and the structures were solved by molecular replacement using the program PHASER[8] with the structure of the complex with tynorphin (PDB-code: 3T6B) as search model. The structure was further refined using the program PHENIX.[9] The program COOT[10] was used for model fitting and real space refinement using σ_A-weighted 2Fo-Fc and Fo-Fc electron density maps. Rfree-values were computed from 5% randomly chosen reflections not used for refinement. The inhibitor could be placed into well-defined electron density in the active site of hDPP3, the occupancy of the ligand refined to 0.78. Coordinates and structure factors were deposited in the Protein Data Bank (PDB) under accession code 7OUP. Details of the data collection, processing and structure refinement are summarized in Table S1.

Table S1: Data collection and refinement statistics.

	**DPP3_SHE (PDB-code: 7OUP)**
Wavelength [Å]	0.95373
Resolution range [Å]	42.5 - 2.65 (2.75 - 2.65)
Space group	*C*2
Unit cell [Å, °]	119.34 105.60 64.86 90 93.7 90
Total reflections	77136 (7279)
Unique reflections	23086 (2244)
Multiplicity	3.3 (3.2)
Completeness (%)	98.56 (96.39)
Mean I/sigma(I)	8.03 (1.56)
Wilson B-factor	53.69
R-merge	0.1165 (0.7036)
R-meas	0.1391 (0.8407)
R-pim	0.07503 (0.4547)
CC1/2	0.993 (0.64)
CC*	0.998 (0.884)
Reflections used in refinement	23058 (2241)
Reflections used for R-free	1154 (112)
R-work	0.1971 (0.3060)
R-free	0.2530 (0.3637)
CC(work)	0.954 (0.789)

CC(free)	0.937 (0.712)
Number of non-hydrogen atoms	5871
macromolecules	5748
ligands	50
solvent	73
Protein residues	724
RMS(bonds)	0.003
RMS(angles)	0.59
Ramachandran favored (%)	96.95
Ramachandran allowed (%)	2.91
Ramachandran outliers (%)	0.14
Rotamer outliers (%)	4.44
Clashscore	6.48
Average B-factor	53.65
macromolecules	53.77
ligands	58.71
solvent	40.60

Statistics for the highest-resolution shell are shown in parentheses.

2.3 Synthetic Procedures

Synthesis of *SHE*

2.3.1 *tert*-Butyl *N*-[(2*S*)-3-methyl-1-oxobutan-2-yl]carbamate (2)

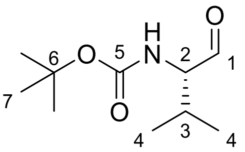

2

A 1000 mL two-neck round-bottom flask with a Schlenk adapter, a glass stopper and a magnetic stirring bar was heated, dried under vacuum and purged with N_2. Boc-L-Val-OH (10.864 g, 50.0 mmol, 1.0 eq) was added and dissolved in abs. dichloromethane (333 mL). The solution was cooled to 0 °C (ice bath) and 1,1'-carbonyldiimidazole (8.918 g, 55.0 mmol, 1.1 eq) was added. A gas bubbler was mounted instead of the glass stopper to allow pressure relief. After stirring for 60 min the gas bubbler was removed and the colorless reaction solution was cooled to -78 °C (CO_2/acetone bath) for 15 min. A septum was mounted instead of the glass stopper while maintaining a gentle counter flow of N_2. Subsequently, 1.0 M DIBAL-H solution in toluene (105 mL, 105 mmol, 2.1 eq) was added dropwise with a syringe through the septum throughout 110 min. The reaction mixture was stirred at -78 °C until TLC indicated quantitative conversion (45 min). The reaction mixture was quenched by the addition of EtOAc (335 mL). The acetone bath was removed, the gas bubbler was mounted, and 25% aqueous tartaric acid (222 mL) was added to the mixture under vigorous stirring. The mixture was warmed up by immersing the vessel into a water bath at RT and stirred vigorously for 15 min. The stirring was stopped and the layers were separated. The aqueous phase was extracted with EtOAc (333 mL) and the combined organic extracts were washed with 1 M HCl (222 mL), 0.8 M $NaHCO_3$ (222 mL) and brine (222 mL), dried over Na_2SO_4, filtered and concentrated under reduced pressure. The crude product was frozen in liquid nitrogen and was allowed to reach room temperature under high vacuum. The freeze-thaw procedure was repeated two times. Crude product (8.474 g, 42.10 mmol, 84 %) was furnished as a viscous colorless liquid, and used without further purification.

Yield: 8.474 g (42.10 mmol, 84 %), viscous colorless liquid.

$[\alpha]_D^{23}$ = + 78.6 ° (c = 1.07, CH_2Cl_2), lit. $[\alpha]_D^{20}$ = +82.1 ° (c = 1, CH_2Cl_2).

R_f = 0.61 (cyclohexane/ethyl acetate = 2:1 (v/v); staining: $KMnO_4$).

^{1}H NMR (300 MHz, $CDCl_3$) δ = 9.63 (s, 1H, H–C1), 5.15-4.99 (m, 1H, NH), 4.33-4.15 (m, 1H, H–C2), 2.37-2.14 (m, 1H, H–C3), 1.44 (s, 9H, H–C7), 1.02 (d, ^3J = 6.9 Hz, 3H, H–C4), 0.93 (d, ^3J = 7.0 Hz, 3H, H–C4).

^{13}C NMR (75 MHz, $CDCl_3$) δ = 200.5 (s, 1C, C1), 156.0 (s, 1C, C5), 80.1 (s, 1C, C6), 64.8 (s, 1C, C2), 29.2 (s, 1C, C3), 28.4 (s, 3C, C7), 19.2 (s, 1C, C4), 17.7 (s, 1C, C4).

GC-FID (CP-Chiralsil Dex): t_R((*S*)-**2**) = 9.0 min, 100%; t_R((*R*)-**2**) = 9.2 min, no abundance detected; ee > 99%.

2.3.2 Racemic *tert*-butyl *N*-[(2*S*)-3-methyl-1-oxobutan-2-yl]carbamate (*rac*-2)

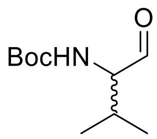

***rac*-2**

A 10 mL Schlenk tube, a glass stopper and a magnetic stirring bar was heated, dried under vacuum and purged with N_2. Boc-DL-Val-OH (43 mg, 0.20 mmol, 1.0 eq) was added and dissolved in abs. dichloromethane (1.3 mL). The solution was cooled to 0 °C (ice bath) and 1,1'-carbonyldiimidazole (36 mg, 0.22 mmol, 1.1 eq) was added. A gas bubbler was mounted instead of the glass stopper to allow pressure relief. After stirring for 60 min the gas bubbler was removed and the colorless reaction solution was cooled to -78 °C (CO_2/acetone bath) for 15 min. A septum was mounted instead of the glass stopper while maintaining a gentle counter flow of N_2. Subsequently, 1.0 M DIBAL-H solution in toluene (0.42 mL, 0.42 mmol, 2.1 eq) was added dropwise with a syringe through the septum throughout 10 min. The reaction mixture was stirred at -78 °C until TLC indicated quantitative conversion (60 min). The reaction mixture was quenched by the addition of EtOAc (1.3 mL). The acetone bath was removed, the gas bubbler was mounted, and 25% aqueous tartaric acid solution (1.0 mL) was added to the mixture under vigorous stirring. The mixture was warmed up by immersing the vessel into a water bath at RT and stirred vigorously for 15 min. The stirring was stopped and the layers were separated. The aqueous phase was extracted with EtOAc (1.3 mL) and the combined organic extracts were washed with 1 M HCl (1.0 mL), 0.8 M $NaHCO_3$ (1.0 mL) and brine (1.0 mL), dried over Na_2SO_4, filtered and concentrated under reduced pressure. The crude product was frozen in liquid nitrogen and was allowed to reach room temperature under high vacuum. The freeze-thaw procedure was repeated two times. The crude product (33 mg, 0.16 mmol, 80 %) was furnished as a colorless liquid.

Yield: 33 mg (0.16 mmol, 80 %), colorless liquid.

R_f = 0.61 (cyclohexane/ethyl acetate = 2:1 (v/v); staining: $KMnO_4$).

GC-FID (CP-Chiralsil Dex CP): t_R ((*S*)-**2**) = 9.0 min; t_R ((*R*)-**2**) = 9.2 min.

2.3.3 Ethyl (5*S*)-5-{[(*tert*-butoxy)carbonyl]amino}-4-hydroxy-6-methylhept-2-ynoate (3)

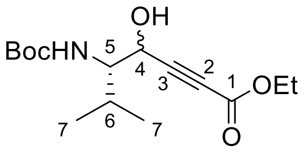

3

In an oven dried and nitrogen-purged 500 mL Schlenk vessel equipped with a Teflon®-coated magnetic stirring bar, 1-pentyne (7.87 mL, 79.8 mmol, 2.10 eq) was dissolved in 125 mL absolute THF and cooled to 0 °C in an ice bath. To the stirred solution 2.50 M *n*-BuLi in hexanes (30.40 mL, 76.0 mmol, 2.00 eq) was added dropwise via syringe and septum within 3 min. The yellow reaction solution was stirred and cooled for 15 min to -78 °C in an acetone/dry ice bath and subsequently ethyl propiolate (7.70 mL, 76.00 mmol, 2.00 eq) was added dropwise via syringe and septum. The orange reaction solution was stirred at -78 °C for 15 min and then a freshly prepared solution of aldehyde **2** (7.648 g, 38.00 mmol, 1.00 eq) in absolute THF (65 mL) was added dropwise within 10 min via a cannula by applying a gentle nitrogen overpressure from the donor vessel. The reaction was stirred at -78 °C until TLC indicated full conversion of the aldehyde (60 min). The orange reaction mixture was quenched by the dropwise addition of a solution of AcOH (10 mL) in THF (20 mL) and was brought to RT by immersion in a water bath and stirring for 15 min. The mixture was diluted with EtOAc (380 mL) and washed with 5% $NaHCO_3$ (2x100 mL) and brine (50 mL). The organic extract was concentrated under reduced pressure. Flash chromatography (SiO_2, cyclohexane/EtOAc 7:1) afforded a viscous orange liquid (9.515 g, 31.78 mmol, 84%) as a mixture of two diastereomers.

Yield: 9.515 g (31.78 mmol, 84%), viscous orange liquid.

R_f = 0.30 (cyclohexane/EtOAc 3:1 (v/v); staining: $KMnO_4$).

^{1}H NMR (300 MHz, $CDCl_3$, mixture of 2 diastereomers, based on COSY and HSQC) δ = 4.91 and 4.69 (d, ^3J = 8.6 Hz, 1H, *H*NCO), 4.58 (br s and d, ^3J = 4.8 Hz, 1H, H–C4), 4.28–4.14 (m, 2H, C*H*$_2$CH$_3$), 3.69–3.56 and 3.51–3.36 (m, 1H, H–C5), 2.20–2.01 and 1.88–1.73 (m, 1H, H–C6), 1.46 and 1.44 (s, 9H, (C*H*$_3$)$_3$), 1.34–1.25 (m, 3H, CH$_2$C*H*$_3$), 1.03–0.89 (m, 6H, H–C7).

^{13}C NMR (75 MHz, $CDCl_3$, mixture of 2 diastereomers, based on COSY and HSQC) δ = 157.6 and 157.1 (s, 1C, HNCO), 153.4 and 153.3 (s, 1C, C1), 86.2 and 84.8 (s, 1C, C3), 80.7 and 80.2 (s, 1C, (CH$_3$)$_3$*C*), 78.0 (s, 1C, C2), 64.9 and 64.0 (s, 1C, C4), 62.4 and 62.2 (s, 1C, *C*H$_2$CH$_3$), 61.4 and 60.8 (s, 1C, C5), 30.3 and 28.8 (s, 1C, C6), 28.4 (s, 3C, (*C*H$_3$)$_3$), 20.1 and 20.0 (s, 1C, C7), 19.2 and 18.5 (s, 1C, C7), 14.1 (CH$_2$*C*H$_3$).

2.3.4 ***tert*-Butyl *N*-[(1*S*)-2-methyl-1-[(2*S*)-5-oxooxolan-2-yl]propyl]carbamate (4) and *tert*-butyl *N*-[(1*S*)-2-methyl-1-[(2*R*)-5-oxooxolan-2-yl]propyl]carbamate (5)**

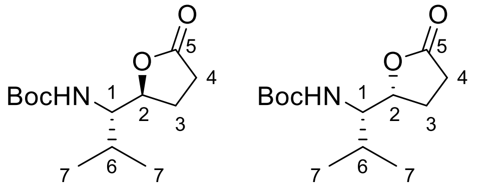

4 **5**

Hydrogenation: In a 250 mL round-bottom two-neck flask equipped with a Schlenk adapter, glass stopper and a Teflon®-coated magnetic stirring bar, **3** (9.400 g, 31.40 mmol, 1.00 eq) was dissolved in EtOAc (126 mL) and stirred at RT. The solution was degassed three times by alternate evacuation and filling with N_2 gas. 5% Pd/C (668 mg, 0.310 mmol, 0.01 eq) was added to the solution and a hydrogen balloon was mounted. The solution was purged three times by alternate evacuation and filling with H_2 gas. The black mixture was vigorously stirred in the H_2-atmosphere until complete consumption of starting material was indicated by TLC (20 h). The reaction flask was disconnected from the hydrogen balloon and purged with N_2. Under nitrogen atmosphere the content of the flask was transferred to the nitrogen-purged fritted Schlenk type funnel containing a 1.5 cm thick compressed bed of Celite. The product was eluted from the filter cake with EtOAc (3×13 mL). The Celite bed with the solid catalyst was washed with THF (5 mL) and water (5 mL), and stored under water in a container dedicated for catalyst waste. The product containing filtrate was concentrated under reduced pressure to furnish a yellow liquid residue.

Lactonization: In a 250 mL round-bottom flask equipped with a Teflon®-coated magnetic stirring bar, the yellow liquid residue was dissolved in toluene (126 mL) and *p*-TsOH×H_2O (60 mg, 0.31 mmol, 0.01 eq) was added. The pale yellow solution was stirred and heated at 50 °C (oil bath) for 2 h. The reaction solution was cooled to RT, washed with 5% $NaHCO_3$ (2×100 mL) and brine (100 mL), dried over Na_2SO_4 and concentrated under reduced pressure. Flash chromatography (SiO_2, cyclohexane/EtOAc 6:1 to 4:1) afforded two separated diastereomers: **4** (2.661 g, 10.34 mmol, 33%) as a viscous pale yellow liquid, and **5** (1.262 g, 4.904 mmol, 16%) as a pale yellow solid.

*Characterization of tert-butyl N-[(1S)-2-methyl-1-[(2S)-5-oxooxolan-2-yl]propyl]carbamate (***4***):*

Yield: 2.661 g (10.34 mmol, 33%, 2 steps), viscous pale yellow liquid.

$[\alpha]_D^{23}$ = -51.7 ° (c = 0.56, $CHCl_3$).

R_f = 0.31 (cyclohexane/EtOAc 2:1 (v/v); staining: $KMnO_4$).

^{1}H NMR (300 MHz, $CDCl_3$, based on HSQC) δ = 4.76–4.64 (m, 1H, H–C2), 4.57 (d, ^{3}J = 10.1 Hz, 1H, NH), 3.43 (m, 1H, H–C1), 2.50 (dd, ^{3}J = 9.4 Hz, 7.4 Hz, 2H, H–C4), 2.28–1.98 (m, 2H, H–C3), 1.90–1.73 (m, 1H, H–C6), 1.42 (s, 9H, $(CH_3)_3$), 1.06–0.89 (m, 6H, H–C7).

^{13}C NMR (75 MHz, $CDCl_3$, based on HSQC) δ = 177.5 (s, 1C, C5), 156.5 (s, 1C, HNCO), 80.0 (s, 1C, C2), 79.8 (s, 1C, Me_3C), 58.5 (s, 1C, C1), 31.5 (s, 1C, C6), 28.7 (s, 1C, C4), 28.4 (s, 3C, $(CH_3)_3$), 24.9 (s, 1C, C3), 19.8 (s, 1C, C7), 19.4 (s, 1C, C7).

*Characterization of tert-butyl N-[(1S)-2-methyl-1-[(2R)-5-oxooxolan-2-yl]propyl]carbamate (**5**):*

Yield: 1.262 g (4.904 mmol, 16%, 2 steps), pale yellow solid.

$[\alpha]_D^{23}$ = -7.6 ° (c = 1.00, $CHCl_3$).

m.p. = 100–105 °C.

R_f = 0.24 (cyclohexane/EtOAc 2:1 (v/v); staining: $KMnO_4$).

^{1}H NMR (300 MHz, $CDCl_3$, based on HSQC) δ = 4.43 (d, ^{3}J = 9.5 Hz, 1H, NH), 4.38–4.25 (m, 1H, H–C2), 3.74–3.51 (m, 1H, H–C1), 2.66–2.38 (m, 2H, H–C4), 2.36–1.94 (m, 3H, H–C3 and H–C6), 1.42 (s, 9H, $(CH_3)_3$), 1.06–0.75 (m, 6H, H–C7).

^{13}C NMR (75 MHz, $CDCl_3$, based on HSQC) δ = 177.0 (s, 1C, C5), 156.2 (s, 1C, HNCO), 79.9 (s, 1C, Me_3C), 79.8 (s, 1C, C2), 57.8 (s, 1C, C1), 28.4 (s, 3C, $(CH_3)_3$), 28.3–27.9 (m, 2C, C4 and C6), 25.2 (s, 1C, C3), 19.9 (s, 1C, C7), 15.7 (s, 1C, C7).

2.3.5 *tert*-Butyl *N*-[(1*S*)-1-[(2*S*,4*R*)-4-benzyl-5-oxooxolan-2-yl]-2-methylpropyl]carbamate (6)

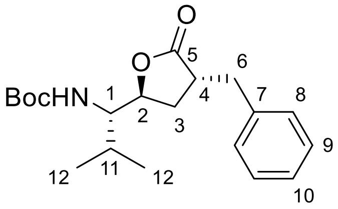

6

In an oven dried, argon purged 250 mL two-neck round-bottom flask, equipped with a dropping funnel, a gas valve adapter and a Teflon®-coated magnetic stirring bar, **4** (1.590 g, 6.228 mmol, 1.00 eq) was dissolved in THF (32.8 mL), stirred and cooled to -78 °C in an acetone/dry ice bath. 1.0 M LiHMDS solution in hexanes (12.8 mL, 12.8 mmol, 2.05 eq) was added dropwise within 5 min and the resulting solution was stirred for 30 min. A solution of benzyl bromide (741 µL, 6.23 mmol, 1.00 eq) in THF (32.8 mL) was charged into the dropping funnel, added dropwise to the reaction mixture within 10 min and the resulting orange reaction solution was stirred at -78 °C, until TLC indicated full conversion (55 min). The reaction mixture was poured into a vigorously stirred 3 M NH_4Cl aqueous solution (65.6 mL). The layers were separated and the aqueous layer was extracted with EtOAc (2×66 mL). The combined organic extracts were washed with 0.1 M HCl (33 mL), $NaHCO_3$ (33 mL), brine (11 mL), dried over Na_2SO_4, and concentrated and dried under reduced pressure. Flash chromatography (SiO_2, cyclohexane/EtOAc 8:1 to 5:1) furnished **6** (1.330 g, 3.828 mmol, 61%) as a colorless viscous liqiud.

Yield: 1.330 g (3.828 mmol, 61%), colorless viscous liquid.

$[\alpha]_D^{23}$ = -13.0 ° (c = 1.0, $CHCl_3$); lit. $[\alpha]$ not disclosed.

R_f = 0.60 (cyclohexane/EtOAc 2:1 (v/v); staining: $KMnO_4$).

^{1}H NMR (300 MHz, $CDCl_3$) δ = 7.40–7.11 (m, 5H, H–C8, H–C9 and H–C10), 4.55 (d, ^3J = 10.1 Hz, 1H, NH), 4.48–4.38 (m, 1H, H–C2), 3.42–3.29 (m, 1H, H–C1), 3.14 (dd, ^2J = 13.5 Hz, ^3J = 4.2 Hz, 1H, H_a–C6), 3.05–2.90 (m, 1H, H–C4), 2.82 (dd, ^2J = 13.5 Hz, ^3J = 8.9 Hz, 1H, H_b–C6), 2.27–2.12 (m, 1H, H_a–C3), 2.11–1.96 (m, 1H, H_b–C3), 1.86–1.69 (m, 1H, H–C11), 1.42 (s, 9H, $(CH_3)_3$), 0.94 (d, ^3J = 6.7 Hz, 6H, H–C12).

^{13}C NMR (75 MHz, $CDCl_3$) δ = 179.3 (s, 1C, C5), 156.4 (s, 1C, HNCO), 138.1 (s, 1C, C7), 129.0 (s, 2C, H–C9), 128.8 (s, 2C, H–C8), 127.0 (s, 1C, H–C10), 79.8 (s, 1C, Me_3C), 78.2 (s, 1C, C2), 59.0 (s, 1C, C1), 41.4 (s, 1C, C4), 37.0 (s, 1C, C6), 31.06 (s, 1C, C3), 30.0 (s, 1C, C11), 28.4 (s, 3C, $(CH_3)_3$), 19.8 (s, 1C, C12), 19.3 (s, 1C, C12).

2.3.6 (2*R*,4*S*,5*S*)-2-Benzyl-5-{[(*tert*-butoxy)carbonyl]amino}-4-[(*tert*-butyldimethylsilyl)oxy]-6-methylheptanoic acid (7)

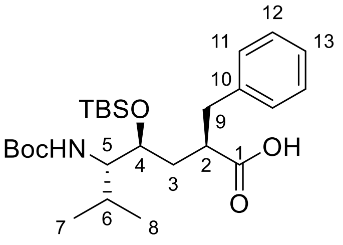

7

Lactone opening: In a 25 mL round-bottom flask, equipped with a Teflon®-coated magnetic stirring bar, **6** (500 mg, 1.44 mmol, 1.00 eq) was dissolved in THF (5.3 mL) and stirred vigorously. A 1 M solution of LiOH×H_2O (329 mg, 7.84 mmol, 4.00 eq) in H_2O (7.8 mL) was added dropwise from a syringe within 3 min. TLC indicated full conversion after 70 min. Et_2O (7.8 mL) was added and the biphasic mixture was cooled down to 0 °C in an ice bath. Under vigorous stirring the aqueous phase was carefully adjusted to pH = 4 with 25% aqueous citric acid. The layers were separated and the aqueous layer was extracted with Et_2O (2×7.8 mL). The combined organic extracts were washed with H_2O (5.0 mL), dried over Na_2SO_4, and concentrated and dried under reduced pressure and temperatures <30 °C to furnish a white solid substance.

Silylation: In a nitrogen-purged 25 mL Schlenk tube equipped with a Teflon®-coated magnetic stirring bar, the isolated white solid and *N*-methylimidazole (941 µL, 11.8 mmol, 6.00 eq) were dissolved in absolute CH_2Cl_2 (7.8 mL) and stirred. The solution was cooled to 0 °C in an ice bath and iodine (2.990 g, 11.8 mmol, 6.00 eq) was added. After stirring for 15 min TBSCl (0.886 g, 5.88 mmol, 3.00 eq) was added in portions within 1 min and the cooling bath was removed. TLC indicated full conversion after 14 h. The dark red mixture was transferred into a separation funnel, diluted with Et_2O (25 mL) and treated with saturated aqueous $Na_2S_2O_3$ (7.8 mL). The organic phase was washed with 25% citric acid (7.8 mL) and brine, concentrated under reduced pressure and dried *in vacuo* to yield a yellow oil.

Silyl ester methanolysis: In a 10 mL round-bottom flask equipped with a Teflon®-coated magnetic stirring bar, the yellow oil was dissolved in MeOH (1.0 mL) and 25% citric acid (20 µL) was added. The mixture was stirred until TLC indicated full conversion (22 h). The mixture was concentrated under reduced pressure and purified via flash chromatography (SiO_2, cyclohexane/EtOAc 4:1 to 2:1) to furnish **7** (383 mg, 0.798 mmol, 55% in 3 steps) as a colorless viscous liqiud.

Yield: 383 mg (0.798 mmol, 55%, 3 steps), colorless viscous liquid.

$[\alpha]_D^{23}$ = -17.2 ° (c = 1.0, $CHCl_3$); lit. $[\alpha]$ not disclosed.

R_f = 0.47 (cyclohexane/EtOAc/AcOH 2:1:0.05 (v/v); staining: $KMnO_4$).

^{1}H NMR (300 MHz, $CDCl_3$, based on HSQC) δ = 7.36–7.10 (m, 5H, H–C11, H–C12 and H–C13), 4.70 (d, ^{3}J = 10.1 Hz, 1H, NH), 3.89–3.78 (m, 1H, H–C4), 3.26–3.16 (m, 1H, H–C5), 3.11 (dd, ^{2}J = 13.5 Hz, ^{3}J = 7.3 Hz, 1H, H_a–C9), 2.87–2.76 (m, 1H, H–C2), 2.71 (dd, ^{2}J = 13.5 Hz, ^{3}J = 6.1 Hz, 1H, H_b–C9), 1.94–1.77 (m, 1H, H_a–C3), 1.72–1.54 (m, 2H, H_b–C3 and H–C6), 1.44 (s, 9H, $(C\mathit{H}_3)_3$CO), 0.92 (d, ^{3}J = 6.6 Hz, 3H, H–C7), 0.86 (s, 9H, $(C\mathit{H}_3)_3$CSi), 0.80 (d, ^{3}J = 6.6 Hz, 3H, H–C8), 0.04 (s, 3H, CH_3Si), 0.00 (s, 3H, CH_3Si).

^{13}C NMR (75 MHz, $CDCl_3$, based on HSQC) δ = 177.0 (s, 1C, C1), 157.7 (s, 1C, HNCO), 139.4 (s, 1C, C10), 129.1 (s, 2C, H–C12), 128.8 (s, 2C, H–C11), 126.7 (s, 1C, H–C13), 80.3 (s, 1C, $Me_3\mathit{C}$), 69.6 (s, 1C, C4), 58.8 (s, 1C, C5), 44.0 (s, 1C, C2), 38.1 (s, 1C, C9), 37.6 (s, 1C, C3), 29.6 (s, 1C, C6), 28.6 (s, 3C, $(\mathit{C}H_3)_3$CO), 26.0 (s, 3C, $(\mathit{C}H_3)_3$CSi), 20.0 (s, 1C, C7), 18.2 (s, 1C, C8), -3.7 (CH_3Si), -4.5 (CH_3Si).

2.3.7 *tert*-Butyl (2*S*)-2-{[(2*S*)-3-(1*H*-indol-3-yl)-1-methoxy-1-oxopropan-2-yl]carbamoyl}pyrrolidine-1-carboxylate (Boc-Pro-Trp-OMe)

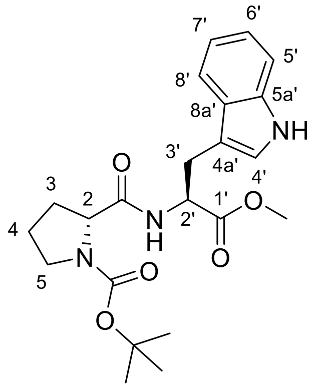

A 250 mL three-neck round-bottom flask, equipped with a Schlenk adapter, a dropping funnel and a Teflon®-coated magnetic stirring bar, was purged with nitrogen. In the flask, Boc-Pro-OH (6.458 g, 30.00 mmol, 1.00 eq) was dissolved in DCM (50 mL), stirred and cooled to 0 °C in an ice bath. Diisopropylcarbodiimide (5.637 mL, 36.00 mmol, 1.20 eq) was added and the resulting mixture was stirred for 10 min. A solution of H-Trp-OMe×HCl (7.641 g, 30.00 mmol, 1.00 eq) and triethylamine (8.363 mL, 60.00 mmol, 2.00 eq) in DCM (50 mL) was added dropwise within 5 min. The reaction mixture was stirred for 2 h and then concentrated under reduced pressure. The residue was dissolved in EtOAc (250 mL) and washed with 1 M HCl (2×125 mL), 0.1 M NaOH (3×125 mL), H_2O (100 mL) and brine (50 mL). The organic phase was dried over Na_2SO_4 and stored in a sealed flask overnight at 4 °C. Subsequently, the solution was cooled to -20 °C for 30 min and filtered to remove a white precipitate. The filtrate was concentrated and dried *in vacuo* to furnish a white solid substance (7.560 g, 18.20 mmol, 61%).

Yield: 7.560 g (18.20 mmol, 61%), white solid.

m.p. = 97–100 °C; lit. m.p. not disclosed.

$[\alpha]_D^{23}$ = -33.1 ° (c = 1.37, $CHCl_3$); lit. $[\alpha]$ not disclosed.

R_f = 0.26 (cyclohexane/EtOAc 1:1 (v/v); staining: $KMnO_4$).

^{1}H NMR (300 MHz, $CDCl_3$, based on HSQC) δ = 8.37 (br s, 1H, indole NH), 7.52 (d, ^3J = 7.7 Hz, 1H, H–C8’), 7.33 (d, ^3J = 7.7 Hz, 1H, H–C5’), 7.23–6.89 (m, 3H, H–C5’, H–C6’ and H–C7’), 6.51 (br s, 1H, HNCO), 4.89 (br s, 1H, H–C2’), 4.34–4.09 (m, 1H, H–C2), 3.67 (s, 3H, CH_3O), 3.42–3.03 (m, 4H, H–C3’ and H–C5), 2.31–1.52 (m, 4H, H–C3 and H–C4), 1.38 (s, 9H, $(CH_3)_3$CO).

^{13}C NMR (75 MHz, $CDCl_3$, based on HSQC) δ = 172.2 (m, 2C, CONH and H–C1’), 154.8 (s, 1C, CO_2t-Bu), 136.3 (s, 1C, C5a’), 127.7 (s, 1C, C8a’), 122.8 (s, 1C, C4’), 122.3 (s, 1C, C6’), 119.7 (s, 1C, C7’), 118.5 (s, 1C, C8’), 111.4 (s, 1C, C5’), 110.1 (s, 1C, C4a’), 81.2–79.9 (m,

1C, $Me_3\mathit{C}$), 61.6–59.6 (s, 1C, C2), 53.7–52.5 (m, 1C, C2’), 52.4 (s, 1C, $\mathit{C}H_3O$), 47.0 (s, 1C, C5), 30.7 (br s, 1C, C3), 28.3 (s, 3C, $(\mathit{C}H_3)_3CO$), 27.9 (s, 1C, C3’), 25.1–22.9 (m, 1C, C4).

HPLC-ESI-MS (Agilent Poroshell120; method: fast_Poroshell120_001HCOOH_10to95): t_R = 5.41 min, 100%, $[M + Na]^+$ = 438, $[M + K]^+$ = 454.

2.3.8 Methyl (2*S*)-2-{[(2*S*)-1-[(2*R*,4*S*,5*S*)-2-benzyl-5-{[(*tert*-butoxy)carbonyl]amino}-4-[(*tert*-butyldimethylsilyl)oxy]-6-methylheptanoyl]pyrrolidin-2-yl]formamido}-3-(1*H*-indol-3-yl)propanoate (8)

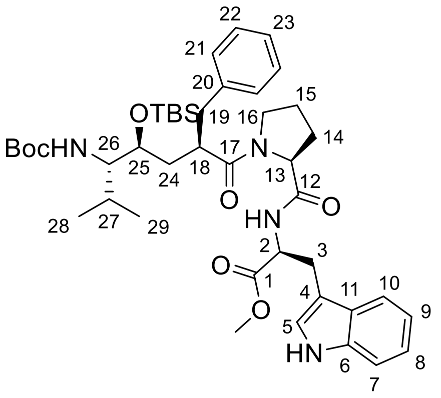

8

In a 5 mL glass vial equipped with a Teflon®-coated magnetic stirring bar, Boc-Pro-Trp-OMe (200 mg, 0.481 mmol, 1.20 eq) was dissolved in TFA (1.10 mL). Ethanethiol (357 µL, 4.77 mmol, 1.19 eq) was added and the solution was stirred for 60 min at RT. The volatiles were evaporated, the residue was dried in high vacuum to constant mass to yield deprotected H-Pro-Trp-OMe.

In a nitrogen-purged 10 mL Schlenk tube equipped with a Teflon®-coated magnetic stirring bar, **7** (189 mg, 0.400 mmol, 1.00 eq) and Hünig´s base (69 µL, 0.40 mmol, 1.0 eq) were dissolved in absolute DMF (1.60 mL). The solution was stirred, cooled to 0 °C in an ice bath and HBTU (180 mg, 0.47 mmol, 1.20 eq)) was added. Immediately after 5 min of activation a solution of the freshly prepared H-Pro-Trp-OMe and Hünig´s base (137 uL, 0.793 mmol, 1.98 eq) in absolute DMF (1.00 mL) was added via syringe and septum. The ice bath was removed and the mixture was stirred for 120 min. Subsequently, brine (2.6 mL) and EtOAc (8.0 mL) were added and the mixture was stirred vigorously for 5 min. The layers were separated and the organic phase washed with brine (3×2.6 mL), dried over Na_2SO_4, and concentrated under reduced pressure. After purification via flash chromatography (SiO_2, CH_2Cl_2/MeOH 80:1 to 20:1) **8** (231 mg, 0.297 mmol) was achieved as a white solid.

Yield: 231 mg (0.297 mmol, 74% from **7**), white solid.

m.p. = 76–79 °C.

$[\alpha]_D^{23}$ = -22 ° (c = 0.2, $CHCl_3$).

R_f = 0.38 (CH_2Cl_2/MeOH 20:1 (v/v); staining: $KMnO_4$).

^{1}H NMR (300 MHz, $CDCl_3$, based on COSY and HSQC) δ = 8.42 (br s, 1H, indole NH), 7.49 (d, ^3J = 7.6 Hz, 1H, H–C10), 7.35–6.98 (m, 10H, H–Ar and HNCO), 4.75–4.64 (m, 2H, $HNCO_2$ and H–C2), 4.57–4.54 (m, 1H, H–C13), 3.84 (dd, ^3J = 9.3 Hz, 3.3 Hz, 1H, H–C25), 3.68 (s, 3H,

C*H*$_3$O), 3.55–3.41 (m, 1H, H–C16), 3.31–3.20 (m, 2H, H–C3 and H–C26), 3.13 (dd, ^2J = 14.6 Hz , ^3J = 7.3 Hz, 1H, H–C26), 3.04–2.96 (m, 1H, H–C16), 2.86–2.83 (m, 1H, H–C18), 2.65 (dd, ^2J = 13.4 Hz, ^3J = 8.1 Hz, 1H, H–C19), 2.44 (dd, ^2J = 13.4 Hz, ^3J = 5.7 Hz, 1H, H–C19), 2.27–2.14 (m, 1H, H–C14), 1.87–1.55 (m, 6H, H_2–C15, H–C14, H_2–C24 and H–C27), 1.44 (s, 9H, (C*H*$_3$)$_3$C), 0.94 (d, ^3J = 6.7 Hz, 3H, H–C28), 0.87 (s, 9H, (C*H*$_3$)$_3$CSi), 0.83 (d, ^3J = 6.6 Hz, 3H, H–C29), 0.06 (s, 3H, CH_3Si), 0.02 (s, 3H, CH_3Si).

^{13}C NMR (75 MHz, $CDCl_3$, based on COSY, HSQC and HMBC) δ = 174.9 (s, 1C, C=O), 172.5 (s, 1C, C=O), 171.1 (s, 1C, C=O), 156.7 (s, 1C, *C*O$_2$*t*-Bu), 139.5 (s, 1C, C20), 136.3 (s, 1C, C6), 129.0 (s, 2C, C22), 128.7 (s, 2C, C21), 127.6 (s, 1C, C11), 126.7 (s, 1C, C23), 123.7 (s, 1C, C5), 122.1 (s, 1C, C8), 119.5 (s, 1C, C9), 118.7 (s, 1C, C10), 111.3 (s, 1C, C7), 110.1 (s, 1C, C4), 79.1 (s, 1C, Me$_3$*C*), 69.4 (s, 1C, C25), 59.9 (s, 1C, C13), 58.5 (s, 1C, C26), 53.4 (s, 1C, C2), 52.3 (s, 1C, *C*H$_3$O), 47.1 (s, 1C, C16), 42.4 (s, 1C, C18), 38.2 (s, 1C, C19), 36.6 (s, 1C, 24), 30.2 (s, 1C, C27), 28.5 (s, 3C, (*C*H$_3$)$_3$CO), 27.7 (s, 1C, C3), 27.3 (s, 1C, C14), 26.0 (s, 3C, (*C*H$_3$)$_3$CSi), 24.8 (s, 1C, C15), 20.0 (s, 1C, C28), 19.8 (s, 1C, C29), 18.2 (s, 1C, (CH$_3$)$_3$*C*Si), -3.66 (CH_3Si), -4.61 (CH_3Si).

HRMS (ESI): m/z (%): 777.4620 (87%, $[M + H]^+$, calcd for $C_{43}H_{65}N_4O_7Si^+$: 777.4617), 799.4441 (100%, $[M + Na]^+$, calcd for $C_{43}H_{64}N_4NaO_7Si^+$: 799.4442), 815.4184 (100%, $[M + K]^+$, calcd for $C_{43}H_{64}KN_4O_7Si^+$: 815.4181).

2.3.9 Methyl (2*S*)-2-{[(2*S*)-1-[(2*R*,4*S*,5*S*)-2-benzyl-5-[(2*S*)-2-{[(*tert*-butoxy)carbonyl]amino}-3-methylbutanamido]-4-hydroxy-6-methylheptanoyl]pyrrolidin-2-yl]formamido}-3-(1*H*-indol-3-yl)propanoate (9)

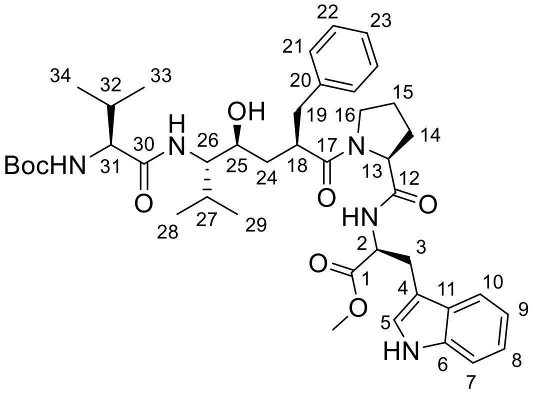

9

Deprotection: In a 5 mL glass vial equipped with a Teflon®-coated magnetic stirring bar, **8** (132 mg, 0.170 mmol, 1.00 eq) was dissolved in 2,2,2-trifluoroethanol (1.7 mL). Ethanethiol (51 µL, 0.68 mmol, 4.0 eq) and $ZnBr_2$ were added, and the solution was stirred for 4 h at RT, accompanied with formation of a white precipitate. Subsequently, the reaction mixture was treated with 25% aqueous ammonia (0.80 mL). After EtOAc (5.1 mL) was added, the mixture was transferred into a 20 mL Erlenmeyer flask and stirred vigorously for 5 min. The layers were separated, and the aqueous layer was extracted with EtOAc (2×3.4 mL). The combined organic extracts were dried over Na_2SO_4, concentrated under reduced pressure and the residue was dried in high vacuum to constant mass to yield the crude deprotected intermediate as a white amorphous solid (97 mg).

Coupling: In an oven-dried, nitrogen-purged 10 mL Schlenk tube, equipped with a Teflon®-coated magnetic stirring bar, Boc-Val-OH (44 mg, 0.20 mmol, 1.2 eq) and Hünig´s base (30 µL, 0.17 mmol, 1.0 eq) were dissolved in absolute DMF (0.70 mL) and stirred at RT. A solution of HATU (78 mg, 0.20 mmol, 1.2 eq) in abs. DMF (0.70 mL) was added and the reaction solution was stirred for 1 min before a solution of the deprotected intermediate and Hünig´s base (30 uL, 0.17 mmol, 1.0 eq) in abs. DMF (1.00 mL) was added. After TLC indicated full conversion of the intermediate (15 min), the reaction was quenched by addition of brine (1.0 mL) and extracted with EtOAc (3×3.6 mL). The combined organic extracts were washed with brine (3×1.0 mL), dried over Na_2SO_4, and concentrated under reduced pressure. Purification via flash chromatography (SiO_2, CH_2Cl_2/MeOH 50:1 to 20:1) furnished **9** (68 mg, 0.089 mmol) as a white solid.

Yield: 68 mg (0.089 mmol, 52%, 2 steps, from **8**), white solid.

m.p. = 90–93 °C.

$[\alpha]_D^{23}$ = -26.4 ° (c = 0.23, $CHCl_3$).

R_f = 0.51 (CH_2Cl_2/MeOH 10:1 (v/v); staining: $KMnO_4$).

^{1}H NMR (300 MHz, $CDCl_3$, complex mixture of signals of 2 rotamers in 1.2:1 ratio, assigned based on COSY, HSQC, HMBC and EXSY) δ = 9.19 and 8.63 (br s, 1H, indole NH), 7.60–7.47 (m, 1H, H–C10), 7.46–6.97 (m, 7.5H, H–Ar and amide H from *cis*-rotamer´s Trp), 6.54–6.31 (m, 1H, H–Ar), 6.12 (d, ^3J = 7.3 Hz, 0.5H, amide H from *trans*-rotamer´s Trp), 5.19–4.97 (m, 1H, carbamate H), 4.86–4.68 (m, 1H, H–C2), 4.58–4.27 (m and br s, 1.5H, H–C13 from one rotamer, and OH), 3.89–3.73 (m, 2H, H–C31 and H–C25), 3.76 and 3.68 (s, 3H, CH_3O), 3.56–2.38 (m, 8.5H, H_2–C3, H–C13 from one rotamer, H_2–C16, H–C18, H_2–C19, H–C26), 2.34–1.05 (m, 17H, H_2–C14, H_2–C15, H_2–C24, H–C27, H–C32 and $(CH_3)_3C$), 1.04–0.72 (m, 12H, H_3–C28, H_3–C29, H_3–C33 and H_3–C34).

^{13}C NMR (75 MHz, $CDCl_3$, complex mixture of signals of 2 rotamers, assigned based on COSY, HSQC, HMBC and EXSY) δ = 176.2 and 175.2 (s, 1C, C17), 172.9, 172.5, 172.4, 172.2, 172.1 and 170.7 (s, 3C, 2 amide C=O and an ester C=O), 156.2–156.0 (m, 1C, carbamate C=O), 139.1 and 138.8 (s, 1C, C20), 136.5 and 136.4 (s, 1C, C6), 128.9 and 128.8 (s, 2C, C22), 128.6 (s, 2C, C21), 127.6 and 127.5 (s, 1C, C11), 126.7 and 126.6 (s, 1C, C23), 123.8 and 123.0 (s, 1C, C5), 122.4 and 122.1 (s, 1C, C8), 119.8 and 119.5 (s, 1C, C9), 118.5 and 118.1 (s, 1C, C10), 111.8 and 111.4 (s, 1C, C7), 109.7 and 109.5 (s, 1C, C4), 80.3–79.9 (m, 1C, Me_3C), 67.3 and 67.0 (s, 1C, C25), 61.1–60.6 (m, 1C, C31), 60.5 and 59.8 (s, 1C, C13), 60.0 and 59.7 (s, 1C, C26), 53.4 and 53.2 (s, 1C, C2), 52.8 and 52.4 (s, 1C, CH_3O), 47.5 and 46.4 (s, 1C, C16), 43.3 and 42.7 (s, 1C, C18), 40.0 and 37.8 (s, 1C, C19), 37.8 and 35.9 (s, 1C, 24), 31.3 and 27.5 (s, 1C, C14), 30.4, 30.1 and 29.8 (s, 2C, C27 and C32), 28.7–28.1 (m, 3C, $(CH_3)_3CO$), 27.6 and 26.9 (s, 1C, C3), 24.9 and 22.0 (s, 1C, C15), 20.2–20.0, 19.9–19.6, 19.3–19.2 and 18.0–17.7 (m, 4C, C33, C34, C28, C29).

HPLC-ESI-MS (Agilent Poroshell120; method: SHE_Poroshell120_HCOOHMeCN_40_60_95): t_R(**9**) = 7.62 min, 100%, $[M + Na]^+$ = 785, $[M + K]^+$ = 801; t_R(***epi*-9**) = 7.79 min, no abundance detected; de >99%, based on comparison to the HPLC-ESI-MS trace of reference epimer mixture (*vide infra*).

HRMS (ESI): m/z (%): 784.4257 (100%, $[M + Na]^+$, calcd for $C_{42}H_{59}N_5NaO_8^+$: 784.4261).

2.3.10 Reference epimer mixture microsynthesis:

Methyl (2*S*)-2-{[(2*S*)-1-[(2*R*,4*S*,5*S*)-2-benzyl-5-[(2*S*)-2-{[(*tert*-butoxy)carbonyl]amino}-3-methylbutanamido]-4-hydroxy-6-methylheptanoyl]pyrrolidin-2-yl]formamido}-3-(1*H*-indol-3-yl)propanoate (9) and

Methyl (2*S*)-2-{[(2*S*)-1-[(2*R*,4*S*,5*S*)-2-benzyl-5-[(2*R*)-2-{[(*tert*-butoxy)carbonyl]amino}-3-methylbutanamido]-4-hydroxy-6-methylheptanoyl]pyrrolidin-2-yl]formamido}-3-(1*H*-indol-3-yl)propanoate (*epi*-9)

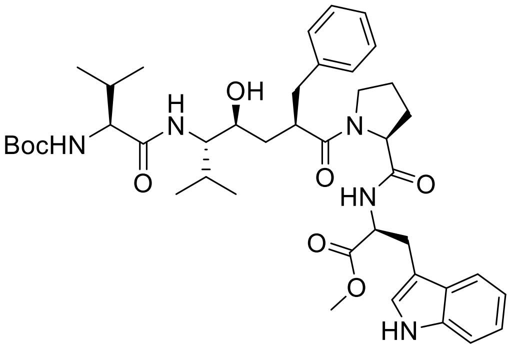

9

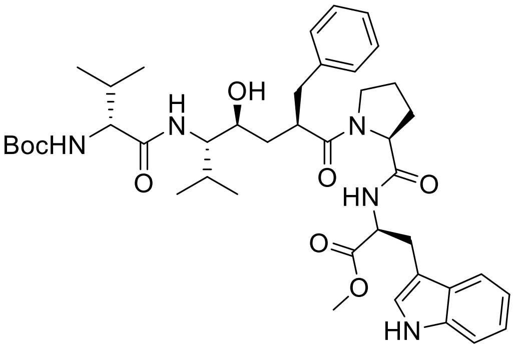

***epi*-9**

According to the procedure for preparation of **9**, in a 1.5 mL glass vial, equipped with a small Teflon®-coated magnetic stirring bar, an aliquot (88 µL, 1.2 eq) of a stock solution of Boc-DL-Val-OH (5.0 mg, 0.023 mmol) in absolute DMF (1.00 mL) was treated with an aliquot (7 µL, 1.2 eq) of a stock solution of HATU (78 mg, 0.21 mmol) in absolute DMF (0.70 mL) using microliter-syringes. The mixture was stirred for 1 min and then immediately treated with an aliquot (10 µL) of the solution of the crude deprotected intermediate in absolute DMF (97 mg in 1.00 mL, from the preparation of **9**). After 15 min, the reaction was quenched by the addition of water (200 µL) and extracted with EtOAc (500 µL). The organic layer was separated, and evaporated under reduced pressure. The residue was dissolved in 1000 µL of MeCN and analyzed by TLC and HPLC-ESI-MS.

R_f = 0.51 (CH_2Cl_2/MeOH 10:1 (v/v); staining: $KMnO_4$).

HPLC-ESI-MS (Agilent Poroshell120; method: SHE_Poroshell120_HCOOHMeCN_40_60_95): t_R(**9**) = 7.61 min, 48.2%, $[M + Na]^+$ = 785, $[M + K]^+$ = 801; t_R(***epi*-9**) = 7.79 min, 47.1%$[M + Na]^+$ = 785, $[M + K]^+$ = 801.

2.3.11 (2*S*)-2-{[(2*S*)-1-[(2*R*,4*S*,5*S*)-5-[(2*S*)-2-Azaniumyl-3-methylbutanamido]-2-benzyl-4-hydroxy-6-methylheptanoyl]pyrrolidin-2-yl]formamido}-3-(1*H*-indol-3-yl)propanoate (10)

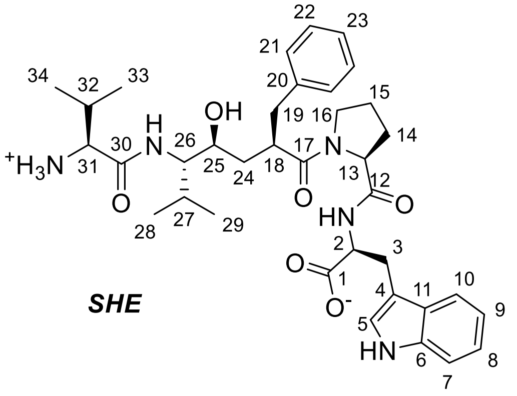

10

Saponification: In a 5 mL glass vial equipped with a Teflon®-coated magnetic stirring bar, **9** (46 mg, 0.060 mmol, 1.0 eq) was dissolved in THF (0.24 mL). A solution of LiOH×H_2O (21 mg, 0.48 mmol, 8.0 eq) in H_2O (0.40 mL) was added and the mixture was stirred vigorously until TLC indicated full conversion (15 min). The solution was adjusted to pH = 4 with AcOH. The stirring was stopped and the white colloidal mixture was extracted with Et_2O (3×2 mL). The combined organic extracts were dried over Na_2SO_4, concentrated under reduced pressure and dried in high vacuum to constant mass to yield a saponified intermediate as an off-white powder (40 mg, m.p. = 121–124 °C).

Boc-deprotection: In a 5 mL glass vial equipped with a Teflon®-coated magnetic stirring bar, the saponified intermediate (40 mg) was dissolved in 2,2,2-trifluoroethanol (1.20 mL). Under vigorous stirring ethanethiol (18 µL, 0.24 mmol, 4.0 eq) and $ZnBr_2$ (108 mg, 0.480 mmol, 8.0 eq) were added. The reaction mixture was stirred vigorously for 10 h and a white precipitate formed. The mixture was concentrated under reduced pressure and the residue was dissolved in water (1.20 mL) and MeOH (200 µL). Purification via preparative reverse phase HPLC (method: JKV_NucleodurC18_001HCOOH_10to85) afforded **10** (18 mg, 0.028 mmol, 46%, 2 steps) as a white solid.

Yield: 18 mg (0.028 mmol, 46%, 2 steps, from **9**), white solid.

m.p. = 151–155 °C

$[\alpha]_D^{23}$ = -24 ° (c = 0.61, MeOH).

^{1}H NMR (300 MHz, methanol-d4/D_2O 5:1, complex mixture of signals of cabamate rotamers and intramolecular interaction stabilized conformers in equilibrium, assigned based on COSY and HSQC) δ = 7.69–7.55 (m, 1H, H–C10), 7.41–6.89 (m, 9H, H–Ar), 4.65–4.37 (m, 1H, H–

C2), 3.83–2.58 (m, 11H, H–C13, H–C31, H–C25, H_2–C3, H_2–C16, H–C18, H_2–C19, H–C26), 2.36–2.18 (m, 1H, H–C32), 2.11–1.14 (m, 7H, H_2–C14, H_2–C15, H_2–C24 and H–C27), 1.14–0.76 (m, 12H, H_3–C28, H_3–C29, H_3–C33 and H_3–C34).

^{13}C NMR (75 MHz, methanol-d4/D_2O 5:1, complex mixture of signals of cabamate rotamers and intramolecular interaction conformers in equilibrium, assigned based on COSY and HSQC) δ = 177.2, 176.4, 173.6 and 169.2 (s, 4C, 3 amide C=O and an ester C=O), 140.0 (s, 1C, C20), 137.9(s, 1C, C6), 130.4–126.7 (m, 6C, C11, C21, C22 and C23), 124.4 (s, 1C, C5), 122.4 (s, 1C, C8), 119.8 (s, 1C, C9), 119.6 (s, 1C, C10), 112.3 (s, 1C, C7), 68.6 (s, 1C, C25), 62.3–61.9 (m, 2C, C31 and C13), 47.5 (s, 1C, C16), 44.2 (s, 1C, C18), 42,8 (s), 41.4 (s, 1C, C19), 40.4, 39.2 (s, 1C, C24), 37.4 (s, 1C, C2), 32.2 (s, 1C, C14), 31.6 (s, 1C, C32), 30.6 (s, 1C, C27), 28.6 (s, 1C, C3), 22.4 (s, 1C, C15), 20.8–17.5 (m, 4C, C33, C34, C28, C29).

HPLC-ESI-MS (Agilent Poroshell120; method: fast_Poroshell_001HCOOH_8mingradient.lc): t_R(**10**) = 5.54 min, 100%, $[M + 1]^+$ = 648, $[M + Na]^+$ = 670.

HRMS (ESI): m/z (%): 648.3756 (100%, $[M + 1]^+$, calcd for $C_{36}H_{50}N_5O_6^+$: 648.3756), 670.3577 (100%, $[M + Na]^+$, calcd for $C_{36}H_{49}N_5NaO_6^+$: 670.3575).

Synthesis of *HER* and derivatives

The synthetic route towards the hydroxyethylene core structure is following the methodology of B. E. Haug and D. H. Rich.[11]

2.3.12 Methyl (*tert*-butoxycarbonyl)-L-valinate (11)

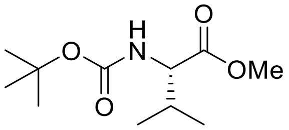

11

A 500 mL three-necked round bottom flask with magnetic stirring bar, fitted with a gas adapter and a septum with ballon was predried (evacuated, heated, N_2-purged) and charged with a solution of 21.7 g (100 mmol, 1.0 eq) Boc-Val-OH in 147 mL abs. DMF and 20.0 g (200 mmol, 2.0 eq) potassium hydrogen carbonate. 9.96 mL (160 mmol, 1.6 eq) methyl iodide were added dropwise to the stirred white suspension with a syringe (syringe pump, flowrate: 20 mL/h) over a period of 30 min. After the addition was finished the pale yellow reaction mixture was stirred for additional 3 h at RT and transferred into a 1 L separation funnel afterwards. The mixture was diluted with water (400 mL) and the product was extracted with a mixture of cyclohexane/EtOAc (1:1 v/v, 3×85 mL). The combined organic layers were washed with H_2O (2×85 mL), 5 % aqueous Na_2SO_4 (2×85 mL) and brine (85 mL). After drying over Na_2SO_4 the solvents were removed under reduced pressure and the residue was dried *in vacuo* to yield a colorless oil which was used without further purification.

Yield: 22.9 g (99.1 mmol, 99 %), colorless oil.

$C_{11}H_{21}NO_4$ [231.29 g/mol].

$[\alpha]_D^{24}$ = +13.2 (c = 1.91, $CHCl_3$); lit. $[\alpha]_D^{22}$ = +12.9 (c = 2.43, $CHCl_3$)[12].

R_f = 0.86 (cyclohexane/EtOAc = 1:4 (v/v); staining: $KMnO_4$).

^{1}H NMR (300 MHz, $CDCl_3$): δ = 5.01 (d, ^3J = 7,3 Hz, 1H, NH), 4.20 (dd, ^3J = 8.5, 4.6 Hz, 1H, HNCH), 3.71 (s, 3H, OCH_3), 2.18–1.98 (m, 1H, $(CH_3)_2CH$), 1.42 (s, 9 H, H-Boc), 0.93 (d, ^3J = 6.8 Hz, 3H, H_3CCHCH_3), 0.87 (d, ^3J = 6.9 Hz, 3H, H_3CCHCH_3).

^{13}C NMR (75 MHz, $CDCl_3$): δ = 173.0 (s, 1C, C=O), 155.8 (s, 1C, OCONH), 79.8 (s, 1C, Me_3C), 58.7 (s, 1C, NHCH), 52.1 (s, 1C, OCH_3), 31.4 (s, 1C, $(CH_3)_2CH$), 28.4 (s, 3C, $(CH_3)_3C$), 19.1 (s, 1C, H_3CCHCH_3), 17.7 (s, 1C, H_3CCHCH_3).

2.3.13 ***tert*-Butyl (*S*)-(1-(dimethoxyphosphoryl)-4-methyl-2-oxopentan-3-yl)carbamate (12)**

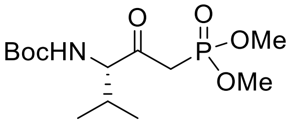

12

A 1 L three-necked round bottom flask with magnetic stirring bar, fitted with a gas valve and a septum was predried (evacuated, heated, N_2-purged) and charged with a solution of 17.0 mL (157 mmol, 3.3 eq) dimethyl methylphosphonate in 430 mL abs. THF. After cooling to –78 °C in a dry ice/acetone bath a 2.5 M *n*-butyllithium solution in hexane (60.8 mL, 152 mmol, 3.2 eq) was added with a syringe (syringe pump, flowrate: 2.0 mL/min) over a period of 30 min. The yellow solution was additionally stirred for 1 h at –78 °C at which point a –78 °C cold solution of the methyl ester **11** (11.0 g, 47.6 mmol, 1.0 eq) in 140 mL abs. THF was added. Stirring was continued for 3 h at –78 °C until TLC indicated full conversion and the reaction mixture was quenched by addition of 200 mL satd. NH_4Cl. The mixture was transferred into a separation funnel, the layers were separated and aqueous layer was extracted with EtOAc (2×200 mL). The combined organic phases were washed with H_2O (2×200 mL) and brine (100 mL), dried over Na_2SO_4, concentrated under reduced pressure and dried *in vacuo*. The resulting pale yellow oil was directly used for the next step without further purification.

Yield: 13.5 g (41.8 mmol, 87 %), pale yellow oil.

$C_{13}H_{26}NO_6P$ [323.33 g/mol].

$[\alpha]_D^{24}$ = +26.1 (c = 0.95, $CHCl_3$); lit. $[\alpha]_D^{22}$ = +16.8 (c = 1.35, $CHCl_3$)[13].

R_f = 0.31 (cyclohexane/EtOAc = 1:4 (v/v); staining: $KMnO_4$).

^{1}H NMR (300 MHz, $CDCl_3$, based on COSY): δ = 5.31 (d, ^3J = 8.8 Hz, 1H, NH), 4.35–4.21 (m, 1H, HNCH), 3.79–3.70 (m, 6H, 2 × OCH_3), 3.35–2.95 (m, 2H, CH_2), 2.35–2.07 (m, 1H, $(CH_3)_2CH$), 1.39 (s, 9H, H-Boc), 0.95 (d, ^3J = 6.7 Hz, 3H, $CH(CH_3)_2$), 0.76 (d, ^3J = 6.7 Hz, 3H, $CH(CH_3)_2$’).

^{13}C NMR (75 MHz, $CDCl_3$, based on HSQC): δ = 201.4 (d, 1C, C=O), 156.0 (s, 1C, OCONH), 79.9 (s, 1C, Me_3C), 65.1 (s, 1C, HNCH), 53.2 (dd, 2C, 2 × OCH_3), 39.6 and 37.8 (CH_2P), 29.2 (s, 1C, $(CH_3)_2CH$), 28.3 (s, 3C, $(CH_3)_3C$), 19.9 (s, 1C, $CH(CH_3)_2$), 16.8 (s, 1C, $CH(CH_3)_2$’).

^{31}P NMR (202 MHz, $CDCl_3$) δ = 22.19 (s).

2.3.14 Methyl glyoxylate (13)

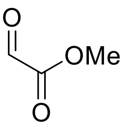

13

The synthesis was performed according to a procedure of P. F. Schuda *et al*.[14]

A dry 500 mL round bottom flask with magnetic stirring bar (evacuated, heated, N_2-purged) was charged with a suspension of 11.9 g (66.8 mmol, 1.0 eq) dimethyl tartrate in 134 mL Et_2O. 15.2 g (66.8 mmol, 1.0 eq) periodic acid were added to the stirred suspension in small portions over a period of 40 min. During the addition a fine, white precipitate was formed. After the addition was finished the reaction mixture was stirred for 20 min until the suspension turned to a clear solution with a white precipitate stuck on the glass wall of the flask. The suspension was filtered through a glass frit (por.4), the filter cake was washed with Et_2O (3×30 mL) and the filtrate was dried over Na_2SO_4 for 30 min. The solvent was removed under reduced pressure (T ≤ 35 °C) and the oily residue dried in high vacuum for 15 min. The crude aldehyde was stored under Ar in the fridge and directly used without further purification on the next day.

R_f = 0.47 (cyclohexane/EtOAc = 1:1 (v/v); product stains immediately with $KMnO_4$).

$C_3H_4O_3$ [88.06 g/mol].

2.3.15 Methyl (*S*)-5-((*tert*-butoxycarbonyl)amino)-6-methyl-4-oxoheptanoate (15)

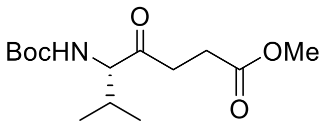

15

Horner-Wadsworth-Emmons reaction: A 1000 mL three-necked round bottom flask with magnetic stirring bar was dried (evacuated, heated, N_2-purged) and equipped with a gas valve and a thermometer. The flask was charged with a solution of 13.5 g (41.8 mmol, 1.0 eq) ketophosphonate **12** in 417 mL abs. THF and cooled to 0 °C (ice bath). 3.34 g (83.5 mmol, 2.0 eq) NaH (60 % dispersion in mineral oil) were added in small portions under a slight stream of N_2 over a period of 30 min and the reaction mixture was additionally stirred for 25 min at 0 °C. After cooling to –78 °C (dry ice/acetone bath) a –78 °C cold solution of the freshly prepared aldehyde **13** in 104 mL abs. THF was added with a cannula and the yellow mixture was stirred for 30 min at –78 °C. The flask was warmed up to –30 °C and the mixture stirred for 1.5 h at that temperature (cooled in an ice/$CaCl_2$ slurry and liquid N_2) until TLC indicated full conversion. The reaction mixture was quenched by slow addition of 5 mL glacial acetic acid in 10 mL THF over a period of 1 h at –30 °C, warmed to RT and the solvent was removed under

reduced pressure. The oily residue was partitioned between EtOAc (300 mL) and H_2O (100 mL). The layers were separated and the organic phase washed with H_2O (100 mL), satd. $NaHCO_3$ (100 mL) and brine (100 mL), dried over Na_2SO_4 and filtered. The solvent was removed under reduced pressure and the resulting yellow oil dried *in vacuo*.

Hydrogenation: The crude oil (13.6 g) was dissolved in 250 mL THF in a 500 mL Schlenk flask with magnetic stirring bar. The flask was evacuated and purged with argon and 890 mg palladium on charcoal (5 % palladium; 1 mol-%) were added. The flask was evacuated and purged with H_2 three times (balloon) and the suspension was stirred at RT for 16 h. Subsequently, the catalyst was carefully removed under an argon atmosphere by filtration through a Schlenk-frit containing a plug of Celite® and the filtrate was concentrated under reduced pressure. Purification via flash chromatography (140 g SiO_2; cyclohexane/EtOAc = 1:0→1:1 (v/v); staining: $KMnO_4$) yielded 9.67 g of a wax-like white solid.

Yield: 9.67 g (33.6 mmol, 80 %, 2 steps), wax-like white solid.

$C_{14}H_{25}NO_5$ [287.36 g/mol].

$[\alpha]_D^{23}$ = +34.0 (c = 1.50, $CHCl_3$).

R_f = 0.70 (cyclohexane/EtOAc = 1:1 (v/v); staining: $KMnO_4$).

^{1}H NMR (300 MHz, $CDCl_3$): δ = 5.09 (d, ^3J = 8.0 Hz, 1H, N<u>H</u>), 4.26 (dd, ^3J = 8.3 Hz, 3.9 Hz, 1H, NHC<u>H</u>), 3.66 (s, 3H, $CO_2C\underline{H}_3$), 3.00–2.45 (m, 4H, 2 × $C\underline{H}_2$), 2.30–2.10 (m, 1H, $(CH_3)_2C\underline{H}$), 1.42 (s, 9H, H-Boc), 1.00 (d, ^3J = 6.8 Hz, 3H, $\underline{H}_3CCH$), 0.79 (d, ^3J = 6.8 Hz, 3H, $\underline{H}_3C$’CH).

^{13}C NMR (75 MHz, $CDCl_3$): δ = 208.0 (s, 1C, C=O), 173.0 (s, 1C, C=O), 156.1 (s, 1C, OCONH), 79.8 (s, 1C, $Me_3\underline{C}$), 64.0 (s, 1C, HNCH), 51.9 (s, 1C, OCH_3), 35.5 (s, 1C, CH_2), 30.4 (s, 1C, $(CH_3)_2\underline{C}H$), 28.4 (s, 3C, $(\underline{C}H_3)_3C$), 27.6 (s, 1C, CH_2’), 20.0 (s, 1C, $H_3\underline{C}CH$), 16.8 (s, 1C, $H_3\underline{C}$’CH).

2.3.16 *tert*-Butyl ((*S*)-2-methyl-1-((*R*)-5-oxotetrahydrofuran-2-yl)propyl)-carbamate (16)

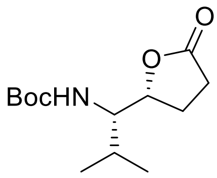

16

Stereoselective reduction: A 500 mL three-necked round bottom flask with magnetic stirring bar was dried (evacuated, heated, N_2-purged) and equipped with a gas valve, septum and thermometer. 9.00 g (31.3 mmol, 1.0 eq) ketoester **15** were dissolved in 313 mL abs. THF and the solution was cooled to –60 °C (dry ice/acetone bath). $LiAlH(O\textit{t}\text{-}Bu)_3$ (23.9 g, 94.0 mmol, 3.0 eq) was added in one portion and the white suspension, which turned yellow after 30 min, was stirred for 20 h at a temperature between –40 to –30 °C using a dry ice/acetone bath. The reaction was quenched by addition of 25 % aqueous tartaric acid (300 mL), extracted with EtOAc (2×300 mL) and the combined organic phases were washed with H_2O (2×100 mL), satd. $NaHCO_3$ (100 mL) and brine (100 mL), dried over Na_2SO_4 and filtered. The solvents were evaporated under reduced pressure to a small volume and the crude residue was directly used for the next step without further purification.

Acid-catalyzed lactonization: The crude residue was dissolved in 150 mL toluene in a 250 mL round bottom flask equipped with a reflux condenser and 6.0 mg (32 µmol, 0.1 mol-%) *p*-TsOH×H_2O were added. The colorless solution was heated to 60 °C (oil bath) and stirred for 12 h. Subsequently, the solvent was removed under reduced pressure and the oily residue was dried in high vacuum for 1 h. The product was precipitated by dissolving the oil in 150 mL *n*-hexane. The product was collected by filtration through a glass frit (por.4), washed with *n*-hexane (3×30 mL) and dried *in vacuo* to obtain 3.28 g (12.8 mmol, 41 %) of the pure diastereomer **16** as a white powder. The filtrate was concentrated *in vacuo* and purified via flash chromatography (430 g SiO_2; cyclohexane/EtOAc = 3:1 (v/v); staining: $KMnO_4$) to obtain additional 1.11 g (4.31 mmol, 14 %) **16**. Single crystals (colorless needles) were obtained by crystallization from *n*-hexane.

Yield: 4.39 g (17.1 mmol, 55 %), white powder.

$C_{13}H_{23}NO_4$ [257.33 g/mol].

mp = 106–108 °C.

$[\alpha]_D^{23}$ = –9.0 (c = 1.02, $CHCl_3$).

R_f = 0.27 (cyclohexane/EtOAc = 2:1 (v/v); staining: $KMnO_4$).

^{1}H NMR (300 MHz, $CDCl_3$, based on COSY and HSQC): δ = 4.41 (d, ^{3}J = 9.7 Hz, 1H, $N\underline{H}$), 4.37-4.27 (m, 1H, $CO_2C\underline{H}$), 3.72-3.53 (m, 1H, $HNC\underline{H}$), 2.65-2.40 (m, 2H, $CH_2C\underline{H}_2CO_2$), 2.35-2.20 (m, 1H, 1 × $C\underline{H}_2CH_2CO_2$), 2.20-1.98 (m, 2H, containing 1 × $C\underline{H}_2CH_2CO_2$ and $(CH_3)_2C\underline{H}$), 1.43 (s, 9H, H-Boc), 0.94 (d, ^{3}J = 6.9 Hz, 3H, $\underline{H}_3CCH$), 0.88 (d, ^{3}J = 6.9 Hz, 3H, $\underline{H}_3C'CH$).

^{13}C NMR (75 MHz, $CDCl_3$, based on HSQC): δ = 177.0 (s, 1C, C=O), 156.2 (s, 1C, OCONH), 80.0 (s, 1C, $Me_3\underline{C}$), 79.8 (s, 1C, $CO_2\underline{C}H$), 57.8 (HNCH), 28.4 (s, 3C, $(\underline{C}H_3)_3C$), 28.2 (s, 2C, containing $CH_2\underline{C}H_2CO_2$ and $(CH_3)_2\underline{C}H$), 25.1 (s, 1C, $\underline{C}H_2CH_2CO_2$), 19.9 (s, 1C, $H_3\underline{C}CH$), 15.7 (s, 1C, $H_3\underline{C}'CH$).

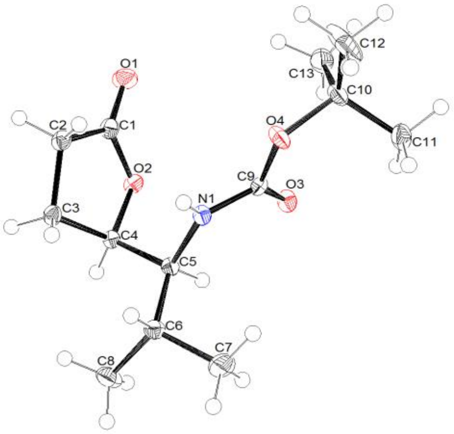

Figure SI2: X-ray crystal structure (ORTEP) of the major (*R*,*S*)-configurated γ-lactone **16**. Thermal ellipsoids are drawn at 50 % probability level. Determined configuration of C-4 is based on the known configuration of C-5. The structure has been deposited at CCDC/FIZ Karlsruhe deposition service under the CCDC Deposition Number 2098559. Data Block Name: data_RF828_0m_a. Unit Cell Parameters: a 5.2256(3) b 11.3275(7) c 24.3494(16) P212121.

2.3.17 *tert*-Butyl ((1*S*)-1-((2*R*)-4-(hydroxy(phenyl)methyl)-5-oxotetrahydrofuran-2-yl)-2-methylpropyl)carbamate (16a)

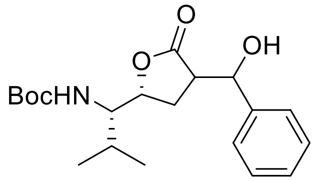

16a

Aldol reaction: A 100 mL three-necked round bottom flask with magnetic stirring bar was dried (evacuated, heated, N_2-purged) and equipped with a gas valve, thermometer and a glass stopper. 1.29 g (5.00 mmol, 1.0 eq) starting lactone **16** were dissolved in 25 mL abs. THF and the colorless solution was cooled to –78 °C (dry ice/acetone bath). Under vigorous stirring 5.00 mL (10.0 mmol, 2.0 eq) LDA solution (2.0 M in THF) were added and stirring was continued for 35 min at –78 °C. Freshly distilled benzaldehyde (1.02 mL, 10.0 mmol, 2.0 eq) was added with a syringe within 5 min and the reaction mixture was stirred for additional 30 min until TLC indicated full conversion. The mixture was quenched by addition of 12.5 mL satd. NH_4Cl solution and 5 mL H_2O and stirring was continued until the emulsion warmed up to RT. The mixture was poured into a separation funnel and the product was extracted with EtOAc (2×12 mL). The combined organic layers were washed with 1 M HCl (20 mL), satd. $NaHCO_3$ (20 mL) and brine (10 mL), dried over Na_2SO_4 and filtered. The solvents were removed under reduced pressure and the oily residue dired *in vacuo*. Purification via flash chromatography (110 g SiO_2; cyclohexane/EtOAc = 3:1→2:1 (v/v); staining: $KMnO_4$) yielded 1.29 g of a mixture of diastereomers.

Yield: 1.29 g (3.54 mmol, 71 %), white amorphous solid.

$C_{20}H_{29}NO_5$ [363.45 g/mol].

R_f = 0.30 and 0.38 (cyclohexane/EtOAc = 2:1 (v/v); staining: $KMnO_4$).

Two diastereomers were characterized separately:

^{1}H NMR (300 MHz, $CDCl_3$, unidentified diastereomer A, based on COSY): δ = 7.42–7.23 (m, 5H, Ar-H), 4.80 (dd, ^3J = 8.6 Hz, 7.0 Hz, 1H, PhCH), 4.35–4.10 (m, 2H, containing NH and *i*-PrCHCH), 3.74–3.46 (m, 1H, *i*-PrCH), 3.23–2.80 (m, 1H, HOCHCH), 2.14–1.76 (m, 3H, containing $CH(CH_3)_2$ and CH_2), 1.45–1.29 (m, 9H, H-Boc), 0.97–0.78 (m, 6H, $CH(CH_3)_2$).

^{1}H NMR (300 MHz, $CDCl_3$, unidentified diastereomer B, based on COSY): δ = 7.40–7.21 (m, 5H, Ar-H), 5.35 (s, 1H, PhCH), 4.70–4.20 (m, 2H, containing NH and *i*-PrCHCH), 3.63–3.36 (m, 1H, *i*-PrCH), 3.14–2.88 (m, 1H, HOCHCH), 2.50–2.15 (m, 1H, 1 × CH_2), 2.12–1.90 (m, 1H, $CH(CH_3)_2$), 1.89–1.74 (m, 1H, 1 × CH_2), 1.50–1.20 (m, 9H, H-Boc), 0.98–0.76 (m, 6H, $CH(CH_3)_2$).

HRMS (MALDI-TOF): Calcd. for $C_{20}H_{29}NO_5Na$ $[M+Na]^+$: 386.1943; found: 386.1945.

2.3.18 *tert*-Butyl ((*S*)-1-((*R*)-4-benzylidene-5-oxotetrahydrofuran-2-yl)-2-methylpropyl)carbamate (16b)

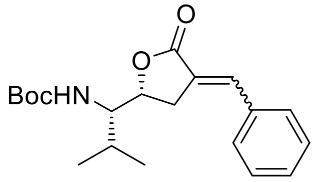

16b

Mesylation: A 50 mL Schlenk flask was dried (evacuated, heated, N_2-purged) and loaded with a solution of 1.20 g (3.29 mmol, 1.0 eq) of compound **16a** in 22 mL abs. DCM. 1.38 mL (9.88 mmol, 3.0 eq) triethylamine were added under stirring and the yellow solution was cooled to 0 °C (ice bath). 1.15 g (6.59 mmol, 2.0 eq) methanesulfonic anhydride were added in small portions within 45 min. Stirring was continued for 15 min at 0 °C, then 3.5 h at RT. Since no conversion could be observed, additional 0.69 mL (4.9 mmol, 1.5 eq) triethylamine and 0.57 g (3.3 mmol, 1.0 eq) methanesulfonic anhydride were added at RT. The reaction mixture was warmed to 30 °C (oil bath) and stirred at that temperature overnight. The reaction was quenched by addition of cold H_2O (11 mL) and the mixture was extracted with EtOAc (2×44 mL). The combined organic phases were washed with brine (11 mL), dried over Na_2SO_4 and filtered. Evaporation of the solvent and drying in vacuum yielded 1.75 g of the crude brown solid which was directly used without further purification.

Elimination: The crude was dissolved in 16.5 mL abs. EtOH in a 50 mL round bottom flask and 0.55 mL (3.95 mmol, 1.2 eq) triethylamine were added. The mixture was heated to 50 °C and stirred for 60 h. Subsequently, the solvent was evaporated under reduced pressure to a small volume and the residue partitioned between EtOAc (33 mL) and H_2O (15 mL). The phases were separated and the aqueous phase was extracted with EtOAc (16.5 mL). The combined organic layers were washed with brine (9 mL), concentrated *in vacuo* and directly used in the next step without further treatment.

R_f = 0.45 (cyclohexane/EtOAc = 2:1 (v/v); stains brown immediately with $KMnO_4$).

$C_{20}H_{27}NO_4$ [345.44 g/mol]

2.3.19 *tert*-Butyl ((*S*)-1-((2*R*,4*R*)-4-benzyl-5-oxotetrahydrofuran-2-yl)-2-methylpropyl)carbamate (17)

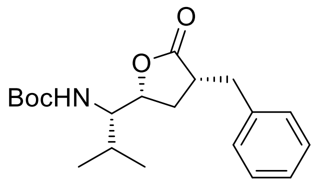

17

Crude compound **16b** (3.29 mmol) was dissolved in 33 mL THF in a 100 mL Schlenk flask with magnetic stirring bar. The flask was evacuated and purged with argon and 57 mg (0.99 mmol, 0.3 eq) Raney®-Nickel (slurry in H_2O) were added. Argon was exchanged by hydrogen (3×evacuation/purging) and the reaction mixture was stirred vigorously under H_2-atmosphere (balloon) for 21 h. Subsequently, the catalyst was carefully removed under an argon atmosphere by filtration through a Schlenk-frit containing a plug of Celite® and the filter cake was washed with EtOAc (3×10 mL). The filtrate was dried over Na_2SO_4 and the solvent evaporated. Purification via flash chromatography (120 g SiO_2; cyclohexane/EtOAc = 6:1 (v/v); staining: $KMnO_4$) yielded compound **17** as a white solid.

Yield: 623 mg (1.79 mmol, 54 % over 3 steps), white solid.

$C_{20}H_{29}NO_4$ [347.46 g/mol]

mp = 57–58 °C.

$[\alpha]_D^{23}$ = –60.8 (c = 1.24, $CHCl_3$).

R_f = 0.49 (cyclohexane/EtOAc = 2:1 (v/v); staining: $KMnO_4$).

^{1}H NMR (300 MHz, $CDCl_3$, based on COSY and NOESY): δ = 7.33–7.14 (m, 5H, Ar-H), 4.31 (d, ^3J = 10.0 Hz, NH), 4.24–4.10 (m, 1H, CO_2CH), 3.73–3.54 (m, 1H, HNCH), 3.29 (dd, ^2J = 13.6 Hz, ^3J = 2.9 Hz, 1H, 1 × $PhCH_2$), 2.94–2.76 (m, 1H, BnCH), 2.75–2.60 (m, 1H, 1 × $PhCH_2$), 2.31–2.16 (m, 1H, 1 × $CHCH_2CH$) 2.14–1.96 (m, 1H, $(CH_3)_2CH$), 1.93–1.76 (m, 1H, 1 × $CHCH_2CH$), 1.42 (s, 9H, H-Boc), 0.93 (d, ^3J = 6.8 Hz, 3H, H_3CCH), 0.84 (d, ^3J = 6.9 Hz, 3H, $H_3C'CH$).

^{13}C NMR (75 MHz, $CDCl_3$, based on HSQC): δ = 177.8 (s, 1C, C=O), 156.1 (s, 1C, OCONH), 138.8 (s, 1C, C_q-Ar), 129.0 (s, 2C, C-Ar), 128.8 (s, 2C, C-Ar), 126.8 (s, 1C, C-Ar), 79.9 (s, 1C, Me_3C), 78.2 (s, 1C, HCOCO), 58.3 (s, 1C, $(CH_3)_2CH$), 42.7 (s, 1C, BnCH), 36.5 (s, 1C, $PhCH_2$), 32.2 (s, 1C, $CHCH_2CH$), 28.4 (s, 4C, containing $(CH_3)_2CH$ and 3 × $(CH_3)_3C$), 19.9 (s, 1C, H_3CCH), 16.0 (s, 1C, $H_3C'CH$).

2.3.20 (2*R*,4*R*,5*S*)-2-Benzyl-5-((*tert*-butoxycarbonyl)amino)-4-((*tert*-butyldimethylsilyl)oxy)-6-methylheptanoic acid (RGSA) (18)

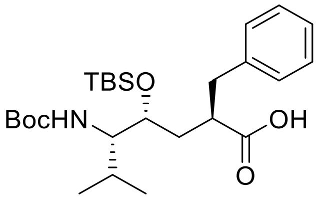

18

Lactone opening: In a 50 mL round bottom flask with magnetic stirring bar 577 mg (1.66 mmol, 1.0 eq) lactone **17** were dissolved in 5.5 mL THF and a solution of 279 mg (6.64 mmol, 4.0 eq) LiOH×H_2O in 8.3 mL water was added under vigorous stirring at RT. After 30 min 16.6 mL Et_2O were added to the stirred solution and the mixture was cooled down to 0 °C (ice bath). After carefully adjusting to pH=4 with citric acid (25 % in H_2O), the phases were separated and the aqueous phase was extracted with Et_2O (2×11 mL). The combined organic layers were washed with H_2O (11 mL) and brine (11 mL), dried over Na_2SO_4, filtered and the solvent was removed under reduced pressure with T ≤ 30 °C. The residue was transferred into a 10 mL Schlenk flask and dried under high vacuum.

Silylation: The crude product was dissolved in 6.6 mL abs. dichloromethane and the turbid solution was cooled to 0 °C (ice bath). 790 µL (9.96 mmol, 6.0 eq) *N*-methylimidazole were added and the colorless solution was stirred for 10 min at 0 °C. After the addition of 2.53 g (9.96 mmol, 6.0 eq) iodine in one portion the reaction mixture was stirred for additional 15 min and 751 mg (4.98 mmol, 3.0 eq) TBSCl were added in small portions. When the addition was finished, the ice bath was removed and the resulting mixture was stirred at RT for 18 h. After this period 12 mL Et_2O were added and the mixture was treated with 24 mL aqueous $Na_2S_2O_3$ (1 M) until decoloration occurred. The mixture was poured into a separation funnel and the phases were separated. The aqueous layer was extracted with Et_2O (2×12 mL) and the combined organic layers were washed with 25 % aqueous citric acid (12 mL) and brine (12 mL), dried over Na_2SO_4 and filtered. The solvents were removed under reduced pressure and the yellowish oil was dried *in vacuo*.

Ester hydrolysis: In a 10 mL glass vial with magnetic stirring bar the crude product was dissolved in 4.2 mL MeOH and 128 µL (0.17 mmol, 0.1 eq) citric acid (25 % in H_2O) were added. The solution was stirred at RT overnight. When the conversion was completed the solvents were removed under reduced pressure and the residue partitioned between EtOAc (12 mL) and H_2O (3 mL). The layers were separated and the solvents removed under reduced pressure. Purification via flash chromatography (85 g SiO_2; CH_2Cl_2/MeOH = 100:1 (v/v) + 0.5 vol-% AcOH) yielded compound **22** as a colorless oil.

Yield: 679 mg (1.42 mmol, 85 %), colorless oil.

$C_{26}H_{45}NO_5Si$ [479.73 g/mol]

$[\alpha]_D^{23}$ = –18.0 (c = 0.31, $CHCl_3$).

R_f = 0.15 (CH_2Cl_2/MeOH = 100:1 (v/v) + 0.5 vol-% AcOH; product stains white immediately with $KMnO_4$).

^{1}H NMR (300 MHz, $CDCl_3$): δ = 7.34–7.15 (m, 5H, Ar-H), 7.85 and 4.59 (d, ^3J = 10.2 Hz, 1H, NH), 3.85–3.69 (m, 1H, $SiOC\underline{H}$), 3.52–3.24 (m, 1H, $HNC\underline{H}$), 3.08–2.94 (m, 1H, 1 × $PhC\underline{H}_2$), 2.84–2.66 (m, 2H, containing 1 × $PhC\underline{H}_2$ and $PhCH_2C\underline{H}$), 1.95–1.78 (m, 1H, 1 × $CHC\underline{H}_2CH$), 1.73–1.50 (m, 2H, containing 1 × $CHC\underline{H}_2CH$ and $(CH_3)_2C\underline{H}$), 1.42 (s, 9H, H-Boc), 0.97–0.70 (m, 15H, containing $(C\underline{H}_3)_3CSi$ and $(C\underline{H}_3)_2CH$), 0.12–0.03 (m, 6H, $(C\underline{H}_3)_2Si$).

^{13}C NMR (75 MHz, $CDCl_3$, mixture of 2 rotamers, citing major rotamer based on COSY, HSQC and EXSY) δ = 179.0 (s, 1C, C=O), 156.2 (s, 1C, HNCO), 138.8 (s, 1C, C_q-Ar), 129.1 (s, 2C, C-Ar), 128.6 (s, 2C, C-Ar), 126.7 (s, 1C, C-Ar), 79.4 (s, 1C, $Me_3\underline{C}$), 71.9 (s, 1C, SiOCH), 58.0 (s, 1C, HNCH), 43.5 (s, 1C, $PhCH_2\underline{C}H$), 38.6 (s, 1C, $Ph\underline{C}H_2$), 35.3 (s, 1C, $CH\underline{C}H_2CH$), 28.6 (s, 3C, $(\underline{C}H_3)_3CO$), 28.1 (s, 1C, $(CH_3)_2\underline{C}H$), 26.0 (s, 3C, $(\underline{C}H_3)_3CSi$), 20.9 (s, 1C, 1 × $(\underline{C}H_3)_2CH$), 18.1 (s, 1C, 1 × $(\underline{C}H_3)_2CH$), -4.3 (CH_3Si), -4.6 (CH_3’Si).

2.3.21 Methyl ((2*R*,4*R*,5*S*)-2-benzyl-5-((*tert*-butoxycarbonyl)amino)-4-((*tert*-butyldimethylsilyl)oxy)-6-methylheptanoyl)-L-prolyl-L-tryptophanate (Boc-RGSA-Pro-Trp-OMe) (19)

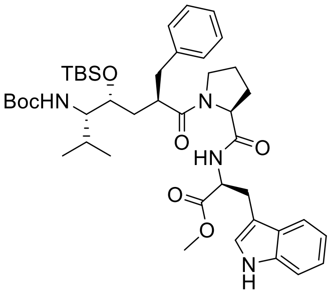

19

Boc-deprotection of Boc-Pro-Trp-OMe: A 10 mL round bottom flask with magnetic stirring bar was charged with 700 mg (1.68 mmol, 1.2 eq) Boc-Pro-Trp-OMe and 1.05 mL (14.0 mmol, 10 eq) ethanethiol. The heterogeneous mixture was dissolved in 3.24 mL (42.1 mmol, 30 eq) trifluoroacetic acid and stirred at RT for 60 min. Subsequently, the volatile componds were removed under reduced pressure and the residue was partitioned between EtOAc (13 mL) and 25 % aqueous NH_3 (3.2 mL). The layers were separated and the aqueous layer was extracted with EtOAc (3×13 mL). The combined organic phases were washed with brine (13 mL), dried over Na_2SO_4 and filtered. The solvent was removed under reduced pressure and the crude product was dried *in vacuo*. The deprotected H-Pro-Trp-OMe was used without further purification.

Peptide coupling: In a 50 mL round bottom flask with magnetic stirring bar 673 mg (1.40 mmol, 1.0 eq) acid **18** were dissolved in 6.3 mL abs. DMF and 245 µL (1.40 mmol, 1.0 eq) DIPEA were added to the stirred solution. After cooling to 0 °C (ice bath) 639 mg (1.68 mmol, 1.2 eq) HBTU were added in one portion. After 5 min of activation time a solution of the crude H-Pro-Trp-OMe (1.68 mmol, 1.2 eq) and 490 µL (2.81 mmol, 2.0 eq) DIPEA in 3.1 mL DMF was added and the ice bath was removed. The reaction mixture was stirred at RT for 90 min until TLC indicated full conversion. The mixture was quenched by the addition of 15 mL brine, transferred into a separation funnel and the product was extracted with EtOAc (45 mL). The organic phase was washed with brine (3×15 mL), dried over Na_2SO_4, filtered and the solvents were removed under reduced pressure. Purification via flash chromatography (110 g SiO_2; CH_2Cl_2/MeOH = 100:3 v/v), followed by H_2O washing (5×100 mL; product dissolved in 150 mL Et_2O) to remove remaining tetramethylurea, yielded compound **19** as an off-white solid.

Yield: 794 mg (1.02 mmol, 73 %), off-white solid.

$C_{43}H_{64}N_4O_7Si$ [777.09 g/mol].

mp = 62–67 °C.

$[\alpha]_D^{23}$ = –5.9 (c = 1.00, $CHCl_3$).

R_f = 0.80 (CH_2Cl_2/MeOH = 10:1 (v/v); staining: $KMnO_4$).

^{1}H NMR (300 MHz, $CDCl_3$): δ = 8.13 (bs, 1H, $N\underline{H}$ indole), 7.53 (d, ^3J = 7.1 Hz, 1H, H-Ar indole), 7.34 (d, ^3J = 7.1 Hz, 1H, $N\underline{H}$ amide), 7.29–6.97 (m, 9H, H-Ar), 4.81–4.71 (m, 1H, $C\underline{H}CO_2$), 4.62–4.46 (m, 2H, containing prolyl-$C\underline{H}$ and $N\underline{H}$ carbamate), 3.67 (s, 4H, containing $CO_2C\underline{H}_3$ and $SiOC\underline{H}$), 3.55–3.40 (m, 1H, 1 × prolyl-$C\underline{H}_2$), 3.55–3.40 (m, 3H, containing *i*-$PrC\underline{H}$ and $C\underline{H}_2$-tryptophan), 2.99–2.88 (m, 1H, 1 × prolyl-$C\underline{H}_2$), 2.80–2.68 (m, 1H, $BnC\underline{H}$), 2.67–2.45 (m, 2H, $PhC\underline{H}$), 2.28–2.15 (m, 1H, 1 × prolyl-$C\underline{H}_2$), 1.95–1.52 (m, 6H, containing $(CH_3)_2C\underline{H}$, $SiOCHC\underline{H}_2$ and 3 × prolyl-$C\underline{H}_2$), 1.40 (s, 9H, H-Boc), 0.94–0.75 (m, 15H, containing $(C\underline{H}_3)_3CSi$ and $(C\underline{H}_3)_2CH$), 0.15–0.03 (m, 6H, $(CH_3)_2Si$).

^{13}C APT NMR (75 MHz, $CDCl_3$, only *trans*-Pro rotamer observable, based on HSQC) δ = 175.0 (s, 1C, C=O), 172.5 (s, 1C, C=O), 171.2 (s, 1C, C=O), 156.0 (s, 1C, carbamate C=O), 139.2 (s, 1C, C_q from Ph), 136.2 (s, 1C, HNC_q from indole), 129.0 (s, 2C, C-Ar from Ph), 128.5 (s, 2C, C-Ar from Ph), 127.8 (s, 1C, C_q from indole), 126.6 (s, 1C, C-Ar from Ph), 123.5 (s, 1C, HNCH from indole), 122.2 (s, 1C, C-Ar indole), 119.6 (s, 1C, C-Ar indole), 118.7 (s, 1C, C-Ar indole), 111.3 (s, 1C, C-Ar indole), 110.3 (s, 1C, $\underline{C}_qCH_2$ indole), 79.0 (s, 1C, $Me_3\underline{C}$), 72.2 (s, 1C, SiOCH), 60.1 (s, 1C, prolyl-$\underline{C}H$), 57.7 (s, 1C, *i*-$Pr\underline{C}HCOSi$), 53.5 (s, 1C, $\underline{C}HCO_2$), 52.4 (s, 1C, $CO_2\underline{C}H_3$), 47.3 (s, 1C, prolyl-$\underline{C}H_2N$), 42.0 (s, 1C, $Bn\underline{C}H$), 39.0 (s, 1C, $Ph\underline{C}H_2$), 35.5 (s, 1C, $BnCH\underline{C}H_2$), 28.5 (s, 3C, $(\underline{C}H_3)_3CO$), 27.8 (s, 1C, $(CH_3)_2\underline{C}HCHCOSi$), 27.6 (s, 1C, $\underline{C}H_2$ from Trp), 27.4 (s, 1C, $CH\underline{C}H_2$ from Pro), 26.0 (s, 3C, $(\underline{C}H_3)_3CSi$), 24.9 (s, 1C, $CH_2\underline{C}H_2CH_2$), 21.4 (s, 1C, 1 × $(\underline{C}H_3)_2CH$ from Val), 18.4 (s, 1C, 1 × $(\underline{C}H_3)_2CH$ from Val), 18.1 (s, 1C, $(CH_3)_3\underline{C}Si$), -4.1 (CH_3Si), -4.7 (CH_3'Si).

HRMS (ESI): m/z (%): 777.4620 (87%, $[M + H]^+$, calcd for $C_{43}H_{65}N_4O_7Si^+$: 777.4617), 799.4441 (100%, $[M + Na]^+$, calcd for $C_{43}H_{64}N_4NaO_7Si^+$: 799.4442), 815.4184 (100%, $[M + K]^+$, calcd for $C_{43}H_{64}KN_4O_7Si^+$: 815.4181).

2.3.22 Methyl ((2*R*,4*R*,5*S*)-2-benzyl-5-((*S*)-2-((*tert*-butoxycarbonyl)amino)-3-methylbutanamido)-4-((*tert*-butyldimethylsilyl)oxy)-6-methylheptanoyl)-L-prolyl-L-tryptophanate (fully protected *HER*) (20)

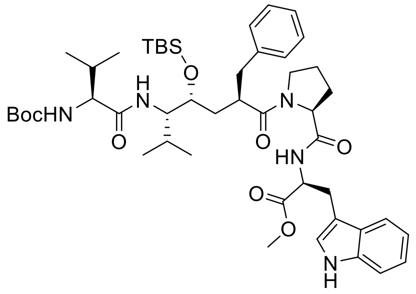

20

Boc-deprotection: 429 mg (0.552 mmol, 1.0 eq) compound **19** were dissolved in 2.55 mL (33.1 mmol, 60 eq) ice cold trifluoroacetic acid in a 50 mL round bottom flask with magnetic stirring bar. The ice bath was removed and the brown reaction mixture was stirred for 5 min at RT. The acid was removed under reduced pressure and the crude product dried *in vacuo* overnight.

Peptide coupling: A 10 mL Schlenk flask with magnetic stirring bar was dried under vacuum with a heat gun and purged with N_2. 144 mg (0.662 mmol, 1.2 eq) Boc-Val-OH were dissolved in 2.75 mL abs. DMF and 96.4 µL (0.552 mmol, 1.0 eq) DIPEA were added to the stirred solution. After cooling to 0 °C (ice bath) 252 mg (0.662 mmol, 1.2 eq) HATU were added in one portion and the yellow solution was stirred for 2 min. In a second 10 mL round bottom flask with Schlenk adaptor the previously deprotected crude compound was dissolved in 2.75 mL abs. DMF and 386 µL (2.21 mmol, 4.0 eq) DIPEA were added. After cooling to 0 °C (ice bath) the preactivated Boc-Val-OH solution was added with a syringe. After 5 min the ice bath was removed and the reaction mixture was stirred at RT for 45 min. The mixture was quenched by the addition of 5.5 mL brine and extracted with EtOAc (3×20 mL). The combined organic layers were washed with with a 1:1 mixture of H_2O/brine (6×20 mL) and brine (2×10 mL), dried over Na_2SO_4 and filtered. The product was concentrated under reduced pressure and dried *in vacuo*. Purification via flash chromatography (54 g SiO_2; cyclohexane/EtOAc = 1:1 v/v), followed by H_2O washing (5×10 mL; product dissolved in 60 mL EtOAc) to remove remaining tetramethylurea, provided compound **20** as a white solid.

Yield: 309 mg (353 µmol, 64 %), white solid.

$C_{48}H_{73}N_5O_8Si$ [876.22 g/mol].

mp = 82–88 °C.

$[\alpha]_D^{23}$ = –3.5 (c = 0.47, $CHCl_3$).

R_f = 0.29 (cyclohexane/EtOAc = 1:1 (v/v); staining: $KMnO_4$).

^{1}H NMR (300 MHz, $CDCl_3$, based on COSY and HSQC): δ = 8.73 (bs, 1H, N$\underline{H}$ indole), 7.49 (d, ^{3}J = 7.6 Hz, 1H, Ar-H), 7.39–6.90 (m, 10H, Ar-H and amide-N$\underline{H}$ from Trp), 6.21 (d, ^{3}J = 8.6 Hz, 1H, *i*-PrCHN$\underline{H}$), 5.00 (d, ^{3}J = 9.1 Hz, 1H, N$\underline{H}$ carbamate), 4.82–4.71 (m, 1H, C$\underline{H}CO_2$), 4.52 (d, ^{3}J = 8.6 Hz, 1H, prolyl-C$\underline{H}$), 3.92–3.80 (m, 1H, HNC$\underline{H}$ from Val), 3.79–3.69 (m, 2H, containing *i*-PrC$\underline{H}$, and SiOC$\underline{H}$), 3.66 (s, 3H, $CO_2C\underline{H}_3$), 3.57–3.40 (m, 1H, 1 × prolyl-$C\underline{H}_2N$), 3.27 (d, ^{3}J = 5.6 Hz, 2H, $C\underline{H}_2$-tryptophan), 3.14–2.98 (m, 1H, 1 × prolyl-$C\underline{H}_2N$), 2.82–2.61 (m, 2H, containing BnC$\underline{H}$ and 1 × $PhC\underline{H}_2$), 2.54–2.38 (m, 1H, 1 × $PhC\underline{H}_2$), 2.28–2.15 (m, 1H, 1 × $CHC\underline{H}_2$ from Pro), 2.15–2.02 (m, 1H, $(CH_3)_2C\underline{H}$ from Val), 1.95–1.71 (m, 4H, containing 1 × $SiOCHC\underline{H}_2$, 1 × $CHC\underline{H}_2$ from Pro and $CH_2C\underline{H}_2CH_2$), 1.70–1.51 (m, 2H, containing 1 × $SiOCHC\underline{H}_2$, and $(CH_3)_2CHCHCOSi$), 1.44 (s, 9H, H-Boc), 1.01–0.71 (m, 21H, containing $(C\underline{H}_3)_2CHCOSi$, $(C\underline{H}_3)_3CSi$ and $(C\underline{H}_3)_2CH$ from Val), 0.10 (s, 3H, $SiCH_3$), 0.06 (s, 3H, $SiCH_3$’).

^{13}C NMR (75 MHz, $CDCl_3$, based on HSQC): δ = 174.8 (s, 1C, C_2NC=O), 172.4 (s, 1C, C=O), 171.5 (s, 1C, C=O), 171.1 (s, 1C, C=O), 156.2 (s, 1C, C=O carbamate), 139.1 (s, 1C, C_q from Ph), 136.3 (s, 1C, HNC_q from indole), 129.0 (s, 2C, C-Ar from Ph), 128.6 (s, 2C, C-Ar from Ph), 127.7 (s, 1C, C_q from indole), 126.6 (s, 1C, C-Ar from Ph), 123.8 (s, 1C, HNCH from indole), 122.0 (s, 1C, C-Ar indole), 119.4 (s, 1C, C-Ar indole), 118.6 (s, 1C, C-Ar indole), 111.4 (s, 1C, C-Ar indole), 109.8 (s, 1C, $\underline{C}_qCH_2$ indole), 80.1 (s, 1C, $Me_3\underline{C}O$), 71.7 (s, 1C, SiOCH), 60.5 (s, 1C, HN$\underline{C}$H from Val), 60.0 (s, 1C, prolyl-$\underline{C}$H), 57.1 (s, 1C, *i*-Pr$\underline{C}$HCOSi), 53.3 (s, 1C, $\underline{C}HCO_2$), 52.4 (s, 1C, $CO_2\underline{C}H_3$), 47.2 (s, 1C, prolyl-$\underline{C}H_2N$), 41.8 (s, 1C, Bn$\underline{C}$H), 38.7 (s, 1C, $Ph\underline{C}H_2$), 34.6 (s, 1C, $BnCH\underline{C}H_2$), 30.6 (s, 1C, $(CH_3)_2\underline{C}H$ from Val), 28.4 (s, 4C, $(\underline{C}H_3)_3CO$ and $(CH_3)_2\underline{C}HCHCOSi$), 27.5 (s, 2C, $\underline{C}H_2$ from Trp and $CH\underline{C}H_2$ from Pro), 26.0 (s, 3C, $(\underline{C}H_3)_3CSi$), 24.9 (s, 1C, $CH_2\underline{C}H_2CH_2$), 21.2 (s, 1C, 1 × $(\underline{C}H_3)_2CHCHCOSi$), 19.7 (s, 1C, 1 × $(\underline{C}H_3)_2CH$ from Val), 18.9 (s, 1C, 1 × $(\underline{C}H_3)_2CHCHCOSi$), 18.1 (s, 1C, 1 × $(\underline{C}H_3)_2CH$ from Val), 17.8 (s, 1C, $Me_3\underline{C}Si$), –3.9 (s, 1C, $SiCH_3$), –4.6 (s, 1C, $SiCH_3$’).

HRMS (MALDI-TOF): Calcd. for $C_{48}H_{73}N_5O_8SiNa$ $[M+Na]^+$: 898.5126; found: 898.5116.

2.3.23 Methyl ((2*R*,4*R*,5*S*)-2-benzyl-5-((*S*)-2-((*tert*-butoxycarbonyl)amino)-3-methylbutanamido)-4-hydroxy-6-methylheptanoyl)-L-prolyl-L-tryptophanate (protected *HER*) (21)

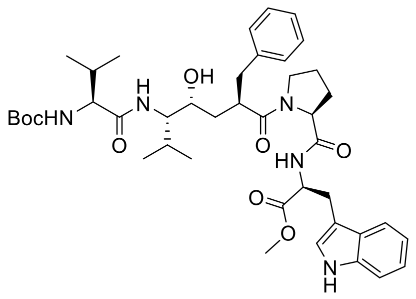

21

309 mg (353 µmol, 1.0 eq) compound **20** were dissolved in 3.5 mL THF in a 15 mL polypropylene vial with magnetic stirring bar. 917 µL (35.3 mmol, 100 eq) HF/pyridine (70 % HF) were added to the solution and the reaction mixture was stirred at RT for 25 min. Since no full conversion could be observed, additional 917 µL (35.3 mmol, 100 eq) HF/pyridine were added at RT and the mixture was stirred for additional 20 min. The reaction was quenched by pouring into 60 mL ice cold satd. $NaHCO_3$ solution and the product was extracted with EtOAc (2×60 mL). The combined organic layers were washed with H_2O (30 mL) and brine (30 mL), dried over Na_2SO_4 and filtered. The solvents were removed under reduced pressure and the product was dried *in vacuo*. Purification via flash chromatography (24 g SiO_2; CH_2Cl_2/MeOH = 20:1 v/v) provided compound **21** as an off-white solid.

Yield: 96 mg (0.13 mmol, 36 %), white solid.

$C_{42}H_{59}N_5O_8$ [761.96 g/mol].

mp = 93–96 °C.

$[\alpha]_D^{23}$ = –25.7 (c = 1.56, $CHCl_3$).

R_f = 0.20 (CH_2Cl_2/MeOH = 20:1 (v/v); staining: $KMnO_4$).

^{1}H NMR (300 MHz, $CDCl_3$, mixture of 2 rotamers in 4:1 ratio, major rotamer): δ = 9.03 (bs, 1H, N<u>H</u> indole), 7.48 (d, ^3J = 7.6 Hz, 1H, Ar-H), 7.38–6.86 (m, 10H, Ar-H and amide-N<u>H</u> from Trp), 6.07 (d, ^3J = 9.7 Hz, 1H, HOCHCHN<u>H</u>), 5.02 (d, ^3J = 8.7 Hz, 1H, N<u>H</u> carbamate), 4.69–4.55 (m, 1H, C<u>H</u>CO_2), 3.93–3.80 (m, 1H, *i*-PrC<u>H</u> from Val), 3.79–3.53 (m, 5H, containing *i*-PrC<u>H</u>, C<u>H</u>OH and $CO_2C\underline{H}_3$), 3.43–3.13 (m, 3H, containing $C\underline{H}_2$-tryptophan and prolyl-C<u>H</u>), 3.10–2.95 (m, 1H, 1 × prolyl-$C\underline{H}_2N$), 2.87–2.72 (m, 1H, 1 × prolyl-$C\underline{H}_2N$), 2.70–2.52 (m, 2H, $PhC\underline{H}_2$), 2.51–2.34 (m, 1H, BnC<u>H</u>), 2.30–1.59 (m, 5H, containing $(CH_3)_2C\underline{H}$), $(CH_3)_2C\underline{H}$’), $C\underline{H}_2CHOH$) and 1 × $CHC\underline{H}_2$ from Pro), 1.47–1.36 (m, 9H, H-Boc), 1.35–1.25 (m, 1H, 1 × $CH_2C\underline{H}_2CH_2$), 1.08–0.70 (m, 14H, containing 1 × $CHC\underline{H}_2$ from Pro, 1 × $CH_2C\underline{H}_2CH_2$, $(C\underline{H}_3)_2CH$ and $(C\underline{H}_3)_2CH$’).

^{13}C NMR (75 MHz, $CDCl_3$, mixture of 2 rotamers in 4:1 ratio, major rotamer): δ = 175.3 (s, 1C, $C_2NC=O$), 172.8 (s, 1C, C=O), 172.7 (s, 1C, C=O), 172.4 (s, 1C, C=O), 156.2 (s, 1C, C=O carbamate), 138.8 (s, 1C, C_q from Ph), 136.3 (s, 1C, HNC_q from indole), 129.2 (s, 2C, C-Ar from Ph), 128.6 (s, 2C, C-Ar from Ph), 127.6 (s, 1C, C_q from indole), 126.8 (s, 1C, C-Ar from Ph), 123.3 (s, 1C, HNCH from indole), 122.3 (s, 1C, C-Ar indole), 119.9 (s, 1C, C-Ar indole), 118.0 (s, 1C, C-Ar indole), 111.8 (s, 1C, C-Ar indole), 109.9 (s, 1C, $\underline{C}_qCH_2$ indole), 80.4 (s, 1C, $Me_3\underline{C}O$), 71.0 (s, 1C, SiOCH), 61.2 (s, 1C, HN$\underline{C}$H from Val), 60.7 (s, 1C, prolyl-$\underline{C}$H), 59.2 (s, 1C, *i*-Pr$\underline{C}$HCOH), 53.7 (s, 1C, $\underline{C}HCO_2$), 52.6 (s, 1C, $CO_2\underline{C}H_3$), 46.2 (s, 1C, prolyl-$\underline{C}H_2N$), 43.9 (s, 1C, Bn$\underline{C}$H), 39.9 (s, 1C, $Ph\underline{C}H_2$), 37.1 (s, 1C, $BnCH\underline{C}H_2$), 31.0 (s, 1C, $CH\underline{C}H_2$ from Pro), 30.0 (s, 1C, $(CH_3)_2\underline{C}H$ from Val), 28.5 (s, 3C, $(\underline{C}H_3)_3CO$), 28.1 (s, 1C, $(CH_3)_2\underline{C}HCHCOH$), 26.2 (s, 1C, $\underline{C}H_2$ from Trp), 21.8 (s, 1C, $CH_2\underline{C}H_2CH_2$), 20.6 (s, 1C, 1 × $(\underline{C}H_3)_2CHCHCOH$), 20.0 (s, 1C, 1 × $(\underline{C}H_3)_2CH$ from Val), 18.2 (s, 1C, 1 × $(\underline{C}H_3)_2CHCHCOH$), 16.8 (s, 1C, 1 × $(\underline{C}H_3)_2CH$ from Val).

2.3.24 ((2*R*,4*R*,5*S*)-5-((*S*)-2-Ammonio-3-methylbutanamido)-2-benzyl-4-hydroxy-6-methylheptanoyl)-L-prolyl-L-tryptophanate (*HER*) (23)

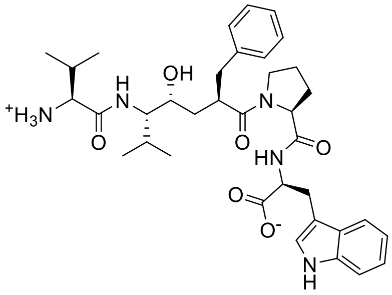

23

Saponification: 94.8 mg (124 µmol, 1.0 eq) compound **21** were dissolved in 0.4 mL THF in a 10 mL round bottom flask with magnetic stirring bar and a solution of 20.9 mg (498 µmol, 4.0 eq) LiOH×H_2O in 0.6 mL H_2O was added under vigorous stirring at RT. After 50 min the reaction mixture was carefully acidified with 1 M HCl to pH=5. The solvents were removed under reduced pressure and the yellow solid mass was dried *in vacuo* to obtain intermediate **22**.

Boc-deprotection: The reaction was carried out in two equal batches. The crude saponified intermediate **22** and 38 µL (0.50 mmol, 4.0 eq) ethanethiol were dissolved in 1.2 mL 2,2,2-trifluoroethanol in a 5 mL glass vial with magnetic stirring bar. 224 mg (996 µmol, 8.0 eq) zinc(II) bromide were added and the turbid yellowish solution was stirred at RT for 7 h. The solvent was evaporated and the product was purified by preparative HPLC (*JKV_NucleodurC18_001HCOOH_10to85*) to yield compound **23** as a white powder.

Yield: 35 mg (54 µmol, 44 %), white powder.

$C_{36}H_{49}N_5O_6$ [647.82 g/mol].

mp = 148–152 °C.

$[\alpha]_D^{23}$ = –20.8 (c = 1.54, MeOH).

^{1}H NMR (300 MHz, $CDCl_3$, based on COSY and HSQC, mixture of 2 rotamers in 3:1 ratio, major rotamer): δ = 8.41 (bs, 1H, N$\underline{H}$ indole), 7.54 (d, ^3J = 7.7 Hz, 1H, Ar-H), 7.32–6.86 (m, 9H, Ar-H), 4.60–4.47 (m, 1H, C$\underline{H}CO_2^-$), 3.83–3.59 (m, 3H, containing H_3N^+CH, *i*-PrC$\underline{H}$ and C$\underline{H}$OH), 3.45–3.30 (m, 2H, containing 1 × C$\underline{H}_2$-tryptophan and prolyl-C$\underline{H}$), 3.21–3.09 (m, 1H, 1 × C$\underline{H}_2$-tryptophan), 3.09–2.95 (m, 1H, 1 × prolyl-C$\underline{H}_2$N), 2.95–2.79 (m, 1H, 1 × prolyl-C$\underline{H}_2$N), 2.77–2.45 (m, 3H, containing BnC$\underline{H}$ and PhC$\underline{H}_2$), 2.30–2.17 (m, 1H, $(CH_3)_2$C$\underline{H}$), 2.17–2.04 (m, 1H, $(CH_3)_2$C$\underline{H}$’), 2.03–1.88 (m, 1H, 1 × C$\underline{H}_2$CHOH), 1.67–1.53 (m, 1H, 1 × C$\underline{H}_2$CHOH), 1.53–1.39 (m, 1H, 1 × CHC$\underline{H}_2$ from Pro), 1.20–0.74 (m, 14H, containing 1 × CHC$\underline{H}_2$ from Pro, 1 × CH_2C$\underline{H}_2$$CH_2$, (C$\underline{H}_3$)$_2$CH and (C$\underline{H}_3$)$_2$CH’), 0.73–0.54 (m, 1H, 1 × CH_2C$\underline{H}_2$$CH_2$).

^{13}C NMR (75 MHz, $CDCl_3$, based on HSQC, mixture of 2 rotamers in 3:1 ratio, major rotamer): δ = 177.7 (s, 2C, C=O), 173.3 (s, 1C, C=O), 170.4 (s, 1C, C=O), 140.2 (s, 1C, C_q from Ph), 138.0 (s, 1C, HNC_q from indole), 130.1 (s, 2C, C-Ar from Ph), 129.6 (s, 2C, C-Ar from Ph), 129.0 (s, 1C, C_q from indole), 127.7 (s, 1C, C-Ar from Ph), 124.3 (s, 1C, HNCH from indole), 122.4 (s, 1C, C-Ar indole), 119.8 (s, 1C, C-Ar indole), 119.4 (s, 1C, C-Ar indole), 112.2 (s, 1C, $\underline{C}_qCH_2$ indole), 112.0 (s, 1C, C-Ar indole), 71.1 (s, 1C, CHOH), 62.2 (s, 1C, prolyl-$\underline{C}H$), 61.2 (s, 1C, *i*-Pr$\underline{C}$HCOH), 60.2 (s, 1C, $H_3N^+\underline{C}H$), 56.6 (s, 1C, $\underline{C}HCO_2^-$), 47.3 (s, 1C, prolyl-$\underline{C}H_2N$), 45.4 (s, 1C, Bn$\underline{C}$H), 40.7 (s, 1C, Ph$\underline{C}H_2$), 37.7 (s, 1C, BnCH$\underline{C}H_2$), 31.9 (s, 1C, CH$\underline{C}H_2$ from Pro), 31.6 (s, 1C, $(CH_3)_2\underline{C}H$ from Val), 29.3 (s, 1C, $(CH_3)_2\underline{C}$HCHCOH), 28.3 (s, 1C, $\underline{C}H_2$ from Trp), 22.4 (s, 1C, $CH_2\underline{C}H_2CH_2$), 21.0 (s, 1C, 1 × $(\underline{C}H_3)_2$CHCHCOH), 19.6 (s, 1C, 1 × $(\underline{C}H_3)_2$CH from Val), 18.1 (s, 1C, 1 × $(\underline{C}H_3)_2$CHCHCOH), 17.5 (s, 1C, 1 × $(\underline{C}H_3)_2$CH from Val).

HPLC/MS (FAST_POROSHELL120_001HCOOH_8MINGRADIENT.M): t_R = 6.73 min; m/z (ESI+) = 648.4 $[M+H]^+$.

HRMS (MALDI-TOF): Calcd. for $C_{36}H_{49}N_5O_6Na$ $[M+Na]^+$: 670.3580; found: 670.3586.

2.3.25 *tert*-Butyl ((1*S*)-1-((2*R*)-4-((4-fluorophenyl)(hydroxy)methyl)-5-oxotetrahydrofuran-2-yl)-2-methylpropyl)carbamate (24a)

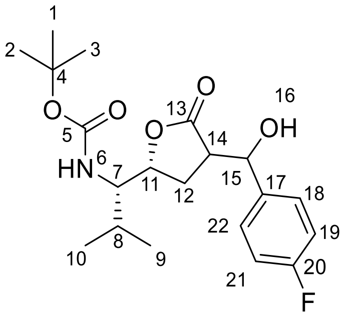

24a

A 100 mL three-necked round bottom flask, equipped with gas valve and magnetic stirring bar, was dried (evacuated, heated, N_2-purged) and charged with 700 mg (2.72 mmol, 1 eq.) lactone **16** and 15 mL abs. THF. The reaction mixture was cooled to -78 °C in a dry ice/acetone cooling bath followed by the dropwise addition of 4.08 mL (8.16 mmol, 3 eq.) LDA solution (2.0 M in THF). The deep orange reaction mixture was stirred at -78 °C for additional 60 min. Subsequently, 437 µL (4.08 mmol, 1.5 eq.) 4-fluorobenzaldehyde were added dropwise within 5 min. The reaction mixture turned yellow after a few min and was stirred for additional 50 min at -78 °C until TLC indicated full conversion. The reaction was quenched by the addition of 5 mL satd. NH_4Cl. The reaction mixture was allowed to warm to RT followed by the addition of 40 mL H_2O and 50 mL EtOAc. The phases were separated and the aqueous layer extracted with EtOAc (2x30 mL). The combined organic layers were washed with 1 M HCl (30 mL), brine (30 mL), dried over Na_2SO_4 and the solvent was removed under reduced pressure. The residue was purified via flash chromatography (300 g SiO_2, cyclohexane/EtOAc 7:1 to 1:1 (v/v)) to furnish two diastereomers, which were separated and characterized separately.

$C_{20}H_{28}FNO_5$ [381.44 g/mol].

Yield: unidentified diastereomer A: 225 mg, unidentified diastereomer B: 672 mg, total: 897 mg (2.35 mmol, 86%), colorless solid.

R_f = 0.44, 0.38 (cyclohexane/EtOAc = 1:1 (v/v); staining: $KMnO_4$).

m.p. = 74-76 °C.

^{1}H NMR (300 MHz, $CDCl_3$, unidentified diastereomer A): δ = 7.24 (d, ^3J = 7.6 Hz, 2H, *H*-22, 21), 7.16 (d, ^3J = 7.6 Hz, 2H, *H*-19, 18), 5.38- 5.31 (m, 1H, *H*-15), 4.46-4.19 (m, 2H, *H*-11, 6),

3.68-3.26 (m, 1H, *H*-7), 3.14-2.96 (m, 1H, *H*-14), 2.12-1.81 (m, 3H, *H*-12, 8), 1.47-1.33 (m, 9H, *H*-3, 2, 1), 0.88 (dd, ^3J = 19.3, 7.0 Hz, 6H, *H*-10, 9).

^{1}H NMR (300 MHz, $CDCl_3$, unidentified diastereomer B): δ = 7.25 (d, ^3J = 7.5 Hz, 2H, *H*-22, 21), 7.15 (d, ^3J = 7.6 Hz, 2H, *H*-19, 18), 4.78 (d, ^3J = 8.3 Hz, 1H, *H*-15), 4.29 (d, ^3J = 10.0 Hz, 1H, *H*-11), 4.22-4.09 (m, 1H, *H*-6), 3.63-3.37 (m, 1H, *H*-7), 3.25-3.04 (m, 1H, *H*-14), 2.08-1.84 (m, 3H, *H*-12, 8), 1.43-1.33 (m, 9H, *H*-3, 2, 1), 0.95-0.78 (m, 6H, *H*-10, 9).

^{19}F NMR (470 MHz, $CDCl_3$): δ = -113.8 (s).

^{19}F NMR (470 MHz, $CDCl_3$): δ = -116.7 (s).

2.3.26 *tert*-Butyl ((*S*)-1-((2*R*,4*R*)-4-(4-fluorobenzyl)-5-oxotetrahydrofuran-2-yl)-2-methylpropyl)carbamate (25a)

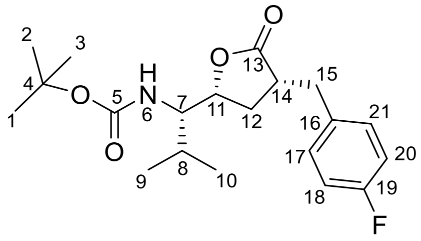

25a

Mesylation: A 100 mL round bottom flask, equipped with a Schlenk adapter and magnetic stirring bar, was dried (evacuated, heated, N_2-purged) and charged with 778 mg (2.04 mmol, 1 eq.) compound **24a**, 20 mL abs. dichloromethane and 1.41 mL (10.2 mmol, 5 eq.) triethylamine. The reaction mixture was cooled to 0 °C in an ice bath followed by the dropwise addition of 474 µL (6.12 mmol, 3 eq.) methanesulfonyl chloride. The reaction mixture was stirred at 0 °C for additional 2 h and at RT overnight. Since the conversion was not complete, additional 848 µL (6.12 mmol, 3 eq.) triethylamine and 315 µL (4.08 mmol, 2 eq.) methanesulfonyl chloride were added and the reaction mixture heated to 35 °C and stirred overnight. After 22 h TLC indicated full conversion. The reaction was quenched by the addition of 50 mL H_2O. The reaction mixture was transferred into a separation funnel and 20 mL dichloromethane were added. The phases were separated and the aqueous layer was extracted with dichloromethane (3x20 mL). The combined organic layers were washed with brine (50 mL), dried over Na_2SO_4 and the solvent was removed under reduced pressure to furnish a deep red solid residue which was used in the next step without further purification.

Elimination: A 100 mL round bottom flask, equipped with a Schlenk adapter and magnetic stirring bar, was dried (evacuated, heated, N_2-purged) and charged with the crude residue of the previous step and 25 mL abs. ethanol were added, followed by the addition of 565 µL (4.08 mmol, 2 eq.) triethylamine. The reaction mixture was heated to 40 °C and stirred at this temperature for 22 h. Since no full conversion could be observed the temperature was raised to 45 °C and stirred for additional 20 h. After this period the reaction mixture was allowed to come to RT and concentrated to a small volume. The residue was purified via flash chromatography (300 g SiO_2, cyclohexane/EtOAc 6:1 to 3:1 (v/v)) to give the corresponding olefin as a colorless solid.

Hydrogenation: In a two-necked round bottom flask, equipped with gas valve and magnetic stirring bar, the olefin was dissolved in 14 mL THF and 47 mg (0.82 mmol, 0.4 eq) Raney®-Nickel (slurry in H_2O) were added. Nitrogen atmosphere was exchanged by hydrogen (3×evacuation/purging) and the reaction mixture was stirred at RT for 2 d. After NMR indicated full conversion, the catalyst was removed under an argon atmosphere by filtration through a Schlenk-frit containing a plug of Celite® and the filter cake was washed with EtOAc (3×20 mL). The filtrate was concentrated and dried *in vacuo* to furnish the pure diastereomer as a colorless solid.

$C_{20}H_{28}FNO_4$ [365.44 g/mol].

Yield: 465 mg (1.27 mmol, 62%, 3 steps from **24a**), colorless solid.

R_f (olefin) = 0.28 (cyclohexane/EtOAc = 3:1 (v/v); staining: $KMnO_4$, stains with $KMnO_4$ without heating).

$[\alpha]_D^{24}$ = -49.6 (c = 0.62, $CHCl_3$).

m.p. = 65-67 °C.

^{1}H NMR (300 MHz, $CDCl_3$): δ = 7.22-7.11 (m, 2H, H-20, 18), 7.05-6.92 (m, 2H, H-21, 17), 4.39-4.11 (m, 2H, H-11, 6), 3.76-3.54 (m, 1H, H-7), 3.26 (d, ^3J = 12.9 Hz, 1H, H-15), 2.95-2.62 (m, 2H, H-15, 14), 2.33-2.17 (m, 1H, H-12), 2.16-2.01 (m, 1H, H-12), 1.86 (dd, ^3J = 16.2, 12.9 Hz, 1H, H-8), 1.43 (s, 9H, H-3, 2, 1), 0.95 (d, ^3J = 6.5 Hz, 3H, H-9), 0.86 (d, ^3J = 6.5 Hz, 3H, H-10).

^{13}C NMR (75 MHz, $CDCl_3$): δ = 177.6 (*C*-13), 160.3 (*C*-19), 156.1 (*C*-5), 134.4 (*C*-16), 130.5 (d, J_{C-F} = 7.9 Hz, *C*-21, 17), 115.6 (d, J_{C-F} = 20.9 Hz, *C*-20, 18), 80.0 (*C*-11), 78.2 (*C*-4), 58.4 (*C*-7), 42.6 (*C*-14), 35.6 (*C*-15), 32.0 (*C*-12), 28.5-28.2 (m, *C*-8, 3, 2, 1), 19.9 (*C*-9), 15.9 (*C*-10).

^{19}F NMR (470 MHz, $CDCl_3$): δ = -116.4 (s).

HRMS (MALDI-TOF): *m/z* calcd. for $C_{20}H_{28}FNO_4Na$ $[M+Na]^+$: 388.1900; found: 388.1882.

2.3.27 (2*R*,4*R*,5*S*)-5-((*tert*-Butoxycarbonyl)amino)-4-((*tert*-butyldimethylsilyl)oxy)-2-(4-fluorobenzyl)-6-methylheptanoic acid (27a)

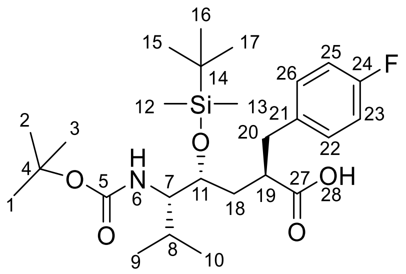

27a

Lactone opening: A 100 mL round bottom flask with magnetic stirring bar was charged with 465 mg (1.27 mmol, 1 eq.) alkylated lactone **25a** and 4.5 mL THF. A solution of 214 mg (5.09 mmol, 4 eq.) LiOHxH_2O in 6.7 mL H_2O was added and the turbid reaction mixture was stirred vigorously at RT. After 160 min TLC indicated full conversion and 12 mL Et_2O were added. The reaction mixture was cooled to 0 °C with an ice bath, followed by the careful addition of citric acid solution (25% in H_2O) until a pH of 4 was adjusted. Subsequently, the reaction mixture was transferred into a separation funnel and the phases were separated. The aqueous layer was extracted with Et_2O (3x20 mL). The combined organic layers were washed with brine (30 mL), dried over Na_2SO_4 and the solvent was evaporated under reduced pressure (T < 30 °C). The residue was dried *in vacuo* to furnish the intermediate product as a colorless solid. The crude product was used in the next step without further purification.

Silylation: A 100 mL three-necked round bottom flask, equipped with gas valve and magnetic stirring bar, was dried (evacuated, heated, N_2-purged) and charged with the crude product of the previous step and 5 mL abs. dichloromethane were added. After the addition of 609 µL (7.63 mmol, 6 eq.) *N*-methylimidazole the reaction mixture was cooled to 0 °C in an ice bath. After 10 min 1.93 g (7.63 mmol, 6 eq.) iodine were added and the deep red reaction mixture stirred for additional 15 min. 767 mg (5.09 mmol, 4 eq.) TBSCl were added portionwise within a period of 5 min. After the addition was complete the reaction mixture was stirred for additional 3 h at 0 °C and at RT overnight. Afterwards, 20 mL Et_2O were added and the mixture was transferred into a separation funnel. 30 mL 1 M $Na_2S_2O_3$-solution were added and the organic layer was washed until total decoloration occurred. The phases were separated and the aqueous layer was extracted with Et_2O (2x30 mL). The combined organic layers were washed with brine

(50 mL), dried over Na_2SO_4 and the solvents removed under reduced pressure. The yellow oily residue was used in the next step without further purification.

Ester hydrolysis: In a 50 mL round bottom flask, equipped with magnetic stirring bar the residue of the previous step was dissolved in 3.5 mL MeOH and 100 µL (0.13 mmol, 0.1 eq.) citric acid (25% in H_2O) were added. The yellow reaction mixture was stirred at RT overnight. After 23 h the reaction mixture was concentrated and purified via flash chromatography (375 g SiO_2, cyclohexane/EtOAc/AcOH 4:1:0.01 to 2:1:0.05 (v/v/v)) to give the protected acid **27a** as a viscous oil.

$C_{26}H_{44}FNO_5Si$ [497.72 g/mol].

Yield: 538 mg (1.08 mmol, 85%, 3 steps from **25a**), pale orange oil.

R_f = 0.37 (cyclohexane/EtOAc/AcOH = 2:1:0.05 (v/v/v); staining: $KMnO_4$, stains white with $KMnO_4$).

$[\alpha]_D^{24}$ = -8.3 (c = 0.51, $CHCl_3$).

^{1}H NMR (300 MHz, $CDCl_3$): δ = 7.20-7.08 (m, 2H, *H*-25, 23), 7.02-6.89 (m, 2H, *H*-26, 22), 4.60 (d, ^3J = 9.8 Hz, 1H, *H*-6), 3.86-3.64 (m, 1H, *H*-11), 3.52-3.25 (m, 1H, *H*-7), 3.04-2.86 (m, 1H, *H*-20), 2.84-2.62 (m, 2H, *H*-20, 19), 1.96-1.76 (m, 1H, *H*-18), 1.73-1.50 (m, 2H, *H*-18, 8), 1.41 (s, 9H, *H*-3, 2, 1), 0.89-0.74 (m, 15H, *H*-17, 16, 15, 10, 9), 0.10-0.02 (m, 6H, *H*-13, 12).

^{13}C NMR (75 MHz, $CDCl_3$): δ = 179.9 (*C*-27),160.2 (*C*-24), 156.2 (*C*-5), 134.5 (*C*-21), 130.6 (*C*-22), 130.5 (*C*-26), 115.6 (*C*-23), 115.3 (*C*-25), 79.5 (*C*-4), 71.9 (*C*-11), 57.9 (*C*-7), 43.7 (*C*-19), 37.8 (*C*-20), 35.7 (*C*-18), 28.6 (*C*-3, 2, 1), 28.0 (*C*-8), 25.8 (*C*-14), 26.0 (*C*-17, 16, 15), 20.9 (*C*-9), 18.1 (*C*-10),-4.3 (*C*-13), -4,.6 (*C*-12).

^{19}F NMR (470 MHz, $CDCl_3$): δ = -116.7 (s).

HRMS (MALDI-TOF): *m/z* calcd. for $C_{26}H_{44}FNO_5SiNa$ $[M+Na]^+$: 520.2870; found: 520.2848.

2.3.28 Methyl ((2*R*,4*R*,5*S*)-5-((*tert*-butoxycarbonyl)amino)-4-((*tert*-butyldimethylsilyl)oxy)-2-(4-fluorobenzyl)-6-methylheptanoyl)-L-prolyl-L-tryptophanate (28a)

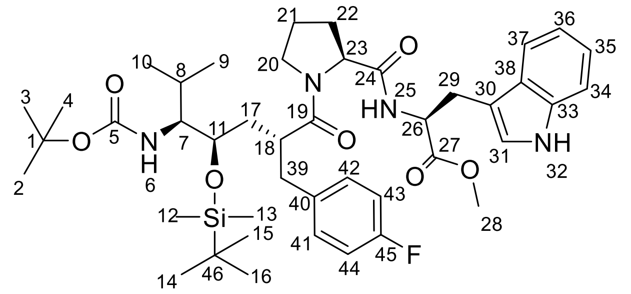

28a

Boc-Deprotection of Boc-Pro-Trp-OMe: In a 50 mL round bottom flask, equipped with a magnetic stirring bar, 516 mg (1.24 mmol, 1.2 eq.) Boc-Trp-OMe peptide were dissolved in 766 µL (10.4 mmol, 10 eq.) ethanethiol. Subsequently, 2.39 mL (31.0 mmol, 30 eq.) trifluoroacetic acid were added and the yellowish reaction mixture was stirred vigorously at RT. After 1 h the volatiles were removed under reduced pressure with a cooling trap. The residue was dissolved in 15 mL EtOAc, 25% aqueous ammonia (10 mL) were added and the reaction mixture was stirred for 5 min. The phases were separated and the aqueous layer was extracted with EtOAc (2x10 mL). The combined organic layers were dried over Na_2SO_4 and the solvent was removed under reduced pressure. The residue was dried *in vacuo* to furnish the deprotected intermediate as a colorless solid.

Coupling: A 50 mL round bottom flask, equipped with a Schlenk adapter and magnetic stirring bar, was dried (evacuated, heated, N_2-purged) and charged with 515 mg (1.03 mmol, 1 eq.) compound **27a** which was then dissolved in 5 mL abs. DMF, followed by the addition of 180 µL (1.03 mmol, 1 eq.) Hünig`s base. The flask was cooled to 0 °C in an ice bath and 471 mg (1.24 mmol, 1.2 eq.) HBTU were added to the reaction mixture. The previously deprotected peptide was dissolved in 2.5 mL abs. DMF and 360 µL (2.06 mmol, 2 eq.) Hünig`s base were added. After 5 min, the solution of the deprotected intermediate was added to the solution of acid **27a** with a syringe. After the addition was complete, the ice bath was removed and the yellow reaction mixture was stirred at RT for additional 2 h. Subsequently, the reaction mixture was quenched by the addition of brine (10 mL). Additionally, 20 mL of EtOAc were added and the mixture was stirred vigorously for 10 min. Afterwards, the mixture was transferred into a separation funnel and the phases were separated. The aqueous layer was extracted with EtOAc (2x20 mL). The combined organic layers were washed with brine (50 mL), dried over Na_2SO_4

and the solvents were removed under reduced pressure. The residue was purified via flash chromatography (375 g SiO_2, dichloromethane/MeOH 100:1 to 30:1 (v/v)) to furnish a pale orange oily residue. The residue was dissolved in 50 mL EtOAc and the organic layer washed with H_2O (5x50 mL) in order to remove remaining DMF and tetramethylurea. The organic layer was dried over Na_2SO_4, the solvent removed under reduced pressure and the residue dried *in vacuo* to yield the desired product as a colorless solid.

$C_{43}H_{63}FN_4O_7Si$ [795.08 g/mol].

Yield: 471 mg (0.59 mmol, 58%, 2 steps from **27a**), colorless solid.

R_f = 0.64 (dichloromethane/MeOH = 30:1 (v/v); staining: $KMnO_4$).

$[\alpha]^{24}_{D}$ = -0.6 (c = 0.99, $CHCl_3$).

m.p. = 79-81 °C.

^{1}H NMR (300 MHz, $CDCl_3$): δ = 8.14 (br s, 1H, *H*-32), 7.55 (d, ^3J = 7.0 Hz, 1H, *H*-37), 7.32-6.76 (m, 9H, *H*-44, 43, 42, 41, 36, 35, 34, 31, 25), 4.84-4.71 (m, 1H, *H*-26), 4.64-4.41 (m, 2H, *H*-23, 6), 3.71-3.60 (m, 4H, *H*-28, 11), 3.54-3.43 (m, 1H, *H*-20), 3.40-3.20 (m, 3H, *H*-29, 7), 2.99-2.88 (m, 1H, *H*-20), 2.73-2.42 (m, 3H, *H*-39, 18), 2.27-2.17 (m, 1H, *H*-22), 1.92-1.51 (m, 6H, *H*-22, 21, 17, 8), 1.40 (s, 9H, *H*-4, 3, 2), 0.91-0.78 (m, 15H, *H*-16, 15, 14, 10, 9), 0.09 (d, ^3J = 9.5 Hz, 6H, *H*-13, 12).

^{13}C NMR (75 MHz, $CDCl_3$): δ = 174.4 (*C*-19), 172.5 (*C*-27), 171.2 (*C*-24), 163.3 (*C*-45), 156.0 (*C*-5), 136.2 (*C*-40), 134.9 (*C*-33), 130.5 (*C*-42), 130.4 (*C*-41), 127.7 (*C*-38), 123.5 (*C*-31), 122.2 (*C*-35), 119.7 (*C*-36), 118.7 (*C*-37), 115.4 (*C*-44), 115.1 (*C*-43), 111.3 *C*-34), 110.2 (*C*-30), 79.0 (*C*-1), 72.2 (*C*-11), 60.2 (*C*-23), 57.7 (*C*-7), 53.5 (*C*-26), 52.4 (*C*-28), 47.3 (*C*-20), 42.0 (*C*-18), 38.1 (*C*-39), 35.6 (*C*-17), 28.6 (*C*-8), 28.5 (*C*-4, 3, 2), 27.8 (*C*-29), 27.6 (*C*-22), 26.0 (*C*-16, 15, 14), 24.9 (*C*-21), 21.4 (*C*-10), 18.4 (*C*-9), 18.0 (*C*-46), -4.1 (*C*-12), (*C*-13).

^{19}F NMR (470 MHz, $CDCl_3$): δ = -116.5 (s).

HRMS (MALDI-TOF): *m/z* calcd. for $C_{43}H_{63}FN_4O_7SiNa$ $[M+Na]^+$: 817.4348; found: 817.4398.

2.3.29 Methyl ((2*R*,4*R*,5*S*)-5-((*S*)-2-((*tert*-butoxycarbonyl)amino)-3-methylbutanamido)-2-(4-fluorobenzyl)-4-hydroxy-6-methylheptanoyl)-L-prolyl-L-tryptophanate (30a)

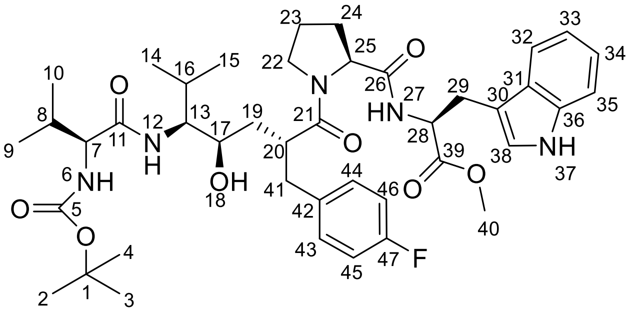

30a

Tandem deprotection of Boc- and TBS-moiety: In a 50 mL round bottom flask, equipped with magnetic stirring bar, 225 mg (0.283 mmol, 1 eq.) **28a** were dissolved in 3 mL 2,2,2-trifluoroethanol. Afterwards, 83 µL (1.13 mmol, 4 eq.) ethanethiol were added, followed by the addition of 509 mg (2.26 mmol, 8 eq.) zinc dibromide. The reaction mixture was stirred vigorously at RT and after 15 min the formation of a white precipitate could be observed. The turbid reaction mixture was stirred for additional 90 min, followed by the addition of 25% aqueous ammonia (6 mL) and EtOAc (10 mL). The reaction mixture was stirred for 10 min and subsequently transferred into a separation funnel and the phases were separated. The aqueous layer was extracted with EtOAc (2x10 mL). The combined organic layers were dried over Na_2SO_4 and the solvents were removed under reduced pressure. The residue was dried *in vacuo* for 30 min to furnish the deprotected intermediate as a colorless solid.

Coupling: A 50 mL round bottom flask, equipped with magnetic stirring bar and Schlenk adapter, was dried (evacuated, heated, N_2-purged) and charged with 74 mg (0.340 mmol, 1.2 eq.) Boc-L-Val-OH and 0.8 mL abs. DMF, followed by the addition of 49 µL (0.283 mmol, 1 eq.) Hünig's base. The flask was cooled to 0 °C with an ice bath. Subsequently, 129 mg (0.340 mmol, 1.2 eq.) HATU were added to the reaction mixture and stirred for additional 6 min. In a second 50 mL round bottom flask, equipped with magnetic stirring bar and Schlenk adapter, the previously deprotected peptide was dissolved in 1.6 mL abs. DMF and 99 µL (0.566 mmol, 2 eq.) Hünig's base were added. Afterwards, the solution of the deprotected peptide was added to the solution of the activated acid using a syringe. After the addition was complete, the ice bath was removed and the deep yellow reaction mixture was stirred for additional 90 min at RT. The reaction was quenched by the addition of 6 mL brine, followed by the addition of

EtOAc (10 mL). The reaction mixture was stirred for 10 min and subsequently transferred into a separation funnel. The phases were separated and the aqueous layer was extracted with EtOAc (2x15 mL). The combined organic extracts were dried over Na_2SO_4 and the solvents removed concentrated under reduced pressure. The residue was purified via flash chromatography (375 g SiO_2, dichloromethane/MeOH = 20:1 (v/v)) to furnish a pale yellow oily residue. The residue was dissolved in 50 mL EtOAc and the organic layer washed with H_2O (3x50 mL) The organic layer was dried over Na_2SO_4, the solvent removed under reduced pressure and the residue dried *in vacuo* to yield the desired product **30a** as a colorless solid.

$C_{42}H_{58}FN_5O_8$ [779.95 g/mol].

Yield: 107 mg (0.137 mmol, 48%, 2 steps from **28a**), colorless solid.

R_f = 0.21 (dichloromethane/MeOH = 20:1 (v/v); staining: CAM).

$[\alpha]_D^{24}$ = -23.8 (c = 0.86, $CHCl_3$).

m.p. = 88-90 °C.

^{1}H NMR (300 MHz, $CDCl_3$, mixture of 2 rotamers in 5:1 ratio): δ = 8.96 (bs, 1H, *H*-37), 7.38 (d, ^3J = 7.6 Hz, 1H, *H*-32), 7.22-6.84 (m, 9H, *H*-46, 45, 44, 43, 38, 35, 34, 33, 27), 6.06 (d, ^3J = 9.8 Hz, 1H, *H*-12), 4.97 (d, ^3J = 8.6 Hz, 1H, *H*-6), 4-59-4.55 (m, 1H, *H*-28), 3.80-3.71 (m, 1H, *H*-7), 3.70-3.44 (m, 5H, *H*-40, 17, 13), 3.33-3.05 (m, 3H, *H*-29, 25), 2.99-2.82 (m, 1H, *H*-22), 2.76-2.60 (m, 1H, *H*-22), 2.57-2.40 (m, 2H, *H*-41), 2.39-2.25 (m, 1H, *H*-20), 2.16-1.54 (m, 5H, *H*-24, 19, 16, 8), 1.38-1.26 (m, 9H, *H*-4, 3, 2), 1.17-1.11 (m, 1H, *H*-22), 0.99-0.61 (m, 14H, *H*-24, 23, 15, 14, 10, 9).

^{13}C NMR (75 MHz, $CDCl_3$, mixture of 2 rotamers in 5:1 ratio): δ = 175.2 (*C*-21), 172.8 (*C*-39), 172.7 (*C*-26), 172.2 (*C*-11), 163.4 (*C*-47), 156.2 (*C*-5), 136.3 (*C*-42), 134.5 (*C*-36), 130.7 (*C*-44), 130.6 (*C*-43), 127.6 (*C*-31), 123.4 (*C*-28), 122.4 (*C*-34), 119.9 (*C*-33), 118.0 (*C*-32), 115.5 (*C*-46), 115.2 (*C*-45), 111.8 (*C*-35), 109.8 (*C*-30), 80.4 (*C*-1), 71.1 (*C*-17), 60.8 (*C*-7), 60.1 (*C*-25), 59.3 (*C*-13), 53.7 (*C*-28), 52.6 (*C*-40), 46.2 (*C*-22), 43.9 (*C*-20), 38.8 (*C*-41), 37.0 (*C*-19), 31.3 (*C*-24), 29.8 (*C*-8), 28.5 (*C*-4, 3, 2), 28.0 (*C*-16), 26.2 (*C*-29), 21.8 (*C*-23), 20.6 (*C*-14), 19.9 (*C*-10), 18.2 (*C*-9), 16.7 (*C*-15).

^{19}F NMR (470 MHz, $CDCl_3$): δ = -116.1 (s).

HRMS (MALDI-TOF): *m/z* calcd. for $C_{42}H_{58}FN_5O_8Na$ $[M+Na]^+$: 802.4167; found: 802.3501.

2.3.30 ((2*R*,4*R*,5*S*)-5-((*S*)-2-Ammonio-3-methylbutanamido)-2-(4-fluorobenzyl)-4-hydroxy-6-methylheptanoyl)-L-prolyl-L-tryptophanate (31a)

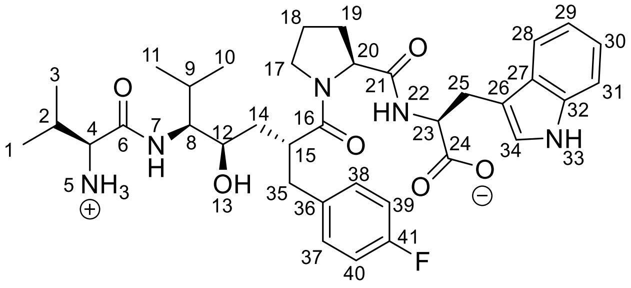

31a

Saponification: In a 25 mL round bottom flask, equipped with magnetic stirring bar, 84 mg (0.108 mmol, 1 eq.) of compound **30a** were dissolved in 1 mL THF. Afterwards, a solution of 36 mg (0.862 mmol, 8 eq.) LiOHxH_2O in 3 mL H_2O was added and the reaction mixture was stirred at RT. After 120 min TLC indicated full conversion. Then, 1 M HCl was added until a pH of ~3 was achieved. Subsequently, 10 mL of EtOAc were added and the mixture transferred into a separation funnel. The phases were separated and the aqueous layer was extracted with EtOAc (2x5 mL). The combined organic extracts were dried over Na_2SO_4 and the solvents removed under reduced pressure. The residue was dried *in vacuo* for 30 min to yield the saponified intermediate as a colorless solid.

Boc-Deprotection: In a 25 mL round bottom flask, equipped with magnetic stirring bar, the crude intermediate was dissolved in 1.5 mL 2,2,2-trifluoroethanol, followed by the addition of 32 µL (0.431 mmol, 4 eq.) ethanethiol and 194 mg (0.862 mmol, 8 eq.) zinc dibromide. The white turbid reaction mixture was stirred vigorously at RT for 40 h. Afterwards, the reaction mixture was concentrated and the residue dissolved in mixture of H_2O (500 µL), acetonitrile (490 µL) and DMSO (10 µL) and purified by preparative HPLC (*CLF_NucleodurC18_pc001_HCOOH_5to95*) to furnish compound **31a** as colorless solid.

$C_{36}H_{48}FN_5O_6$ [665.81 g/mol].

Yield: 21.0 mg (0.032 mmol, 29%), colorless solid.

$[\alpha]_D^{24}$ = -19.0 (c = 0.53, MeOH).

m.p. = 168-170 °C.

^{1}H NMR (500 MHz, MeOD, complex mixture of 2 rotamers in 4:1 ratio): δ = 8.36 (br s, 1H, *H*-33), 7.59 (d, ^{3}J = 8.4 Hz, 1H, *H*-28), 7.33 (d, ^{3}J = 8.4 Hz, 1H, *H*-31), 7.13-6.91 (m, 7H, *H*-40, 39, 38, 37, 34, 30, 29), 4.66-4.58 (m, 1H, *H*-23), 3.83-3.64 (m, 3H, *H*-12, 8, 4), 3.52-3.35 (m, 2H, *H*-25, 20), 3.21-3.05 (m, 2H, *H*-25, 17), 2.98-2.90 (m, 1H, *H*-17), 2.74-2.53 (m, 3H, *H*-35, 15), 2.29-2.07 (m, 2H, *H*-9, 2), 1.95-1.86 (m, 1H, *H*-14), 1.66-1.53 (m, 2H, *H*-19, 14), 1.18-0.77 (m, 15H, *H*-19, 18, 11, 10, 3, 1).

^{13}C NMR (125 MHz, MeOD, complex mixture of 2 rotamers in 4:1 ratio): δ = 177.5 (*C*-24, 16), 173.4 (*C*-21), 170.2 (*C*-6), 164.0 (*C*-41), 138.0 (*C*-6), 136.2 (*C*-36), 131.8 (*C*-38), 131.7 (*C*-37), 129.0 (*C*-27), 124.3 (*C*-34), 122.4 (*C*-30), 119.9 (*C*-29), 119.4 (*C*-28), 116.3 (*C*-40), 116.1 (*C*-39), 112.3 (*C*-31), 112.0 (*C*-26), 71.1 (*C*-12), 62.1 (*C*-20), 61.1 (*C*-8), 60.1 (*C*-4), 56.3 (*C*-23), 47.5 (*C*-17), 45.4 (*C*-15), 39.7 (*C*-35), 37.6 (*C*-14), 32.1 (*C*-2), 31.6 (*C*-19), 29.4 (*C*-9), 28.3 (*C*-25), 22.5 (*C*-18), 21.0 (*C*-11), 19.6 (*C*-3), 18.1 (*C*-10), 17.9 (*C*-1).

^{19}F NMR (470 MHz, $CDCl_3$): δ = -118.6 (s), -119.0 (s).

HRMS (MALDI-TOF): *m/z* calcd. for $C_{36}H_{48}FN_5O_6Na$ $[M+Na]^+$: 688.3486; found: 688.4046.

2.3.31 *tert*-Butyl ((1*S*)-1-((2*R*)-4-(hydroxy(p-tolyl)methyl)-5-oxotetrahydrofuran-2-yl)-2-methylpropyl)carbamate (24b)

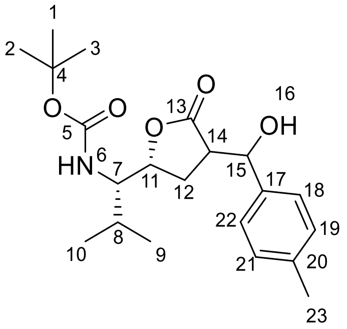

24b

A 100 mL three-necked round bottom flask, equipped with gas valve and magnetic stirring bar, was dried (evacuated, heated, N_2-purged) and charged with 500 mg (1.95 mmol, 1 eq.) lactone **16** and 20 mL abs. THF. The reaction mixture was cooled to -78 °C in a dry ice/acetone cooling bath followed by the dropwise addition of 2.90 mL (5.83 mmol, 3 eq.) LDA solution (2.0 M in THF). The deep orange reaction mixture was stirred at -78 °C for additional 45 min. Subsequently, 344 µL (2.93 mmol, 1.5 eq.) *p*-tolualdehyde were added dropwise within 5 min. The reaction mixture turned yellow after a few min and was stirred for additional 45 min at -78 °C until TLC indicated full conversion. The reaction was quenched by the addition of 10 mL satd. NH_4Cl and was allowed to warm up to RT followed by the addition of 30 mL H_2O and 50 mL EtOAc. The phases were separated and the aqueous layer extracted with EtOAc (2x30 mL). The combined organic layers were washed with 1 M HCl (50 mL), brine (50 mL), dried over Na_2SO_4 and the solvent was removed under reduced pressure. The residue was purified via flash chromatography (270 g SiO_2, cyclohexane/EtOAc 7:1 to 2:1 (v/v)) to furnish two diastereomers, which were separated and characterized separately.

$C_{21}H_{31}NO_5$ [377.48 g/mol].

Yield: unidentified diastereomer A: 159 mg, unidentified diastereomer B: 310 mg, total: 469 mg (1.24 mmol, 63%), colorless solid.

R_f = 0.29, 0.21 (cyclohexane/EtOAc = 3:1 (v/v); staining: $KMnO_4$).

m.p. = 92-94 °C.

^{1}H NMR (300 MHz, $CDCl_3$, unidentified diastereomer A): δ = 7.24 (d, ^3J = 7.6 Hz, 2H, *H*-22, 21), 7.16 (d, ^3J = 7.6 Hz, 2H, *H*-19, 18), 5.38- 5.31 (m, 1H, *H*-15), 4.46-4.19 (m, 2H, *H*-11, 6),

3.68-3.26 (m, 1H, *H*-7), 3.14-2.96 (m, 1H, *H*-14), 2.33 (s, 3H, *H*-23), 2.12-1.81 (m, 3H, *H*-12, 8), 1.47-1.33 (m, 9H, *H*-3, 2, 1), 0.88 (dd, ^3J = 19.3, 7.0 Hz, 6H, *H*-10, 9).

^{1}H NMR (300 MHz, $CDCl_3$, unidentified diastereomer B): δ = 7.25 (d, ^3J = 7.5 Hz, 2H, *H*-22, 21), 7.15 (d, ^3J = 7.6 Hz, 2H, *H*-19, 18), 4.78 (d, ^3J = 8.3 Hz, 1H, *H*-15), 4.29 (d, ^3J = 10.0 Hz, 1H, *H*-11), 4.22-4.09 (m, 1H, *H*-6), 3.63-3.37 (m, 1H, *H*-7), 3.25-3.04 (m, 1H, *H*-14), 2.32 (s, 3H, *H*-23), 2.08-1.84 (m, 3H, *H*-12, 8), 1.43-1.33 (m, 9H, *H*-3, 2, 1), 0.95-0.78 (m, 6H, *H*-10, 9).

2.3.32 *tert*-Butyl ((*S*)-2-methyl-1-((2*R*,4*R*)-4-(4-methylbenzyl)-5-oxotetrahydrofuran-2-yl)propyl)carbamate (25b)

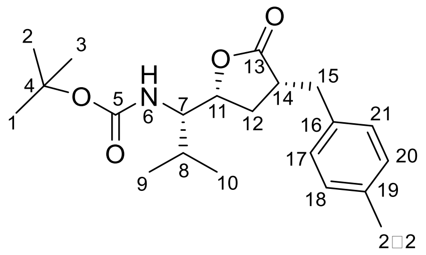

25b

Mesylation: A 100 mL round bottom flask, equipped with a Schlenk adapter and magnetic stirring bar, was dried (evacuated, heated, N_2-purged) and charged with 439 mg (1.16 mmol, 1 eq.) **24b**, 12 mL abs. dichloromethane and 806 µL (5.81 mmol, 5 eq.) triethylamine. Subsequently, 607 mg (3.48 mmol, 3 eq.) methanesulfonic anhydride were added portionwise within 3 min and the reaction mixture heated to 35 °C and stirred overnight. After 23 h the reaction was quenched by the addition of 50 mL H_2O. The mixture was transferred into a separation funnel and additional 30 mL dichloromethane were added. The phases were separated and the aqueous layer was extracted with dichloromethane (3x40 mL). The combined organic layers were washed with brine (50 mL), dried over Na_2SO_4 and the solvent was removed under reduced pressure to furnish a deep red solid residue which was used in the next step without further purification.

Elimination: A 50 mL round bottom flask, equipped with a Schlenk adapter and magnetic stirring bar, was dried (evacuated, heated, N_2-purged) and charged with the crude residue of the previous step and 8 mL abs. ethanol were added, followed by the addition of 323 µL (2.33 mmol, 2 eq.) triethylamine. The reaction mixture was heated to 50 °C and stirred at this temperature for 18 h. After this period the reaction mixture was allowed to warm up to RT and concentrated to a small volume. The residue was purified via flash chromatography (225 g SiO_2, cyclohexane/EtOAc 5:1 to 3:1 (v/v)) to furnish the corresponding olefin as a colorless solid

Hydrogenation: In a two-necked round bottom flask, equipped with gas valve and magnetic stirring bar, the olefin was dissolved in 10 mL THF and 27 mg (0.46 mmol, 0.4 eq) Raney®-Nickel (slurry in H_2O) were added. Nitrogen atmosphere was exchanged by hydrogen (3×evacuation/purging) and the reaction mixture was stirred under a H_2 balloon atmosphere at 40 °C for 24 h. After NMR indicated full conversion, the catalyst was removed under an argon atmosphere by filtration through a Schlenk-frit containing a plug of Celite® and the filter cake

was washed with EtOAc (3×20 mL). The filtrate was concentrated and dried *in vacuo* to furnish the pure diastereomer as a colorless solid.

$C_{21}H_{31}NO_4$ [361.48 g/mol].

Yield: 236 mg (0.65 mmol, 56%, 3 steps from **24b**), colorless solid.

R_f (olefin) = 0.42 (cyclohexane/EtOAc = 3:1 (v/v); staining: $KMnO_4$).

$[\alpha]_D^{24}$ = -45.8 (c = 2.03, $CHCl_3$).

m.p. = 76-79 °C.

^{1}H NMR (300 MHz, $CDCl_3$): δ = 7.09 (s, 4H, *H*-21, 20, 18, 17), 4.33 (d, ^3J = 8.9 Hz, 1H, *H*-6), 4.23-4.10 (m, 1H, *H*-11), 3.75-3.49 (m, 1H, *H*-7), 3.26 (d, ^3J = 12.2 Hz, 1H, *H*-15), 2.89-2.74 (m, 1H, *H*-14), 2.73-2.57 (m, 1H, *H*-15), 2.36-2.19 (m, 4H, *H*-22, 12), 2.13-1.99 (m, 1H, *H*-12), 1.96-1.76 (m, 1H, *H*-8), 1.42 (s, 9H, *H*-3, 2, 1), 0.93 (d, ^3J = 6.5 Hz, 3H, *H*-9), 0.85 (d, ^3J = 6.5 Hz, 3H, *H*-10).

^{13}C NMR (75 MHz, $CDCl_3$): δ = 177.8 (*C*-13), 156.6 (*C*-5), 136.3 (*C*-16), 135.7 (*C*-19), 129.4 (*C*-21, 17), 128.4 (*C*-20, 18), 79.9 (*C*-11), 78.2 (*C*-4), 58.3 (*C*-7), 42.7 (*C*-14), 36.0 (*C*-15), 32.2 (*C*-12), 28.6-28.2 (m, *C*-8, 3, 2, 1), 21.1 (*C*-22), 19.9 (*C*-9), 16.0 (*C*-10).

HRMS (MALDI-TOF): *m/z* calcd. for $C_{21}H_{31}NO_4$ Na $[M+Na]^+$: 384.2151; found: 384.2204.

2.3.33 (2*R*,4*R*,5*S*)-5-((*tert*-Butoxycarbonyl)amino)-4-((tert-butyldimethylsilyl)oxy)-6-methyl-2-(4-methylbenzyl)heptanoic acid (27b)

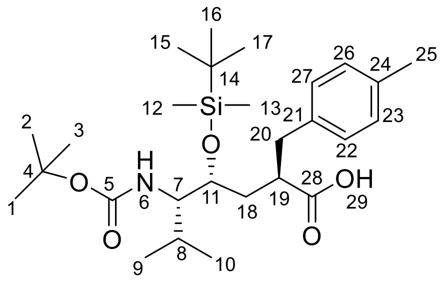

27b

Lactone opening: A 50 mL round bottom flask with magnetic stirring bar was charged with 221 mg (0.61 mmol, 1 eq.) alkylated lactone **25b** and 2 mL THF. A solution of 103 mg (2.44 mmol, 4 eq.) LiOHxH_2O in 3 mL H_2O was added and the turbid reaction mixture was stirred vigorously at RT. After 75 min TLC indicated full conversion and 6 mL Et_2O were added. The reaction mixture was cooled to 0 °C in an ice bath, followed by the careful addition of citric acid solution (25% in H_2O) until a pH of 4 was adjusted. Subsequently, the reaction mixture was transferred into a separation funnel and the phases were separated. The aqueous layer was extracted with Et_2O (2x10 mL). The combined organic layers were washed with H_2O (10 mL), brine (10 mL), dried over Na_2SO_4 and the solvent was evaporated under reduced pressure (T < 30 °C). The residue was dried *in vacuo* for 30 min to furnish the intermediate product as a colorless solid. The crude product was used in the next step without further purification.

Silylation: A 100 mL three-necked round bottom flask, equipped with gas valve and magnetic stirring bar, was dried (evacuated, heated, N_2-purged) and charged with the crude product of the previous step and 3.5 mL abs. dichloromethane were added. After the addition of 293 µL (3.67 mmol, 6 eq.) *N*-methylimidazole the reaction mixture was cooled to 0 °C in an ice bath. After 10 min, 931 mg (3.67 mmol, 6 eq.) iodine were added and the deep red reaction mixture was stirred for additional 5 min. 277 mg (1.83 mmol, 3 eq.) TBSCl were added in one portion to the reaction mixture. After the addition, the ice bath was removed and the deep reaction solution stirred at RT overnight. Afterwards, 15 mL Et_2O were added and the mixture was transferred into a separation funnel. 15 mL 1 M $Na_2S_2O_3$ were added and the organic layer was washed until total decoloration occurred. The phases were separated and the aqueous layer was extracted with Et_2O (2x10 mL). The combined organic layers were washed with citric acid solution [(25% in H_2O), 10 mL], brine (10 mL), dried over Na_2SO_4 and the solvents removed

under reduced pressure. The yellow oily residue was used in the next step without further purification.

Ester hydrolysis: In a 25 mL round bottom flask, equipped with magnetic stirring bar the residue of the previous step was dissolved in 2.5 mL MeOH and 80 µL (0.06 mmol, 0.1 eq.) citric acid (25% in H_2O) were added. The yellow reaction mixture was stirred at RT overnight. After 23 h the reaction mixture was concentrated and purified via flash chromatography (120 g SiO_2, cyclohexane/EtOAc 6:1 to 2:1 (v/v)) to give the protected acid **27b** as a colorless oil.

$C_{27}H_{47}NO_5Si$ [493.76 g/mol].

Yield: 238 mg (0.48 mmol, 79%, 3 steps from **25b**), colorless oil.

R_f = 0.43 (cyclohexane/EtOAc = 2:1 (v/v); staining: $KMnO_4$, stains white with $KMnO_4$).

$[\alpha]_D^{24}$ = -7.0 (c = 0.35, $CHCl_3$).

^{1}H NMR (300 MHz, $CDCl_3$): δ = 7.07 (s, 4H, *H*-27, 26, 23, 22), 4.61 (d, ^3J = 9.9 Hz, 1H, *H*-6), 3.82-3.59 (m, 1H, *H*-11), 3.52-3.27 (m, 1H, *H*-7), 3.00-2.85 (m, 1H, *H*-20), 2.81-2.58 (m, 2H, *H*-20, 19), 2.30 (s, 3H, *H*-25), 1.96-1.77 (m, 1H, *H*-18), 1.73-1.53 (m, 2H, *H*-18, 8), 1.42 (s, 9H, *H*-3, 2, 1), 0.88-0.74 (m, 15H, *H*-17, 16, 15, 10, 9), 0.09-0.04 (m, 6H, *H*-13, 12).

^{13}C NMR (75 MHz, $CDCl_3$): δ = 179.4 (*C*-28),155.2 (*C*-5), 135.2 (*C*-21), 128.3 (*C*-27, 22), 128.0 (*C*-26, 23), 127.9 (*C*-24), 78.3 (*C*-4), 70.8 (*C*-11), 57.2 (*C*-7), 42.8 (*C*-19), 37.2 (*C*-20), 34.4 (*C*-18), 27.6 (*C*-3, 2, 1), 27.4 (*C*-8), 25.0 (*C*-17, 16, 15), 20.2 (*C*-9), 20.0 (*C*-25), 18.1 (*C*-10),-4.3 (*C*-13), -4.6 (*C*-12).

HRMS could not be determined.

2.3.34 Methyl ((2*R*,4*R*,5*S*)-5-((*tert*-butoxycarbonyl)amino)-4-((*tert*-butyldimethylsilyl)oxy)-6-methyl-2-(4-methylbenzyl)heptanoyl)-L-prolyl-L-tryptophanate (28b)

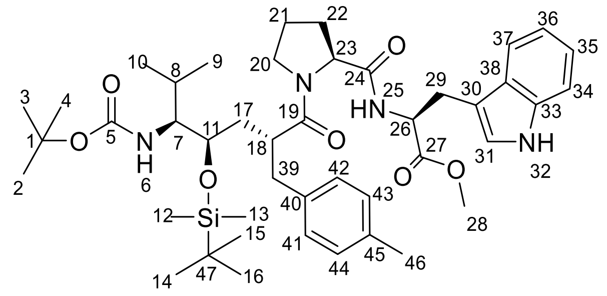

28b

Boc-Deprotection of Boc-Trp-OMe: In a 50 mL round bottom flask, equipped with magnetic stirring bar, 183 mg (0.44 mmol, 1.2 eq.) Boc-Trp-OMe peptide were dissolved in 272 µL (4.4 mmol, 10 eq.) ethanethiol. Subsequently, 850 µL (11.0 mmol, 30 eq.) trifluoroacetic acid were added and the yellowish reaction mixture was stirred vigorously at RT. After 1 h the volatiles were removed under reduced pressure with a cooling trap. The residue was dissolved in 15 mL EtOAc and 25% aqueous ammonia (10 mL) were added and the reaction mixture was stirred for 5 min. The phases were separated and the aqueous layer was extracted with EtOAc (2x10 mL). The combined organic layers were dried over Na_2SO_4 and the solvent was removed under reduced pressure. The residue was dried *in vacuo* to furnish the deprotected intermediate as a colorless solid.

Coupling: A 50 mL round bottom flask, equipped with Schlenk adapter and magnetic stirring bar, was dried (evacuated, heated, N_2-purged) and charged with 181 mg (0.36 mmol, 1 eq.) **27b** which was dissolved in 0.75 mL abs. DMF, followed by the addition of 64 µL (0.36 mmol, 1 eq.) Hünig`s base. The flask was cooled to 0 °C in an ice bath and 168 mg (0.44 mmol, 1.2 eq.) HBTU were added to the reaction mixture. The previously deprotected peptide was dissolved in 1.5 abs. DMF and 128 µL (0.74 mmol, 2 eq.) Hünig`s base were added. After 5 min, the solution of the deprotected intermediate was added to the solution of acid **27b** with a syringe. After the addition was complete, the ice bath was removed and the yellow reaction mixture was stirred at RT for additional 2 h. Subsequently, the reaction mixture was quenched by the addition of brine (5 mL). Additionally, 10 mL of EtOAc were added and the mixture was stirred vigorously for 10 min. Afterwards, the mixture was transferred into a separation funnel and the phases were separated. The aqueous layer was extracted with EtOAc (2x20 mL). The combined organic layers were washed with brine (20 mL), dried over Na_2SO_4 and the solvents were

removed under reduced pressure. The residue was purified via flash chromatography (150 g SiO_2, dichloromethane/MeOH 80:1 to 30:1 (v/v)) to furnish a pale orange oily residue. The residue was dissolved in 50 mL EtOAc and the organic layer washed with H_2O (5x50 mL) in order to remove remaining DMF and tetramethylurea. The organic layer was dried over Na_2SO_4, the solvent removed under reduced pressure and the residue dried *in vacuo* to yield the desired product as a colorless solid.

$C_{44}H_{66}N_4O_7Si$ [791.12 g/mol].

Yield: 105 mg (0.132 mmol, 36%, 2 steps from **27b**), colorless solid.

R_f = 0.53 (dichloromethane/MeOH = 30:1 (v/v); staining: $KMnO_4$).

$[\alpha]_D^{24}$ = -12.9 (c = 0.47, $CHCl_3$).

m.p. = 77-79 °C.

^1H NMR (300 MHz, $CDCl_3$): δ = 8.11 (br s, 1H, *H*-32), 7.52 (d, ^3J = 7.5 Hz, 1H, *H*-37), 7.41-7.30 (m, 1H, *H*-25), 7.24-6.84 (m, 8H, *H*-44, 43, 42, 41, 36, 35, 34, 31), 4.76 (dd, ^3J = 8.9, 6.1 Hz, 1H, *H*-26), 4.63-4.46 (m, 2H, *H*-23, 6), 3.71-3.59 (m, 4H, *H*-28, 11), 3.56-3.46 (m, 1H, *H*-20), 3.40-3.16 (m, 3H, *H*-29, 7), 3.02-2.90 (m, 1H, *H*-20), 2.77-2.45 (m, 3H, *H*-39, 18), 2.34-2.19 (m, 4H, *H*-46, 22), 1.94-1.50 (m, 6H, *H*-22, 21, 17, 8), 1.41 (s, 9H, *H*-3, 2, 1), 0.91-0.78 (m, 24H, *H*-51, 49, 48, 16, 15, 14, 10, 9), 0.16-0.02 (m, 12H, *H*-47, 46, 13, 12).

^{13}C NMR (75 MHz, $CDCl_3$): δ = 175.1 (*C*-19), 172.4 (*C*-27), 171.2 (*C*-24), 156.0 (*C*-5), 136.2 (*C*-33), 136.0 (*C*-45, 40), 129.2 (*C*-44, 43), 128.9 (*C*-42, 41), 127.7 (*C*-38), 123.4 (*C*-31), 122.1 (*C*-35), 119.6 (*C*-36), 118.7 (*C*-37), 111.3 (*C*-34), 110.3 (*C*-31), 79.0 (*C*-1), 72.1 (*C*-11), 60.1 (*C*-23), 57.8 (*C*-7), 53.5 (*C*-26), 52.4 (*C*-28), 47.3 (*C*-20), 41.9 (*C*-18), 38.6 (*C*-39), 35.5 (*C*-17), 28.6 (*C*-4, 3, 2), 27.9 (*C*-8), 27.5 (*C*-29), 27.4 (*C*-22), 26.0 (*C*-16, 15, 14), 24.8 (*C*-21), 21.4 (*C*-10), 21.1 (*C*-46), 18.4 (*C*-9), 18.0 (*C*-47), -4.1 (*C*-12), -4.7 (*C*-13).

HRMS (MALDI-TOF): *m/z* calcd. for $C_{44}H_{66}N_4O_7SiNa$ $[M+Na]^+$: 813.4598; found: 813.4868.

2.3.35 Methyl ((2*R*,4*R*,5*S*)-5-((*S*)-2-((*tert*-butoxycarbonyl)amino)-3-methylbutanamido)-4-hydroxy-6-methyl-2-(4-methylbenzyl)heptanoyl)-L-prolyl-L-tryptophanate (30b)

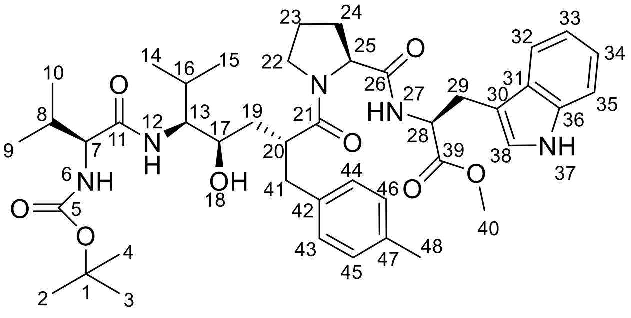

30b

Tandem deprotection of Boc- and TBS-moeity: In a 50 mL round bottom flask, equipped with magnetic stirring bar, 141 mg (0.178 mmol, 1 eq.) **28b** were dissolved in 2 mL 2,2,2-trifluoroethanol. Afterwards, 52 µL (0.713 mmol, 4 eq.) ethanethiol were added, followed by the addition of 321 mg (1.43 mmol, 8 eq.) zinc dibromide. The reaction mixture was stirred vigorously at RT and after 45 min the formation of a white precipitate could be observed. The turbid reaction mixture was stirred for additional 2 h, followed by the addition of 25% aqueous ammonia (5 mL) and EtOAc (15 mL). The reaction mixture was stirred for 15 min and subsequently transferred into a separation funnel and the phases were separated. The aqueous layer was extracted with EtOAc (2x10 mL). The combined organic layers were dried over Na_2SO_4 and the solvents were removed under reduced pressure. The residue was dried *in vacuo* for 30 min to furnish the deprotected intermediate as a colorless solid.

Coupling: A 50 mL round bottom flask, equipped with magnetic stirring bar and Schlenk adapter, was dried (evacuated, heated, N_2-purged) and charged with 47 mg (0.214 mmol, 1.2 eq.) Boc-L-Val-OH and 0.4 mL abs. DMF, followed by the addition of 31 µL (0.178 mmol, 1 eq.) Hünig's base. The flask was cooled to 0 °C in an ice bath. Subsequently, 81 mg (0.214 mmol, 1.2 eq.) HATU were added to the reaction mixture and stirred for additional 5 min. In a second 50 mL round bottom flask, equipped with magnetic stirring bar and Schlenk adapter, the previously deprotected peptide was dissolved in 1 mL abs. DMF and 62 µL (0.356 mmol, 2 eq.) Hünig's base were added. Afterwards, the solution of the deprotected peptide was added to the solution of the activated acid using a syringe. After the addition was complete, the ice bath was removed and the deep yellow reaction mixture was stirred for additional 3 h at RT. The reaction was quenched by the addition of 5 mL brine, followed by the addition of EtOAc

(15 mL). The reaction mixture was stirred for 15 min and subsequently transferred into a separation funnel. The phases were separated and the aqueous layer was extracted with EtOAc (2x15 mL). The combined organic extracts were dried over Na_2SO_4 and the solvents removed concentrated under reduced pressure. The residue was purified via flash chromatography (300 g SiO_2, dichloromethane/MeOH = 20:1 (v/v)) to furnish an oily residue. The residue was dissolved in 50 mL EtOAc and the organic layer washed with H_2O (4x40 mL) in order to remove remaining DMF. The organic layer was dried over Na_2SO_4, the solvent removed under reduced pressure and the residue dried *in vacuo* to yield the desired product **30b** as a colorless solid.

$C_{43}H_{61}N_5O_8$ [775.99 g/mol].

Yield: 52 mg (0.067 mmol, 38%, 2 steps from **28b**), colorless solid.

R_f = 0.21 (dichloromethane/MeOH = 20:1 (v/v); staining: CAM).

$[\alpha]_D^{24}$ = -26.4 (c = 0.89, $CHCl_3$).

m.p. = 92-94 °C.

^{1}H NMR (300 MHz, $CDCl_3$): δ = 9.03 (bs, 1H, *H*-37), 7.51 (d, ^3J = 7.5 Hz, 1H, *H*-32), 7.36 (d, ^3J = 7.6 Hz, 1H, *H*-27), 7.22-6.84 (m, 8H, *H*-46, 45, 44, 43, 38, 35, 34, 33), 6.08 (d, ^3J = 9.9 Hz, 1H, *H*-12), 5.08 (d, ^3J = 8.7 Hz, 1H, *H*-6), 4-78-4.60 (m, 1H, *H*-28), 3.93-3.81 (m, 1H, *H*-7), 3.80-3.61 (m, 5H, *H*-40, 17, 13), 3.40-3.19 (m, 3H, *H*-29, 25), 3.12-2.99 (m, 1H, *H*-22), 2.93-2.78 (m, 1H, *H*-22), 2.69-2.52 (m, 2H, *H*-41), 2.51-2.37 (m, 1H, *H*-20), 2.33-1.64 (m, 8H, *H*-48, 24, 19, 16, 8), 1.49-1.42 (m, 9H, *H*-4, 3, 2), 1.37-1.32 (m, 1H, *H*-22), 1.08-0.83 (m, 14H, *H*-24, 23, 15, 14, 10, 9).

^{13}C NMR (75 MHz, $CDCl_3$): δ = 175.6 (*C*-21), 172.8 (*C*-39), 172.7 (*C*-26), 172.4 (*C*-11), 156.2 (*C*-5), 136.3 (*C*-47, 42), 135.6 (*C*-36), 129.2 (*C*-46, 45), 129.0 (*C*-44, 43), 127.6 (*C*-31), 123.4 (*C*-38), 122.3 (*C*-34), 119.9 (*C*-33), 118.1 (*C*-32), 111.7 (*C*-35), 110.0 (*C*-30), 80.3 (*C*-1), 71.1 (*C*-17), 60.8 (*C*-7), 60.1 (*C*-25), 59.2 (*C*-13), 53.7 (*C*-28), 52.6 (*C*-40), 46.2 (*C*-22), 44.1 (*C*-20), 39.5 (*C*-41), 37.1 (*C*-19), 31.0 (*C*-24), 29.8 (*C*-8), 28.5 (*C*-4, 3, 2), 28.0 (*C*-16), 26.2 (*C*-29), 21.8 (*C*-23), 21.1 (*C*-48), 20.6 (*C*-14), 19.9 (*C*-10), 18.3 (*C*-9), 16.7 (*C*-15).

HRMS (MALDI-TOF): *m/z* calcd. for $C_{43}H_{61}N_5O_8Na$ $[M+Na]^+$: 798.4418; found: 798.5275.

2.3.36 ((2*R*,4*R*,5*S*)-5-((*S*)-2-Ammonio-3-methylbutanamido)-4-hydroxy-6-methyl-2-(4-methylbenzyl)heptanoyl)-L-prolyl-L-tryptophanate (31b)

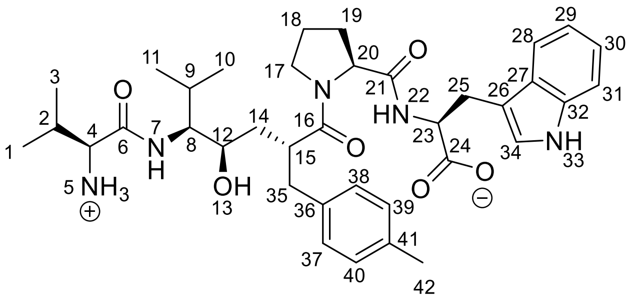

31b

Saponification: In a 25 mL round bottom flask, equipped with magnetic stirring bar, 45 mg (0.058 mmol, 1 eq.) **30b** were dissolved in 0.5 mL THF. Afterwards, a solution of 20 mg (0.464 mmol, 8 eq.) LiOHxH_2O in 1.5 mL H_2O was added and the reaction mixture was stirred at RT. After 90 min TLC indicated full conversion, followed by the addition of 1 M HCl until a pH of ~4 was achieved. Subsequently, 10 mL EtOAc were added and the mixture transferred into a separation funnel. The phases were separated and the aqueous layer was extracted with EtOAc (2x5 mL). The combined organic extracts were dried over Na_2SO_4 and the solvents removed under reduced pressure. The residue was dried *in vacuo* for 30 min to yield the saponified intermediate as a colorless solid.

Boc-Deprotection: In a 25 mL round bottom flask, equipped with magnetic stirring bar, the crude intermediate was dissolved in 1.5 mL 2,2,2-trifluoroethanol, followed by the addition of 17 µL (0.232 mmol, 4 eq.) ethanethiol and 105 mg (0.464 mmol, 8 eq.) zinc dibromide. The reaction mixture turned pink immediately and was stirred vigorously at RT for 2 h. Afterwards, the reaction mixture was concentrated and the residue dissolved in a mixture of H_2O (600 µL), acetonitrile (390 µL) and DMSO (10 µL) and purified by preparative HPLC (*CLF_NucleodurC18_pc001_HCOOH_5to95*) to furnish compound **31b** as a colorless solid.

$C_{37}H_{51}N_5O_6$ [661.84 g/mol].

Yield: 13.8 mg (0.021 mmol, 36%, 2 steps from **30b**), colorless solid.

$[\alpha]_D^{24}$ = -17.6 (c = 0.94, MeOH).

m.p. = 160-162 °C.

^{1}H NMR (500 MHz, MeOD): δ = 8.35 (br s, 1H, *H*-33), 7.57 (d, ^{3}J = 7.9 Hz, 1H, *H*-28), 7.32 (d, ^{3}J = 8.0 Hz, 1H, *H*-31), 7.11-7.00 (m, 5H, *H*-38, 37, 34, 30, 29), 6.95 (d, ^{3}J = 7.8 Hz, 2H, *H*-40, 39), 4.63-4.57 (m, 1H, *H*-23), 3.80-3.68 (m, 3H, *H*-12, 8, 4), 3.51-3.37 (m, 2H, *H*-25, 20), 3.23-3.03 (m, 2H, *H*-25, 17), 2.95-2.87 (m, 1H, *H*-17), 2.72-2.58 (m, 3H, *H*-35, 15), 2.30-2.21 (m, 5H, *H*-42, 9, 2), 2.00-1.94 (m, 1H, *H*-14), 1.63-1.52 (m, 2H, *H*-19, 14), 1.16-0.67 (m, 15H, *H*-19, 18, 11, 10, 3, 1).

^{13}C NMR (125 MHz, MeOD): δ = 177.8 (*C*-24, 16), 173.5 (*C*-21), 170.2 (*C*-6), 138.0 (*C*-6), 137.4 (*C*-36), 137.0 (*C*-41), 130.2 (*C*-38, 37), 130.0 (*C*-40, 39), 129.0 (*C*-27), 124.3 (*C*-34), 122.4 (*C*-30), 119.9 (*C*-29), 119.4 (*C*-28), 112.2 (*C*-31), 111.9 (*C*-26), 71.2 (*C*-12), 62.1 (*C*-20), 61.2 (*C*-8), 60.2 (*C*-4), 56.2 (*C*-23), 47.4 (*C*-17), 45.6 (*C*-15), 40.4 (*C*-35), 37.8 (*C*-14), 32.0 (*C*-2), 31.6 (*C*-19), 29.3 (*C*-9), 28.2 (*C*-25), 22.0 (*C*-18), 21.1 (*C*-11), 21.0 (*C*-42), 19.6 (*C*-3), 18.0 (*C*-10), 17.4 (*C*-1).

HRMS (MALDI-TOF): *m/z* calcd. for $C_{37}H_{51}N_5O_6Na$ $[M+Na]^+$: 684.3737; found: 684.3685.

2.3.37 4-((*tert*-Butyldimethylsilyl)oxy)benzaldehyde

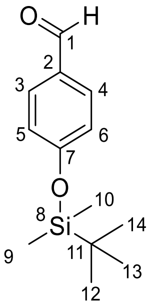

A 250 mL three-necked round bottom flask, equipped with gas valve and magnetic stirring bar, was dried (evacuated, heated, N_2-purged) and charged with 3.00 g (24.0 mmol, 1 eq.) 4-hydroxybenzaldehyde, 5.01 g (73.0 mmol, 3 eq.) imidazole and 50 mL abs. dichloromethane. The solution was cooled to 0 °C in an ice bath. 4.44 g (29.0 mmol, 1.2 eq.) TBSCl were added portionwise within a period of 20 min. The reaction mixture was stirred for additional 30 min at 0 °C and then at RT overnight. The yellow turbid reaction mixture was quenched by pouring the content in 100 mL of ice cold H_2O. The mixture was stirred for 15 min and subsequently transferred into a separation funnel. The phases were separated and the aqueous layer was extracted with dichloromethane (2x50 mL). The combined organic layers were washed with brine (100 mL), dried over Na_2SO_4 and the solvent was removed under reduced pressure. The residue was purified via flash chromatography (450 g SiO_2, cyclohexane/EtOAc 5:1 to 3:1 (v/v)) to give the desired product as a yellow oil.

$C_{13}H_{20}O_2Si$ [236.39 g/mol].

Yield: 3.88 g (16.4 mmol, 67%), yellow oil.

R_f = 0.70 (cyclohexane/EtOAc = 5:1 (v/v); staining: $KMnO_4$).

^{1}H NMR (300 MHz, $CDCl_3$): δ = 9.88 (s, 1H, *H*-1), 7.79 (d, ^3J = 8.6 Hz, 2H, *H*-4, 3), 6.94 (d, ^3J = 8.6 Hz, 2H, *H*-6, 5), 0.99 (s, 9H, *H*-14, 13, 12), 0.25 (s, 6H, *H*-10, 9).

^{13}C NMR (75 MHz, $CDCl_3$): δ = 191.0 (*C*-1), 161.6 (*C*-7), 132.0 (*C*-4, 3), 130.5 (*C*-2), 120.6 (*C*-6, 5), 25.7 (*C*-14, 13, 12), 18.4 (*C*-11), -4.2 (*C*-10, 9).

2.3.38 *tert*-Butyl ((1*S*)-1-((2*R*)-4-((4-((*tert*-butyldimethylsilyl)oxy)phenyl)(hydroxy)methyl)-5-oxotetrahydrofuran-2-yl)-2-methylpropyl)carbamate (24c)

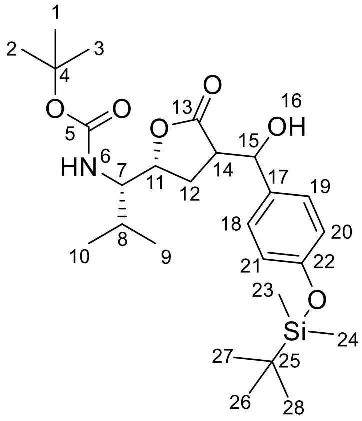

24c

A 100 mL three-necked round bottom flask, equipped with gas valve and magnetic stirring bar, was dried (evacuated, heated, N_2-purged) and charged with 800 mg (3.11 mmol, 1 eq.) lactone **16** and 20 mL abs. THF. The reaction mixture was cooled to -78 °C in a dry ice/acetone cooling bath followed by the dropwise addition of 4.66 mL (9.33 mmol, 3 eq.) LDA solution (2.0 M in THF). The orange reaction mixture was stirred at -78 °C for additional 50 min. Subsequently, 1.14 mL (4.66 mmol, 1.5 eq.) 4-((tert-Butyldimethylsilyl)oxy)benzaldehyde **6** were added in one portion. The reaction mixture turned yellow after a few min and was stirred for additional 45 min at -78 °C until TLC indicated full conversion. The reaction was quenched by the addition of 15 mL satd. NH_4Cl. The reaction mixture was allowed to come to RT, followed by the addition of 50 mL H_2O and 50 mL EtOAc. The phases were separated and the aqueous layer was extracted with EtOAc (2x50 mL). The combined organic layers were washed with brine (100 mL), dried over Na_2SO_4 and the solvent was removed under reduced pressure. The residue was purified via flash chromatography (375 g SiO_2, cyclohexane/EtOAc 7:1 to 2:1 (v/v)) to furnish two diastereomers, which were separated and characterized separately.

$C_{26}H_{43}NO_6Si$ [493.72 g/mol]

Yield: unidentified diastereomer A: 364 mg, unidentified diastereomer B: 647 mg, total: 1.01 g (2.05 mmol, 66%), colorless solid.

R_f = 0.21, 0.18 (cyclohexane/EtOAc = 3:1 (v/v); staining: $KMnO_4$).

m.p. = 96-98 °C.

^{1}H NMR (300 MHz, $CDCl_3$, unidentified diastereomer A): δ = 7.21 (d, ^3J = 8.3 Hz, 2H, *H*-21, 20), 6.81 (d, ^3J = 8.3 Hz, 2H, *H*-18, 19), 5.37-5.22 (m, 1H, *H*-15), 4.60-4.16 (m, 2H, *H*-11, 6), 3.84-3.32 (m, 1H, *H*-7), 3.12-2.74 (m, 1H, *H*-14), 2.47-2.28 (m, 1H, *H*-12), 2.16-1.77 (m, 2H, *H*-12, 8), 1.51-1.23 (m, 9H, *H*-3, 2, 1), 1.00-0.80 (m, 15H, *H*-28, 27, 26, 10, 9), 0.18 (s, 6H, *H*-24, 23).

^{1}H NMR (300 MHz, $CDCl_3$, unidentified diastereomer B): δ =7.23 (d, ^3J = 8.3 Hz, 2H, *H*-21, 20), 6.80 (d, ^3J = 8.3 Hz, 2H, *H*-18, 19), 4.81-4.64 (m, 1H, *H*-15), 4.40-4.05 (m, 2H, *H*-11, 6), 3.71-3.44 (m, 1H, *H*-7), 3.19-2.80 (m, 1H, *H*-14), 2.16-1.71 (m, 3H, *H*-12, 8), 1.47-1.30 (m, 9H, *H*-3, 2, 1), 1.0-0.78 (m, 15H, *H*-28, 27, 26, 10, 9), 0.17 (s, 6H, *H*-24, 23).

2.3.39 *tert*-Butyl ((*S*)-1-((2*R*, 4*R*)-4-(4-((*tert*-butyldimethylsilyl)oxy)benzyl)-5-oxotetrahydrofuran-2-yl)-2-methylpropyl)carbamate (25c)

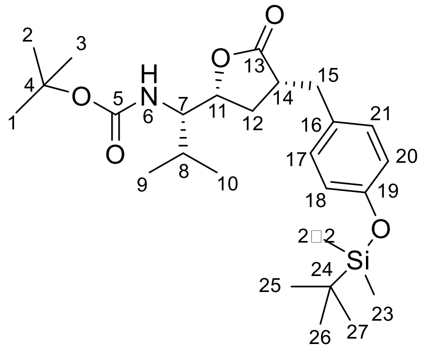

25c

Mesylation: A 50 mL round bottom flask, equipped with a Schlenk adapter and magnetic stirring bar, was dried (evacuated, heated, N_2-purged) and charged with 333 mg (0.67 mmol, 1 eq.) compound **24c**, 10 mL abs. dichloromethane and 468 µL (3.37 mmol, 5 eq.) triethylamine. Subsequently, 353 mg (2.02 mmol, 3 eq.) methanesulfonic anhydride were added in one portion and the reaction mixture heated to 35 °C and stirred overnight. After 23 h the reaction mixture was allowed to warm to RT and was quenched by the addition of 20 mL H_2O. The reaction mixture was transferred into a separation funnel and additional 20 mL dichloromethane were added. The phases were separated and the aqueous layer was extracted with dichloromethane (2x20 mL). The combined organic layers were washed with brine (50 mL), dried over Na_2SO_4 and the solvent was removed under reduced pressure to furnish a deep red solid residue, which was used in the next step without further purification.

Elimination: A 50 mL round bottom flask, equipped with a Schlenk adapter and magnetic stirring bar, was dried (evacuated, heated, N_2-purged) and charged with the crude residue of the previous step and 12 mL abs. ethanol were added, followed by the addition of 187 µL (1.35 mmol, 2 eq.) triethylamine. The reaction mixture was heated to 55 °C and stirred at this temperature for 16 h. After this period the reaction mixture was allowed to warm to RT and concentrated to a small volume. The residue was partioned between EtOAc (20 mL) and H_2O (20 mL). The aqueous layer was extracted with EtOAc (2x20 mL). The combined organic layers were washed with brine (50 mL), dried over Na_2SO_4 and the solvent was removed under reduced pressure to furnish the intermediate olefin as a yellow oil.

Hydrogenation: In a two-necked round bottom flask, equipped with gas valve and magnetic stirring bar, the crude olefin was dissolved in 8 mL THF and 15 mg (0.24 mmol, 0.4 eq) Raney®-Nickel (slurry in H_2O) were added. Nitrogen atmosphere was exchanged by hydrogen

(3×evacuation/purging) and the reaction mixture was stirred under H_2 balloon atmosphere at 40 °C for 48 h. After NMR indicated full conversion, the catalyst was removed under an argon atmosphere by filtration through a Schlenk-frit containing a plug of Celite® and the filter cake was washed with EtOAc (3×20 mL). The filtrate was concentrated and the residue was purified via flash chromatography (225 g SiO_2, cyclohexane/EtOAc 7:1 to 3:1) to furnish the pure diastereomer **25c** as a colorless solid.

$C_{26}H_{43}NO_5Si$ [477.72 g/mol]

Yield: 205 mg (0.43 mmol, 64%, 3 steps from **24c**), colorless solid.

R_f = 0.61 (cyclohexane/EtOAc = 2:1(v/v); staining: $KMnO_4$).

$[\alpha]_D^{24}$ = -40.7 (c = 0.73, $CHCl_3$).

m.p. = 80-82 °C.

^{1}H NMR (300 MHz, $CDCl_3$): δ = 7.09 (s, 4H, *H*-21, 20, 18, 17), 4.33 (d, ^3J = 8.9 Hz, 1H, *H*-6), 4.23-4.10 (m, 1H, *H*-11), 3.75-3.49 (m, 1H, *H*-7), 3.26 (d, ^3J = 12.2 Hz, 1H, *H*-15), 2.89-2.74 (m, 1H, *H*-14), 2.73-2.57 (m, 1H, *H*-15), 2.36-2.19 (m, 4H, *H*-22, 12), 2.13-1.99 (m, 1H, *H*-12), 1.96-1.76 (m, 1H, *H*-8), 1.42 (s, 9H, *H*-3, 2, 1), 0.93 (d, ^3J = 6.5 Hz, 3H, *H*-9), 0.85 (d, ^3J = 6.5 Hz, 3H, *H*-10).

^{13}C NMR (75 MHz, $CDCl_3$): δ = 177.9 (*C*-13), 156.1 (*C*-5), 154.4 (*C*-16), 130.2 (*C*-19), 129.9 (*C*-21, 17), 120.3 (*C*-20, 18), 79.9 (*C*-11), 78.2 (*C*-4), 58.3 (*C*-7), 42.9 (*C*-14), 35.7 (*C*-15), 32.2 (*C*-12), 29.8 (*C*-8), 28.4 (*C*-3, 2, 1), 25.8 (*C*-27, 26, 25), 20.0 (*C*-9), 18.3 (*C*-24), 16.0 (*C*-10), -4.3 (*C*-23, 22).

HRMS (MALDI-TOF): *m/z* calcd. for $C_{26}H_{43}NO_5SiNa$ $[M+Na]^+$: 500.2808; found: 500.2888.

2.3.40 (2*R*,4*R*,5*S*)-5-((*tert*-Butoxycarbonyl)amino)-4-((*tert*-butyldimethylsilyl)oxy)-2-(4-((*tert*-butyldimethylsilyl)oxy)benzyl)-6-methylheptanoic acid (27c)

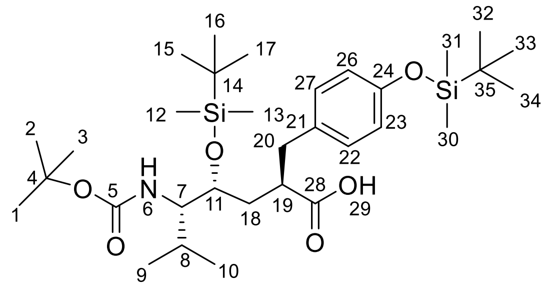

27c

Lactone opening: A 100 mL round bottom flask with magnetic stirring bar was charged with 1.04 g (2.18 mmol, 1 eq.) alkylated lactone **26c** and 11 mL THF. A solution of 366 mg (8.71 mmol, 4 eq.) LiOHxH_2O in 7 mL H_2O was added and the turbid reaction mixture was stirred vigorously at RT. After 165 min TLC indicated full conversion and 22 mL Et_2O were added. The reaction mixture was cooled to 0 °C in an ice bath, followed by the careful addition of citric acid (25% in H_2O) until a pH of 4 was adjusted. Subsequently, the reaction mixture was transferred into a separation funnel and the phases were separated. The aqueous layer was extracted with Et_2O (2x20 mL). The combined organic layers were washed with brine (40 mL), dried over Na_2SO_4 and the solvent was evaporated under reduced pressure (T < 30 °C). The residue was dried *in vacuo* for 30 min to furnish the intermediate product as a colorless solid. The crude product was used in the next step without further purification.

Silylation: A 100 mL three-necked round bottom flask, equipped with gas valve and magnetic stirring bar, was dried (evacuated, heated, N_2-purged) and charged with the crude product of the previous step and 9.0 mL abs. dichloromethane were added. After the addition of 1.04 mL (13.06 mmol, 6 eq.) *N*-methylimidazole the reaction mixture was cooled to 0 °C in an ice bath. After 10 min 3.31 g (13.06 mmol, 6 eq.) iodine were added and the deep red reaction mixture stirred for additional 10 min. 1.31 g (8.71 mmol, 4 eq.) TBSCl were added portionwise within a period of 5 min. After the addition was complete, the ice bath was removed and the deep reaction solution stirred at RT overnight. Afterwards, 30 mL Et_2O were added and the mixture was transferred into a separation funnel. 50 mL 1 M $Na_2S_2O_3$ were added and the organic layer was washed until total decoloration occurred. The phases were separated and the aqueous layer was extracted with Et_2O (2x50 mL). The combined organic layers were washed with brine (100

mL), dried over Na_2SO_4 and the solvents removed under reduced pressure. The yellow oily residue was used in the next step without further purification.

Ester hydrolysis: In a 50 mL round bottom flask, equipped with a magnetic stirring bar the residue of the previous step was dissolved in 6.5 mL MeOH and 165 µL (0.13 mmol, 0.1 eq.) citric acid solution (25% in H_2O) were added. The yellow reaction mixture was stirred at RT overnight. After 23 h the reaction mixture was concentrated and used in the next step without further purification.

Hydrogenation: In a three-necked round bottom flask, equipped with magnetic stirring bar, the crude product was dissolved in 21 mL MeOH and 755 µL (5.45 mmol, 2.5 eq) triethylamine were added. Under nitrogen stream, 48 mg palladium on charcoal (5 % palladium; 1 mol-%) were added to the reaction mixture. Nitrogen atmosphere was exchanged by hydrogen (3×evacuation/purging) and the reaction mixture was stirred under H_2 balloon atmosphere at RT for 4 days. After NMR indicated full conversion and the absence of any iodinated byproduct, the catalyst was removed under an argon atmosphere by filtration through a Schlenk-frit containing a plug of Celite® and the filter cake was washed with MeOH (3×30 mL). The filtrate was concentrated and the residue was purified via flash chromatography (450 g SiO_2, cyclohexane/EtOAc/AcOH 6:1:0.01 to 3:1:0.01 (v/v/v)) to furnish compound **27c** as a colorless oil.

$C_{32}H_{59}NO_6Si_2$ [610.00 g/mol].

Yield: 592 mg (0.97 mmol, 45%, 4 steps from **26c**), colorless oil.

R_f = 0.30 (cyclohexane/EtOAc/AcOH = 3:1:0.01 (v/v); staining: $KMnO_4$, stains white with $KMnO_4$).

$[\alpha]^{24}_{D}$ = -8.3 (c = 0.62, $CHCl_3$).

^1H NMR (300 MHz, $CDCl_3$): δ = 7.03 (d, ^3J = 8.2 Hz, 2H, *H*-27, 22), 6.75 (d, ^3J = 8.2 Hz, 2H, *H*-26, 23), 4.60 (d, ^3J = 9.8 Hz, 1H, *H*-6), 3.85-3.67 (m, 1H, *H*-11), 3.49-3.26 (m, 1H, *H*-7), 3.01-2.86 (m, 1H, *H*-20), 2.76-2.55 (m, 2H, *H*-20, 19), 1.98-1.73 (m, 1H, *H*-18), 1.72-1.50 (m, 2H, *H*-18, 8), 1.42 (s, 9H, *H*-3, 2, 1), 0.98-0.72 (m, 24 H, *H*-35, 33, 32, 17, 16, 15, 10, 9), 0.18-0.05 (m, 12H, *H*-31, 30, 13, 12).

^{13}C NMR (75 MHz, $CDCl_3$): δ = 178.9 (*C*-27), 156.2 (*C*-24), 154.3 (*C*-5), 131.5 (*C*-21), 130.1 (*C*-27, 22), 120.2 (*C*-26, 23), 79.4 (*C*-4), 72.1 (*C*-11), 57.9 (*C*-7), 43.8 (*C*-19), 38.0 (*C*-20), 35.2

(*C*-18), 28.6 (*C*-3, 2, 1), 28.1 (*C*-8), 26.0 (*C*-35, 14), 25.9 (*C*-17, 16, 15), 25.8 (*C*-34, 33, 32), 21.0 (*C*-9), 18.1 (*C*-10),-3.4 (*C*-31, 13), -4.3 (*C*-30, 12).

HRMS (MALDI-TOF): *m/z* calcd. for $C_{32}H_{59}NO_6Si_2Na$ $[M+Na]^+$: 632.3779; found: 632.3652.

2.3.41 Methyl ((2*R*,4*R*,5*S*)-5-((*tert*-butoxycarbonyl)amino)-4-((*tert*-butyldimethylsilyl)oxy)-2-(4-((*tert*-butyldimethylsilyl)oxy)benzyl)-6-methylheptanoyl)-L-prolyl-L-tryptophanate (28c)

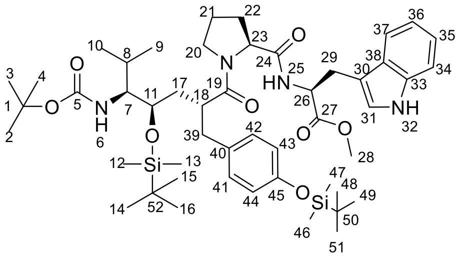

28c

Boc-Deprotection of Boc-Pro-Trp-OMe: In a 50 mL round bottom flask, equipped with magnetic stirring bar, 459 mg (1.10 mmol, 1.2 eq.) Boc-Pro-Trp-OMe peptide were dissolved in 674 µL (9.21 mmol, 10 eq.) ethanethiol. Subsequently, 2.13 mL (27.6 mmol, 30 eq.) trifluoroacetic acid were added and the yellowish reaction mixture was stirred vigorously at RT. After 1 h the volatiles were removed under reduced pressure with a cooling trap. The residue was dissolved in 15 mL EtOAc and 25% aqueous ammonia (10 mL) were added and the reaction mixture was stirred for 10 min. The phases were separated and the aqueous layer was extracted with EtOAc (2x15 mL). The combined organic layers were dried over Na_2SO_4 and the solvent was removed under reduced pressure. The residue was dried *in vacuo* to furnish the deprotected intermediate as a colorless solid.

Coupling: A 50 mL round bottom flask, equipped with a Schlenk adapter and magnetic stirring bar, was dried (evacuated, heated, N_2-purged) and charged with 562 mg (0.92 mmol, 1 eq.) **27c** and dissolved in 5 mL abs. DMF, followed by the addition of 161 µL (0.92 mmol, 1 eq.) Hünig`s base. The flask was cooled to 0 °C in an ice bath and 419 mg (1.10 mmol, 1.2 eq.) HBTU were added to the reaction mixture. The previously deprotected peptide was dissolved in 2.5 abs. DMF and 322 µL (1.84 mmol, 2 eq.) Hünig`s base were added. After 7 min, the solution of the deprotected intermediate was added to the solution of acid **27c** with a syringe. After the addition was complete, the ice bath was removed and the yellow reaction mixture was stirred at RT for

additional 1 h. After that period another 161 µL (0.92 mmol, 1 eq.) Hünig`s base were added to the reaction mixture and stirred for additional 120 min. Subsequently, the reaction mixture was quenched by the addition of brine (15 mL). Additionally, 20 mL EtOAc were added and the mixture was stirred vigorously for 20 min. Afterwards, the mixture was transferred into a separation funnel and the phases were separated. The aqueous layer was extracted with EtOAc (2x30 mL). The combined organic layers were washed with brine (50 mL), dried over Na_2SO_4 and the solvents were removed under reduced pressure. The residue was purified via flash chromatography (375 g SiO_2, dichloromethane/MeOH 100:1 to 30:1 (v/v)) to furnish a pale orange oily residue. The residue was dissolved in 50 mL EtOAc and the organic layer washed with H_2O (5x50 mL) in order to remove remaining DMF and tetramethylurea. The organic layer was dried over Na_2SO_4, the solvent removed under reduced pressure and the residue dried *in vacuo* to yield the desired peptide **28c** as a colorless solid.

$C_{49}H_{78}N_4O_8Si_2$ [907.35 g/mol].

Yield: 590 mg (0.65 mmol, 59%, 2 steps from **27c**), colorless solid.

R_f = 0.28 (dichloromethane/MeOH = 30:1 (v/v); staining: $KMnO_4$).

$[\alpha]_D^{24}$ = -0.7 (c = 1.45, $CHCl_3$).

m.p. = 78-80 °C.

^1H NMR (300 MHz, $CDCl_3$): δ = 8.08 (br s, 1H, *H*-32), 7.56 (d, ^3J = 6.5 Hz, 1H, *H*-37), 7.37 (d, ^3J = 5.3 Hz, 1H, *H*-25), 7.25-7.00 (m, 4H, *H*-36, 35, 34, 31), 6.83 (d, ^3J = 8.1 Hz, 2H, *H*-42, 41), 6.66 (d, ^3J = 8.1 Hz, 2H, *H*-44, 43), 4.84-4.72 (m, 1H, *H*-26), 4.63-4.42 (m, 2H, *H*-23, 6), 3.71-3.63 (m, 4H, *H*-28, 11), 3.54-3.43 (m, 1H, *H*-20), 3.37-3.20 (m, 3H, *H*-29, 7), 2.99-2.87 (m, 1H, *H*-20), 2.71-2.35 (m, 3H, *H*-39, 18), 2.30-2.17 (m, 1H, *H*-22), 1.93-1.54 (m, 6H, *H*-22, 21, 17, 8), 1.40 (s, 9H, *H*-3, 2, 1), 0.99-0.79 (m, 24H, *H*-51, 49, 48, 16, 15, 14, 10, 9), 0.18-0.08 (m, 12H, *H*-47, 46, 13, 12).

^{13}C NMR (75 MHz, $CDCl_3$): δ = 175.1 (*C*-19), 172.5 (*C*-27), 171.2 (*C*-24), 156.0 (*C*-5), 154.3 (*C*-45), 136.2 (*C*-33), 132.0 (*C*-40), 130.0 (*C*-42, 41), 127.8 (*C*-38), 123.5 (*C*-31), 122.2 (*C*-35), 120.2 (*C*-44, 43), 119.6 (*C*-36), 118.7 (*C*-37), 111.3 (*C*-34), 110.3 (*C*-31), 80.0 (*C*-1), 72.4 (*C*-11), 60.1 (*C*-23), 57.6 (*C*-7), 53.5 (*C*-26), 52.4 (*C*-28), 47.3 (*C*-20), 42.2 (*C*-18), 38.4 (*C*-39), 35.4 (*C*-17), 28.5 (*C*-4, 3, 2), 27.7 (*C*-8), 27.6 (*C*-29), 27.4 (*C*-22), 26.0 (*C*-16, 15, 14), 24.9 (*C*-21), 21.5 (*C*-10), 18.4 (*C*-9), 18.3 (*C*-50), 18.1 (*C*-52), -3.4 (*C*-47), -4.1 (*C*-46), -4.3 (*C*-13), -4.8 (*C*-12).

HRMS (MALDI-TOF): *m/z* calcd. for $C_{49}H_{78}N_4O_8Si_2Na$ $[M+Na]^+$: 929.5256; found: 929.3843.

2.3.42 Methyl ((2*R*,4*R*,5*S*)-5-((*S*)-2-((*tert*-butoxycarbonyl)amino)-3-methylbutanamido)-4-hydroxy-2-(4-hydroxybenzyl)-6-methylheptanoyl)-L-prolyl-L-tryptophanate (30c)

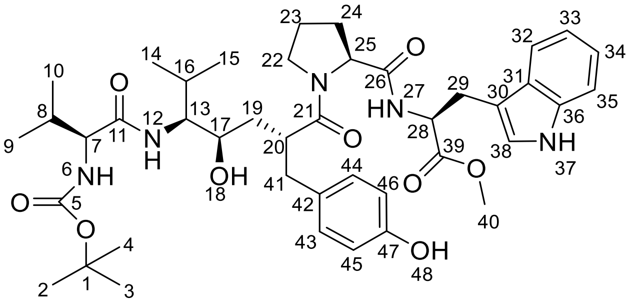

30c

Aryl-TBS-Deprotection: In a 25 mL polypropylene vial, equipped with magnetic stirring bar, 178 mg (0.20 mmol, 1 eq.) **28c** were dissolved in 2 mL MeOH. Afterwards, 61 mg (0.78 mmol, 4 eq.) potassium hydrogen difluoride were added in one portion. The vial was sealed and the reaction mixture was stirred at RT overnight. After 18 h the reaction mixture was concentrated and the residue was purified via flash chromatography (120 g SiO_2, dichloromethane/MeOH = 20:1 (v/v)) to furnish the deprotected aryl alcohol **28c***as a colorless solid (147 mg, 0.19 mmol). The product was used in the next step without further treatment.

Tandem deprotection of Boc- and TBS-moeity: In a 50 mL round bottom flask, equipped with a magnetic stirring bar, 147 mg (0.19 mmol 1 eq.) of the previously deprotected intermediate were dissolved in 2 mL 2,2,2-trifluoroethanol. Subsequently, 55 µL (0.75 mmol, 4 eq.) ethanethiol and 334 mg (1.48 mmol, 8 eq.) zinc dibromide were added in one portion and the mixture was stirred vigorously at RT. After 30 min the formation of a brownish precipitate could be observed. The reaction mixture was stirred for additional 3 h. Afterwards, the reaction

mixture was quenched by the addition of 10 mL 25% aqueous ammonia, followed by the addition of EtOAc (20 mL). The mixture was stirred for 15 min and then transferred into a separation funnel. The phases were separated and the aqueous layer was extracted with EtOAc (2x10 mL). The combined organic layers were dried over Na_2SO_4 and the solvent was removed under reduced pressure and the residue dried *in vacuo* for 30 min to furnish the fully deprotcted intermediate as a colorless solid.

Coupling: A 50 mL round bottom flask, equipped with a Schlenk adapter and magnetic stirring bar, was dried (evacuated, heated, N_2-purged) and charged with 48 mg (0.22 mmol, 1.2 eq.) Boc-Val-OH and dissolved in 0.45 mL abs. DMF, followed by the addition of 33 µL (0.19 mmol, 1 eq.) Hünig`s base. The flask was cooled to 0 °C in an ice bath and 84 mg (0.22 mmol, 1.2 eq.) HATU were added to the reaction mixture. The previously fully deprotected peptide was dissolved in 1 mL abs. DMF and 66 µL (0.38 mmol, 2 eq.) Hünig`s base were added. After 5 min, the solution of the deprotected intermediate was added to the solution of the activated Boc-L-Val-OH with a syringe. After the addition was complete, the ice bath was removed and the yellow reaction mixture was stirred at RT overnight. Subsequently, the reaction mixture was quenched by the addition of brine (5 mL). Additionally, 15 mL of EtOAc were added and the mixture was stirred vigorously for 10 min. Afterwards, the mixture was transferred into a separation funnel and the phases were separated. The aqueous layer was extracted with EtOAc (2x10 mL). The combined organic layers were dried over Na_2SO_4 and the solvents removed under reduced pressure. The residue was purified via flash chromatography (375 g SiO_2, dichloromethane/MeOH = 20:1 (v/v)) to furnish a yellowish oily residue. The residue was dissolved in 50 mL EtOAc and the organic layer washed with H_2O (3x50 mL) in order to remove remaining DMF. The organic layer was dried over Na_2SO_4, the solvent removed under reduced pressure and the residue dried *in vacuo* to yield the desired peptide **30c** as a colorless solid.

$C_{42}H_{59}N_5O_9$ [777.96 g/mol]

Yield: 55 mg (0.071 mmol, 36%, 3 steps from **28c**), colorless solid.

R_f = 0.15 (dichloromethane/MeOH = 20:1 (v/v); staining: CAM).

$[\alpha]_D^{24}$ = -25.6 (c = 1.91, $CHCl_3$).

m.p. = 91-93 °C.

^{1}H NMR (300 MHz, $CDCl_3$, complex mixture of 2 rotamers in 3:1 ratio): δ = 9.07 (bs, 1H, *H*-37), 7.51 (d, ^{3}J = 7.5 Hz, 1H, *H*-32), 7.36-6.81 (m, 7H, *H*-44, 43, 38, 35, 34, 33, 27), 6.67 (d, ^{3}J = 8.1 Hz, 2H, *H*-46, 45), 6.15 (d, ^{3}J = 9.2 Hz, 1H, *H*-12), 5.14-5.03 (m, 1H, *H*-6), 4-72-4.61 (m, 1H, *H*-28), 3.88-3.81 (m, 1H, *H*-7), 3.80-3.59 (m, 5H, *H*-40, 17, 13), 3.50-3.17 (m, 3H, *H*-29, 25), 3.12-3.03 (m, 1H, *H*-22), 2.89-2.74 (m, 1H, *H*-22), 2.63-2.35 (m, 3H, *H*-47, 20), 2.24-1.55 (m, 5H, *H*-24, 19, 16, 8), 1.47-1.39 (m, 9H, *H*-4, 3, 2), 1.37-1.32 (m, 1H, *H*-22), 1.05-0.83 (m, 14H, *H*-24, 23, 15, 14, 10, 9).

^{13}C NMR (75 MHz, $CDCl_3$, complex mixture of 2 rotamers in 3:1 ratio): δ = 176.0 (*C*-21), 173.0 (*C*-39), 172.8 (*C*-26, 11), 156.2 (*C*-5), 155.5 (*C*-47), 136.4 (*C*-36), 130.2 (*C*-44, 43), 130.0 (*C*-42), 127.4 (*C*-31), 123.4 (*C*-38), 122.3 (*C*-34), 119.9 (*C*-33), 118.1 (*C*-32), 115.6 (*C*-46, 45), 111.8 (*C*-35), 109.9 (*C*-30), 80.4 (*C*-1), 71.2 (*C*-17), 61.2 (*C*-7), 60.8 (*C*-25), 59.4 (*C*-13), 53.7 (*C*-28), 52.7 (*C*-40), 46.3 (*C*-22), 44.4 (*C*-20), 39.3 (*C*-41), 36.9 (*C*-19), 31.2 (*C*-24), 29.8 (*C*-8), 28.5 (*C*-4, 3, 2), 28.1 (*C*-16), 26.2 (*C*-29), 21.7 (*C*-23), 20.6 (*C*-14), 19.9 (*C*-10), 18.3 (*C*-9), 16.7 (*C*-15).

HRMS (MALDI-TOF): *m/z* calcd. for $C_{42}H_{59}N_5O_9Na$ $[M+Na]^+$: 800.4210; found: 800.4656.

2.3.43 ((2*R*,4*R*,5*S*)-5-((*S*)-2-Ammonio-3-methylbutanamido)-4-hydroxy-2-(4-hydroxybenzyl)-6-methylheptanoyl)-L-prolyl-L-tryptophanate (31c)

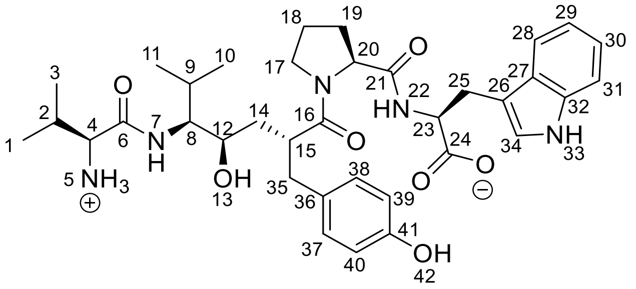

31c

Saponification: In a 25 mL round bottom flask, equipped with magnetic stirring bar, 37.8 mg (0.049 mmol, 1 eq.) **30c** were dissolved in 0.4 mL THF. Afterwards, a solution of 16 mg (0.388 mmol, 8 eq.) LiOHxH_2O in 1.2 mL H_2O was added and the reaction mixture was stirred at RT. After 45 min TLC indicated full conversion, Et_2O (2 mL) was added, followed by the careful addition of glacial acetic acid until a pH of ~4 was achieved. The mixture was transferred into a separation funnel. The phases were separated and the aqueous layer was extracted with Et_2O (2x5 mL). The combined organic extracts were dried over Na_2SO_4 and the solvents removed under reduced pressure. The residue was dried *in vacuo* for 45 min to yield the saponified intermediate as a colorless solid.

Boc-Deprotection: In a 25 mL round bottom flask, equipped with a magnetic stirring bar, the crude intermediate was dissolved in 1 mL 2,2,2-trifluoroethanol, followed by the addition of 14 µL (0.194 mmol, 4 eq.) ethanethiol and 88 mg (0.388 mmol, 8 eq.) zinc dibromide. The reaction mixture turned pink immediately and was stirred vigorously at RT overnight. Afterwards, the reaction mixture was concentrated and the residue dissolved in a mixture of H_2O (700 µL), acetonitrile (290 µL) and DMSO (10 µL) and purified via preparative HPLC (*CLF_NucleodurC18_pc001_HCOOH_5to95*) to furnish compound **31c** as a colorless solid.

$C_{36}H_{49}N_5O_7$ [663.82 g/mol].

Yield: 8.6 mg (0.013 mmol, 27%, 2 steps from **30c**), colorless solid.

$[\alpha]_D^{24}$ = -18.5 (c = 0.59, MeOH).

m.p. = 172-174 °C.

^{1}H NMR (300 MHz, MeOD, complex mixture of 2 rotamers in 4:1 ratio): δ = 8.41 (br s, 1H, *H*-33), 7.58 (d, ^3J = 7.6 Hz, 1H, *H*-28), 7.31 (d, ^3J = 7.9 Hz, 1H, *H*-31), 7.10-6.99 (m, 3H, *H*-34, 30, 29), 6.89 (d, ^3J = 8.3 Hz, 2H, *H*-38, 37), 6.64 (d, ^3J = 8.3 Hz, 2H, *H*-40, 39), 4.63-4.54

(m, 1H, *H*-23), 3.81-3.63 (m, 3H, *H*-12, 8, 4), 3.54 (d, ^3J = 7.2 Hz, 1H, *H*-20), 3.46-3.36 (m, 1H, *H*-25), 3.23-3.04 (m, 2H, *H*-25, 17), 2.96-2.88 (m, 1H, *H*-17), 2.73-2.49 (m, 3H, *H*-35, 15), 2.31-2.09 (m, 2H, *H*-9, 2), 1.99-1.91 (m, 1H, *H*-14), 1.66-1.51 (m, 2H, *H*-19, 14), 1.19-0.60 (m, 15H, *H*-19, 18, 11, 10, 3, 1).

^{13}C NMR (75 MHz, MeOD): δ = 178.1 (*C*-24, 16), 173.4 (*C*-21), 170.2 (*C*-6), 157.3 (*C*-36), 138.0 (*C*-32), 131.0 (*C*-38, 37), 130.8 (*C*-41), 129.0 (*C*-27), 124.3 (*C*-34), 122.4 (*C*-30), 119.8 (*C*-29), 119.4 (*C*-28), 116.3 (*C*-40, 39), 112.2 (*C*-31), 112.0 (*C*-26), 71.2 (*C*-12), 62.2 (*C*-20), 61.3 (*C*-8), 60.2 (*C*-4), 56.5 (*C*-23), 47.4 (*C*-17), 45.7 (*C*-15), 39.9 (*C*-35), 37.6 (*C*-14), 32.0 (*C*-2), 31.6 (*C*-19), 29.3 (*C*-9), 28.3 (*C*-25), 22.0 (*C*-18), 21.0 (*C*-11), 19.6 (*C*-3), 18.0 (*C*-10), 17.4 (*C*-1).

HRMS (MALDI-TOF): *m/z* calcd. for $C_{36}H_{49}N_5O_7Na$ $[M+Na]^+$: 686.3530; found: 686.3739.

2.3.44 Methyl ((2*R*,4*R*,5*S*)-5-((*S*)-2-amino-3-methylbutanamido)-4-hydroxy-2-(4-hydroxybenzyl)-6-methylheptanoyl)-L-prolyl-L-tryptophanate (32)

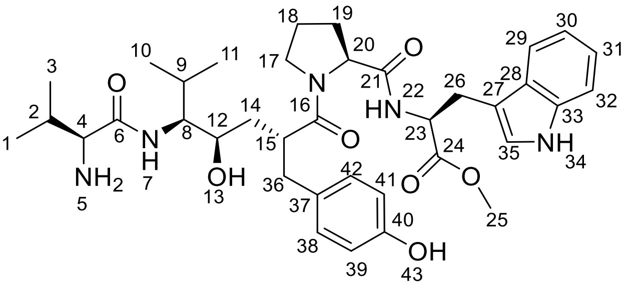

32

In a 5 mL glass vial, equipped with magnetic stirring bar, 15 mg (0.019 mmol, 1 eq.) **30c** were dissolved in 2,2,2-trifluoroethanol, followed by the addition of 5.6 µL (0.154 mmol, 4 eq.) ethanethiol. Afterwards, 35 mg (0.154 mmol, 8 eq.) zinc dibromide were added in one portion and the turbid reaction mixture was stirred vigorously at RT for 24 h. The reaction mixture was concentrated and the residue dissolved in H_2O (300 µL) and acetonitrile (200 µL). The crude product was purified via preparative HPLC (*CLF_NucleodurC18_pc001_HCOOH_5to95*) to yield compound **32** as a colorless solid.

$C_{37}H_{51}N_5O_7$ [677.84 g/mol].

Yield: 4.5 mg (0.006 mmol, 31%), colorless solid.

$[\alpha]_D^{24}$ = -17.8 (c = 0.06, MeOH).

m.p. = 148-150 °C.

^{1}H NMR (500 MHz, MeOD, complex mixture of 2 rotamers in 2:1 ratio): δ = 8.50 (br s, 1H, *H*-34), 7.52 (d, ^3J = 7.8 Hz, 1H, *H*-29), 7.33 (d, ^3J = 8.0 Hz, 1H, *H*-32), 7.10-6.99 (m, 3H, *H*-35, 31, 30), 6.93 (d, ^3J = 8.4 Hz, 2H, *H*-42, 38), 6.66 (d, ^3J = 8.4 Hz, 2H, *H*-41, 39), 4.70-4.63 (m, 1H, *H*-23), 3.87-3.55 (m, 7H, *H*-25, 20, 12, 8 ,4), 3.40-3.33 (m, 1H, *H*-26), 3.20-3.15 (m, 1H, *H*-26), 3.03-2.92 (m, 2H, *H*-17), 2.64-2.48 (m, 3H, *H*-36, 15), 2.27-2.10 (m, 2H, *H*-9, 2), 1.96-1.90 (m, 1H, *H*-14), 1.63-1.55 (m, 2H, *H*-19, 14), 1.34-0.88 (m, 15H, *H*-19, 18, 11, 10, 3, 1).

^{13}C NMR (125 MHz, MeOD): δ = 177.8 (*C*-16), 174.5 (*C*-24), 173.9 (*C*-21), 170.2 (*C*-6), 157.4 (*C*-37), 138.2 (*C*-33), 131.2 (*C*-40), 131.0 (*C*-42, 38), 128.4 (*C*-28), 124.6 (*C*-35), 122.6 (*C*-31), 120.1 (*C*-30), 119.1 (*C*-29), 116.4 (*C*-41, 39), 112.5 (*C*-32), 112.4 (*C*-27), 71.6 (*C*-12), 61.8 (*C*-20), 61.36 (*C*-8), 60.0 (*C*-4), 55.0 (*C*-23), 52.9 (*C*-25), 47.5 (*C*-17), 45.9 (*C*-15), 40.2

(*C*-36), 38.2 (*C*-14), 32.2 (*C*-2), 32.0 (*C*-19), 29.9 (*C*-9), 29.3 (*C*-26), 22.6 (*C*-11), 22.1 (*C*-18), 19.9 (*C*-3), 17.3 (*C*-10), 17.1 (*C*-1).

HRMS (MALDI-TOF): *m/z* calcd. for $C_{37}H_{51}N_5O_7Na$ $[M+Na]^+$: 700.3486; found: 700.3273.

2.3.55 ***tert*-Butyl (*S*)-2-(((*S*)-3-(1*H*-indol-3-yl)-1-methoxy-1-oxopropan-2-yl)carbamoyl)piperidine-1-carboxylate (38)**

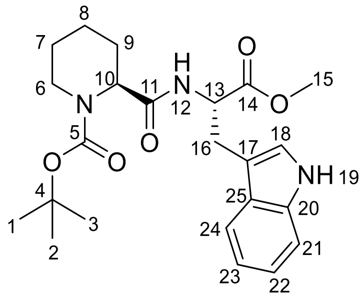

38

A 250 mL three-necked round bottom flask, equipped with gas valve, dropping funnel and magnetic stirring bar, was dried (evacuated, heated, N_2-purged) and charged with 5.00 g (22.0 mmol, 1 eq.) Boc-Pip-OH and 40 mL abs. dichloromethane. The reaction mixture was cooled to 0 °C in an ice bath and 4.05 mL (26.0 mmol, 1.2 eq.) *N,N′*-diisopropylcarbodiimide were added in one portion. Subsequently, 5.55 g (22.0 mmol, 1 eq.) H-Trp-OMe, dissolved in 6.05 mL (44.0 mmol, 2 eq.) triethylamine and 50 mL abs. dichloromethane, were added dropwise via a dropping funnel within 5 min. The reaction mixture was stirred for additional 15 min at 0 °C. The yellowish suspension was allowed to warm up to RT and stirred overnight at this temperature. After 22 h the reaction mixture was concentrated to a small volume and the residue was dissolved in 400 mL EtOAc. The organic layer was washed with 1 M HCl (200 mL), 0.1 M NaOH (200 mL), brine (200 mL), dried over Na_2SO_4 and concentrated. The residue was purified via flash chromatography (600 g SiO_2, cyclohexane/EtOAc = 2:1 (v/v)).

$C_{23}H_{31}N_3O_5$ [429.25 g/mol].

Yield: 4.71 g (11.0 mmol, 50%), colorless solid.

R_f = 0.28 (cyclohexane/EtOAc = 2:1(v/v); staining: CAM).

$[\alpha]_D^{24}$ = -29.5 (c = 1.20, $CHCl_3$).

m.p. = 66-68 °C.

^{1}H NMR (300 MHz, $CDCl_3$): δ = 8.24 (br s, 1H, *H*-19), 7.53 (d, ^3J = 7.6 Hz, 1H, *H*-24), 7.33 (d, ^3J = 8.0 Hz, 1H, *H*-21), 7.23-7.06 (m, 2H, *H*-23, 22), 7.04-6.92 (m, 1H, *H*-18), 6.63-6.37 (m, 1H, *H*-12), 5.00-4.57 (m, 2H, *H*-13, 10), 4.00-3.51 (m, 4H, *H*-15, 6), 3.40-3.21 (m, 2H, *H*-16), 2.60-2.10 (m, 2H, *H*-9, 6), 1.61-1.24 (m, 14H, *H*-9, 8, 7, 3, 2, 1).

^{13}C NMR (75 MHz, $CDCl_3$): δ = 172.4 (*C*-14), 171.0 (*C*-11), 154.8 (*C*-5), 136.3 (*C*-20), 127.5 (*C*-25), 122.9 (*C*-18), 122.4 (*C*-22), 119.8 (*C*-23), 118.7 (*C*-24), 111.3 (*C*-21), 110.0 (*C*-17), 80.7 (*C*-4), 55.5 (*C*-10), 53.0 (*C*-13), 52.5 (*C*-15), 41.0 (*C*-6), 28.3 (*C*-3, 2, 1), 27.9 (*C*-16), 25.6 (*C*-7), 24.9 (*C*-9), 20.5 (*C*-8).

2.3.56 Methyl ((*S*)-1-((2*R*,4*R*,5*S*)-2-benzyl-5-((*tert*-butoxycarbonyl)amino)-4-((*tert*-butyldimethylsilyl)oxy)-6-methylheptanoyl)piperidine-2-carbonyl)-L-tryptophanate (33)

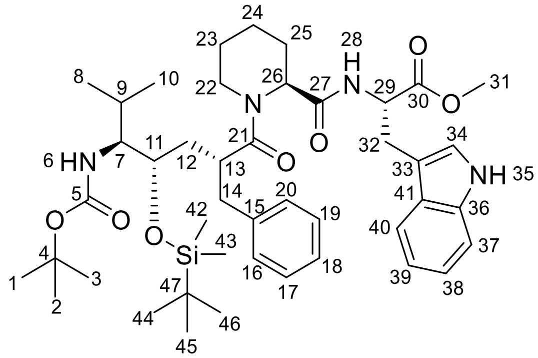

33

Boc-Deprotection of Boc-Pip-Trp-OMe: In a 250 mL round bottom flask, equipped with magnetic stirring bar, 841 mg (1.96 mmol, 1.2 eq.) Boc-Pip-Trp-OMe (**38**) were dissolved in 1.20 mL (16.3 mmol, 10 eq.) ethanethiol. Subsequently, 3.77 mL (49.0 mmol, 30 eq.) trifluoroacetic acid were added and the yellowish reaction mixture was stirred vigorously at RT. After 30 min the volatiles were removed under reduced pressure with a cooling trap. The residue was dissolved in 50 mL EtOAc and 25% aqueous ammonia (70 mL) were added and the reaction mixture was stirred for 20 min. The phases were separated and the aqueous layer was extracted with EtOAc (2x50 mL). The combined organic layers were washed with brine (50 mL), dried over Na_2SO_4 and the solvent was removed under reduced pressure. The residue was dried *in vacuo* to furnish the deprotected intermediate as a colorless solid.

Coupling: A 50 mL round bottom flask, equipped with a Schlenk adapter and magnetic stirring bar, was dried (evacuated, heated, N_2-purged) and charged with 782 mg (1.63 mmol, 1 eq.) pseudopeptide acid **18** and dissolved in 8 mL abs. DMF, followed by the addition of 568 µL (3.26 mmol, 2 eq.) Hünig`s base. The flask was cooled to 0 °C in an ice bath and 744 mg (1.96 mmol, 1.2 eq.) HATU were added to the reaction mixture. The previously deprotected peptide **38** was dissolved in 4 mL abs. DMF and 852 µL (4.90 mmol, 3 eq.) Hünig`s base were added. After 5 min, the solution of the deprotected intermediate was added to the solution of acid **18** with a syringe. After the addition was complete, the ice bath was removed and the yellow reaction mixture was stirred at RT overnight. After 18 h the reaction mixture was quenched by the addition of brine (25 mL). Additionally, 20 mL EtOAc were added and the mixture was stirred vigorously for 5 min. Afterwards, the mixture was transferred into a separation funnel and the phases were separated. The aqueous layer was extracted with EtOAc (2x30 mL). The

combined organic layers were washed with brine (30 mL), dried over Na_2SO_4 and the solvents were removed under reduced pressure. The residue was purified via flash chromatography (400 g SiO_2, dichloromethane/MeOH = 30:1 (v/v)) to furnish an orange oily residue. The residue was dissolved in 50 mL EtOAc and the organic layer washed with H_2O (4x50 mL) in order to remove remaining DMF and tetramethylurea. The organic layer was dried over Na_2SO_4, the solvent removed under reduced pressure and the residue dried *in vacuo* to yield the desired peptide **33** as a colorless solid.

$C_{44}H_{66}N_4O_7Si$ [791.12 g/mol].

Yield: 920 mg (1.16 mmol, 70%, 2 steps from **18**), colorless solid.

R_f = 0.28 (dichloromethane/MeOH = 20:1(v/v); staining: CAM).

$[\alpha]_D^{24}$ = -30.9 (c = 0.91, $CHCl_3$).

m.p. = 82-84 °C.

^{1}H NMR (300 MHz, $CDCl_3$): δ = 8.13 (br s, 1H, *H*-35), 7.51 (d, ^3J = 7.3 Hz, 1H, *H*-40), 7.33-6.84 (m, 9H, *H*-39, 38, 37, 34, 20, 19, 18, 17, 16), 6.20 (d, ^3J = 6.9 Hz, 1H, *H*-28), 5.25-5.10 (m, 1H, *H*-26), 4.82-4.65 (m, 1H, *H*-29), 4.54 (d, ^3J = 10.0 Hz, 1H, *H*-6), 3.80-3.57 (m, 4H, *H*-31, 11), 3.56-3.20 (m, 3H, *H*-32, 22, 7), 3.18-2.91 (m, 2H, *H*-32, 13), 2.87-2.54 (m, 2H, *H*-14), 2.44-2.25 (m, 1H, *H*-22), 2.21-1.98 (m, 1H, *H*-25), 1.93-1.26 (m, 17H, *H*-25, 24, 22, 12, 9, 3, 2, 1), 0.93-0.77 (m, 15H, *H*-46, 45, 44, 10, 8), 0.15-0.02 (m, 6H, *H*-43, 42).

^{13}C NMR (75 MHz, $CDCl_3$): δ = 175.3 (*C*-21), 172.3 (*C*-30), 170.7 (*C*-27), 156.0 (*C*-5), 139.6 (*C*-15), 136.2 (*C*-36), 129.0 (*C*-20, 16), 128.7 (*C*-19, 17), 127.7 (*C*-41), 126.7 (*C*-18), 123.0 (*C*-34), 122.3 (*C*-38), 119.7 (*C*-39), 118.7 (*C*-40), 111.3 (*C*-37), 110.3 (*C*-33), 79.0 (*C*-4), 72.0 (*C*-11), 58.1 (*C*-7), 52.8 (*C*-29), 52.6 (*C*-26), 52.4 (*C*-31), 43.3 (*C*-22), 39.5 (*C*-13), 38.5 (*C*-14), 36.2 (*C*-12), 28.5 (*C*-3, 2, 1), 28.2 (*C*-9), 27.5 (*C*-32), 26.0 (*C*-46, 45, 44), 25.8 (*C*-25), 25.6 (*C*-23), 21.2 (*C*-8), 20.4 (*C*-24), 18.4 (*C*-10), 18.0 (*C*-47), -4.0 (*C*-43), -4.6 (*C*-42).

HRMS (MALDI-TOF): *m/z* calcd. for $C_{44}H_{66}N_4O_7SiNa$ $[M+Na]^+$: 813.4598; found: 813.5087.

2.3.57 Methyl ((*S*)-1-((2*R*,4*R*,5*S*)-2-benzyl-5-((*S*)-2-((*tert*-butoxycarbonyl)amino)-3-methylbutanamido)-4-hydroxy-6-methylheptanoyl)piperidine-2-carbonyl)-L-tryptophanate (36a)

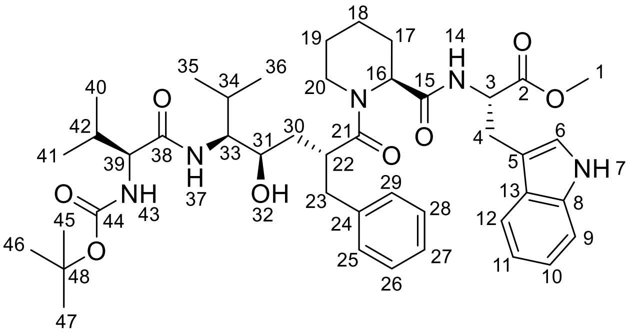

36a

TBS-deprotection of ***33****:* In a 50 mL polypropylene vial, equipped with a magnetic stirring bar, 400 mg (0.51 mmol, 1 eq.) **33** were dissolved in 5 mL THF. Afterwards, 1.30 mL (50.6 mmol, 100 eq.) HF-pyridine (~70% HF) were added in one portion. The reaction mixture turned yellow immediately and was stirred for 90 min, until TLC indicated full conversion. The reaction mixture was quenched by pouring it into 100 mL ice cold satd. $NaHCO_3$ solution. Subsequently, 50 mL EtOAc were added and the mixture transferred into a separation funnel. The phases were separated and the aqueous layer was extracted with EtOAc (2x50 mL). The combined organic extracts were washed with brine (100 mL), dried over Na_2SO_4 and the solvents removed under reduced pressure. The colorless residue was dried *in vacuo* for 15 min and used in the next step without further purification.

Boc-deprotection: In a 50 mL round bottom flask, equipped with magnetic stirring bar, the previously TBS-deprotected peptide was dissolved in 373 µL (5.05 mmol, 10 eq.) ethanethiol. Subsequently, 1.17 mL (15.1 mmol, 30 eq.) trifluoroacetic acid were added and the yellowish reaction mixture was stirred vigorously for 3 min at RT. The volatiles were removed under reduced pressure with a cooling trap. The residue was dissolved in 50 mL EtOAc and 25% aqueous ammonia (50 mL) were added and stirred for 5 min. The phases were separated and the aqueous layer was extracted with EtOAc (2x30 mL). The combined organic layers were washed with brine (50 mL), dried over Na_2SO_4 and the solvent was removed under reduced pressure.

Coupling: A 50 mL round bottom flask, equipped with a magnetic stirring bar and Schlenk adapter, was dried (evacuated, heated, N_2-purged) and charged with 132 mg (0.61 mmol, 1.2 eq.) Boc-L-Val-OH and 1.3 mL abs. DMF, followed by the addition of 176 µL (0.10 mmol, 2 eq.) Hünig's base. The flask was cooled to 0 °C in an ice bath. Subsequently, 231 mg (0.61 mmol, 1.2 eq.) HATU were added to the reaction mixture and stirred for additional 5 min. In a second 50 mL round bottom flask, equipped with a magnetic stirring bar and Schlenk adapter, the previously deprotected peptide was dissolved in 2.6 mL abs. DMF and 264 µL (1.52 mmol, 3 eq.) Hünig's base were added. Afterwards, the solution of the deprotected peptide was added to the solution of the activated acid using a syringe. After the addition was complete, the ice bath was removed and the deep yellow reaction mixture was stirred at RT overnight. The reaction was quenched by the addition of 20 mL brine, followed by the addition of EtOAc (25 mL). The reaction mixture was stirred for 5 min and subsequently transferred into a separation funnel. The phases were separated and the aqueous layer was extracted with EtOAc (2x20 mL). The combined organic extracts were washed with brine (50 mL), dried over Na_2SO_4 and the solvents removed under reduced pressure. The residue was purified via flash chromatography (450 g SiO_2, dichloromethane/MeOH = 30:1 (v/v)) to furnish an oily residue. The residue was dissolved in 50 mL EtOAc and the organic layer washed with H_2O (4x40 mL) The organic layer was dried over Na_2SO_4, the solvent removed under reduced pressure and the residue dried *in vacuo* to yield the desired product **36a** as a colorless solid.

$C_{43}H_{61}N_5O_8$ [775.99 g/mol].

Yield: 122 mg (0.16 mmol, 31%, 3 steps from **33**), colorless solid.

R_f = 0.15 (dichloromethane/MeOH = 20:1(v/v); staining: CAM).

$[\alpha]^{24}_{D} =$ -36.8 (c = 1.36, $CHCl_3$).

m.p. = 100-102 °C.

^1H NMR (300 MHz, $CDCl_3$): δ = 8.76 (br s, 1H, *H*-7), 7.60-7.42 (m, 2H, *H*-14, 12), 7.38-7.30 (m, 1H, *H*-9), 7.25-6.93 (m, 8H, *H*-29, 28, 27, 26, 25, 11, 10, 6), 6.08 (d, ^3J = 8.6 Hz, 1H, *H*-37), 5.16-4.99 (m, 1H, *H*-43), 4.85-4.71 (m, 1H, *H*-3), 4.25 (d, ^3J = 13.2 Hz, 1H, *H*-20), 4.09 (br s, 1H, *H*-16), 3.91-3.62 (m, 5H, *H*-39, 33, 1), 3.53-3.18 (m, 3H, *H*-31, 4), 2.84-2.57 (m, 3H, *H*-23, 22), 2.30-1.79 (m, 5H, *H*-42, 34, 30, 18, 17), 1.72-1.52 (m, 2H, *H*-30, 20), 1.47-1.40 (m, 9H, *H*-47, 46, 45), 1.29-0.78 (m, 15H, *H*-41, 40, 36, 35, 19, 18), -0.06-(-0.28) (m, 1H, *H*-17).

^{13}C NMR (75 MHz, $CDCl_3$): δ = 175.3 (*C*-21), 173.2 (*C*-2), 172.8 (*C*-38), 170.6 (*C*-15), 156.2 (*C*-44), 138.8 (*C*-24), 136.4 (*C*-8), 129.4 (*C*-28, 26), 128.8 (*C*-29, 25), 127.4 (*C*-13), 126.8 (*C*-27), 123.4 (*C*-6), 122.2 (*C*-10), 119.7 (*C*-11), 118.6 (*C*-12), 111.5 (*C*-9), 110.5 (*C*-5), 80.2 (*C*-48), 72.1 (*C*-31), 61.1 (*C*-39), 59.3 (*C*-33), 57.3 (*C*-16), 53.4 (*C*-3), 52.5 (*C*-1), 41.3 (*C*-22), 40.7 (*C*-23), 39.6 (*C*-20), 37.6 (*C*-30), 29.9 (*C*-42), 28.5 (*C*-47, 46, 45), 28.0 (*C*-34), 26.6 (*C*-4), 25.0 (*C*-19), 24.5 (*C*-17), 20.6 (*C*-35), 20.4 (*C*-18), 19.9 (*C*-40), 18.2 (*C*-41), 17.1 (*C*-36).

HRMS (MALDI-TOF): *m/z* calcd. for $C_{43}H_{61}N_5O_8Na$ $[M+Na]^+$: 798.9629; found: 798.3790.

2.3.58 ((*S*)-1-((2*R*,4*R*,5*S*)-5-((*S*)-2-Ammonio-3-methylbutanamido)-2-benzyl-4-hydroxy-6-methylheptanoyl)piperidine-2-carbonyl)-L-tryptophanate (37a)

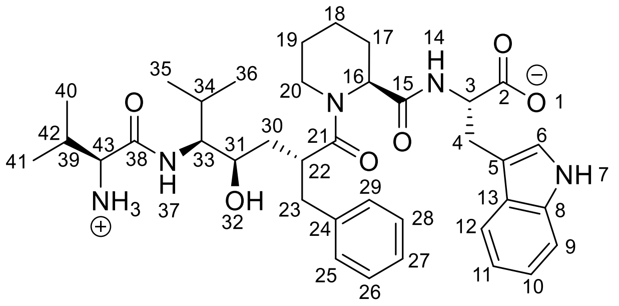

37a

Saponification: In a 50 mL round bottom flask, equipped with magnetic stirring bar, 94 mg (0.121 mmol, 1 eq.) **36a** were dissolved in 1.2 mL THF. Afterwards, a solution of 41 mg (0.969 mmol, 8 eq.) LiOHxH_2O in 3.3 mL H_2O was added and the reaction mixture was stirred at RT. After 3 h TLC indicated full conversion. Then, 1 M HCl were carefully added, until a pH of ~4 was achieved. Additional 15 mL EtOAc were added and the mixture was transferred into a separation funnel. The phases were separated and the aqueous layer was extracted with EtOAc (3x10 mL). The combined organic extracts were dried over Na_2SO_4 and the solvents removed under reduced pressure. The residue was dried *in vacuo* for 2 h to yield the saponified intermediate as a colorless solid.

Boc-Deprotection: In a 10 mL round bottom flask, equipped with magnetic stirring bar, the crude intermediate was dissolved in 90 µL (1.21 mmol, 10 eq.) ethanethiol. Subsequently, 280 µL (3.63 mmol, 30 eq.) trifluoroacetic acid were added and the reaction mixture was stirred vigorously for 5 min. After this period the volatiles were removed with a preceding cooling trap and the residue was dried *in vacuo* for 1 h. The residue was dissolved in in mixture of acetonitrile (650 µL) and H_2O (350 µL) and purified via preparative HPLC (*CLF_NucleodurC18_pc001_HCOOH_5to95*) to yield compound **37a** as a colorless solid.

$C_{37}H_{51}N_5O_6$ [661.84 g/mol].

Yield: 25.2 mg (0.038 mmol, 31%, 2 steps from **37a**), colorless solid.

$[\alpha]_D^{24}$ = -41.2 (c = 0.84, MeOH).

m.p. = 160-162 °C.

^{1}H NMR (300 MHz, MeOD): δ = 8.38 (br s, 1H, H-7), 7.57 (d, 1H, ^3J = 7.7 Hz, *H*-12), 7.33-6.93 (m, 9H, *H*-29, 28, 27, 26, 25, 11, 10, 9, 6), 4.80-4.66 (m, 1H, *H*-3), 4.37-4.25 (m, 1H, *H*-

16), 4.03-3.88 (m, 1H, *H*-20), 3.87-3.65 (m, 3H, *H*-43, 33, 31), 3.50-3.06 (m, 3H, *H*-22, 4), 2.74-2.56 (m, 2H, *H*-23), 2.32-1.94 (m, 3H, *H*-42, 34, 30), 1.79-1.57 (m, 2H, *H*-30, 17), 1.48-0.57 (m, 17H, *H*-41, 40, 36, 35, 20, 19, 18), -0.06-(0.27) (m, 1H, *H*-17).

^{13}C NMR (75 MHz, $CDCl_3$): δ = 178.1 (*C*-21), 177.5 (*C*-2), 171.2 (*C*-15), 170.4 (*C*-38), 140.6 (*C*-24), 138.0 (*C*-8), 130.4 (*C*-28, 26), 129.7 (*C*-29, 25), 128.9 (*C*-13), 127.6 (*C*-27), 124.4 (*C*-6), 122.3 (*C*-10), 119.7 (*C*-11), 119.6 (*C*-12), 112.1 (*C*-9), 112.0 (*C*-5), 71.7 (*C*-31), 61.4 (*C*-43), 60.1 (*C*-33), 58.6 (*C*-16), 56.0 (*C*-3), 42.4 (*C*-22), 40.9 (*C*-23), 40.5 (*C*-20), 38.0 (*C*-30), 31.6 (*C*-39), 29.4 (*C*-34), 28.6 (*C*-4), 26.4 (*C*-19), 25.4 (*C*-17), 21.3 (*C*-18), 20.9 (*C*-35), 19.5 (*C*-41), 18.4 (*C*-40), 17.3 (*C*-36).

HRMS (MALDI-TOF): *m/z* calcd. for $C_{37}H_{51}N_5O_6Na$ $[M+Na]^+$: 684.3737; found: 684.3716.

2.3.59 Methyl ((*S*)-1-((2*R*,4*R*,5*S*)-2-benzyl-5-((*S*)-2-((*tert*-butoxycarbonyl)amino)-3,3-dimethylbutanamido)-4-hydroxy-6-methylheptanoyl)piperidine-2-carbonyl)-L-tryptophanate (36b)

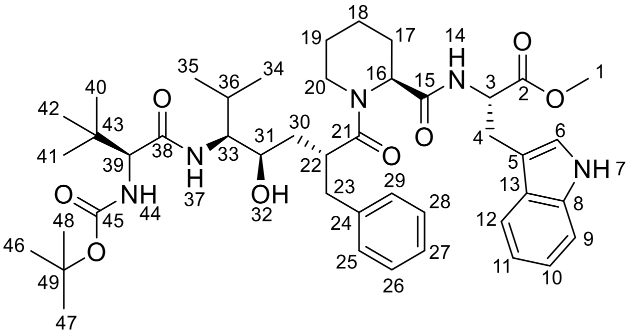

36b

TBS-deprotection of ***33****:* In a 50 mL polypropylene vial, equipped with a magnetic stirring bar, 467 mg (0.59 mmol, 1 eq.) **33** were dissolved in 5 mL THF. Afterwards, 1.30 mL (59.0 mmol, 100 eq.) HF-pyridine (~70% HF) were added in one portion. The reaction mixture turned yellow immediately and was stirred for 100 min, until TLC indicated full conversion. The reaction mixture was quenched by pouring it into 100 mL of an ice cold satd. $NaHCO_3$ solution. Subsequently, 50 mL EtOAc were added and the mixture transferred into a separation funnel. The phases were separated and the aqueous layer was extracted with EtOAc (2x50 mL). The combined organic extracts were washed with brine (100 mL), dried over Na_2SO_4 and the solvents removed under reduced pressure. The colorless residue was dried *in vacuo* for 30 min and used in the next step without further purification.

Boc-deprotection: In a 50 mL round bottom flask, equipped with magnetic stirring bar, the previously TBS-deprotected peptide was dissolved in 440 µL (5.90 mmol, 10 eq.) ethanethiol. Subsequently, 1.37 mL (17.7 mmol, 30 eq.) trifluoroacetic acid were added and the yellowish reaction mixture was stirred vigorously for 2 min at RT. The volatiles were removed under reduced pressure with a cooling trap. The residue was dissolved in 50 mL EtOAc and 25% aqueous ammonia (50 mL) were added and the reaction mixture was stirred for 5 min. The phases were separated and the aqueous layer was extracted with EtOAc (2x50 mL). The combined organic layers were washed with brine (80 mL), dried over Na_2SO_4 and the solvent was removed under reduced pressure.

Coupling: A 50 mL round bottom flask, equipped with magnetic stirring bar and Schlenk adapter, was dried (evacuated, heated, N_2-purged) and charged with 164 mg (0.71 mmol, 1.2 eq.) Boc-L-*tert*-leucine and 1.4 mL abs. DMF, followed by the addition of 205 µL (1.18 mmol,

2 eq.) Hünig's base. The flask was cooled to 0 °C in an ice bath. Subsequently, 270 mg (0.71 mmol, 1.2 eq.) HATU were added to the reaction mixture and stirred for additional 5 min. In a second 50 mL round bottom flask, equipped with magnetic stirring bar and Schlenk adapter, the previously deprotected peptide was dissolved in 2.8 mL abs. DMF and 309 µL (1.77 mmol, 3 eq.) Hünig's base were added. Afterwards, the solution of the deprotected peptide was added to the solution of the activated acid using a syringe. After the addition was complete, the ice bath was removed and the yellow reaction mixture was stirred at RT overnight. The reaction was quenched by the addition of 20 mL brine, followed by the addition of EtOAc (25 mL). The reaction mixture was stirred for 5 min and subsequently transferred into a separation funnel. The phases were separated and the aqueous layer was extracted with EtOAc (2x30 mL). The combined organic extracts were washed with brine (50 mL), dried over Na_2SO_4 and the solvents removed under reduced pressure. The residue was purified via flash chromatography (500 g SiO_2, dichloromethane/MeOH = 30:1 (v/v)) to furnish an oily residue. The residue was dissolved in 50 mL EtOAc and the organic layer washed with H_2O (4x60 mL) The organic layer was dried over Na_2SO_4, the solvent removed under reduced pressure and the residue dried *in vacuo* to yield the desired product **36b** as a colorless solid.

$C_{44}H_{63}N_5O_8$ [790.02 g/mol].

Yield: 195 mg (0.25 mmol, 42%, 3 steps from **33**), colorless solid.

R_f = 0.21 (dichloromethane/MeOH = 20:1(v/v); staining: CAM).

$[\alpha]^{24}_{D}$ = -39.3 (c = 0.99, $CHCl_3$).

m.p. = 106-108 °C.

^{1}H NMR (300 MHz, $CDCl_3$): δ = 8.73 (br s, 1H, *H*-7), 7.60-7.42 (m, 2H, *H*-14, 12), 7.38-7.30 (m, 1H, *H*-9), 7.25-6.93 (m, 8H, *H*-29, 28, 27, 26, 25, 11, 10, 6), 5.77 (d, ^3J = 9.1 Hz, 1H, *H*-37), 5.18 (d, ^3J = 9.4 Hz, 1H, *H*-44), 4.89-4.75 (m, 1H, *H*-3), 4.24 (d, ^3J = 12.9 Hz, 1H, *H*-20), 4.11 (br s, 1H, *H*-16), 3.88-3.61 (m, 5H, *H*-39, 33, 1), 3.54-3.16 (m, 3H, *H*-31, 4), 2.89-2.58 (m, 3H, *H*-23, 22), 2.27-1.78 (m, 4H, *H*-36, 30, 18, 17), 1.72-1.54 (m, 2H, *H*-30, 20), 1.50-1.35 (m, 9H, *H*-48, 46, 47), 1.33-0.77 (m, 18H, *H*-42, 41, 40, 35, 34, 19, 18), -0.05-(-0.22) (m, 1H, *H*-17).

^{13}C NMR (75 MHz, $CDCl_3$): δ = 175.2 (*C*-21), 173.3 (*C*-2), 172.4 (*C*-38), 170.5 (*C*-15), 156.2 (*C*-45), 138.8 (*C*-24), 136.4 (*C*-8), 129.4 (*C*-28, 26), 128.8 (*C*-29, 25), 127.5 (1*C*-13), 126.8 (*C*-27), 123.2 (*C*-6), 122.3 (*C*-10), 119.8 (*C*-11), 118.6 (*C*-12), 111.5 (*C*-9), 110.4 (*C*-5), 80.2 (*C*-

49), 72.0 (*C*-31), 63.0 (*C*-39), 59.4 (*C*-33), 57.3 (*C*-16), 53.2 (*C*-3), 52.5 (*C*-1), 41.3 (*C*-22), 40.5 (*C*-23), 39.6 (*C*-20), 37.4 (*C*-30), 33.9 (*C*-43), 28.5 (*C*-48, 47, 46), 28.2 (*C*-36), 26.8 (*C*-42, 41, 40), 26.7 (*C*-4), 25.0 (*C*-19), 24.5 (*C*-17), 20.7 (*C*-35), 20.4 (*C*-18), 17.1 (*C*-34).

HRMS (MALDI-TOF): *m/z* calcd. for $C_{44}H_{63}N_5O_8NaH$ $[M+Na]^+$: 812.4574; found: 813.3661.

2.3.60 ((*S*)-1-((2*R*,4*R*,5*S*)-5-((*S*)-2-Ammonio-3,3-dimethylbutanamido)-2-benzyl-4-hydroxy-6-methylheptanoyl)piperidine-2-carbonyl)-L-tryptophanate (37b)

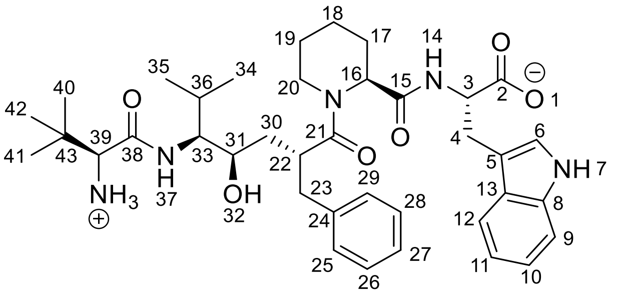

37b

Saponification: In a 50 mL round bottom flask, equipped with a magnetic stirring bar, 146 mg (0.185 mmol, 1 eq.) **33** were dissolved in 1.8 mL THF. Afterwards, a solution of 62 mg (1.48 mmol, 8 eq.) LiOHxH_2O in 5.4 mL H_2O was added and the reaction mixture was stirred at RT. After 3 h TLC indicated full conversion. Then, 1 M HCl were carefully added, until a pH of ~4 was achieved. Additional 15 mL EtOAc were added and the mixture was transferred into a separation funnel. The phases were separated and the aqueous layer was extracted with EtOAc (3x15 mL). The combined organic extracts were dried over Na_2SO_4 and the solvents removed under reduced pressure. The residue was dried *in vacuo* for 2 h to yield the saponified intermediate as a colorless solid.

Boc-Deprotection: In a 10 mL round bottom flask, equipped with a magnetic stirring bar, the crude intermediate was dissolved in 136 µL (1.85 mmol, 10 eq.) ethanethiol. Subsequently, 427 µL (5.55 mmol, 30 eq.) trifluoroacetic acid were added and the reaction mixture was stirred vigorously for 5 min. After this period the volatiles were removed with a preceding cooling trap and the residue was dried *in vacuo* for 1 h. The residue was dissolved in a mixture of acetonitrile (650 µL), H_2O (330 µL) and DMSO (20 µL) and purified via preparative HPLC (*CLF_NucleodurC18_pc001_HCOOH_5to95*) to yield compound **37b** as a colorless solid.

$C_{38}H_{53}N_5O_6$ [675.87 g/mol].

Yield: 30.5 mg (0.045 mmol, 25%, 2 steps from **36b**), colorless solid.

$[\alpha]_D^{24}$ = -32.3 (c = 1.02, MeOH).

m.p. = 164-166 °C.

^{1}H NMR (300 MHz, MeOD): δ = 8.40 (br s, 1H, H-7), 7.61-7.47 (m, 1H, *H*-12), 7.34-6.85 (m, 9H, *H*-29, 28, 27, 26, 25, 11, 10, 9, 6), 4.78-4.64 (m, 1H, *H*-3), 4.36-4.22 (m, 1H, *H*-16), 4.04-3.55 (m, 4H, *H*-43, 33, 31, 20), 3.50-3.06 (m, 3H, *H*-22, 4), 2.80-2.66 (m, 2H, *H*-23), 2.10-1.93

(m, 2H, *H*-36, 30), 1.80-1.56 (m, 2H, *H*-30, 17), 1.47-0.51 (m, 20H, *H*-42, 41, 40, 34, 35, 20, 19, 18), -0.04-(0.26) (m, 1H, *H*-17).

^{13}C NMR (75 MHz, $CDCl_3$): δ = 178.4 (*C*-21), 177.5 (*C*-2), 171.0 (*C*-15), 169.4 (*C*-38), 140.7 (*C*-24), 138.0 (*C*-8), 130.4 (*C*-28, 26), 129.6 (*C*-29, 25), 129.0 (*C*-13), 127.5 (*C*-27), 124.4 (*C*-6), 122.3 (*C*-10), 119.7 (*C*-12, 11), 112.1 (*C*-9), 112.0 (*C*-5), 71.4 (*C*-31), 63.0 (*C*-39), 61.8 (*C*-33), 58.6 (*C*-16), 56.2 (*C*-3), 42.4 (*C*-22), 40.5 (*C*-23, 20), 38.9 (*C*-30), 34.4 (*C*-43), 29.7 (*C*-36), 28.7 (*C*-4), 27.0 (*C*-42, 41, 40), 26.4 (*C*-19), 25.4 (*C*-17), 21.3 (*C*-18), 20.8 (*C*-35), 19.2 (*C*-34).

HRMS (MALDI-TOF): *m/z* calcd. for $C_{38}H_{53}N_5O_6Na$ $[M+Na]^+$: 698.8471; found: 698.3853.

2.4 Biological assays

2.4.1 Protein expression and purification

The active wildtype enzyme used in activity assays and the inactive E451A variant of hDPP3(1–726) used for ITC and crystallization experiments were produced and purified as described previously in (Kumar, P. et al. Sci. Rep. 6, 23787). In brief, the plasmid containing the desired mutations was transformed into a BL21-CodonPlus (DE3) RIL strain. The cell culture was grown in Luria-Bertani (LB) medium containing 50 μg ml−1 kanamycin. Gene expression was induced with 0.4 mM isopropyl-1-thio-D-galactopyranoside (IPTG) after the culture medium reached an OD of 0.6–0.8. After being allowed to grow overnight at 18 °C, the cells were harvested by centrifugation at 4000 g for 10 min. The harvested cell pellet was resuspended in 50 mM Tris-HCl pH 8.0 contaning 300 mM NaCl, 10 mM imidazole and 0.05% 2-mercaptoethanol (lysis buffer) and lysed by sonication. Centrifugation at 18000 g for 1 hour at 4 °C was performed to remove cell debris and the supernatant was subjected to affinity chromatography on Ni-NTA resin (5 mL prepacked His-trap FF, GE Healthcare) previously equilibrated with lysis buffer. After washing, bound protein was eluted using lysis buffer containing 500 mM imidazole. Anion exchange chromatography was performed to improve the purity of the sample using a prepacked MonoQ 5/50 column (GE Healthcare). The column was equilibrated with 20 mM Tris-HCl pH-8.0, 0.05% of 2-mercaptoethanol and the bound protein was eluted using a gradient of the same buffer containing 1 M NaCl. The pure fractions were pooled and incubated with TEV protease overnight at 4 °C to cleave off the His6-tag. After TEV cleavage the protein was again passed through a Ni-NTA column and the flow through containing cleaved protein was collected and pooled. This sample was then applied to a Superdex 200 16/60 gel filtration column (GE Healthcare) and pure fractions corresponding to a molecular mass of ~82 kDa were collected and concentrated. The buffer used for gel filtration was 100 mM multi component buffer (L-malic acid, MES and Tris, pH 8.0) containing 100 mM NaCl and 1 mM tris(2-carboxyethyl)phosphine (TCEP). The purity of the fractions was analyzed by 12.5% SDS-PAGE.

2.4.2 Enzyme inhibition assay

The enzyme activity of hDPP3 was determined by following the release of 2-naphthylamine in a plate reader (excitation at 332 and emission at 420 nm) at 37 °C in a mixture containing 25 µl of 200 µM Arg-Arg-2-naphthylamide as substrate in 50 mM Tris-HCl buffer, pH 8.0 and 0.05-0.1 µM of enzyme in a total reaction mixture of 235 µl (White, Tissue Culture treated Krystal 2000 96-well plate from Porvair sciences, Norfolk, UK). The activity assay was performed by continuous measurement of fluorescence of 2-naphthylamide for 30 min (Molecular Devices, Sunnyvale CA, USA). For the inhibition assay, the inhibitors were added to the mixture without

the substrate and incubated for 10 min at room temperature. The reaction was started by the addition of the substrate. The concentration of an inhibitor that gave 50% inhibition (IC50) was determined through a series of assays with a fixed substrate concentration but with various inhibitor concentrations. 5% DMSO was used in the control assay. The activity (in %) in the presence of increasing concentrations of the inhibitor was calculated using the equation:

% activity = 100 X (Δfluorescence/ Δfluorescence of control)

The activity (in %) against concentration of inhibitor (log scale for inhibitor concentration [x-axis] and linear scale for percent activity [y-axis]) was plotted. The activity in % vs. log of concentration was fitted to a sigmoidal dose-response curve using the four parameter logistic equation entitled "log (inhibitor) vs. response -- variable slope" in GraphPad Prism (San Diego, CA, USA).

All animal experiments were approved by the Austrian Federal Ministry for Science, Research, and Economy (protocol number BMWF-66.007/7-ll/3b/), the ethics committee of the University of Graz, and conducted in compliance with the Council of Europe Convention (ETS 123).

To measure ex vivo inhibition, dppIII-knockout mice (DPP3-/-) bred and maintained in the animal facility of the University of Graz were used. The brains from ~ 16-week old male DPP3-/- mice were surgically removed and washed with PBS. Homogenization was performed on ice in solution A (0.25M sucrose, 1 mM EDTA, 20 μM dithiothreitol, 0.1% Triton X-100, 20 μg/ml leupeptin, 2 μg/ml antipain, 1 μg/ml pepstatin, pH 7.0) using an Ultra Turrax (IKA, Staufen, Germany). 20000 g infranatants were used for further experiments. Protein concentrations in the tissue lysates were estimated using the Protein Assay Dye Reagent (Bio-Rad, Munich, Germany) using bovine serum albumin as the standard. 0.01-0.05 mg/ml of tissue homogenate was used in the same assay as described above.

2.4.3 Isothermal microcalorimetry

The inactive variant E451A of hDPP3 was used for thermodynamic analysis. The titrations were performed in 50 mM Tris-HCl pH 8 containing 100 mM NaCl and 5% DMSO. Both the purified enzyme and ligands were dissolved in exactly the same buffer, and all solutions were degassed immediately before the measurements. The measurements were performed with a VP-ITC microcalorimeter (MicroCal, Northampton, MA, USA). In each experiment the temperature was equilibrated at 298 K. The ligand solution in the syringe (500 μM) was titrated into a 20 μM solution of hDPP3 in the sample cell. In a typical experiment, under constant stirring at 270 rpm, a total of one aliquot of 2 μl and 29 aliquots of 10 μl of the ligand solution

were injected into 1.421 ml of the enzyme solution at a rate of 0.5 µl/s. Every injection was carried out over a period of 20 s with a spacing of 300 s between the injections. The heat of binding was determined by integration of the observed peaks. The observed enthalpies were plotted against the ratio of peptide vs. protein concentration in the cell to generate the binding isotherm. Nonlinear least-squares fitting using Origin® version 7.0 (MicroCal®) was used to obtain association constants (Ka), the enthalpy (ΔH) and stoichiometries. The dissociation constants (Kd) were calculated using Kd = 1/Ka.

2.4.4 Time-dependent inhibition of hDPP3 by tynorphin and HER

Tynorphin (VVYPW), which is an endogenous pentapeptide inhibitor of hDPP3, was shown to be unstable in human serum and was cleaved rapidly to smaller fragments. Being a peptidase, it is likely that hDPP3 degrades tynorphin over time. A comparative study of tynorphin and R-hydroxyethylene was performed to study their behavior in a time-based manner and to assiess their efficiency to inhibit hDPP3 as a function of time. For the assay, 0.1 µM of the active enzyme was incubated at room temperature with inhibitor concentrations equivalent to 5 times their IC50 values. Enzyme in the absence of any inhibitor incubated at room temperature was used as control. At various time points (0, 0.25, 0.50, 0,15, 1, 2, 3, 4, 5, 20 and 24 hours), 10 µl aliquots were added to 200 µl of 50 mM Tris-HCl buffer, pH 8.0. The reaction was initiated by adding 25 µl of 200 µM Arg-Arg-2-naphthylamide and enzyme activity was measured fluorometrically (excitation, 332 nm; emission, 420 nm). The efficacy of inhibition was calculated as percent of control at each time point for tynorphin and **HER**.

2.4.5 Thermal shift assay with HER

Thermal shift assay was performed as described previously by (Ericsson, U. et al. Analytical Biochemistry 357 289–298). For this, a mixture of 10 µM active hDPP3 and 50 µM **HER** was used. A separate mixture with the same concentrations of protein and inhibitor was incubated for 24 hours. A 20 µl aliquot from the pre-incubation solutions were mixed with 2 µl of 1:500 dilution of SYPRO® orange dye (Molecular Probes, Oregon, USA) and added to a white 96-well RT-PCR plate (Bio-Rad, California, USA). Protein incubated in the absence of inhibitor was used as a control sample. The plate was sealed with an Optical-Quality Sealing Tape (Bio-Rad, California, USA). The experiment was started by heating the plate from 20 °C to 95 °C in increments of 0.5 °C/s in a CFX Connect™ Real Time PCR detection system (Bio-Rad, California, USA). Fluorescence changes in the wells of the plate were monitored simultaneously at excitation and emission wavelengths of 470 and 500 nm, respectively.

Melting temperatures (Tm) were determined using CFX manager 3.0 software (Bio-Rad, California, USA).

3 References

[1] W. G. Kofron, L. M. Baclawski, *J. Org. Chem.* **1976**, *41*, 1879–1880. DOI: 10.1021/jo00872a047

[2] H. A. Staab, K. Wendel, *Org. Synth.* **1968**, *48*, 44. DOI: 10.15227/orgsyn.048.0044

[3] Bruker: APEX2 and SAINT. Bruker AXS Inc.: Madison, Wisconsin, USA, 2012.

[4] R. H. Blessing, *Acta Crystallogr., Sect. A* **1995**, *51*, 33–38. DOI: 10.1107/S0108767394005726

[5] G. M. Sheldrick, *Acta Crystallogr., Sect. A* **2008**, *64*, 112–122. DOI: 10.1107/S0108767307043930

[6] A. L. Spek, *J. Appl. Crystallogr.* **2003**, *36*, 7–13. DOI: 10.1107/S0021889802022112

[7] W. Kabsch, *Acta Cryst. D* **2010**, *66*, 125–132. DOI: 10.1107/S0907444909047337

[8] A. J. McCoy, R. W. Grosse-Kunstleve, P. D. Adams, M. D. Winn, L. C. Storoni, R. J. Read, *J. Appl. Cryst*. **2007**, *40*, 658–674. DOI: 10.1107/S0021889807021206

[9] P. D. Adams, et al. *Acta Cryst. D* **2010**, *66*, 213–221. DOI: 10.1107/S0907444909052925

[10] P. Emsley, K. Cowtan, *Acta Cryst. D* **2004**, *60*, 2126–2132. DOI: 10.1107/S0907444904019158

[11] B. E. Haug, D. H. Rich, *Org. Lett.* **2004**, *6*, 4783–4786. DOI: 10.1021/ol047879y

[12] M. J. Burk, J. G. Allen, *J. Org. Chem.* **1997**, *62*, 7054–7057. DOI: 10.1021/jo970903j

[13] R. Déziel, R. Plante, V. Caron, L. Grenier, M. Llinas-Brunet, J.-S. Duceppe, E. Malenfant, N. Moss, *J. Org. Chem.* **1996**, *61*, 2901–2903. DOI: 10.1021/jo951988w

[14] P. F. Schuda, C. B. Ebner, S. J. Potlock, *Synthesis* **1987**, *1987*, 1055–1057. DOI: 10.1055/s-1987-28168

4 Appendix Spectra

^{1}H and ^{13}C NMR spectra of *tert*-Butyl *N*-[(2*S*)-3-methyl-1-oxobutan-2-yl]carbamate (2)

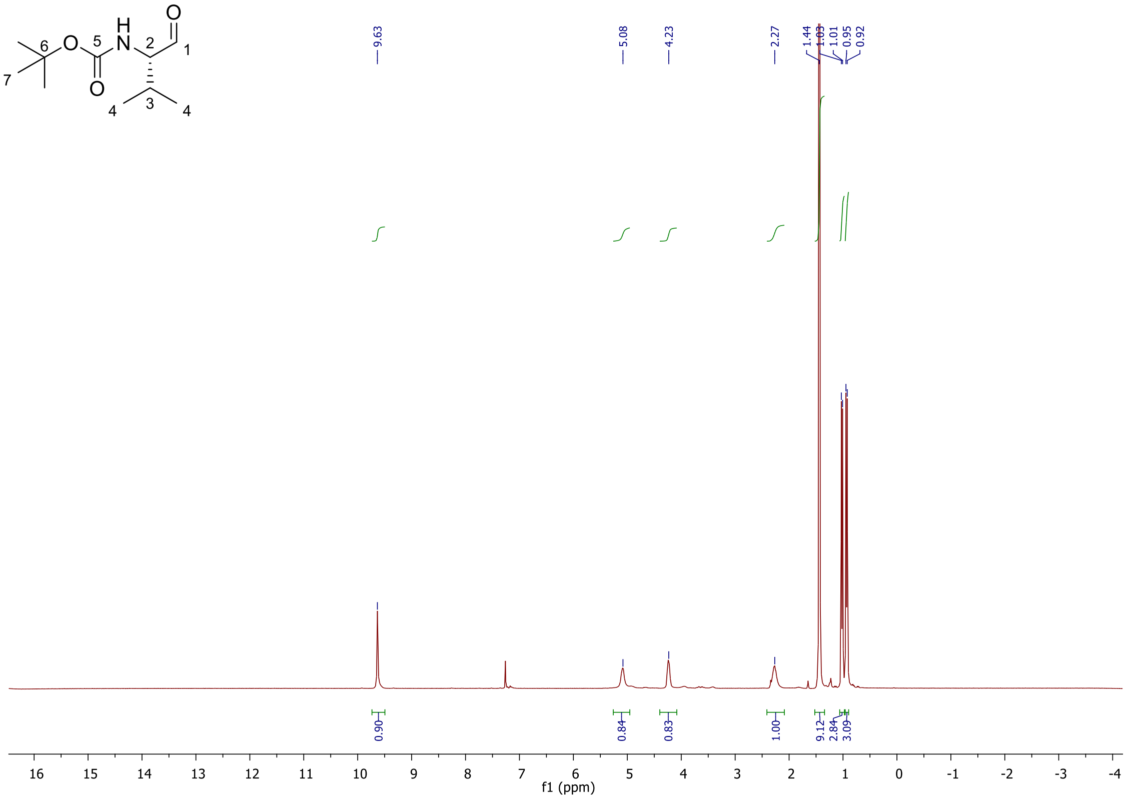

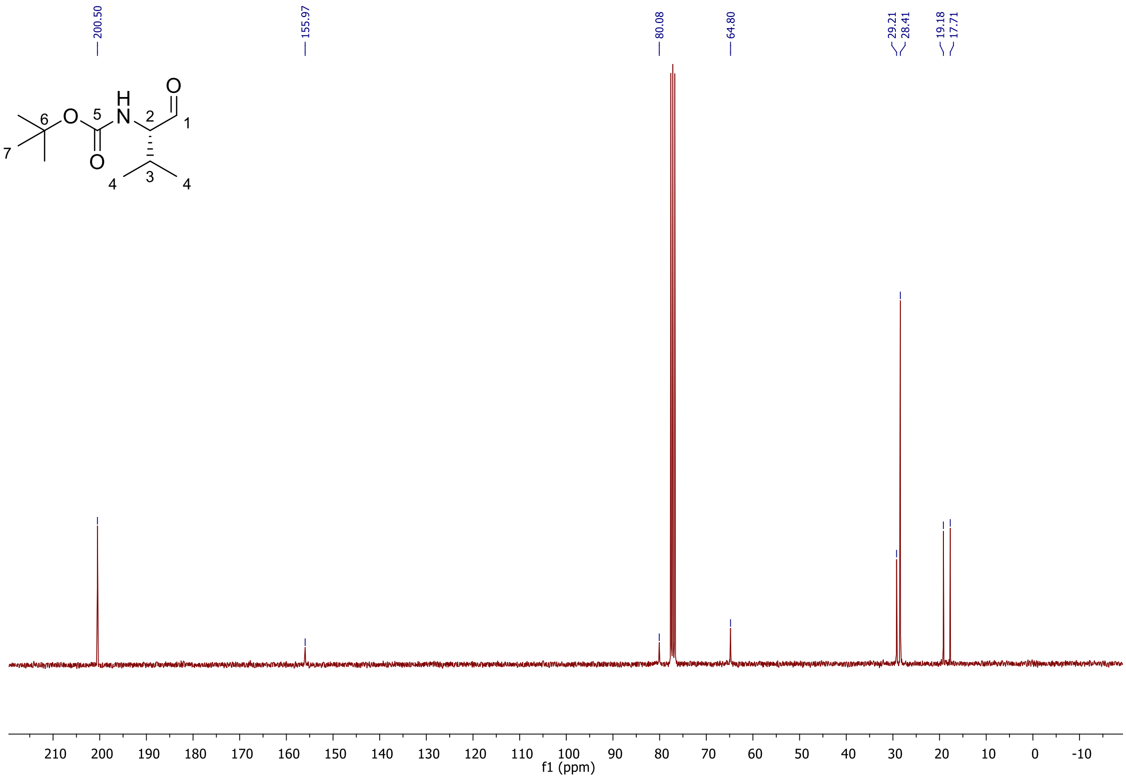

^{1}H and ^{13}C NMR spectra of Ethyl (5*S*)-5-{[(*tert*-butoxy)carbonyl]amino}-4-hydroxy-6-methylhept-2-ynoate (3)

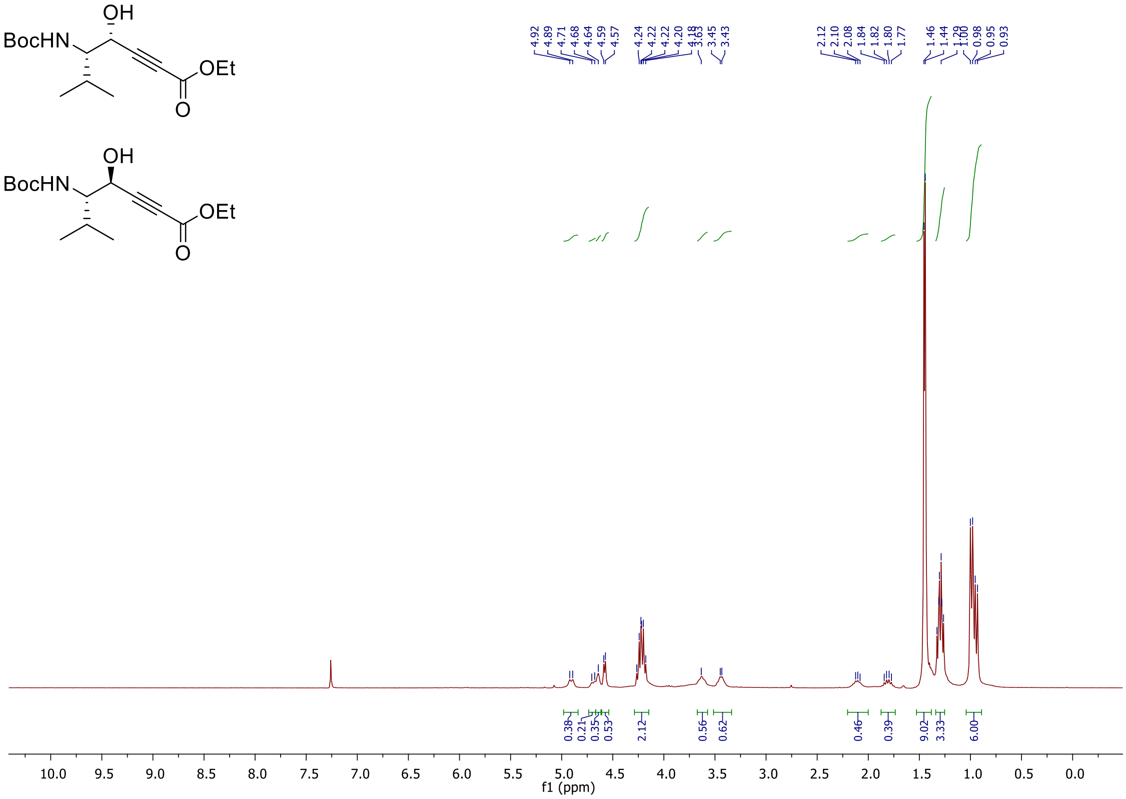

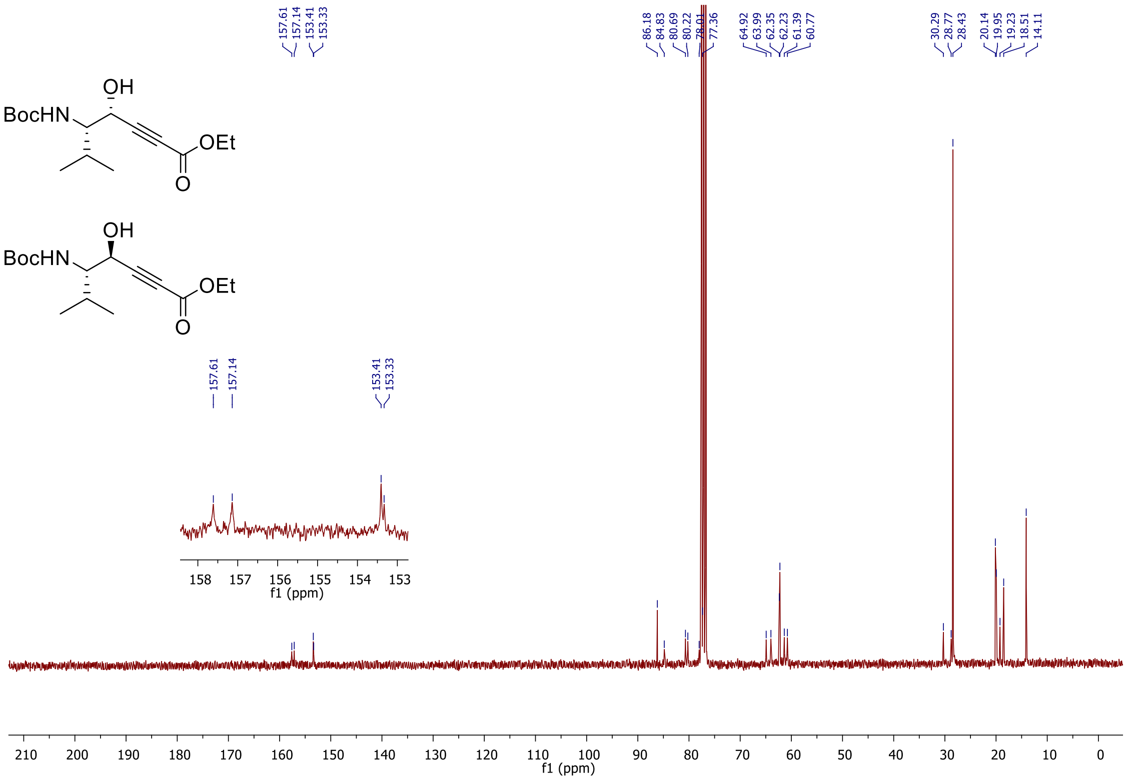

^{1}H and ^{13}C NMR spectra of *tert*-Butyl *N*-[(1*S*)-2-methyl-1-[(2*S*)-5-oxooxolan-2-yl]propyl]carbamate (4)

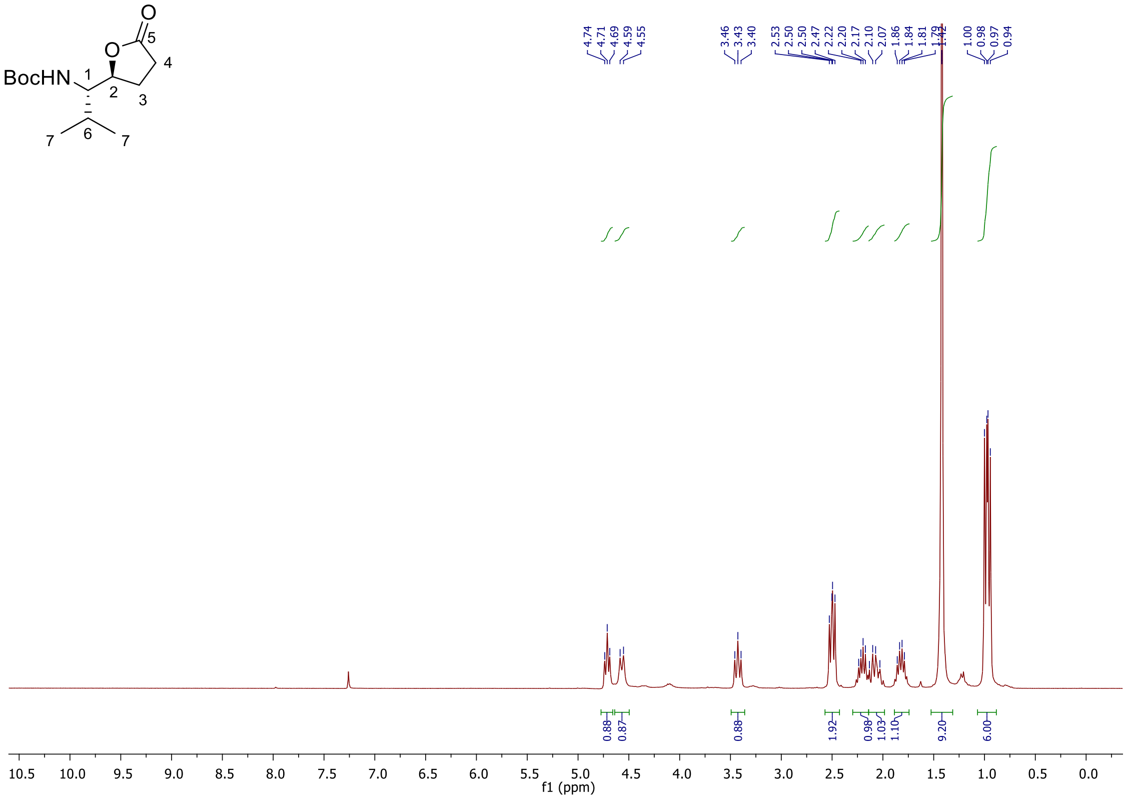

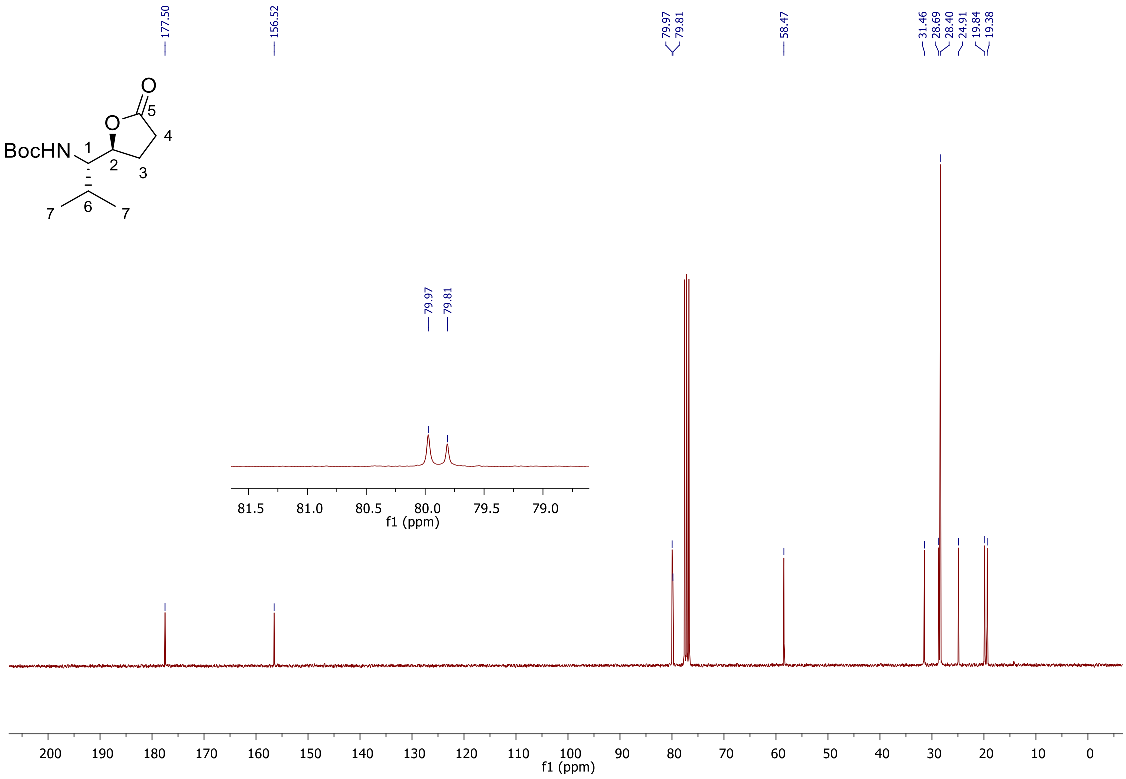

^{1}H and ^{13}C NMR spectra of *tert*-butyl *N*-[(1*S*)-2-methyl-1-[(2*R*)-5-oxooxolan-2-yl]propyl]carbamate (5)

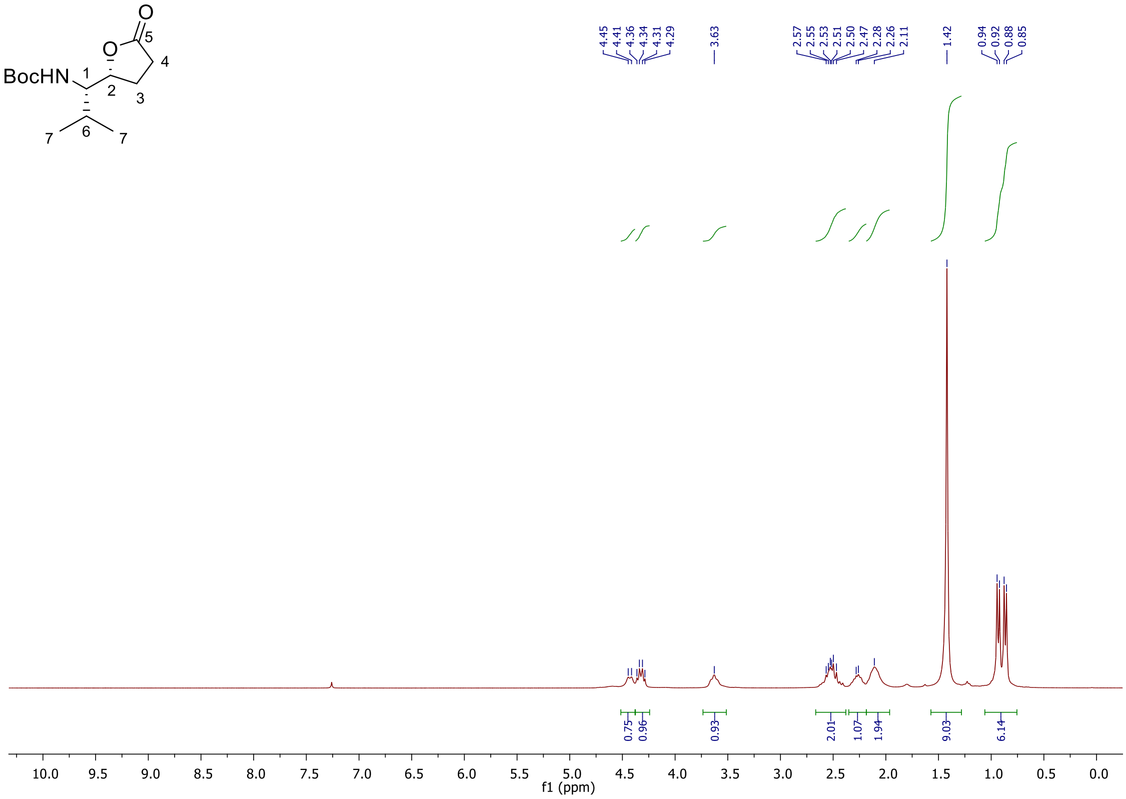

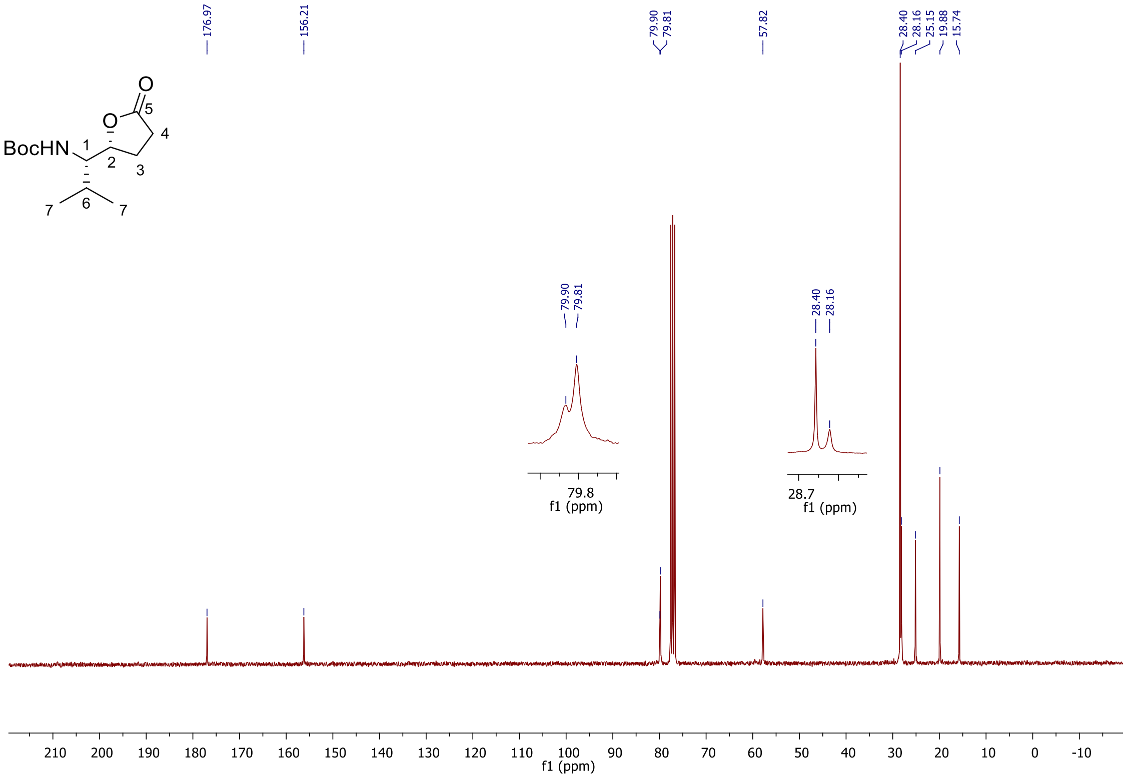

^{1}H and ^{13}C NMR spectra of *tert*-Butyl *N*-[(1*S*)-1-[(2*S*,4*R*)-4-benzyl-5-oxooxolan-2-yl]-2-methylpropyl]carbamate (6)

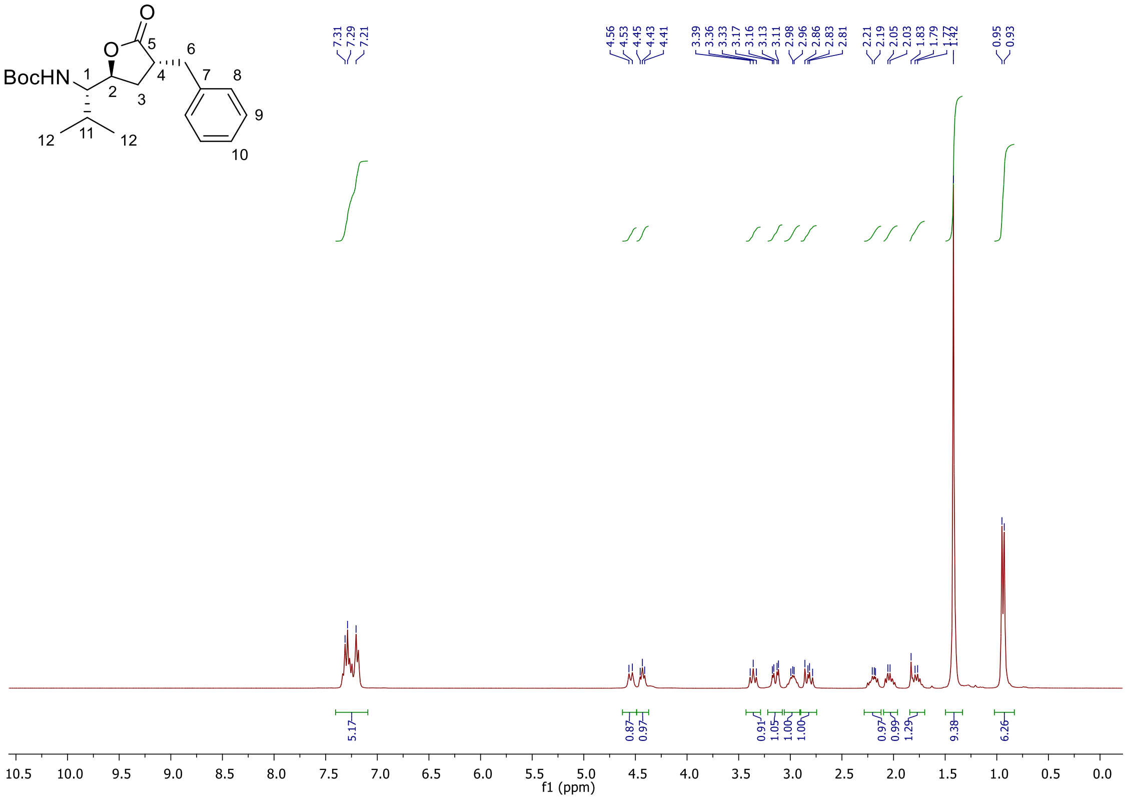

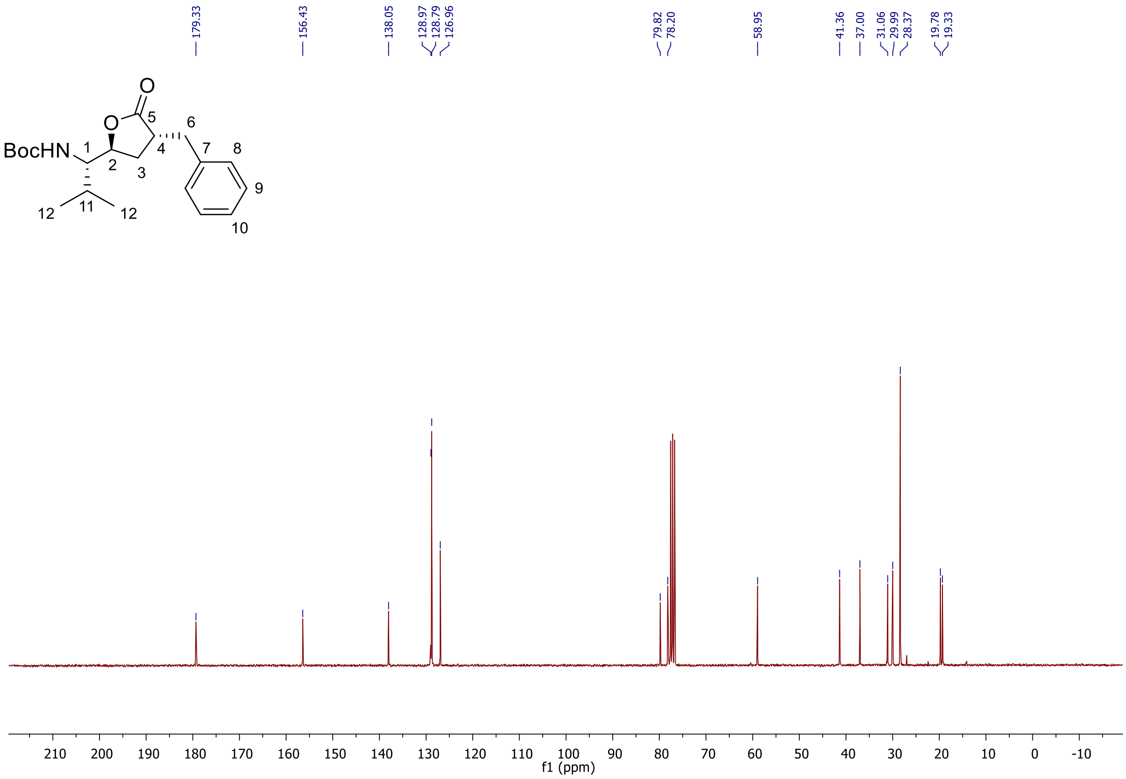

^{1}H and ^{13}C NMR spectra of (2*R*,4*S*,5*S*)-2-Benzyl-5-{[(*tert*-butoxy)carbonyl]-amino}-4-[(*tert*-butyldimethylsilyl)oxy]-6-methylheptanoic acid (7)

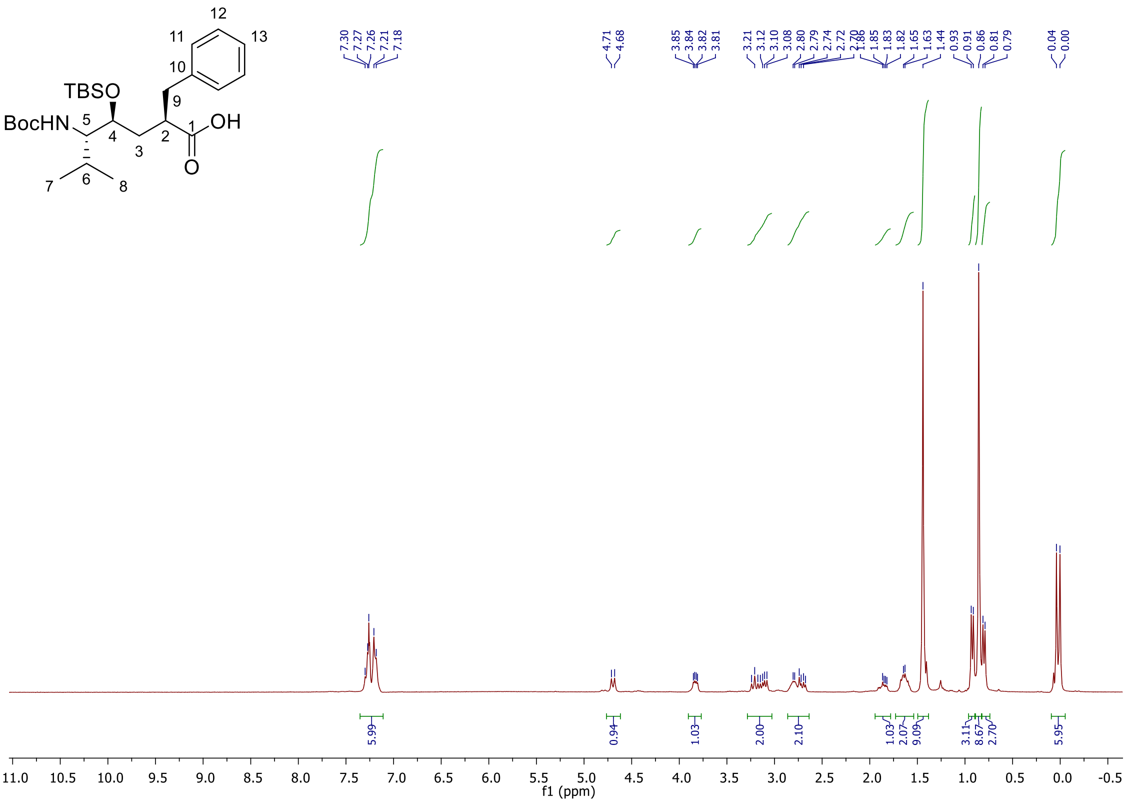

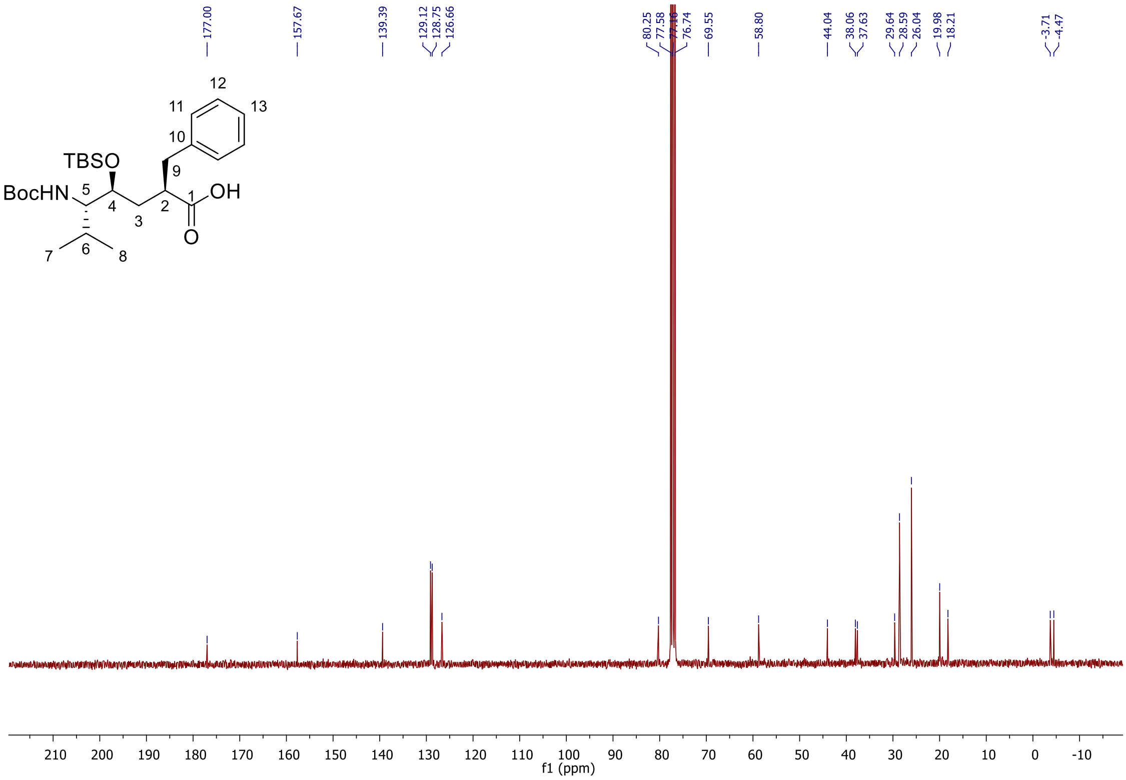

^{1}H and ^{13}C NMR spectra of *tert*-Butyl (2*S*)-2-{[(2*S*)-3-(1*H*-indol-3-yl)-1-methoxy-1-oxopropan-2-yl]carbamoyl}pyrrolidine-1-carboxylate

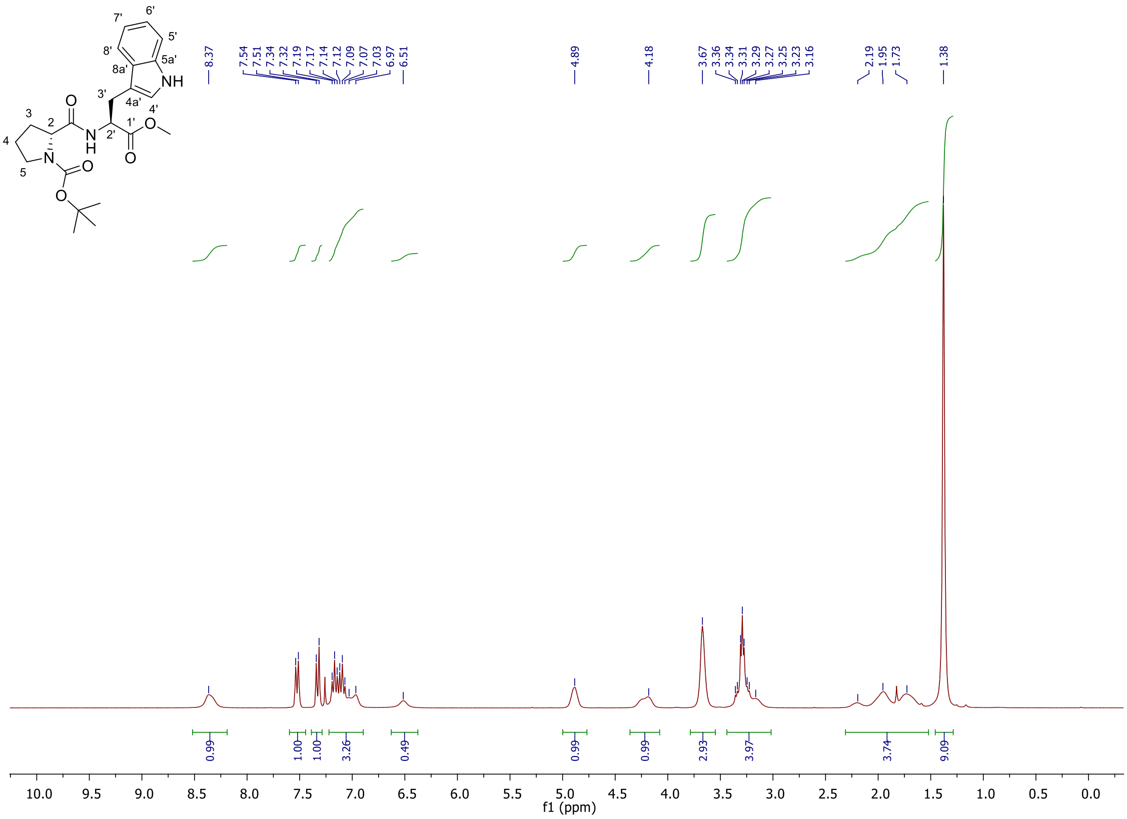

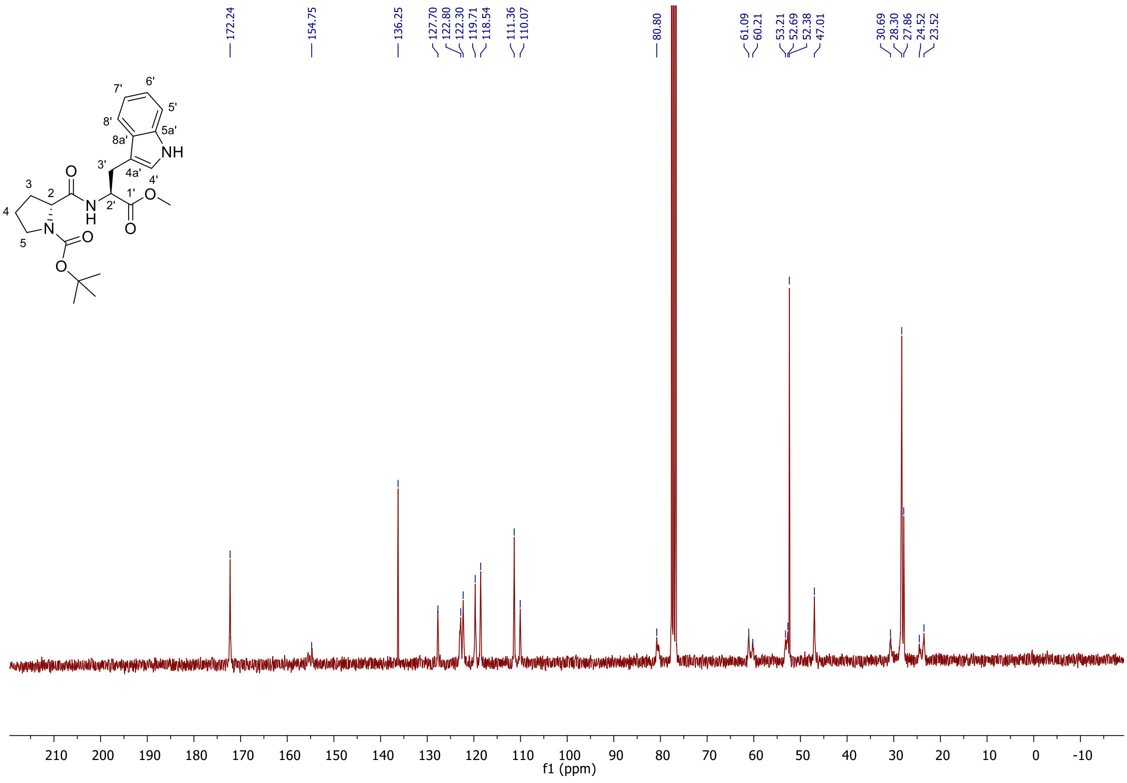

^{1}H and ^{13}C, NMR spectra of Methyl (2*S*)-2-{[(2*S*)-1-[(2*R*,4*S*,5*S*)-2-benzyl-5-{[(*tert*-butoxy)carbonyl]amino}-4-[(*tert*-butyldimethylsilyl)oxy]-6-methyl-heptanoyl]pyrrolidin-2-yl]formamido}-3-(1*H*-indol-3-yl)propanoate (8)

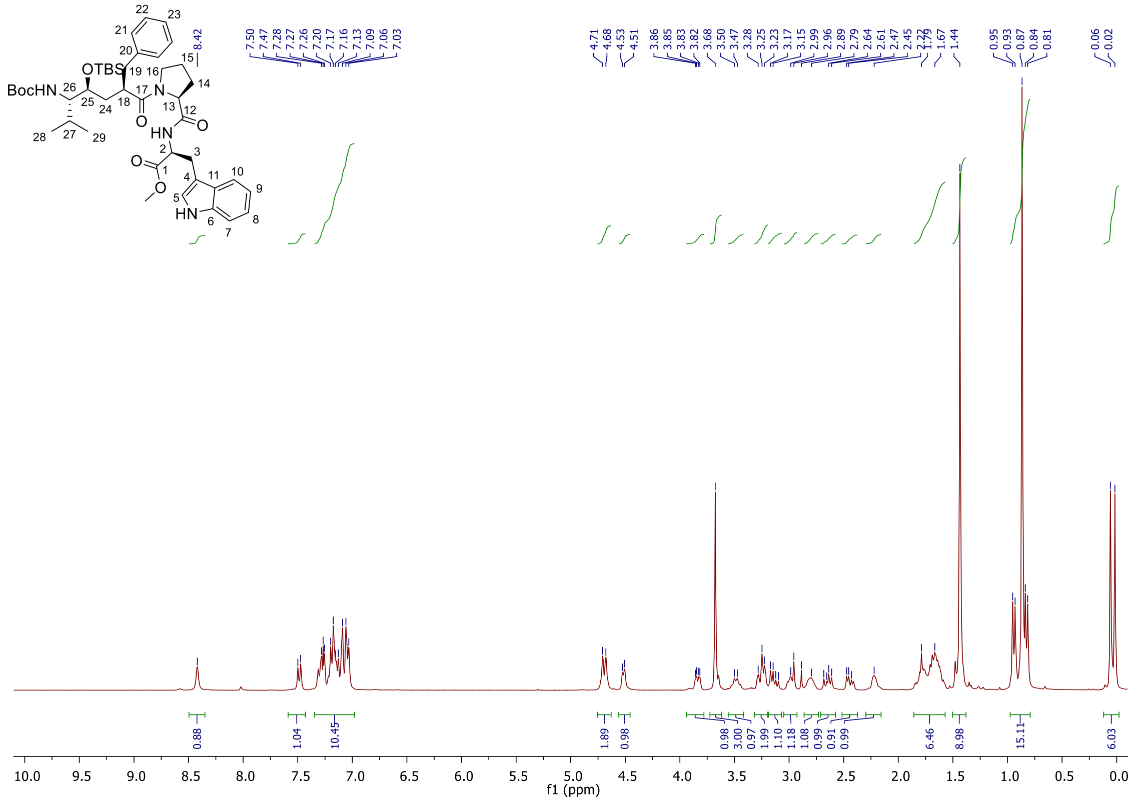

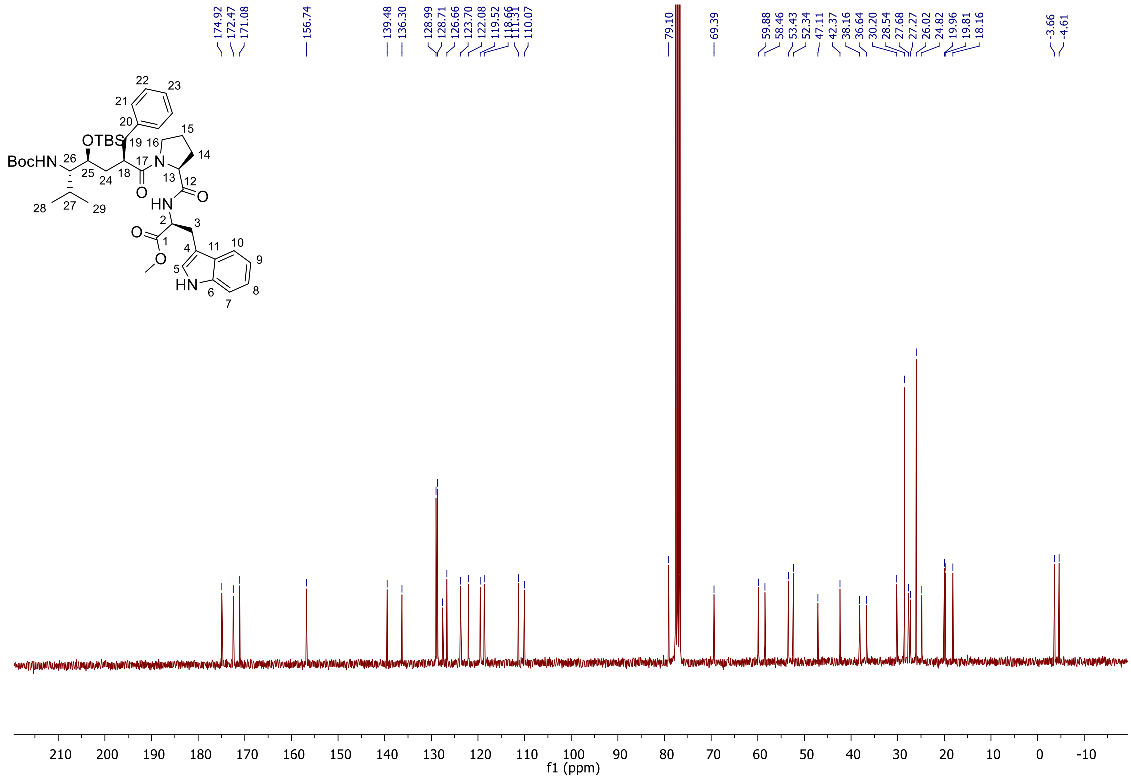

^{1}H, ^{13}C, COSY and HSQC (300/75 and 500/125 MHz) NMR spectra of Methyl (2*S*)-2-{[(2*S*)-1-[(2*R*,4*S*,5*S*)-2-benzyl-5-[(2*S*)-2-{[(*tert*-butoxy)carbonyl]amino}-3-methylbutanamido]-4-hydroxy-6-methylheptanoyl]pyrrolidin-2-yl]formamido}-3-(1*H*-indol-3-yl)propanoate (9)

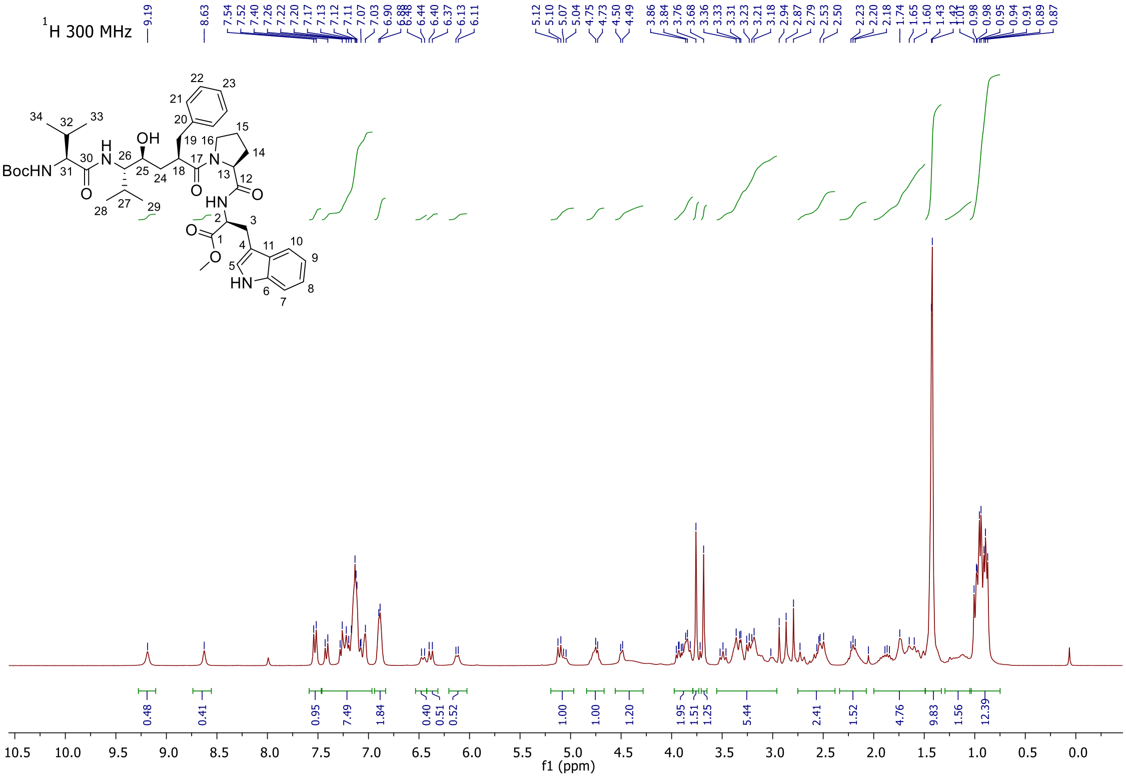

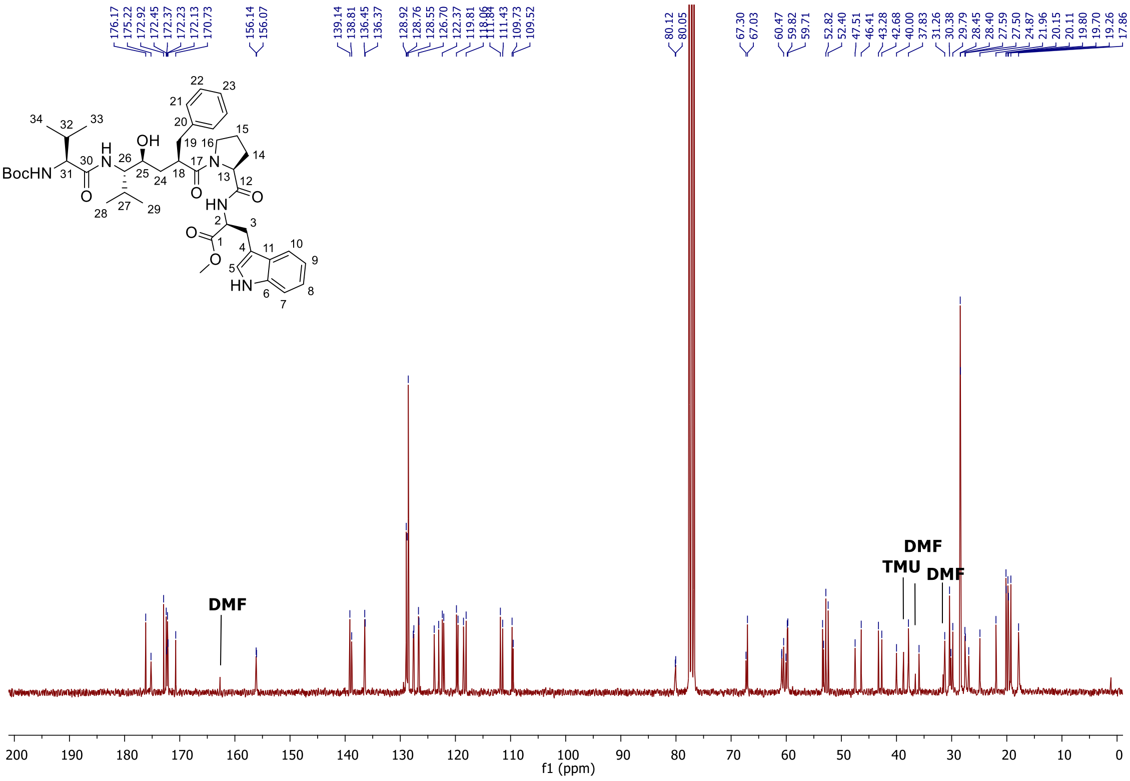

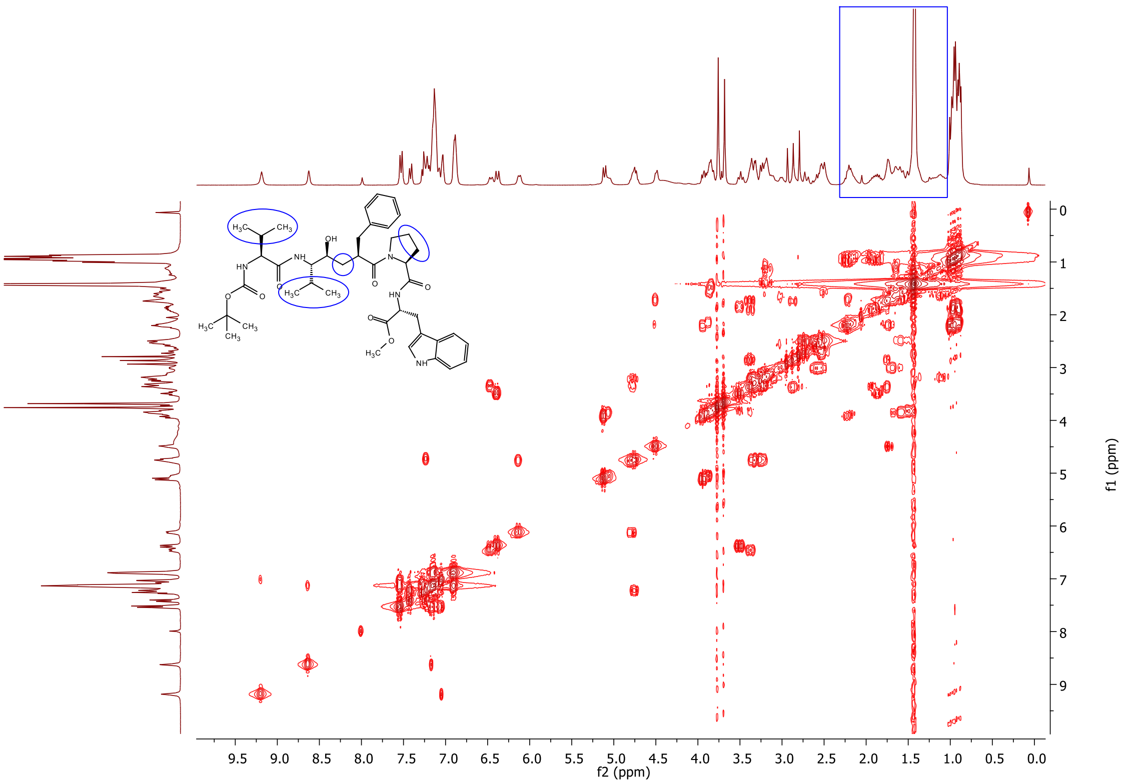

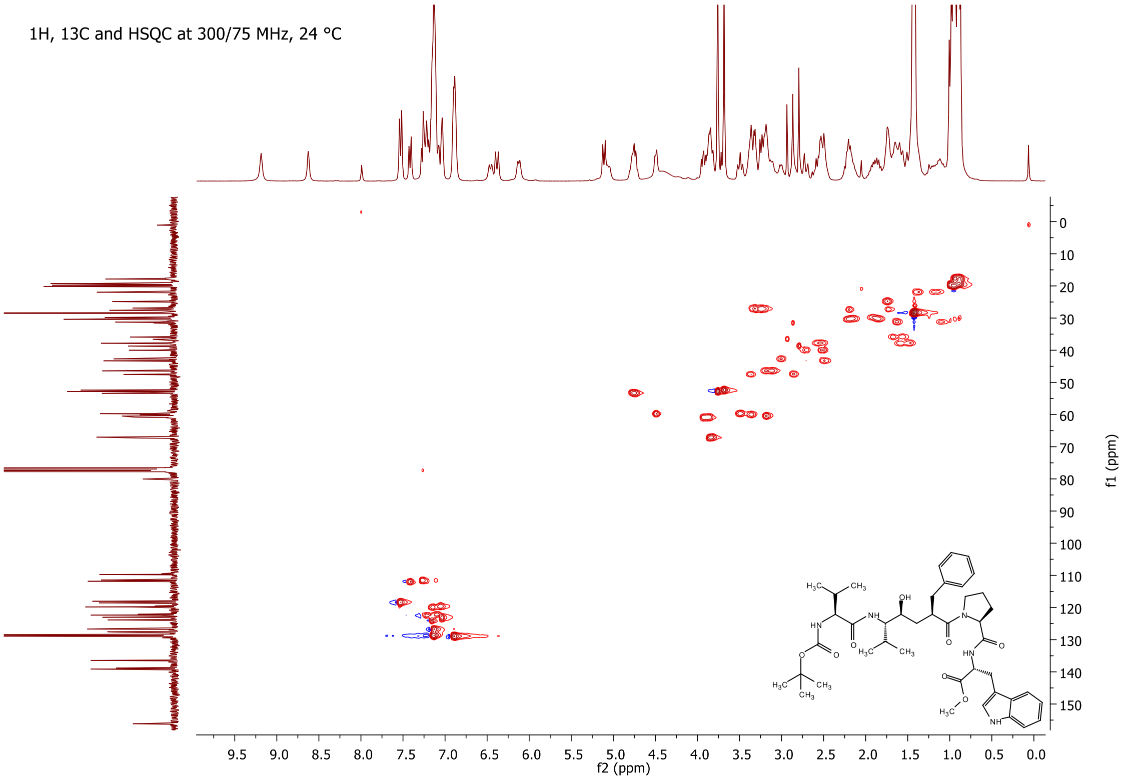

1H, 13C and HSQC at 300/75 MHz, 24 °C

1H, 13C and HSQC at 500/125 MHz, 30 °C

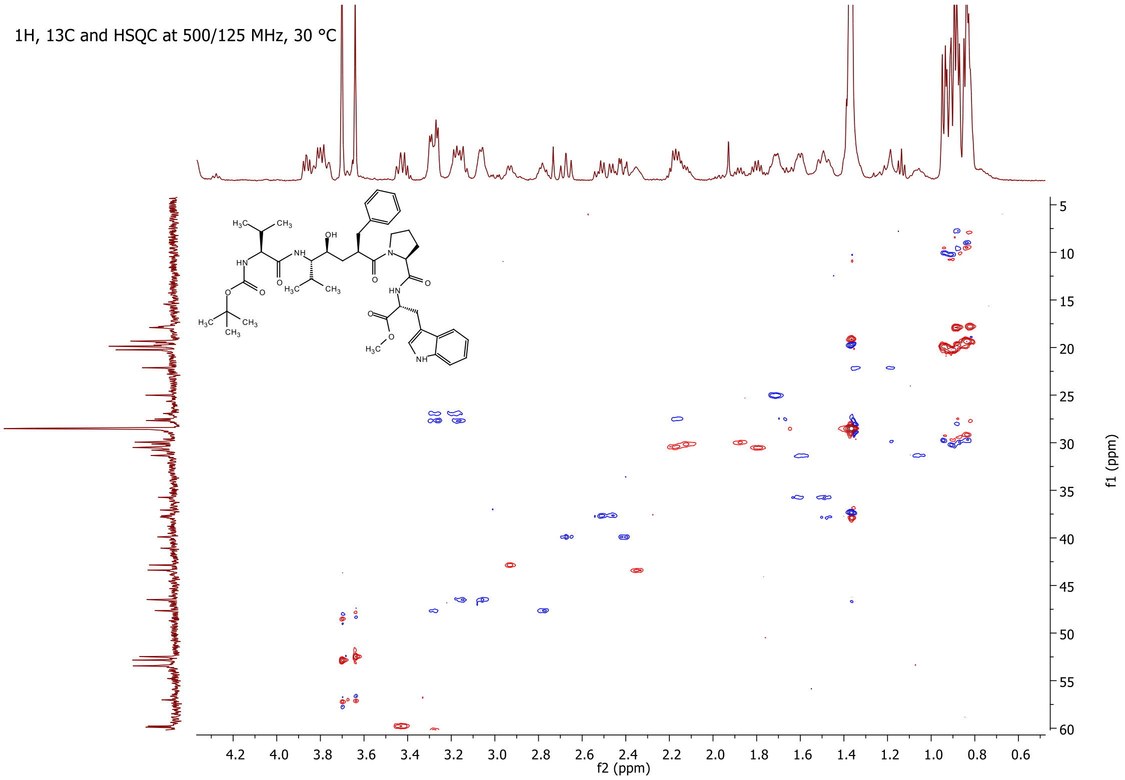

^{1}H, ^{13}C, COSY and HSQC NMR spectra of (2*S*)-2-{[(2*S*)-1-[(2*R*,4*S*,5*S*)-5-[(2*S*)-2-Azaniumyl-3-methylbutanamido]-2-benzyl-4-hydroxy-6-methyl-heptanoyl]pyrrolidin-2-yl]formamido}-3-(1*H*-indol-3-yl)propanoate (10)

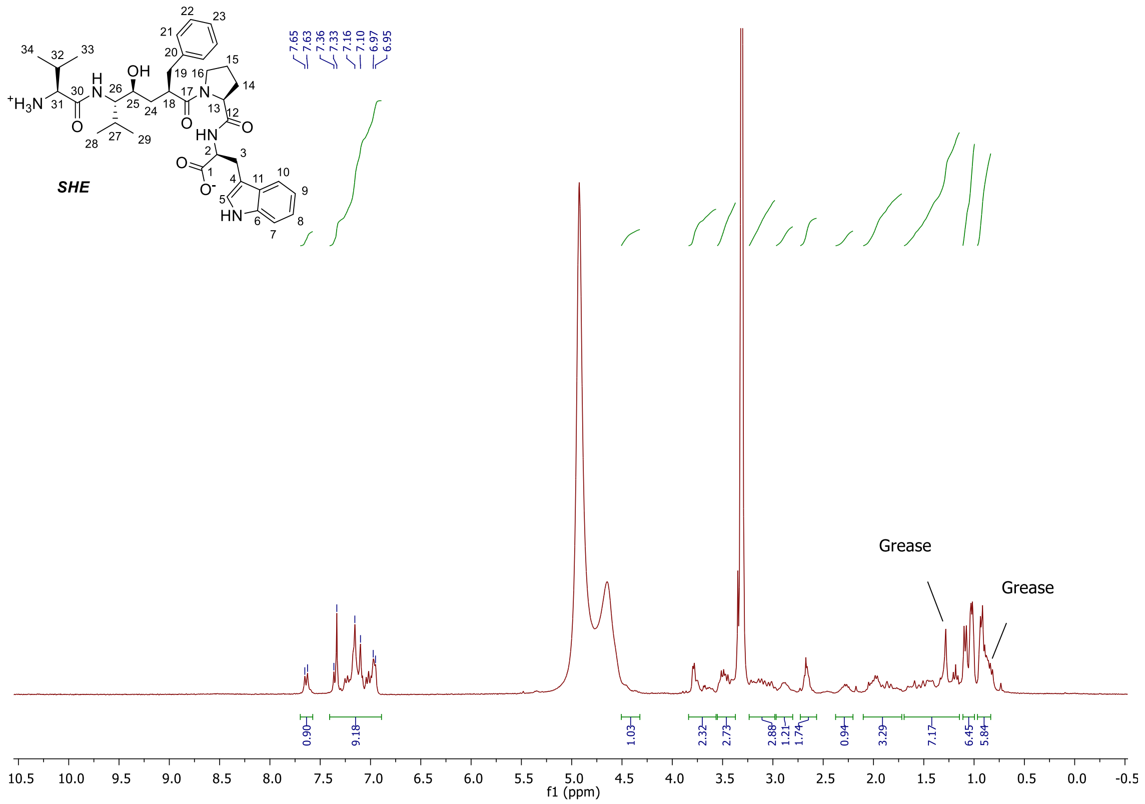

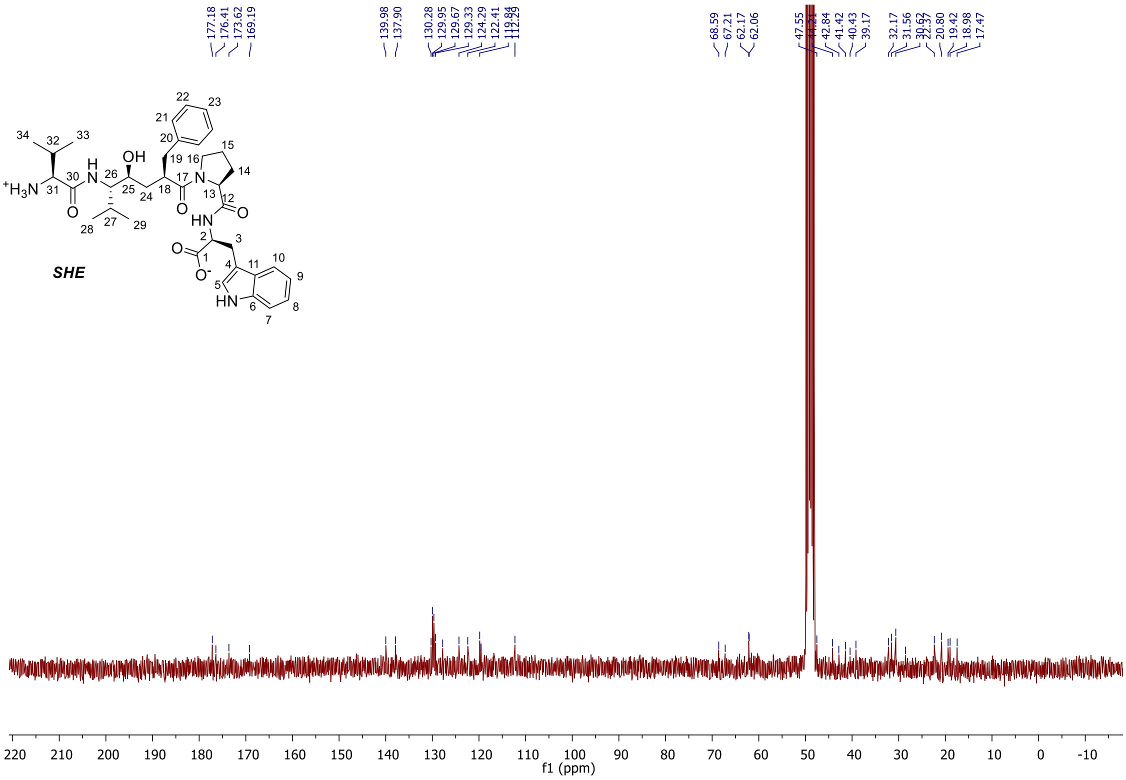
177.18
176.41
173.62
169.19
139.98
137.90
130.28
129.95
129.67
129.33
124.29
122.41
68.59
67.21
62.17
62.06
47.55
42.84
41.42
40.43
39.17
32.17
31.56
20.80
19.42
18.98
17.47
SHE
220 210 200 190 180 170 160 150 140 130 120 110 100 90 80 70 60 50 40 30 20 10 0 -10
f1 (ppm)

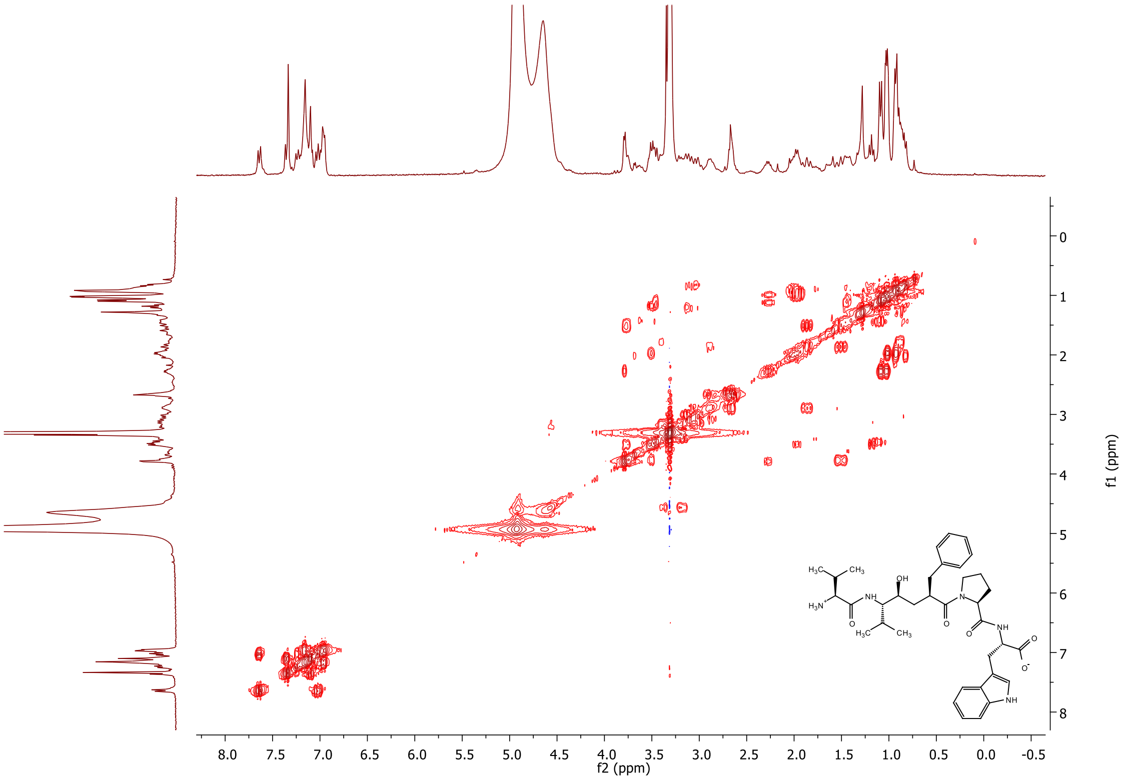
f1 (ppm)
f2 (ppm)
OH
NH
H2N
N
O
O-
NH
CH3
H3C

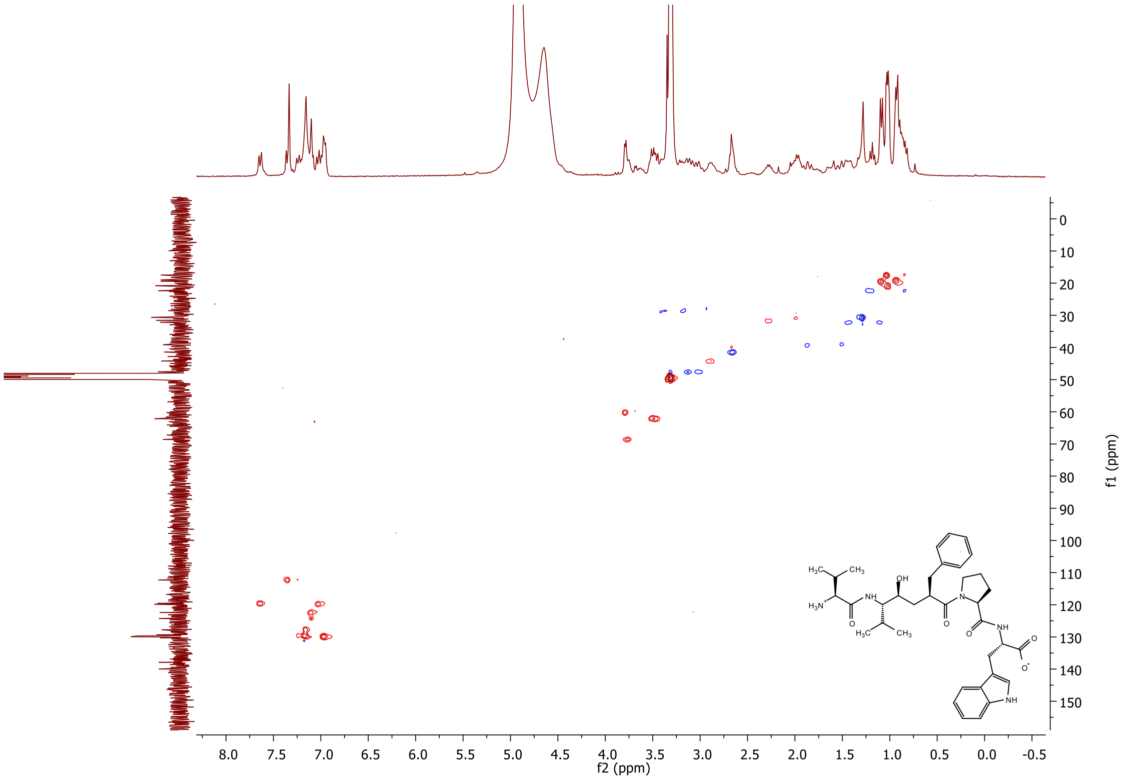
f1 (ppm)
f2 (ppm)
OH
NH
H2N
N
O
O-
NH
CH3
H3C

^{1}H and ^{13}C-APT NMR spectra of Methyl (*tert*-butoxycarbonyl)-L-valinate (11)

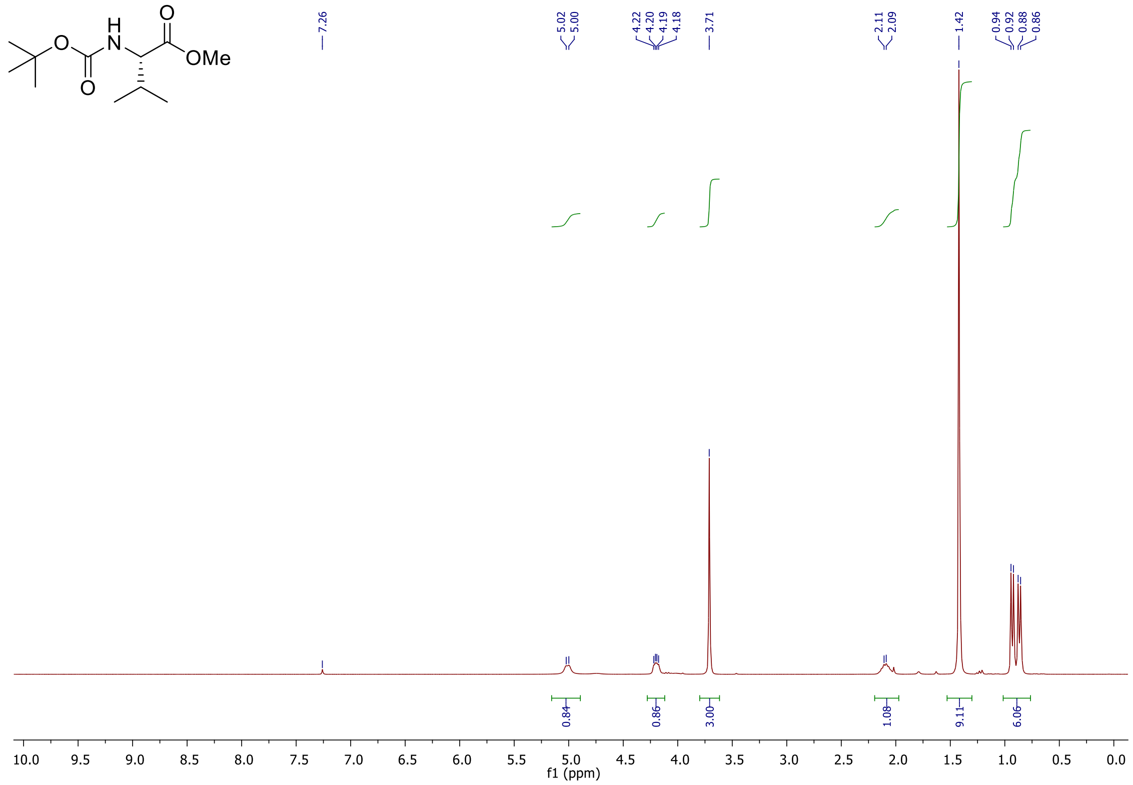

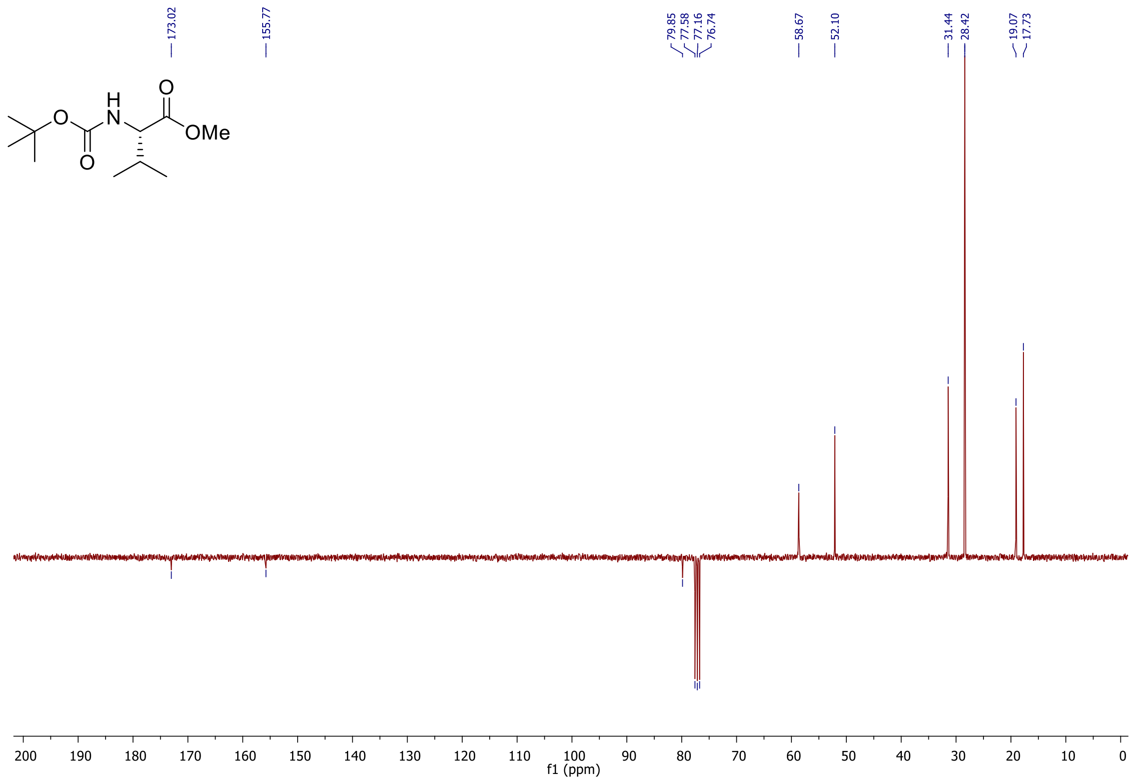

^{1}H and ^{13}C NMR spectra of *tert*-Butyl (*S*)-(1-(dimethoxyphosphoryl)-4-methyl-2-oxopentan-3-yl)carbamate (12)

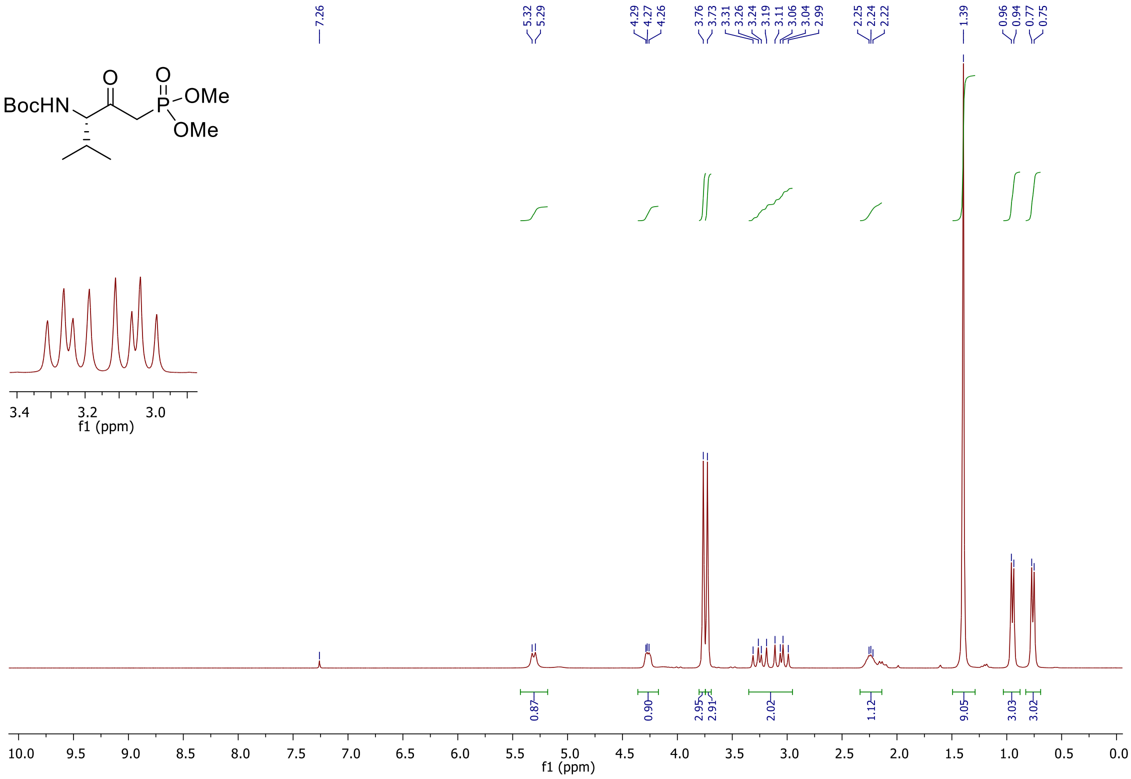

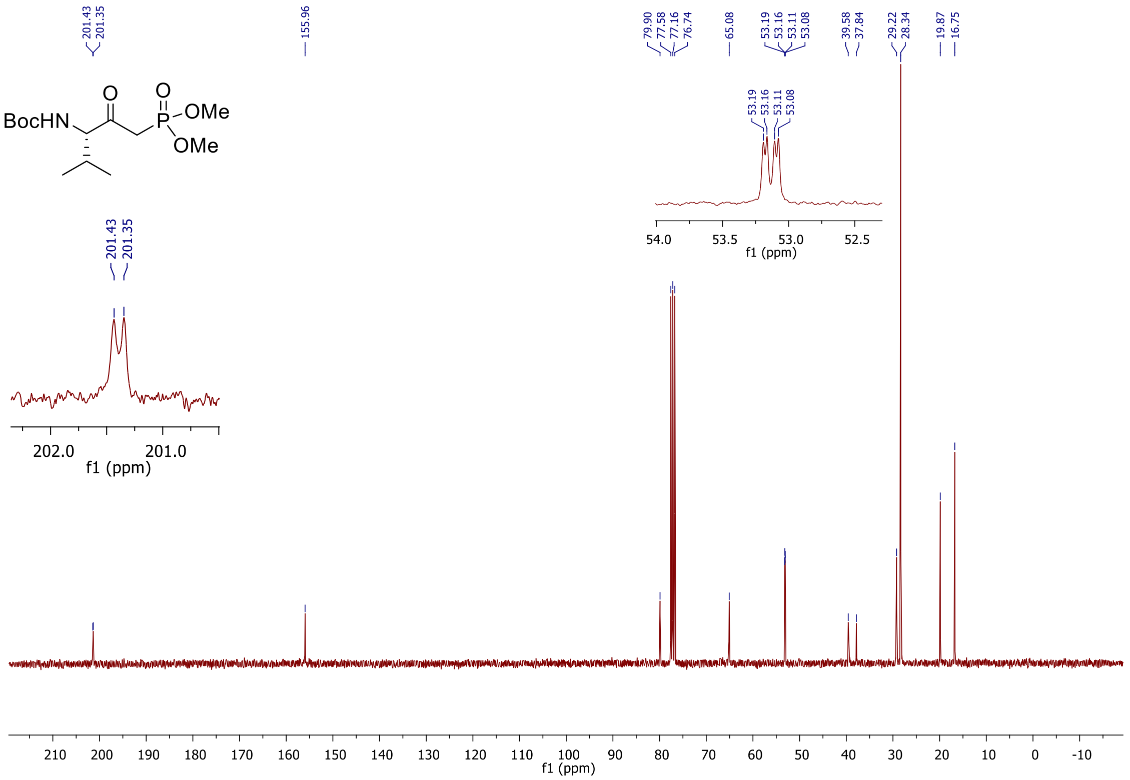

^{1}H and ^{13}C NMR spectra of Methyl (*S*)-5-((*tert*-butoxycarbonyl)amino)-6-methyl-4-oxoheptanoate (15)

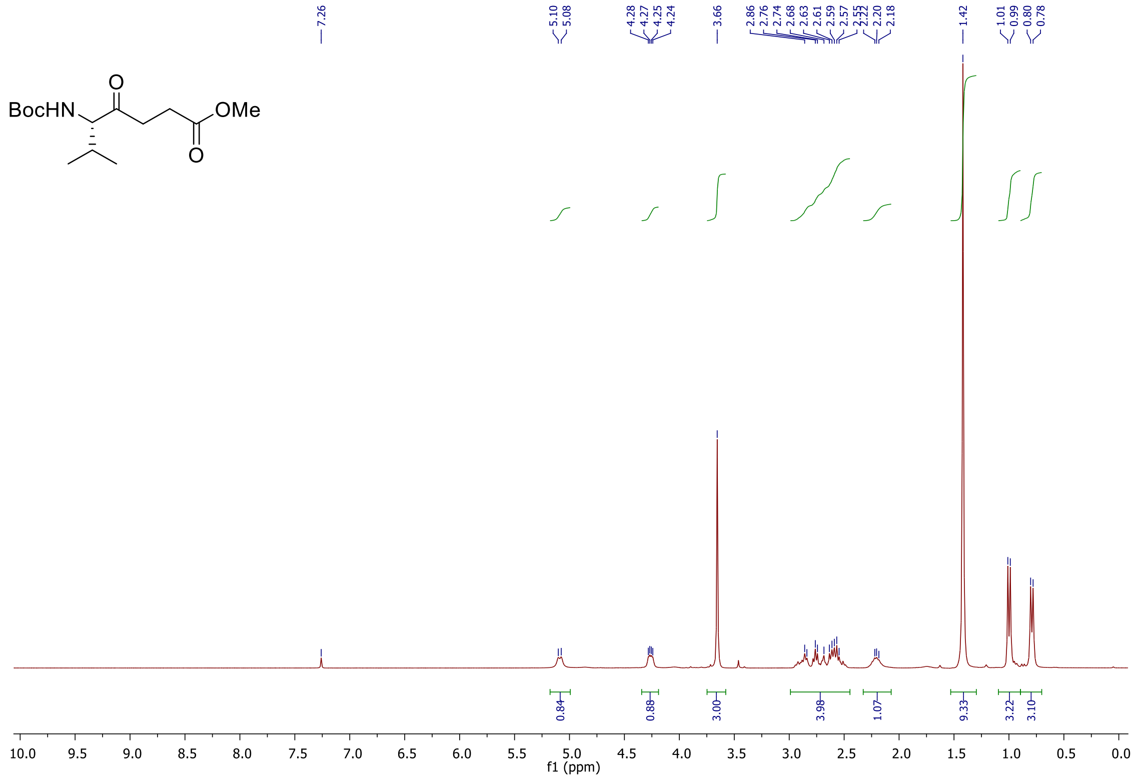

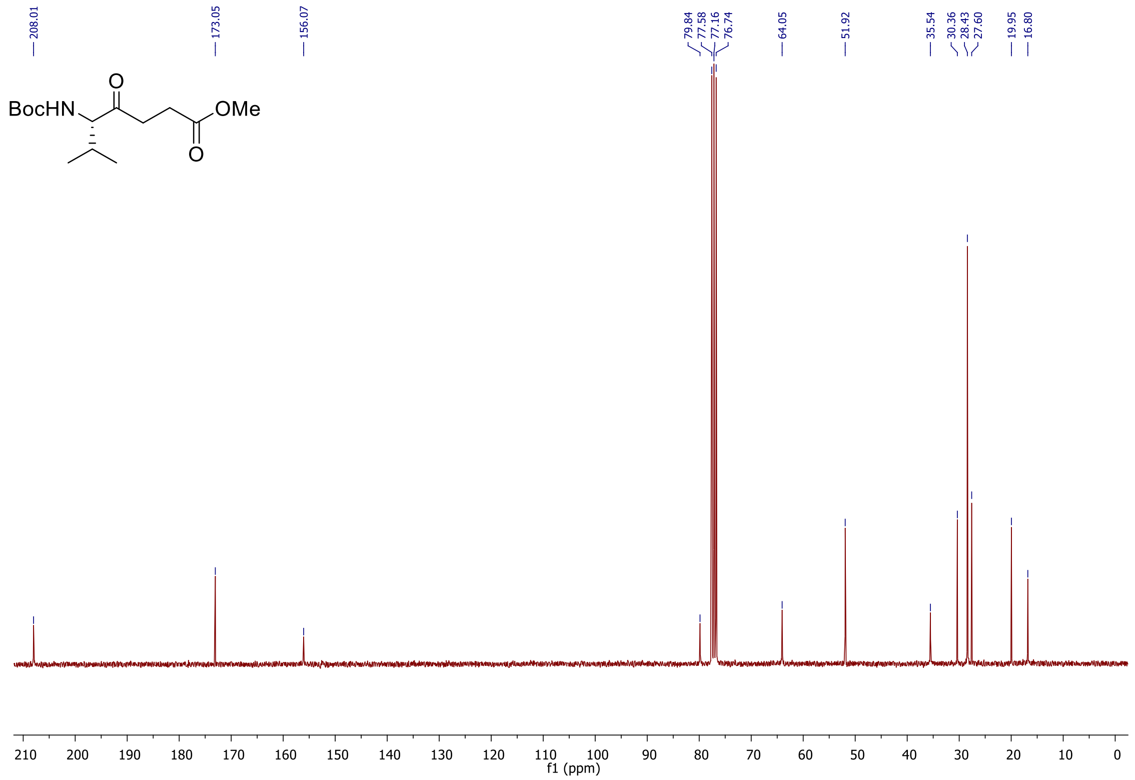

^{1}H and ^{13}C NMR spectra of *tert*-Butyl ((*S*)-2-methyl-1-((*R*)-5-oxotetrahydrofuran-2-yl)propyl)carbamate (16)

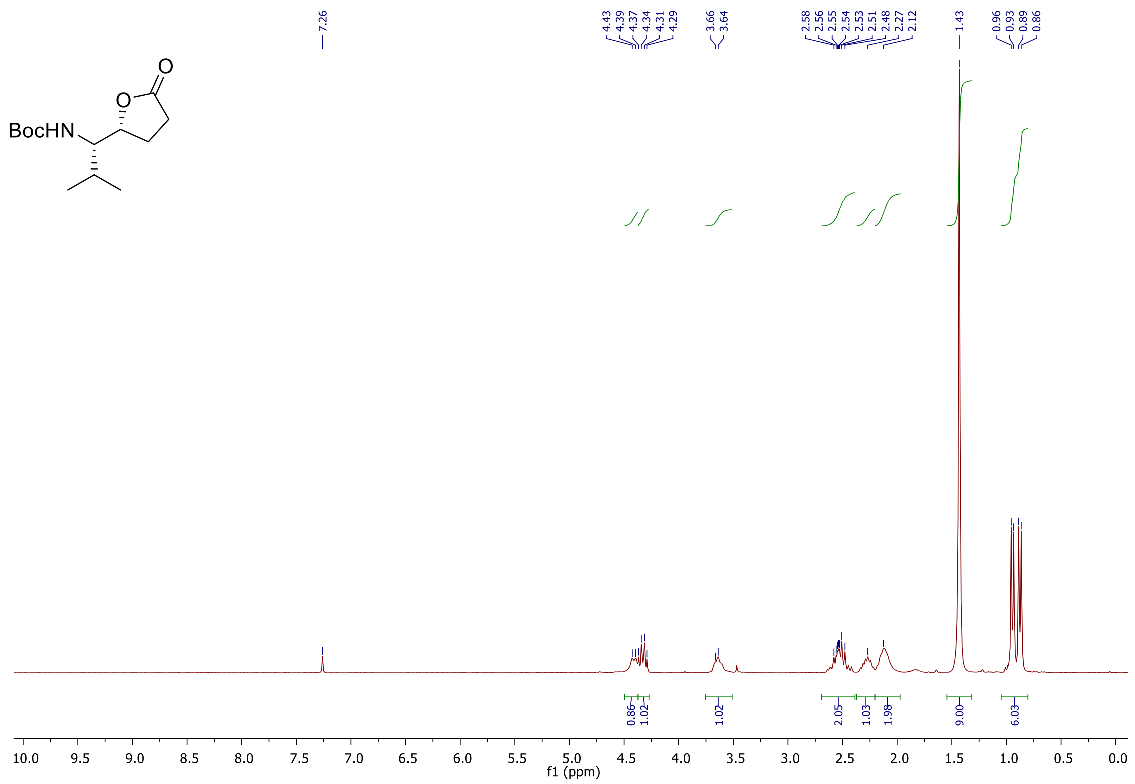

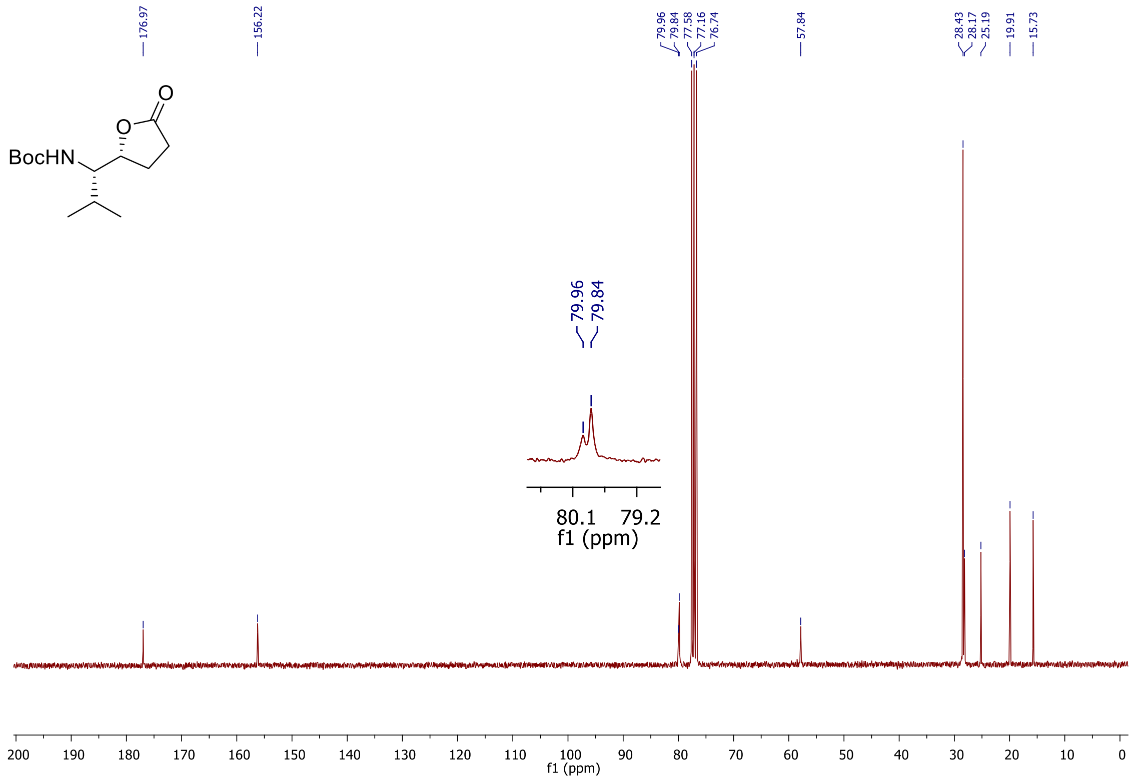

^{1}H NMR spectra of diastereomers of *tert*-butyl ((1*S*)-1-((2*R*)-4-(hydroxyl-(phenyl)methyl)-5-oxotetrahydrofuran-2-yl)-2-methylpropyl)carbamate (16a)

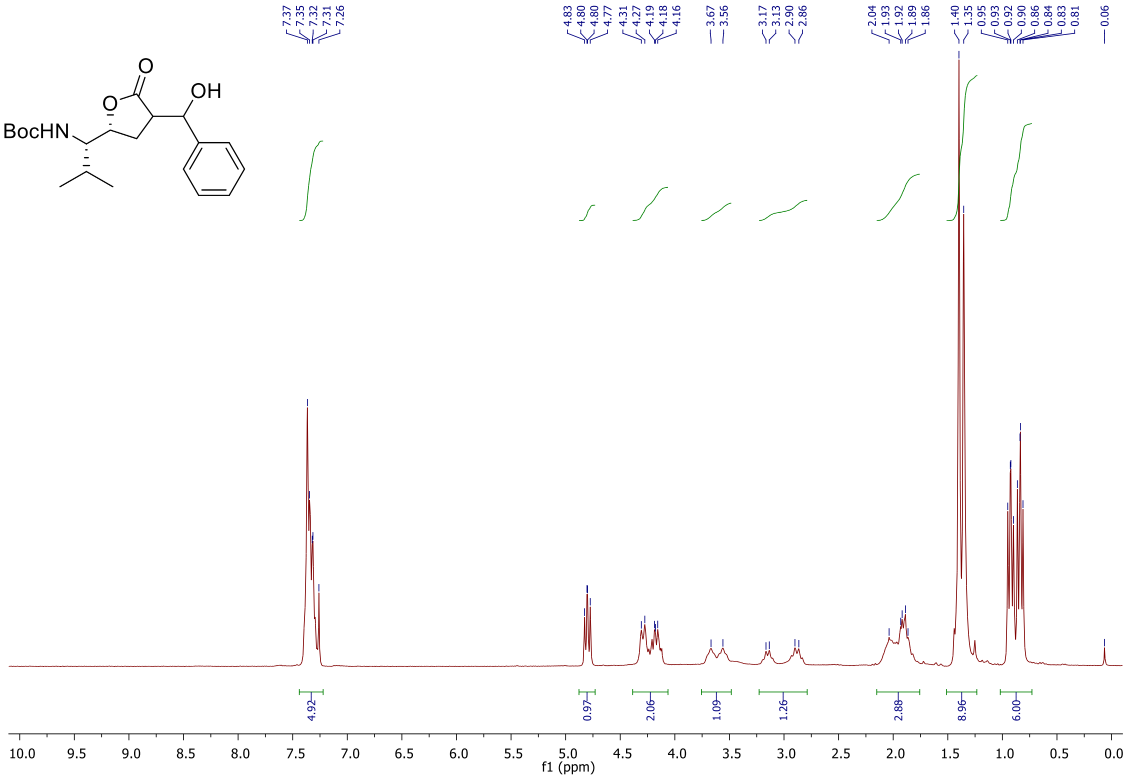

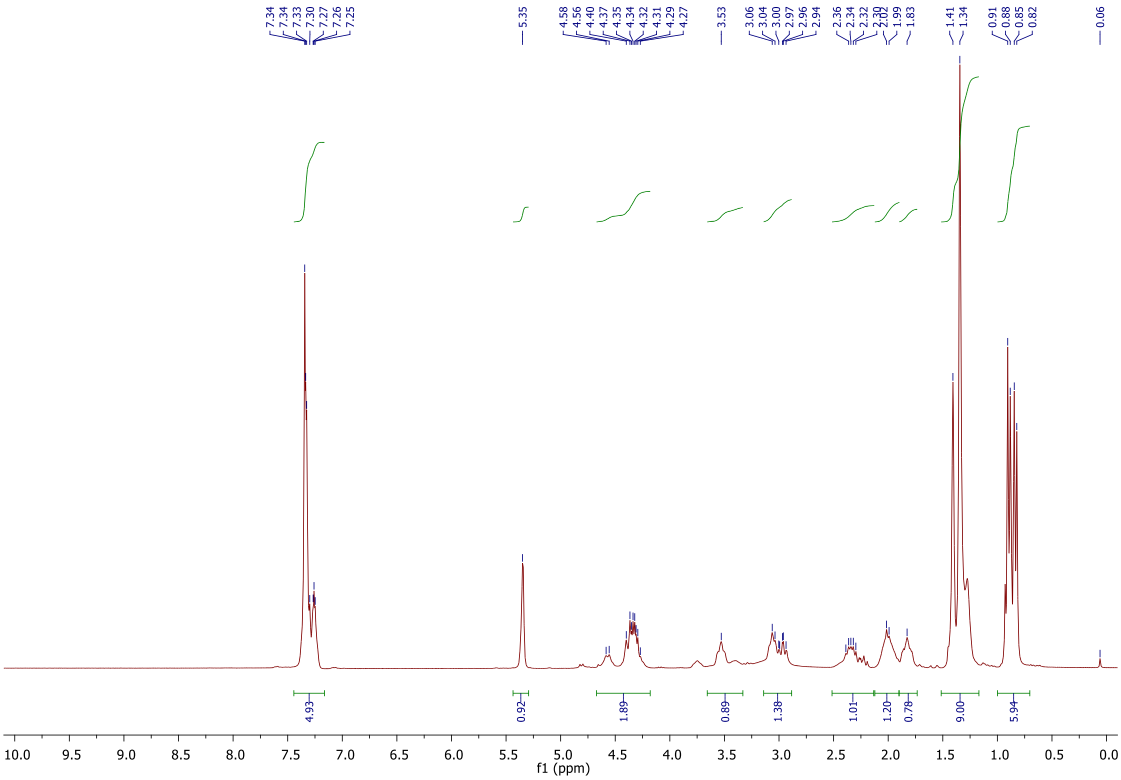

^{1}H and ^{13}C NMR spectra of *tert*-Butyl ((*S*)-1-((2*R*,4*R*)-4-benzyl-5-oxotetra-hydrofuran-2-yl)-2-methylpropyl)carbamate (17)

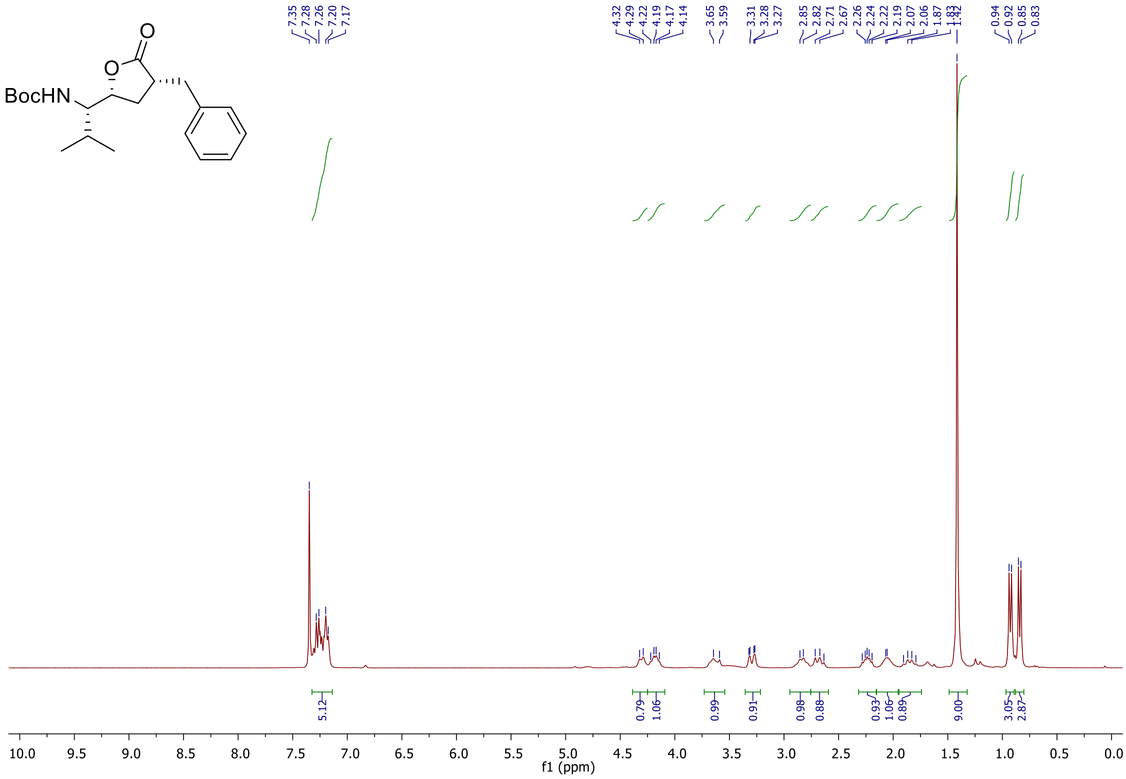

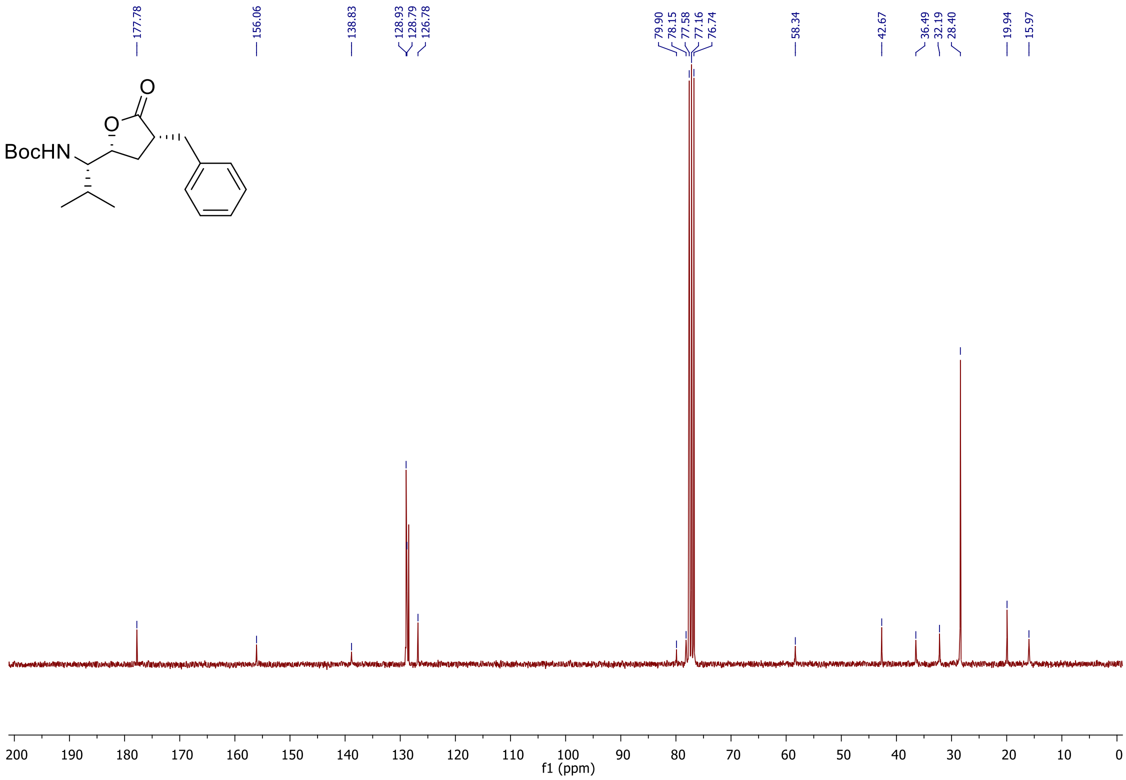

^{1}H and ^{13}C NMR spectrum of (2*R*,4*R*,5*S*)-2-Benzyl-5-((*tert*-butoxycarbonyl)-amino)-4-((*tert*-butyldimethylsilyl)oxy)-6-methylheptanoic acid (RGSA) (18)

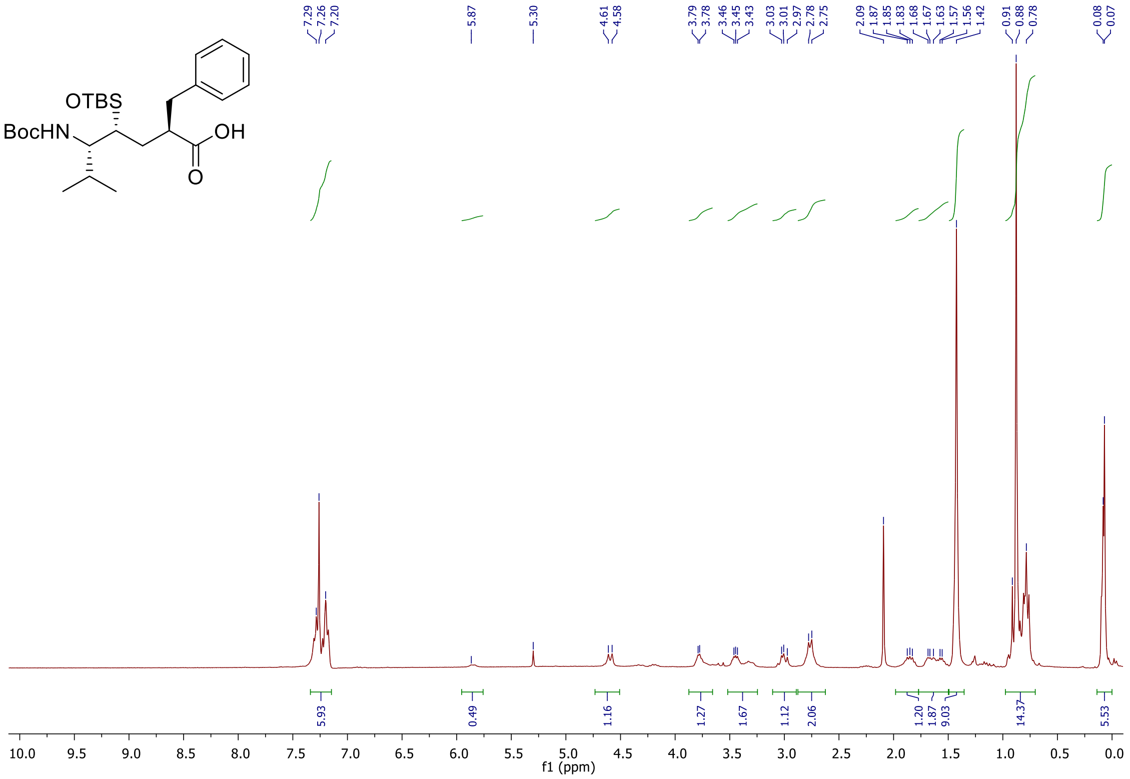

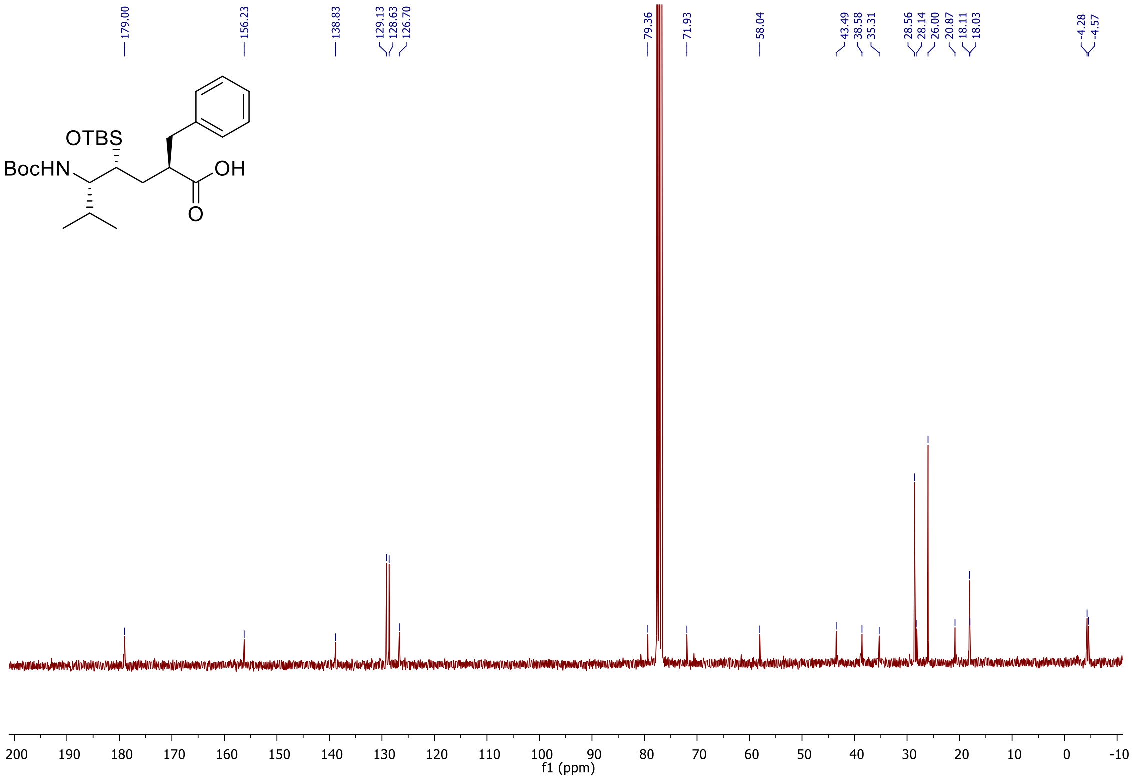

^{1}H and ^{13}C-APT NMR NMR spectrum of Methyl ((2*R*,4*R*,5*S*)-2-benzyl-5-((*tert*-butoxycarbonyl)amino)-4-((*tert*-butyldimethylsilyl)oxy)-6-methylheptanoyl)-L-prolyl-L-tryptophanate (Boc-RGSA-Pro-Trp-OMe) (19)

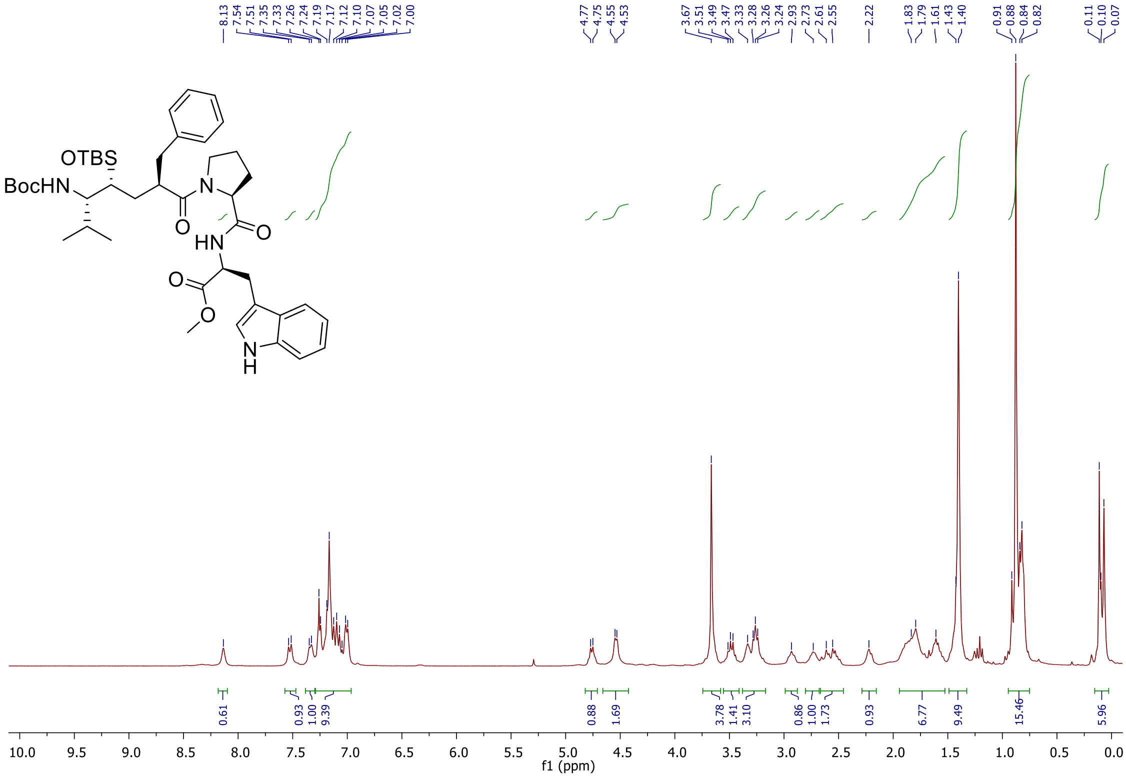

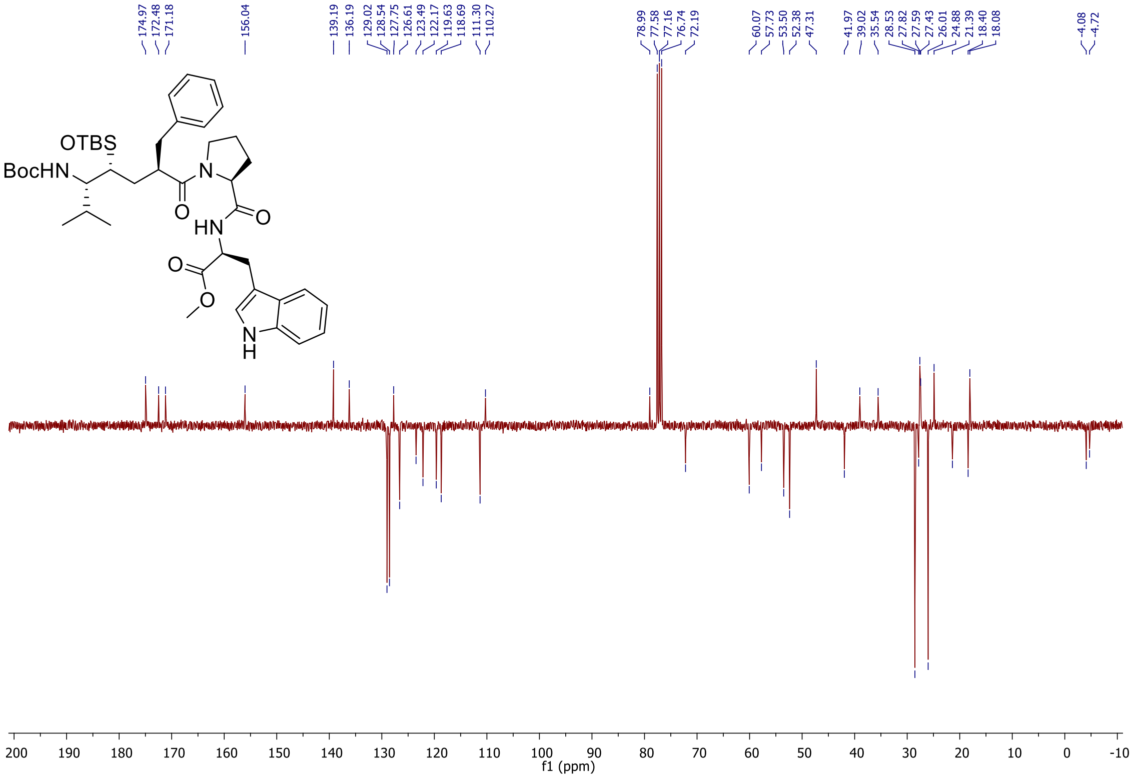

^{1}H and ^{13}C NMR spectra of Methyl ((2*R*,4*R*,5*S*)-2-benzyl-5-((*S*)-2-((*tert*-butoxycarbonyl)amino)-3-methylbutanamido)-4-((*tert*-butyldimethylsilyl)oxy)-6-methylheptanoyl)-L-prolyl-L-tryptophanate (fully protected *HER*) (20)

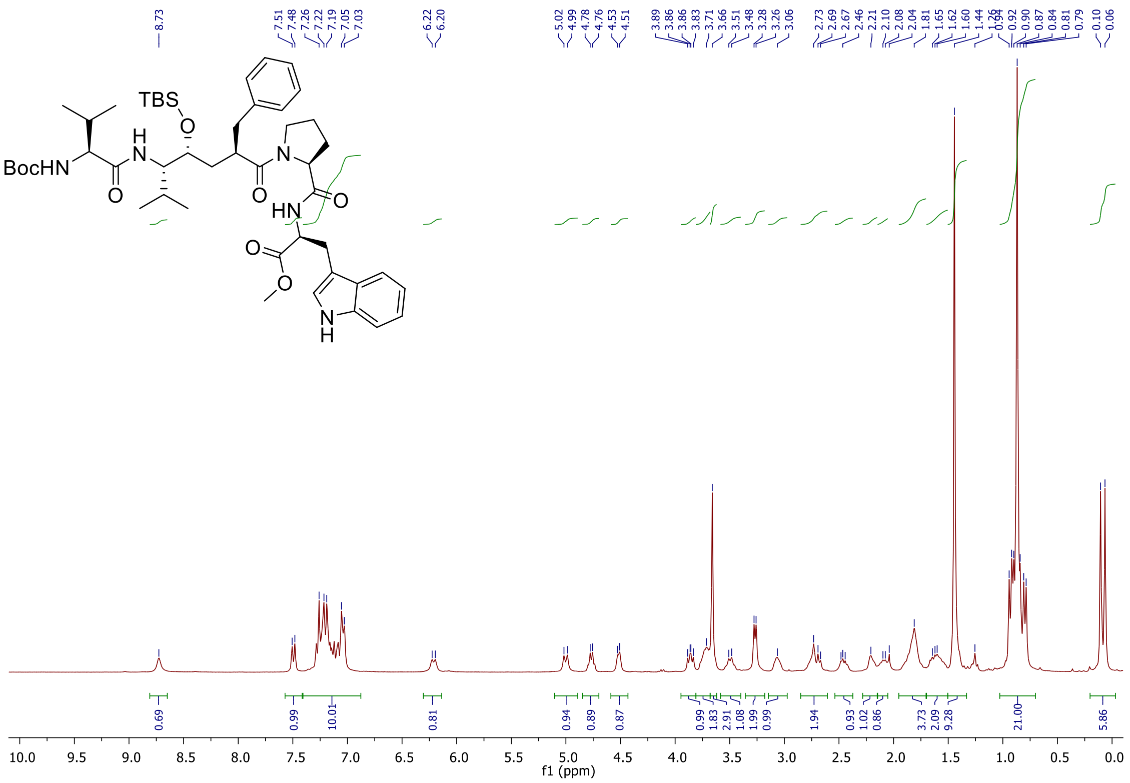

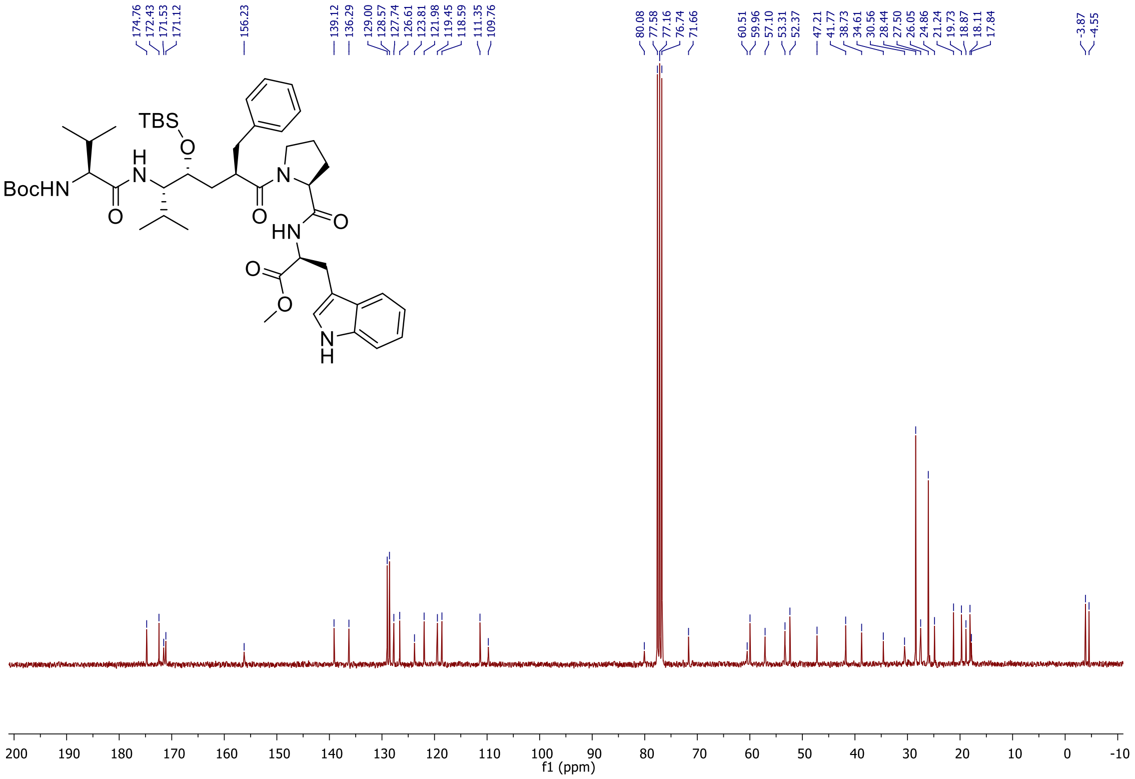

^{1}H and ^{13}C NMR spectra of Methyl ((2*R*,4*R*,5*S*)-2-benzyl-5-((*S*)-2-((*tert*-butoxycarbonyl)amino)-3-methylbutanamido)-4-hydroxy-6-methylheptanoyl)-L-prolyl-L-tryptophanate (protected *HER*) (21)

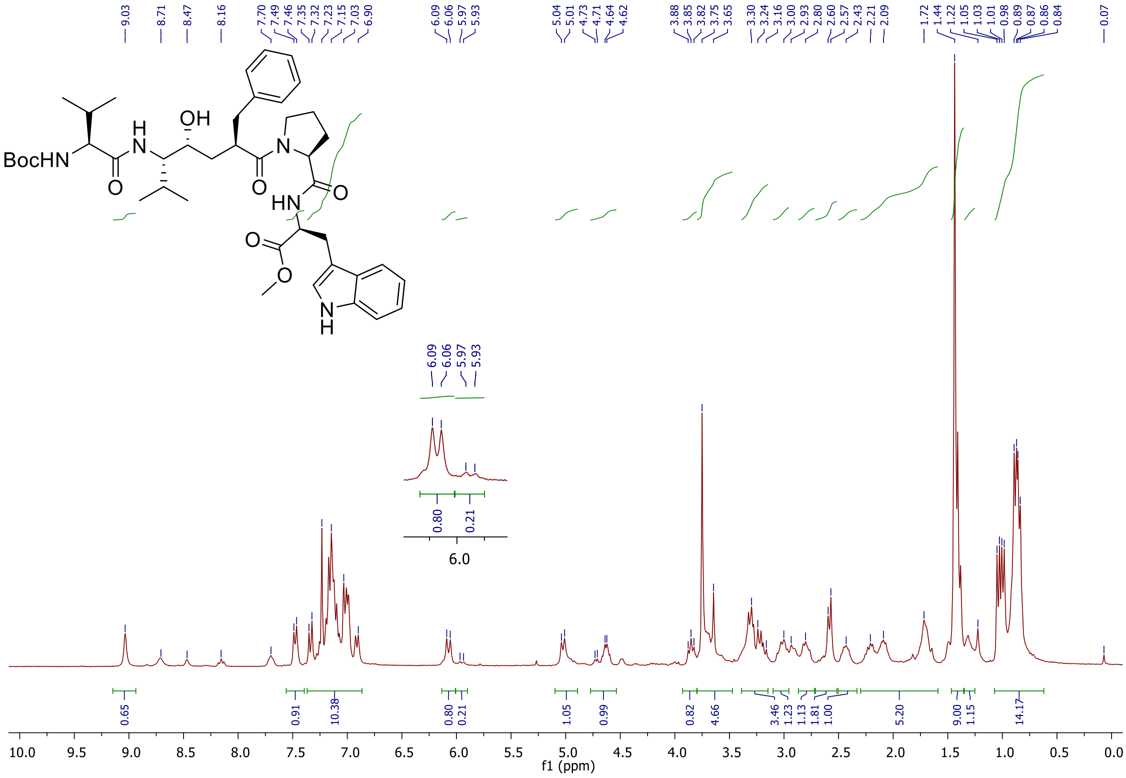

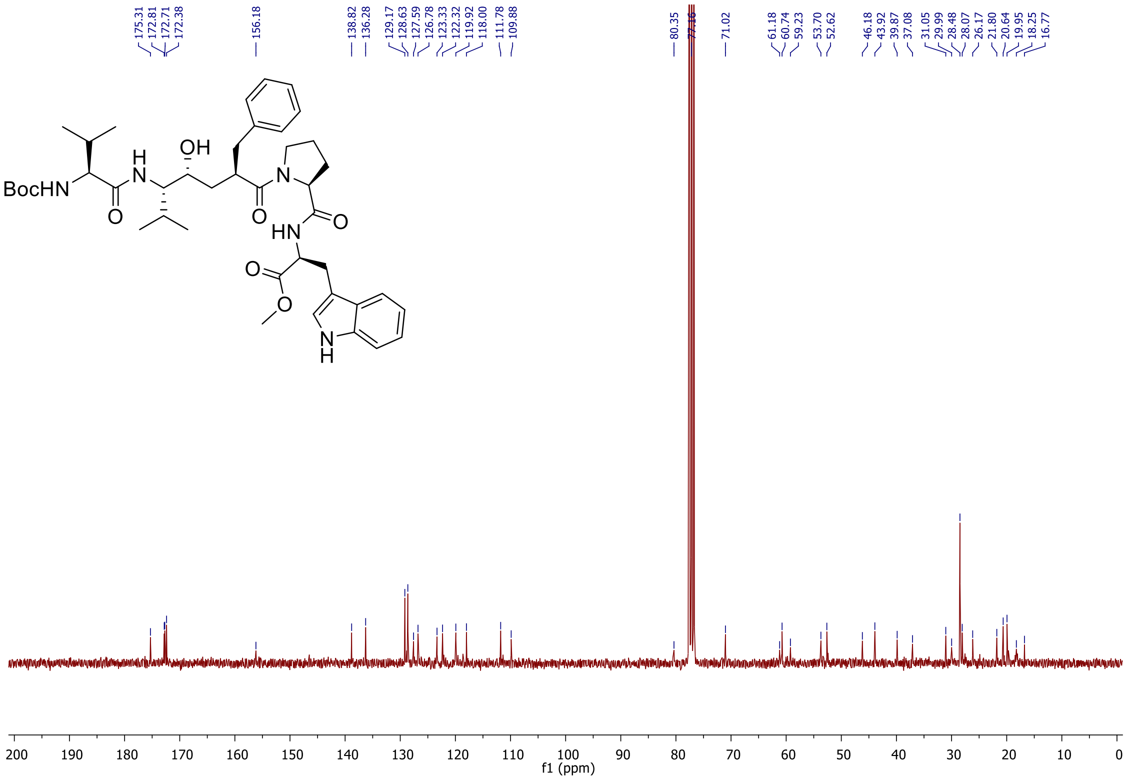

^{1}H and ^{13}C NMR spectra of ((2*R*,4*R*,5*S*)-5-((*S*)-2-Ammonio-3-methylbutanamido)-2-benzyl-4-hydroxy-6-methylheptanoyl)-L-prolyl-L-tryptophanate (*HER*) (23)

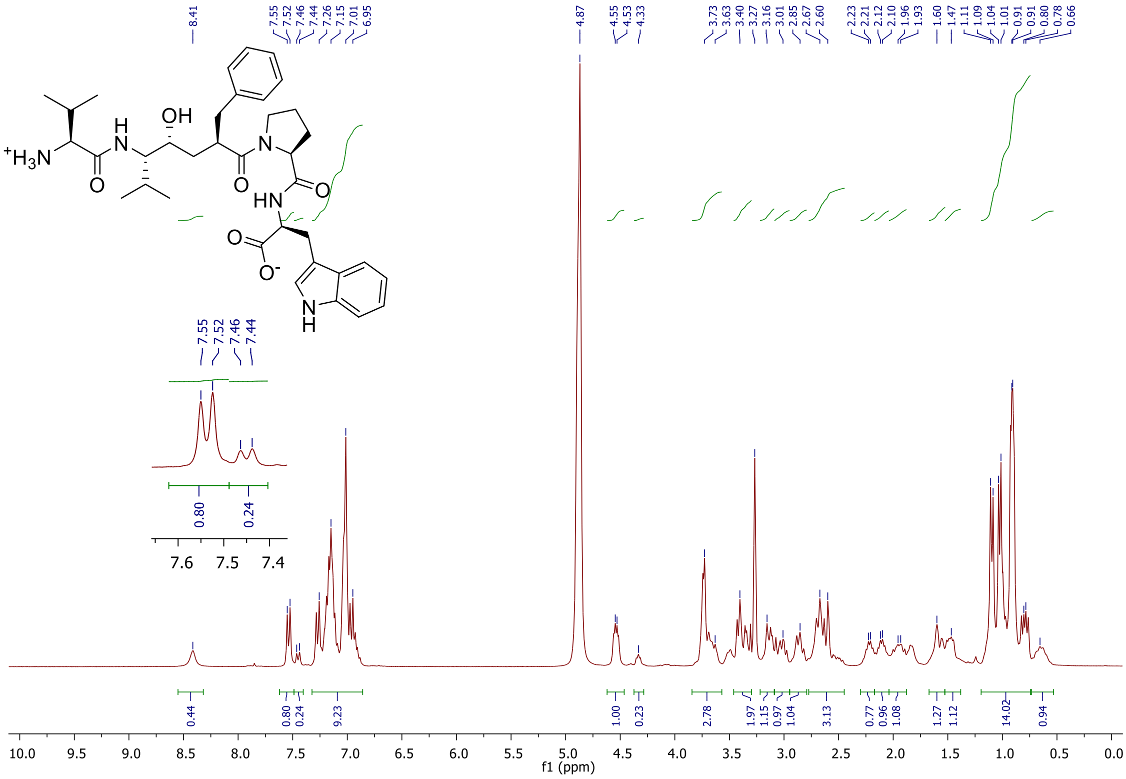

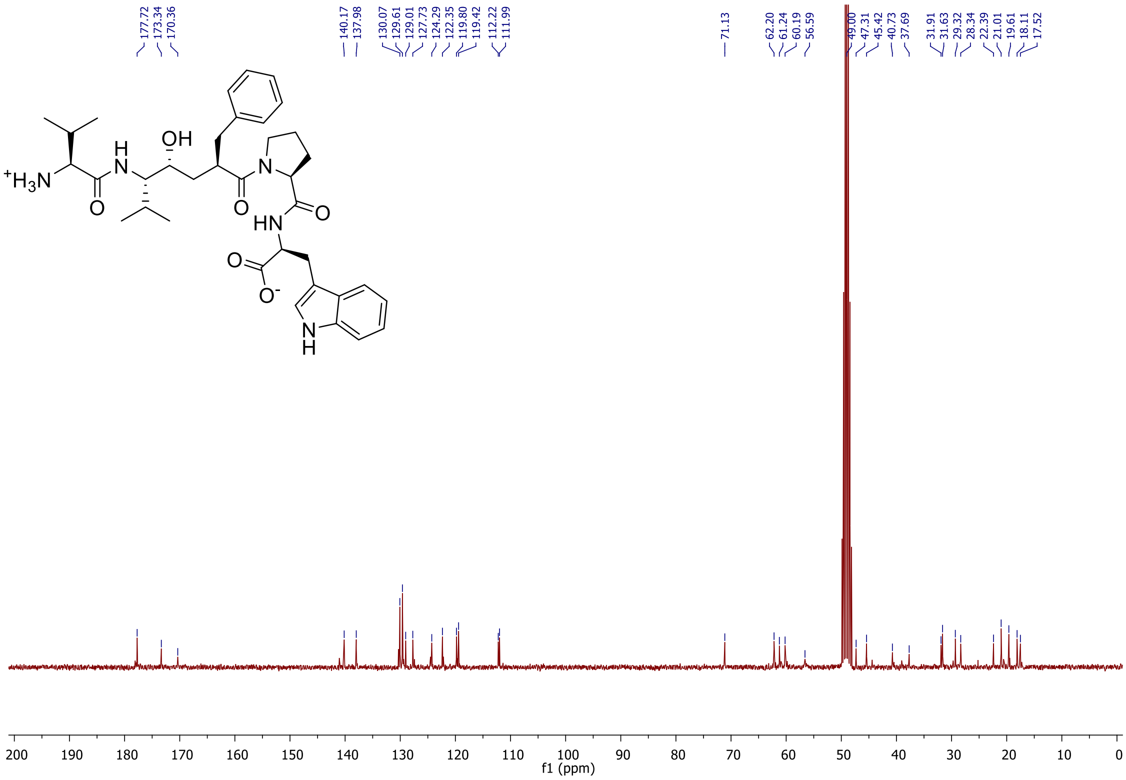

^{1}H and ^{13}C spectra of 4-((*tert*-butyldimethylsilyl)oxy)benzaldehyde

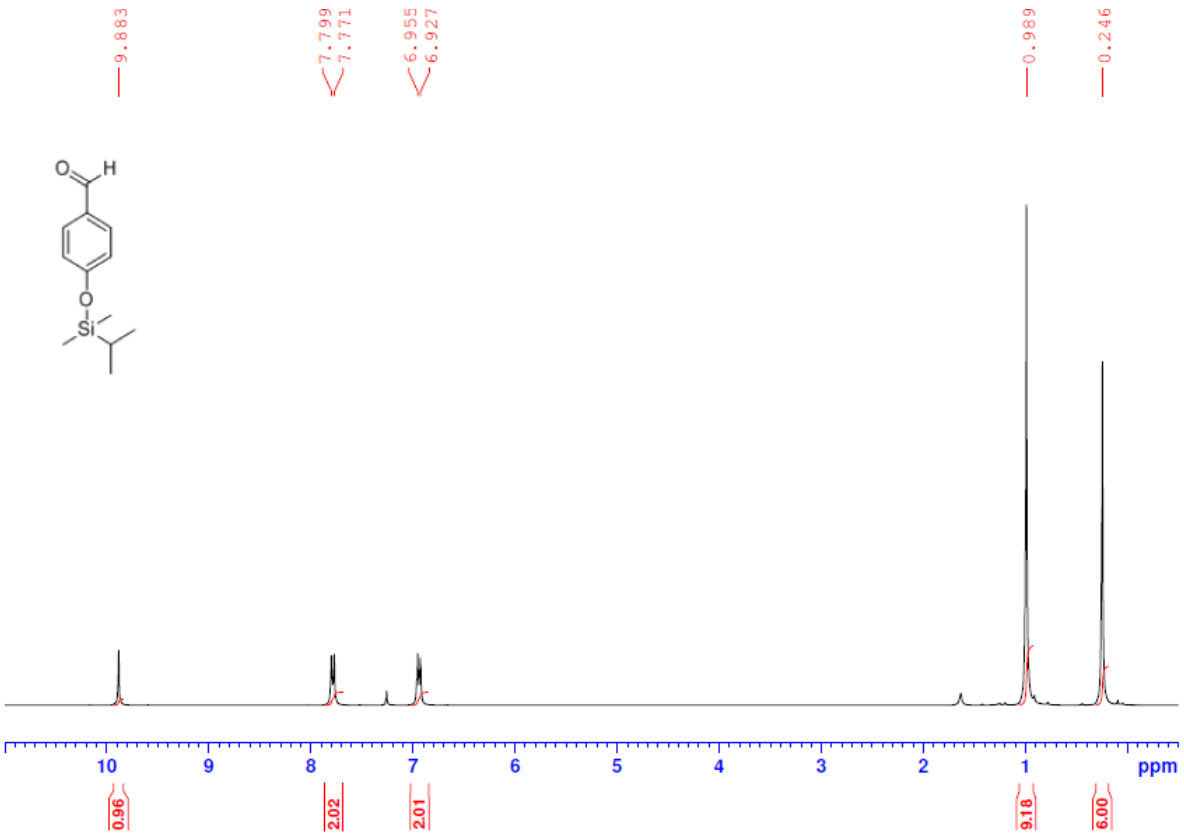

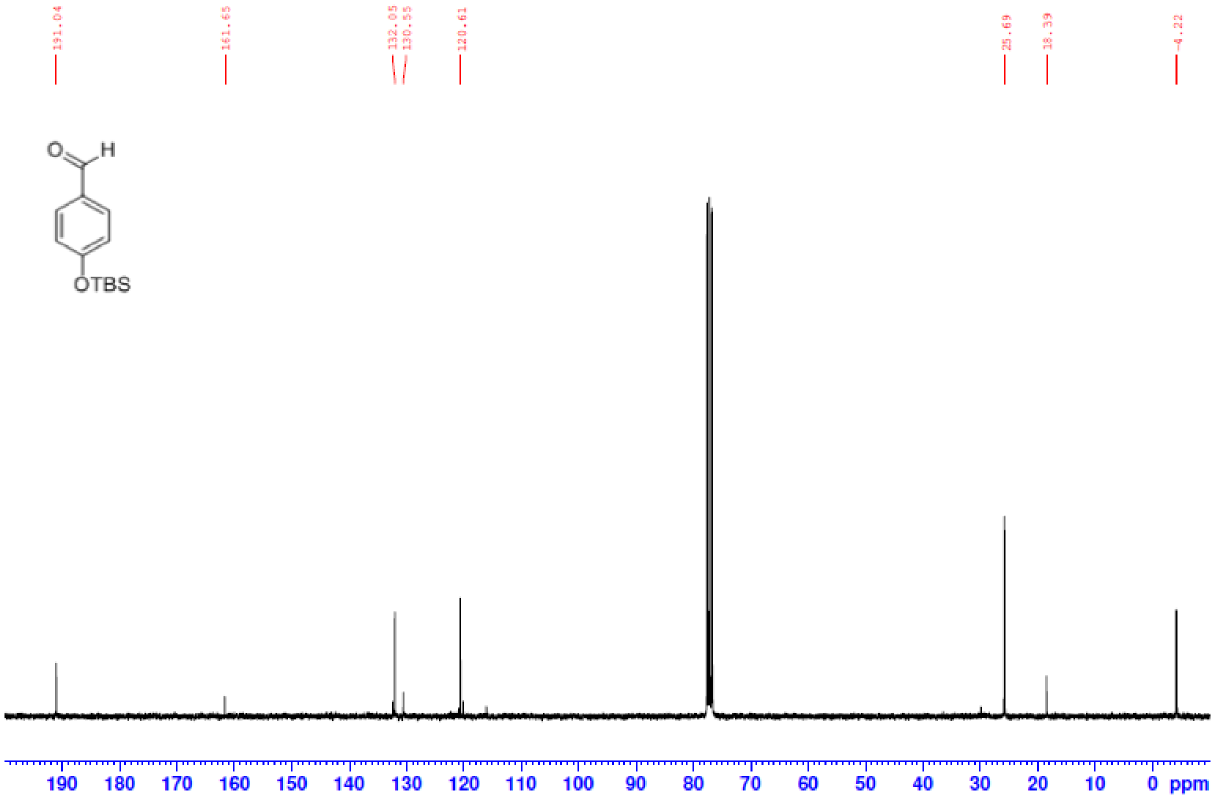

^{1}H and ^{19}F spectra of *tert*-Butyl ((1*S*)-1-((2*R*)-4-((4-fluorophenyl)(hydroxy)methyl)-5-oxotetrahydrofuran-2-yl)-2-methylpropyl)carbamate (24a) *(for each of the two diastereomers)*

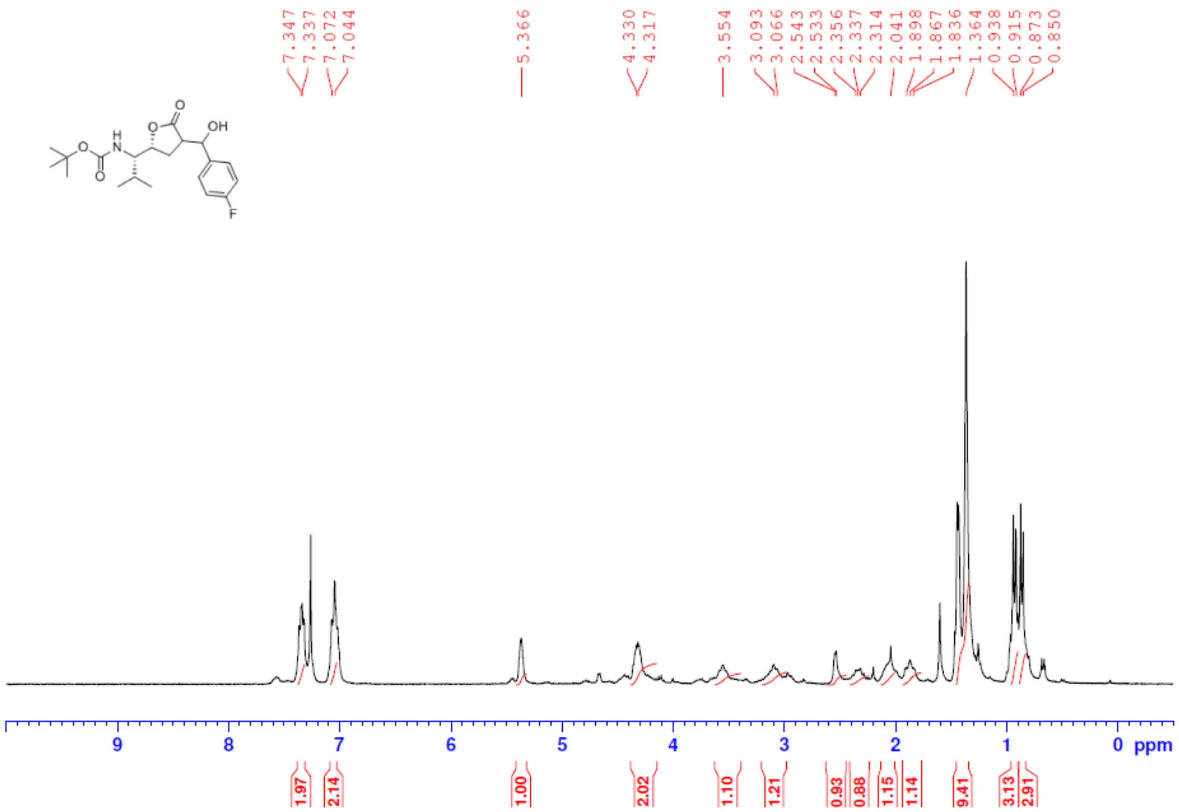

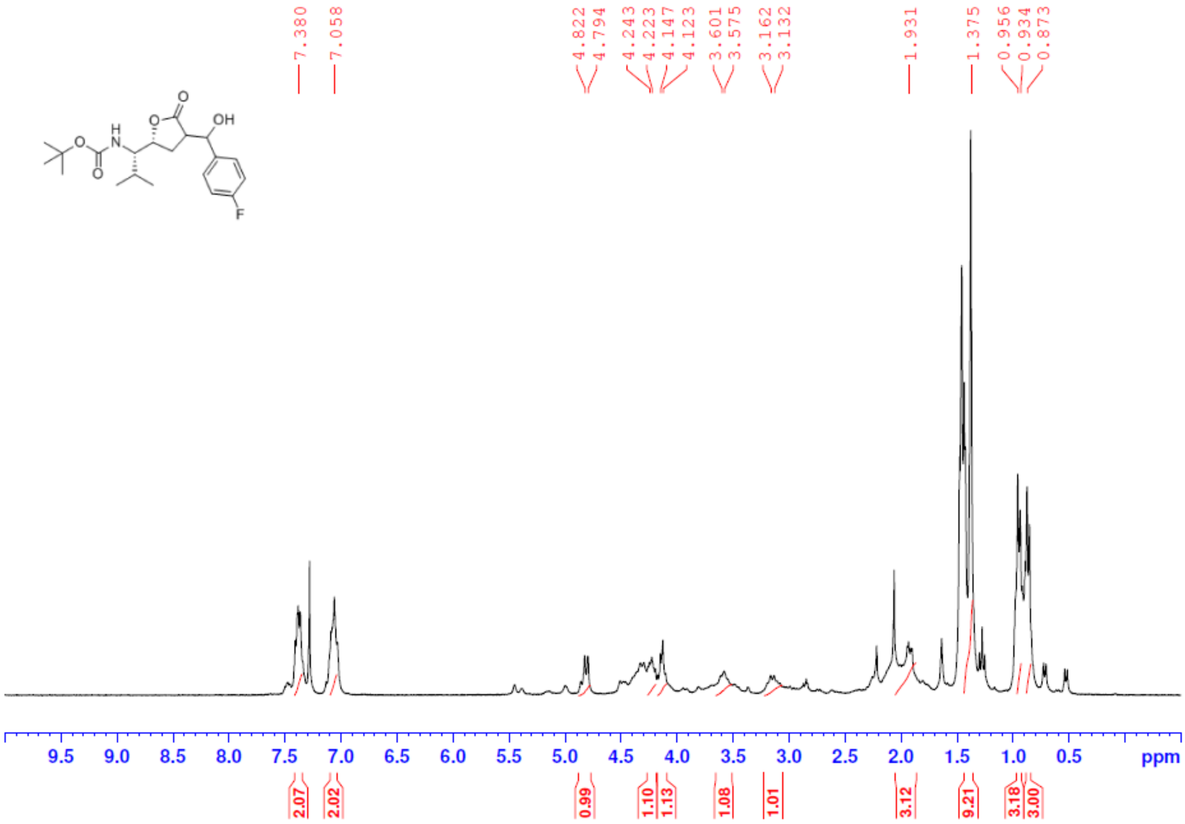

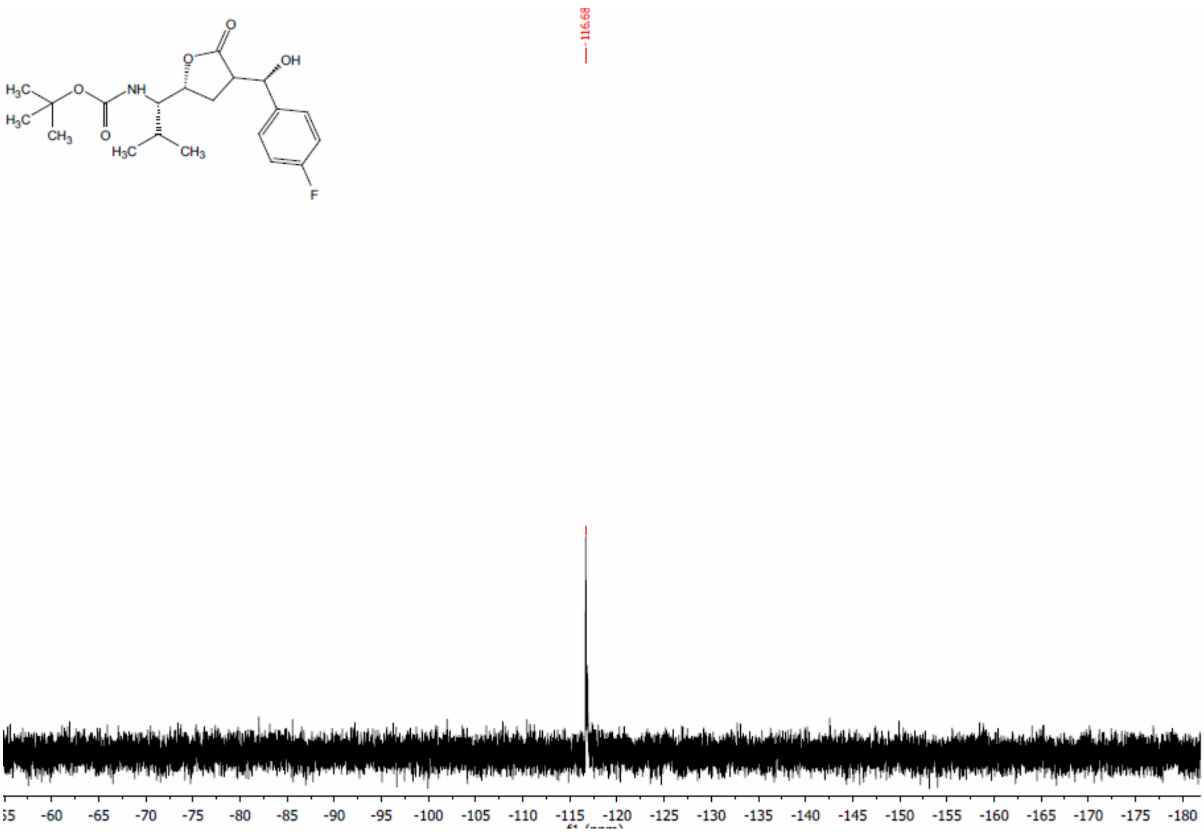
-116.68
H3C
H3C
CH3
O
NH
O
OH
H3C
CH3
F
-60 -65 -70 -75 -80 -85 -90 -95 -100 -105 -110 -115 -120 -125 -130 -135 -140 -145 -150 -155 -160 -165 -170 -175 -180

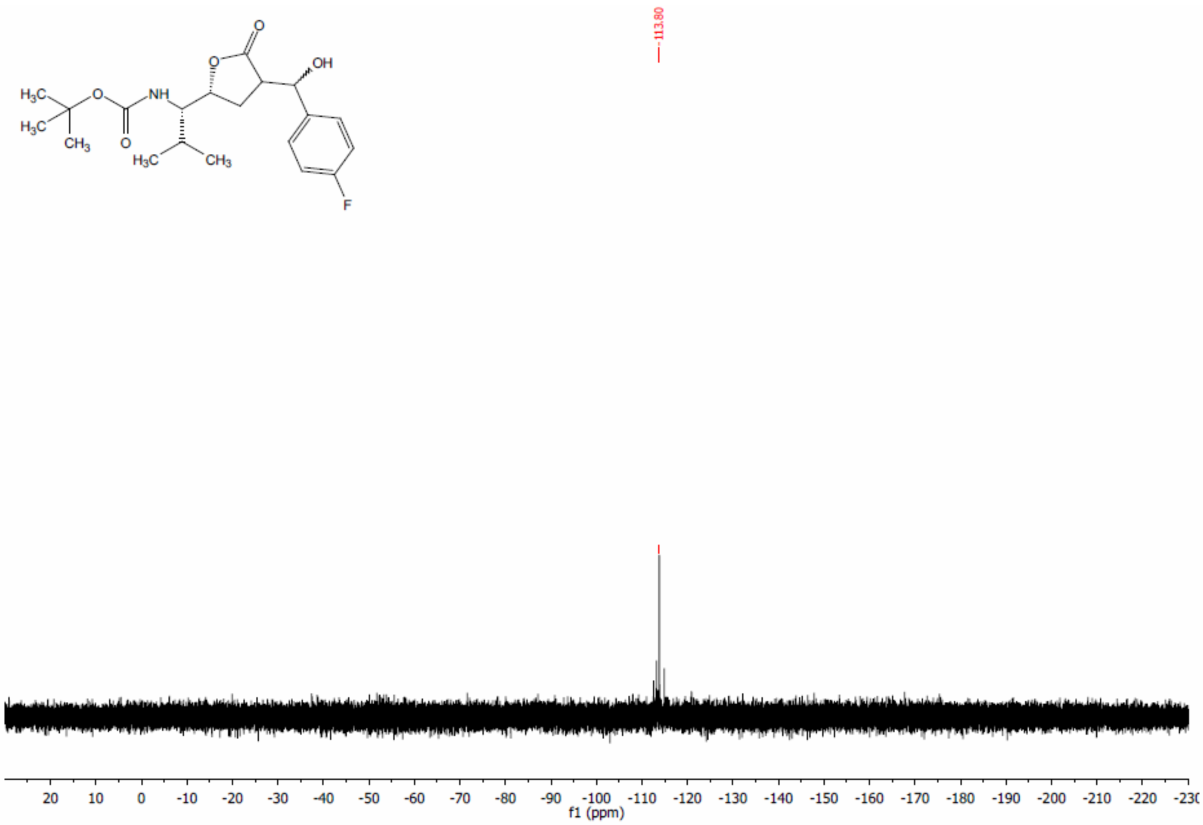
-113.80
H3C
H3C
CH3
O
NH
O
OH
H3C
CH3
F
20 10 0 -10 -20 -30 -40 -50 -60 -70 -80 -90 -100 -110 -120 -130 -140 -150 -160 -170 -180 -190 -200 -210 -220
f1 (ppm)

^{1}H, ^{13}C and ^{19}F spectra of *tert*-butyl ((*S*)-1-((2*R*, 4*R*)-4-(4-fluorobenzyl)-5-oxotetrahydrofuran-2-yl)-2-methylpropyl)carbamate (25a)

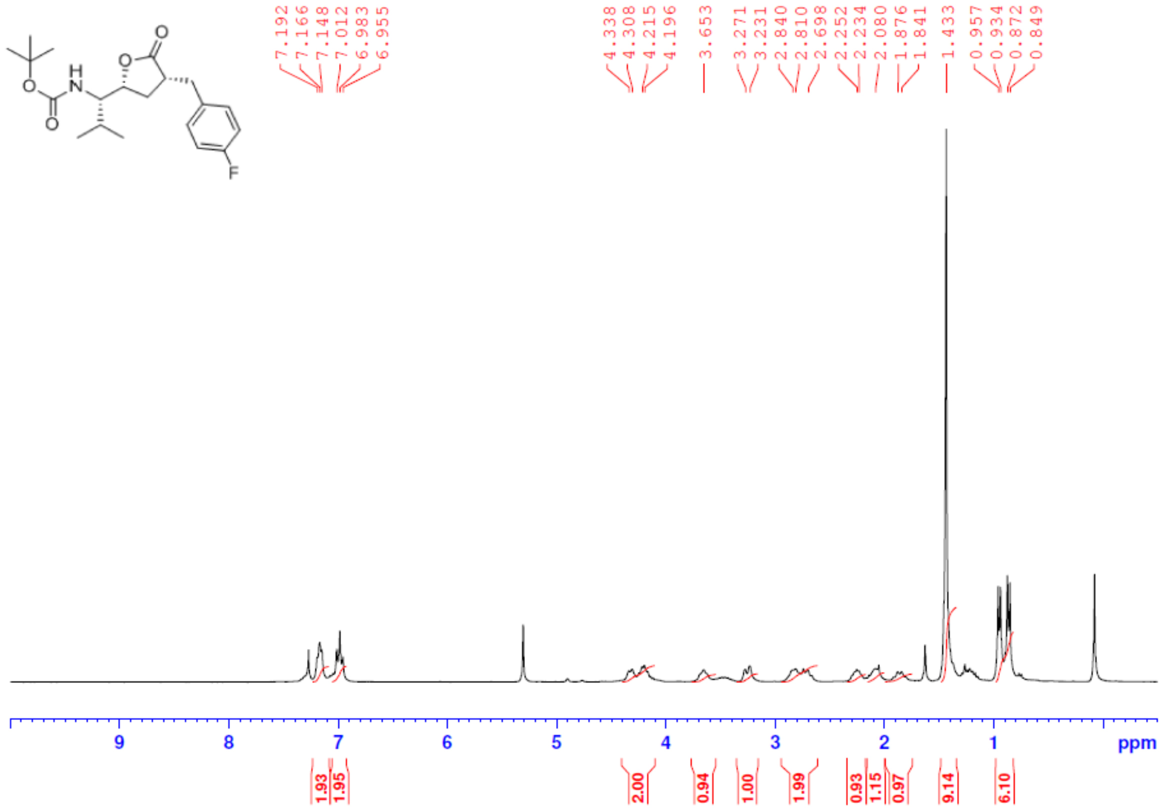

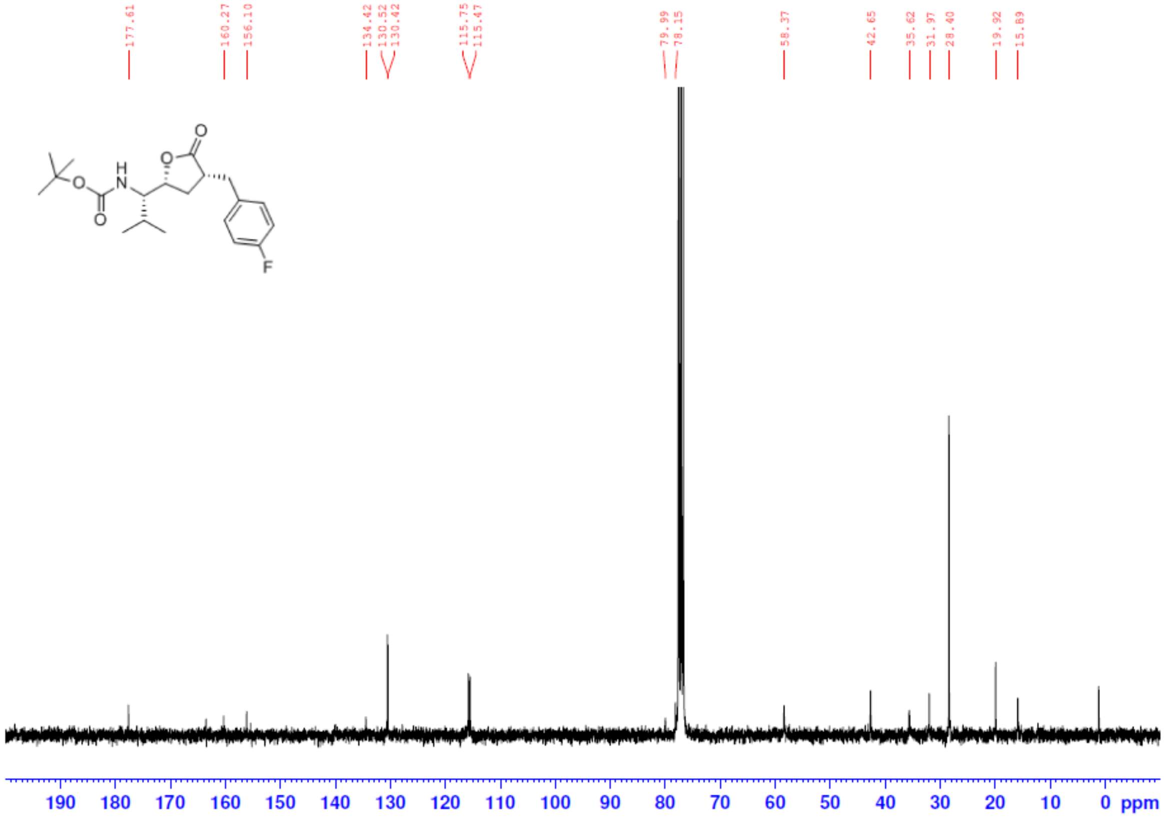
177.61
160.27
156.10
134.42
130.52
130.42
115.75
115.47
79.99
78.15
58.37
42.65
35.62
31.97
28.40
19.92
15.89
ppm

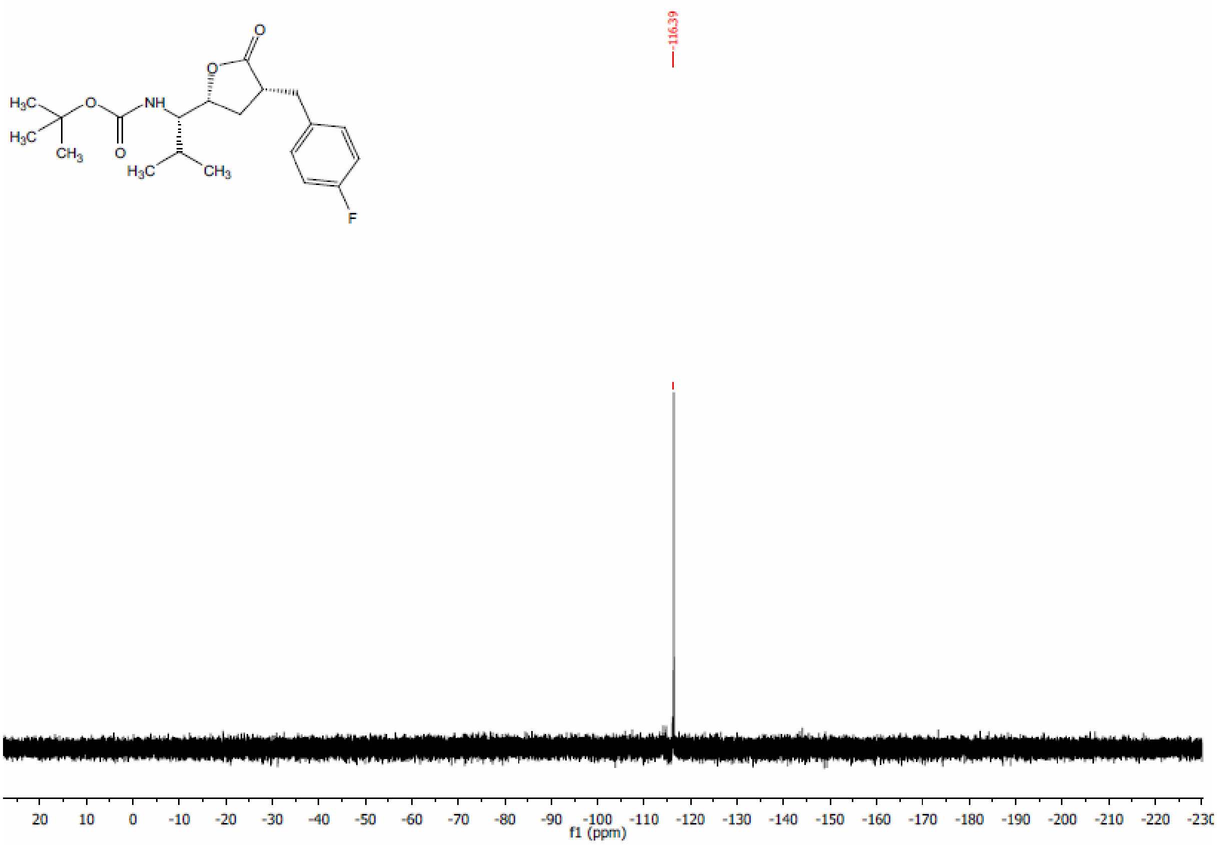
-116.39
f1 (ppm)

^{1}H, ^{13}C and ^{19}F spectra of (2*R*,4*R*,5*S*)-5-((*tert*-Butoxycarbonyl)amino)-4-((*tert*-butyldimethylsilyl)oxy)-2-(4-fluorobenzyl)-6-methylheptanoic acid (27a)

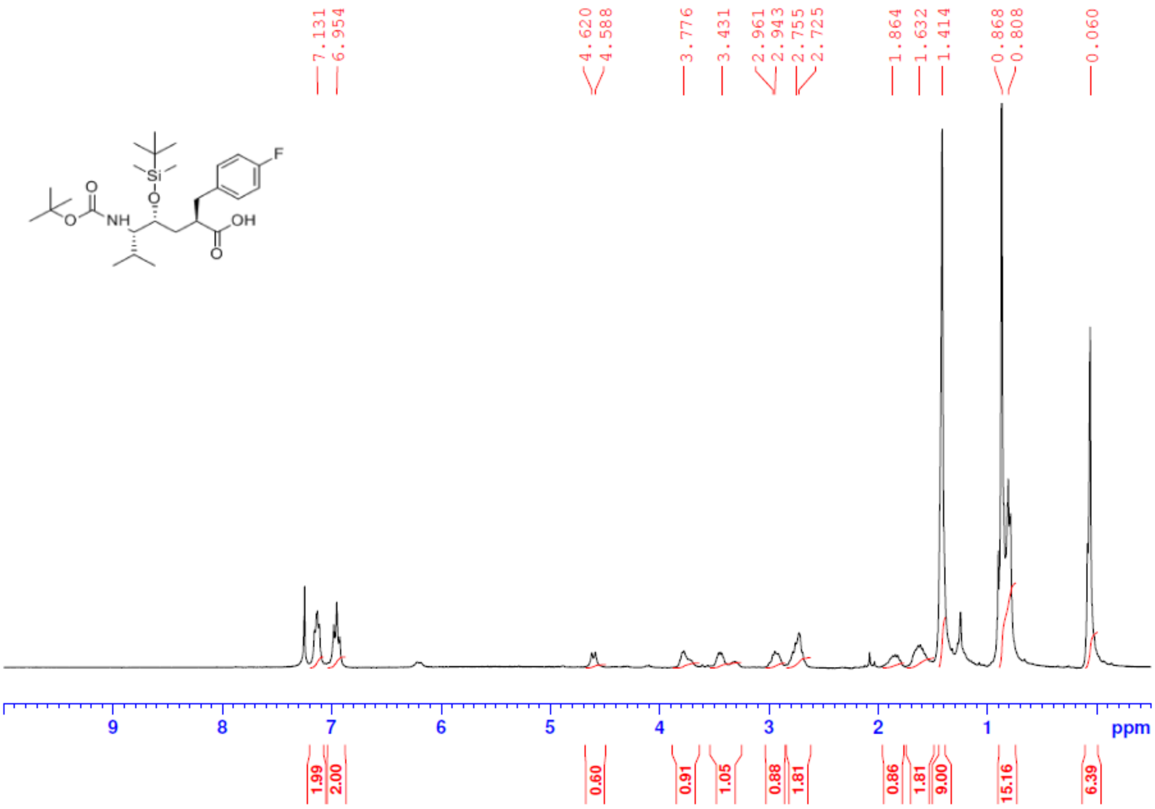

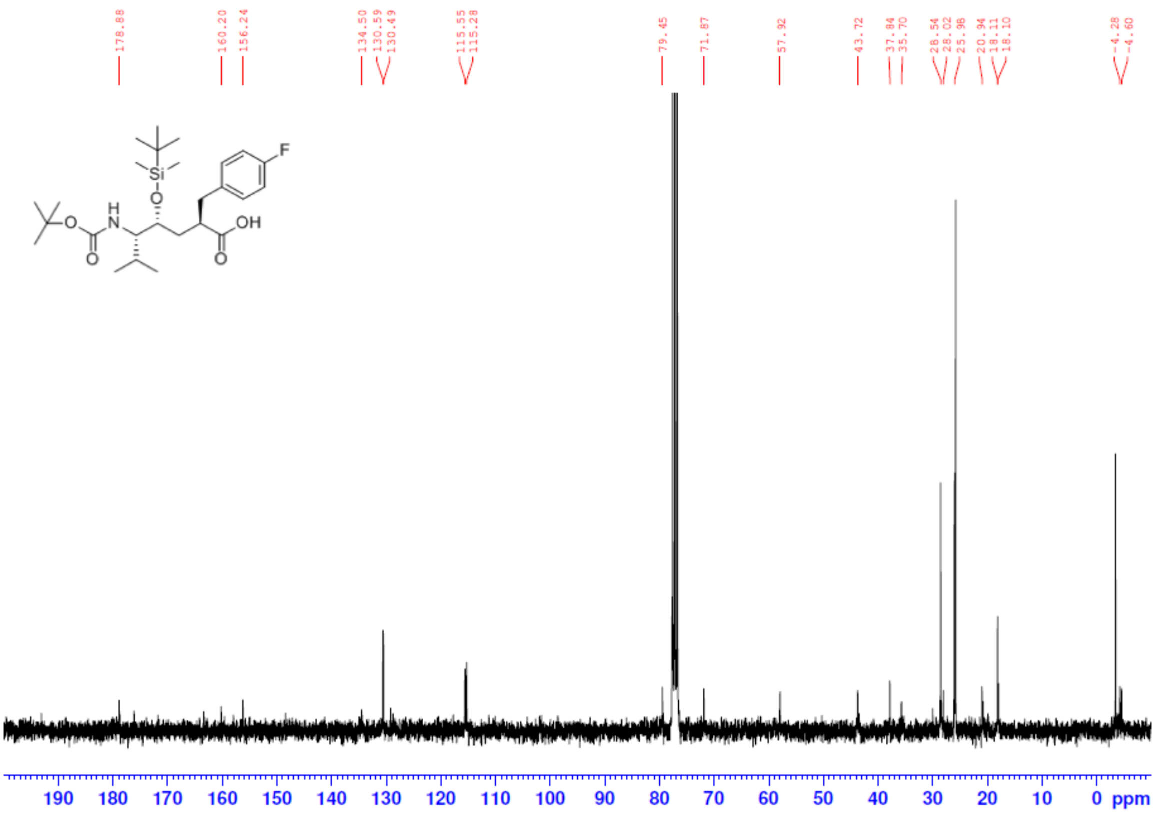
178.88
160.20
156.24
134.50
130.59
130.49
115.55
115.28
79.45
71.87
57.92
43.72
37.84
35.70
28.54
28.02
25.98
20.94
18.11
18.10
-4.28
-4.60
190 180 170 160 150 140 130 120 110 100 90 80 70 60 50 40 30 20 10 0 ppm

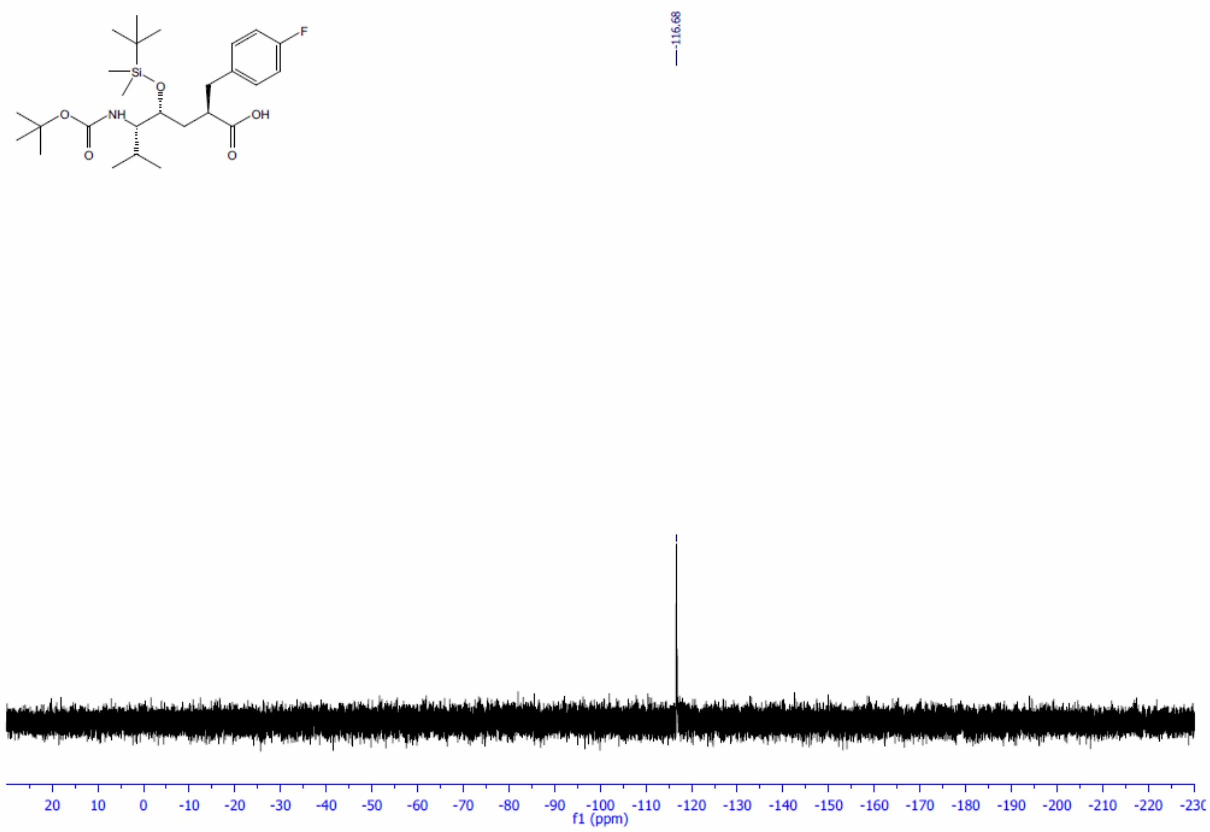
-116.68
20 10 0 -10 -20 -30 -40 -50 -60 -70 -80 -90 -100 -110 -120 -130 -140 -150 -160 -170 -180 -190 -200 -210 -220 -230
f1 (ppm)

^{1}H, ^{13}C and ^{19}F spectra of methyl ((2*R*, 4*R*, 5*S*)-5-((*tert*-butoxycarbonyl)amino)-4-((*tert*-butyldimethylsilyl)oxy)-2-(4-fluorobenzyl)-6-methylheptanoyl)-L-prolyl-L-tryptophanate (28a)

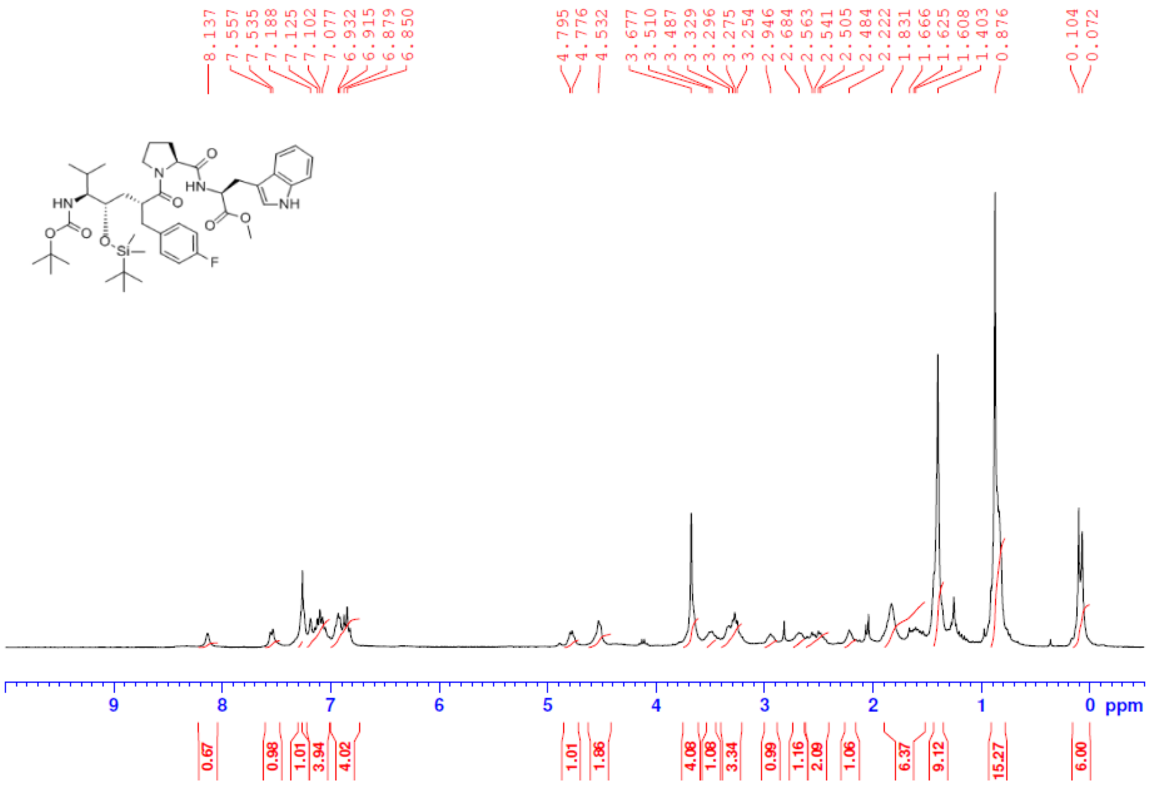

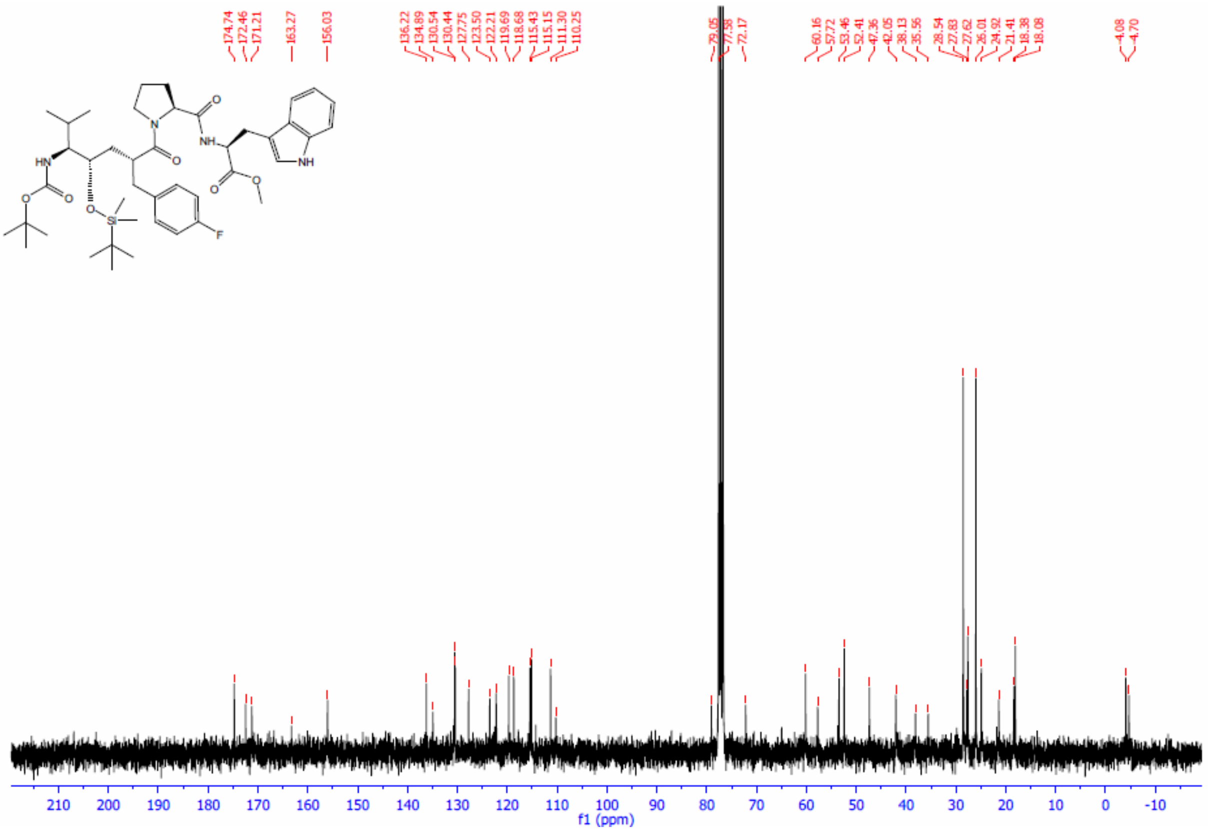

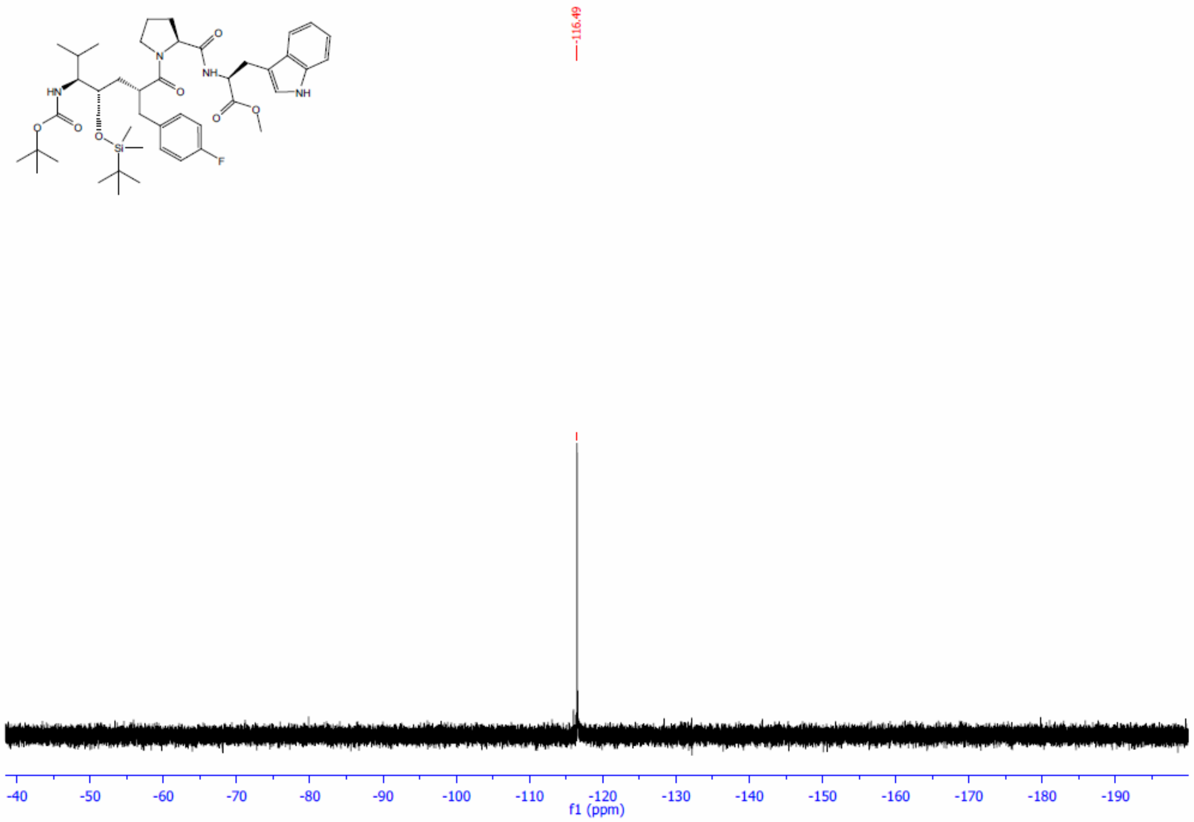

^{1}H, ^{13}C and ^{19}F spectra of methyl ((2*R*,4*R*,5*S*)-5-((*S*)-2-((*tert*-butoxycarbonyl)amino)-3-methylbutanamido)-2-(4-fluorobenzyl)-4-hydroxy-6-methylheptanoyl)-L-prolyl-L-tryptophanate (30a)

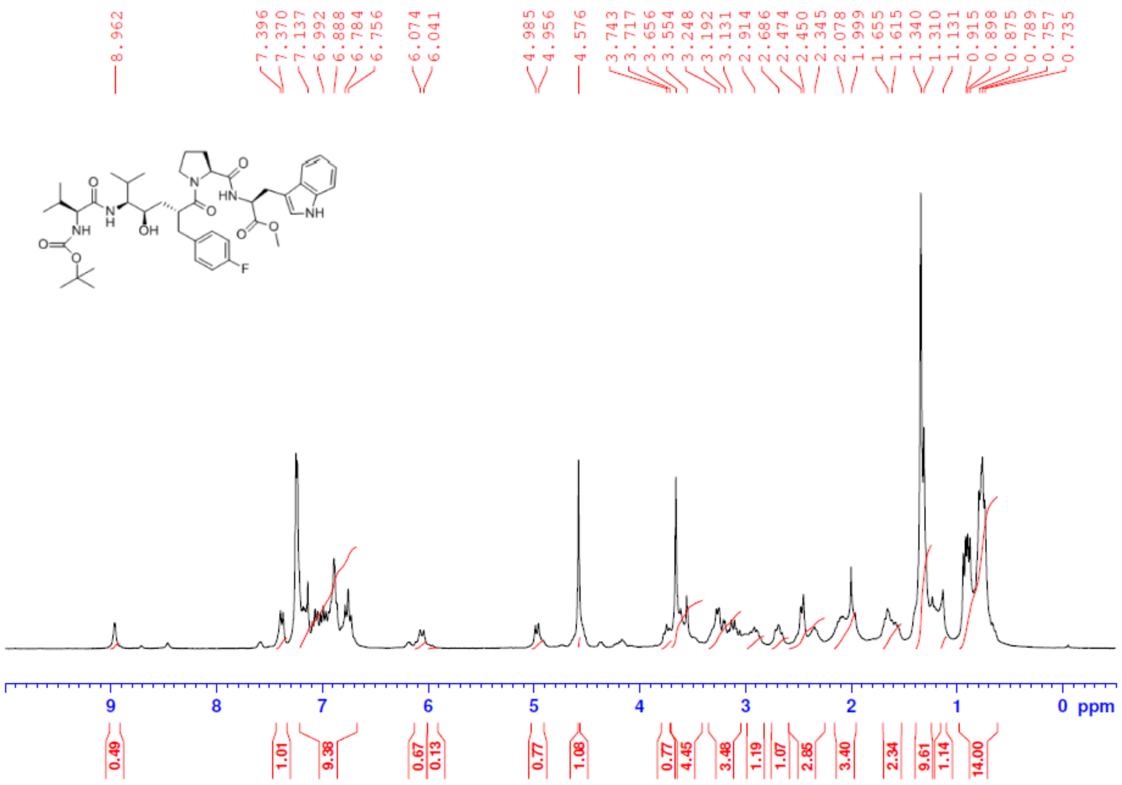

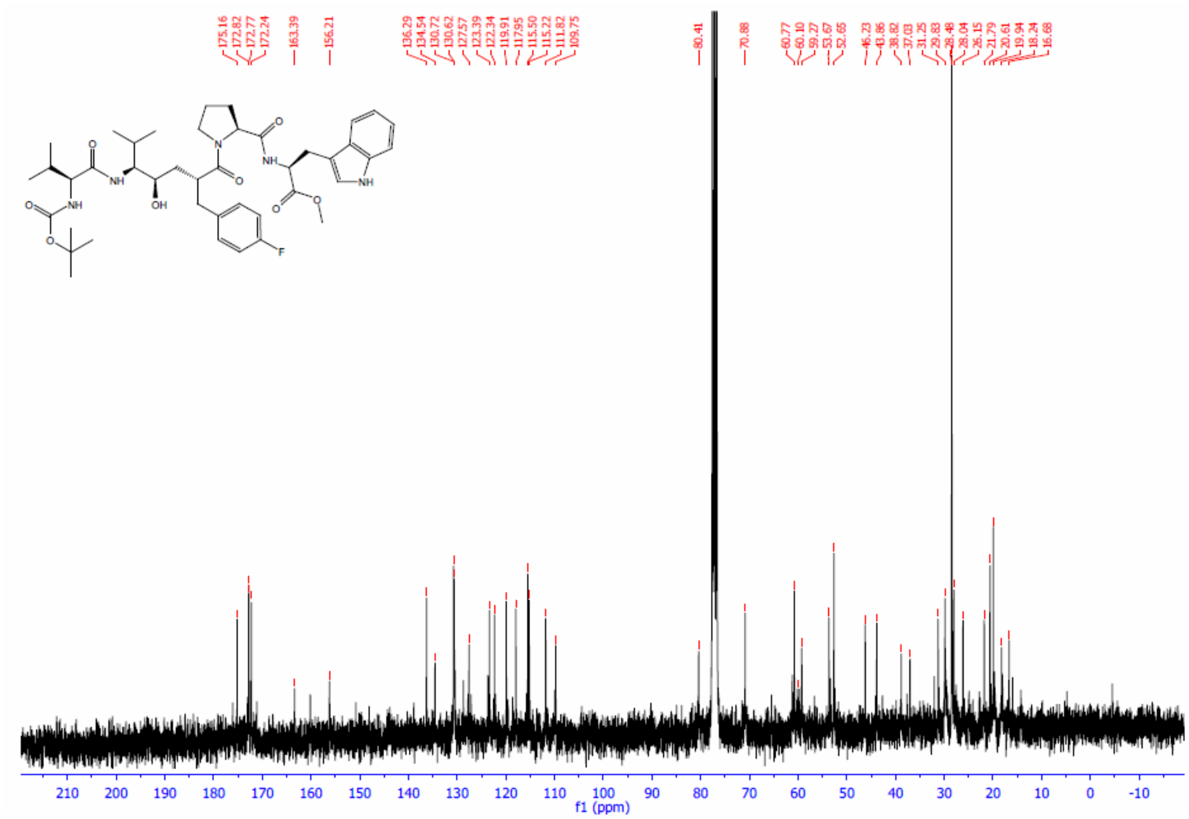

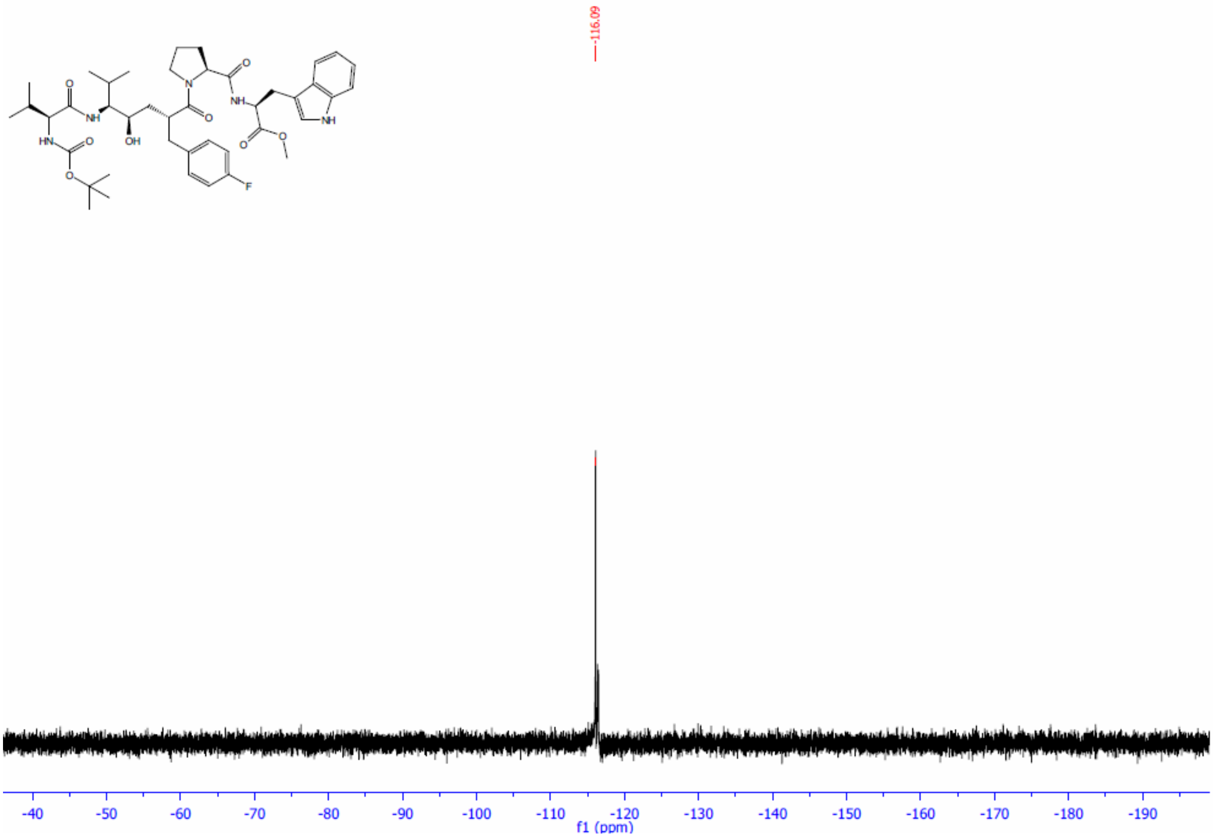

^{1}H, ^{13}C and ^{19}F spectra of ((2*R*,4*R*,5*S*)-5-((*S*)-2-ammonio-3-methylbutanamido)-2-(4-fluorobenzyl)-4-hydroxy-6-methylheptanoyl)-L-prolyl-L-tryptophanate (31a)

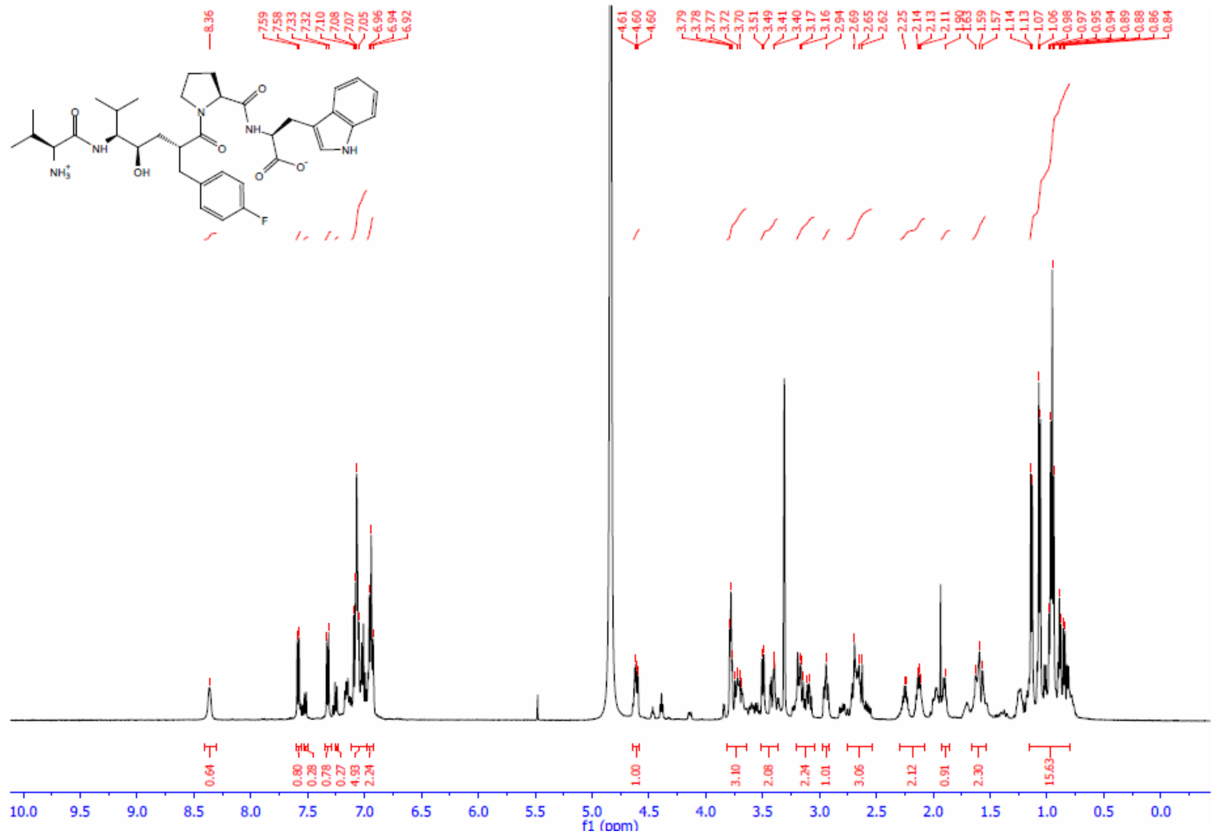

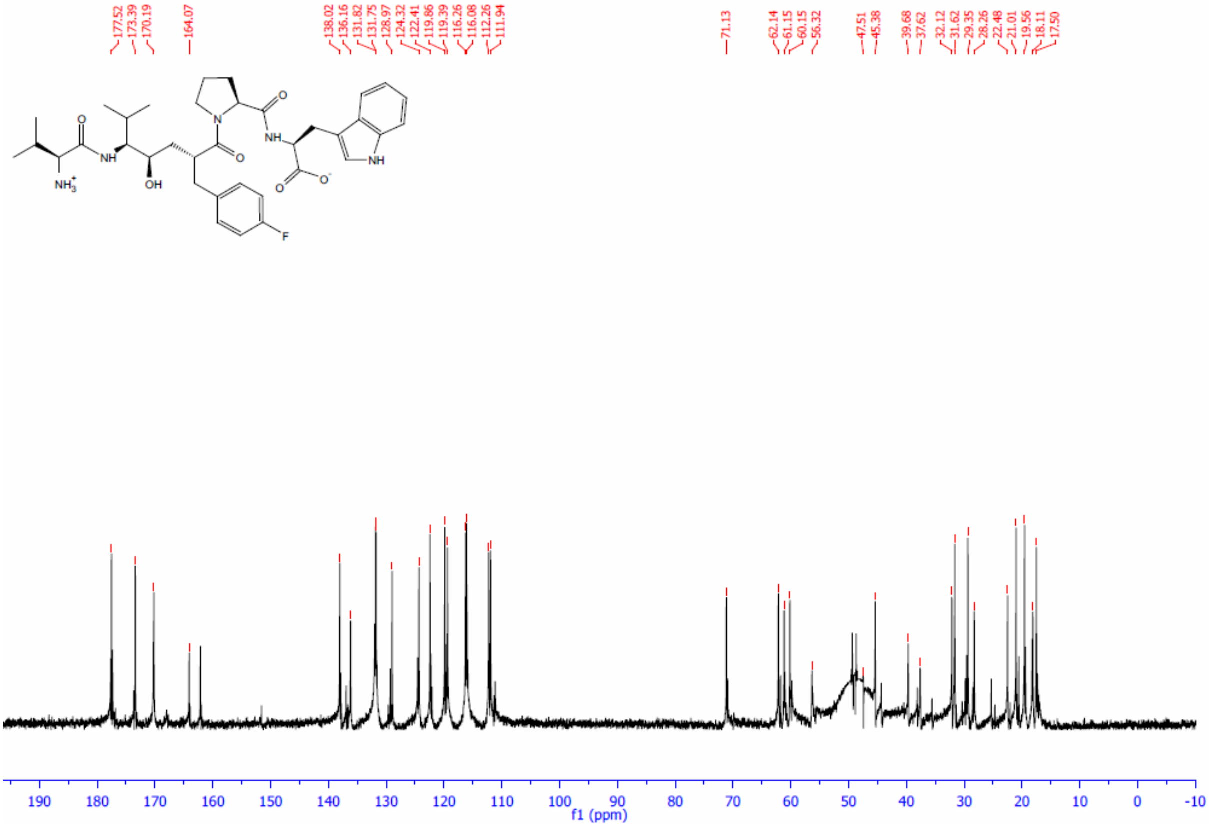

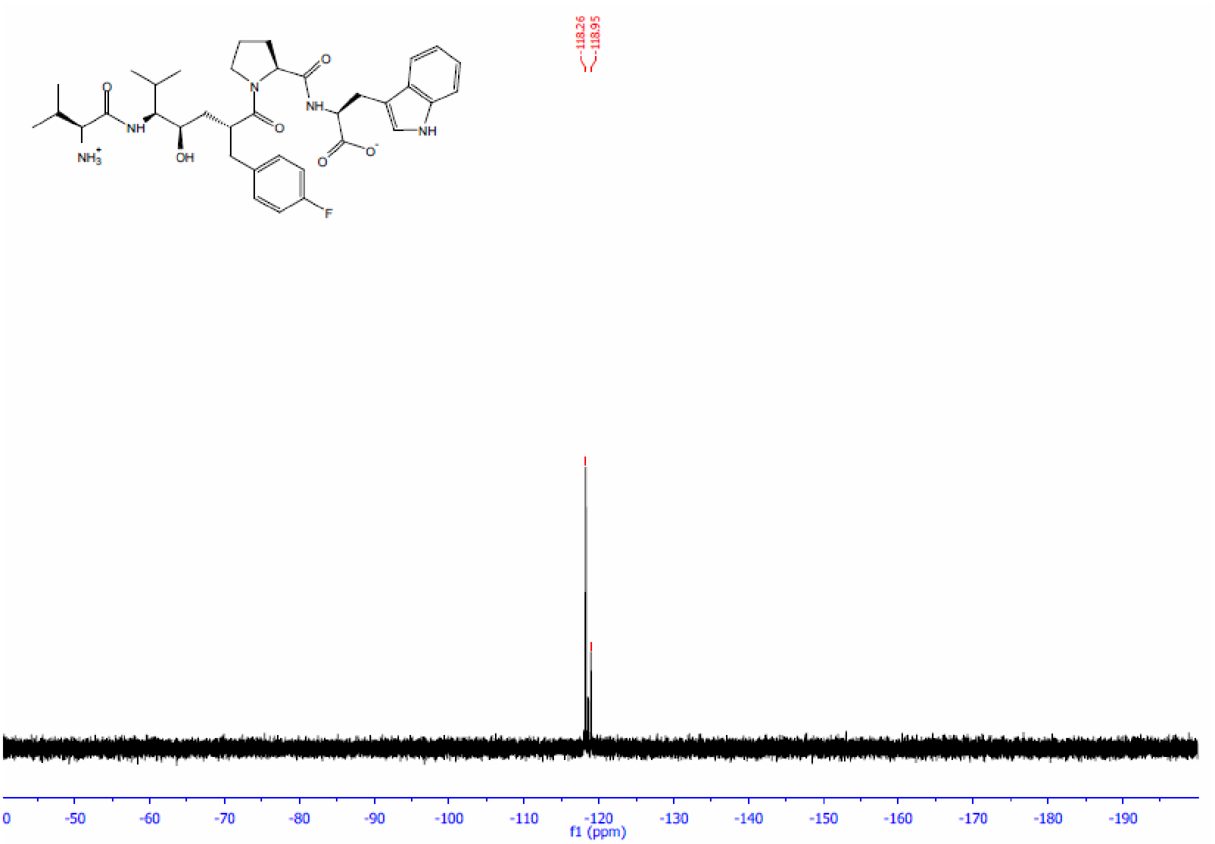

-118.26
-118.95
0
-50
-60
-70
-80
-90
-100
-110
-120
-130
-140
-150
-160
-170
-180
-190
f1 (ppm)

^{1}H and ^{13}C spectra of *tert*-butyl ((1*S*)-1-((2*R*)-4-(hydroxy(*p*-tolyl)methyl)-5-oxotetrahydrofuran-2-yl)-2-methylpropyl)carbamate (24b)

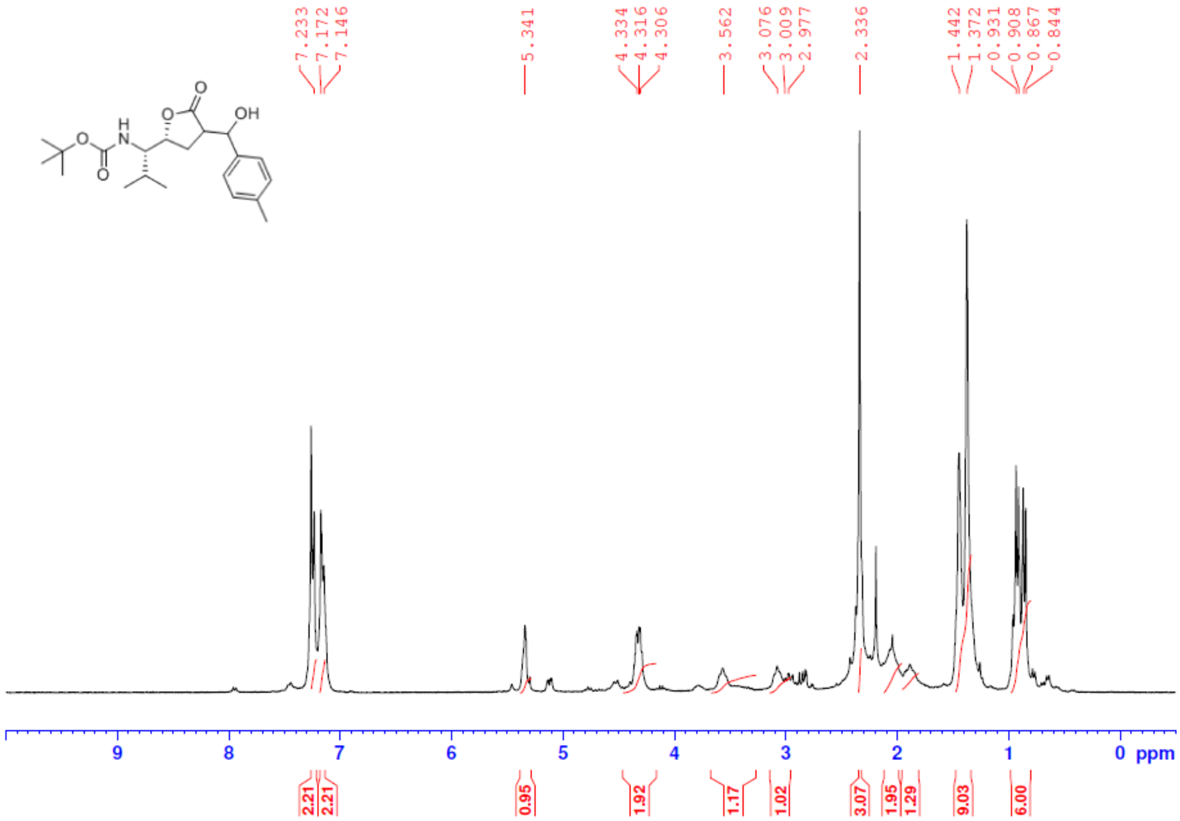

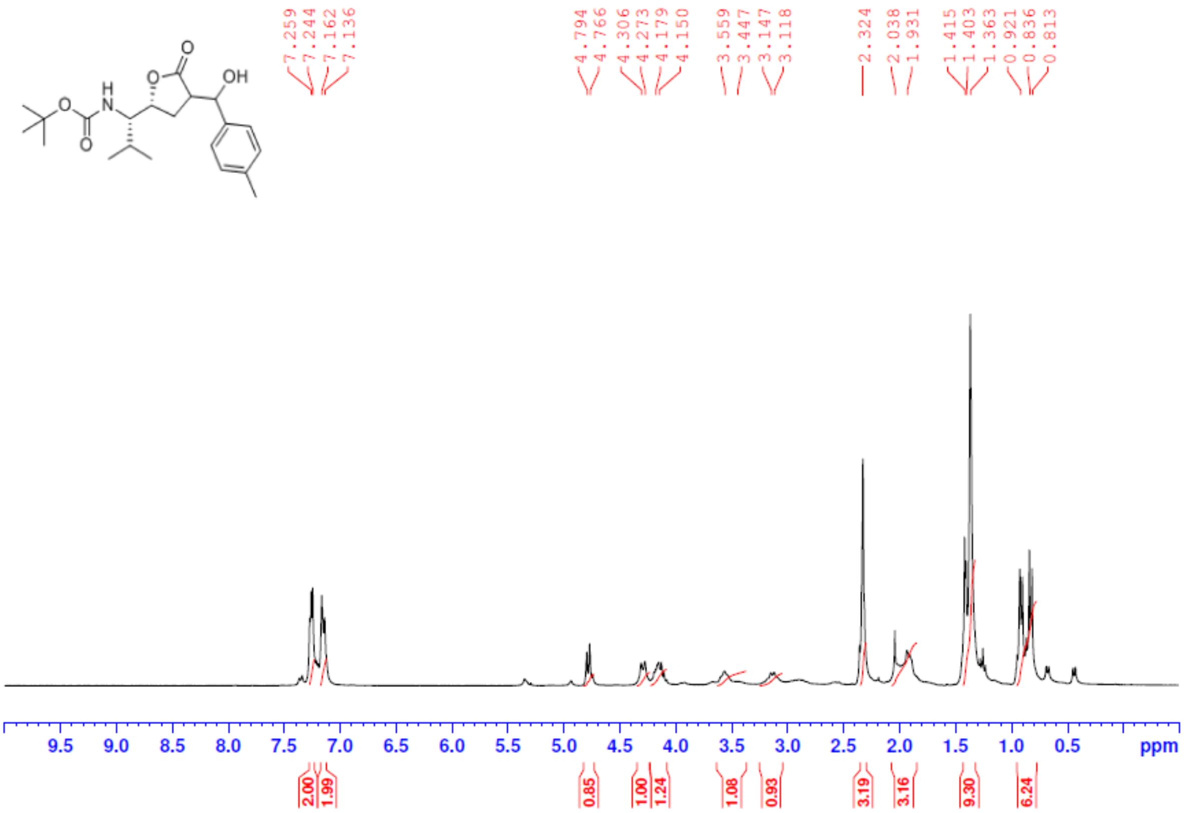

^{1}H and ^{13}C spectra of *tert*-butyl ((*S*)-2-methyl-1-((2*R*,4*R*)-4-(4-methylbenzyl)-5-oxotetrahydrofuran-2-yl)propyl)carbamate (25b)

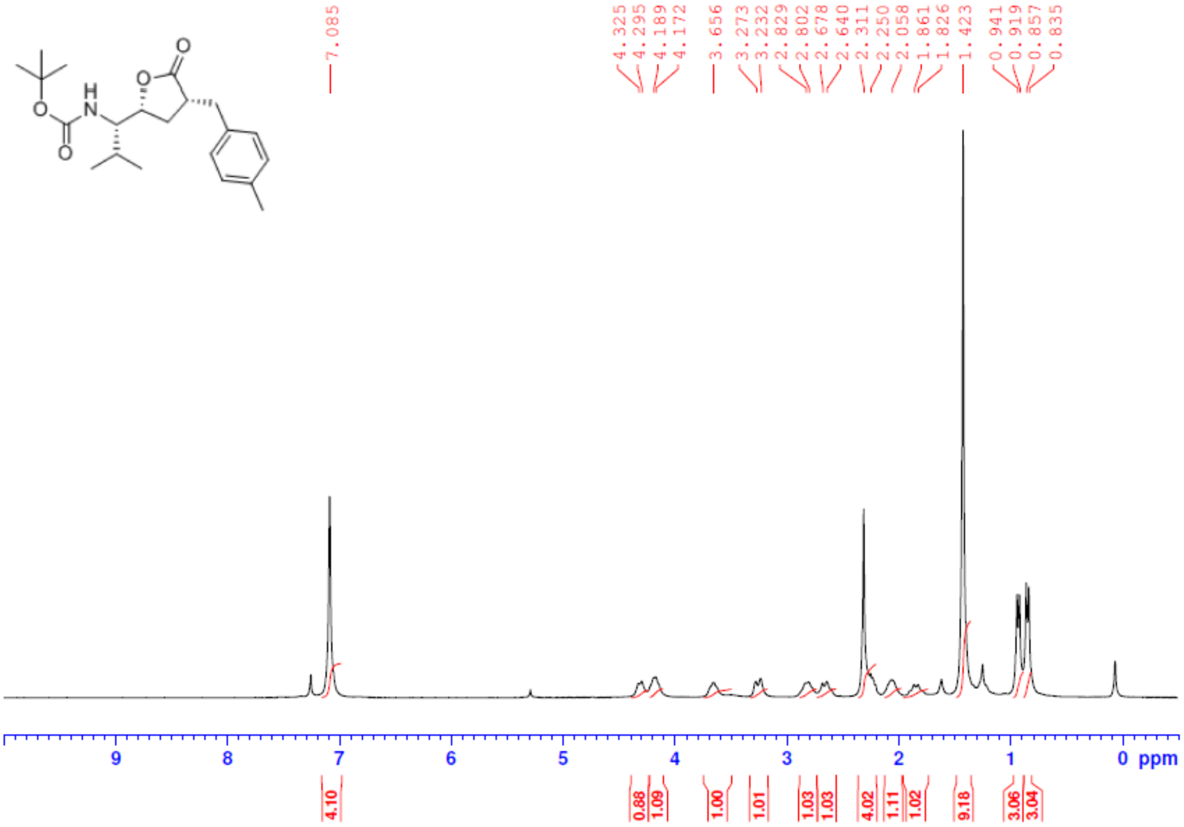

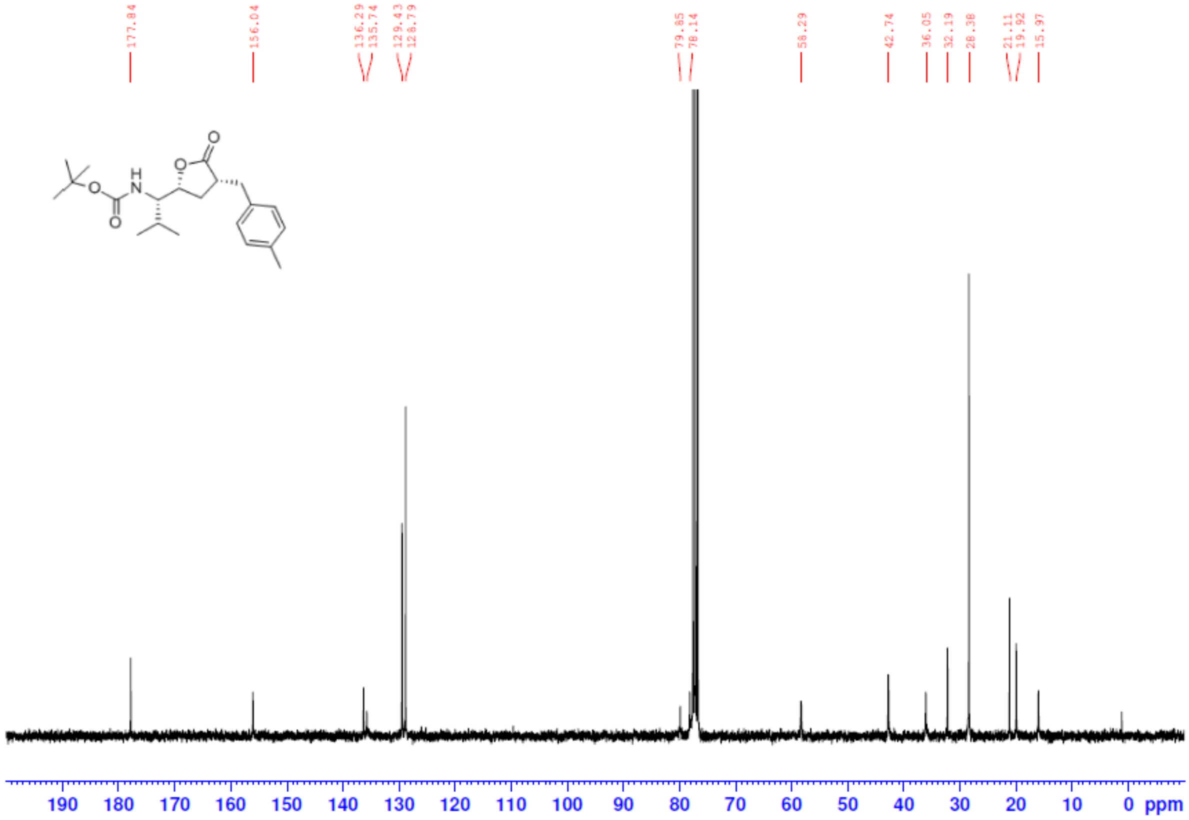

^{1}H and ^{13}C spectra of (2*R*,4*R*,5*S*)-5-((*tert*-butoxycarbonyl)amino)-4-((*tert*-butyldimethylsilyl)oxy)-6-methyl-2-(4-methylbenzyl)heptanoic acid (27b)

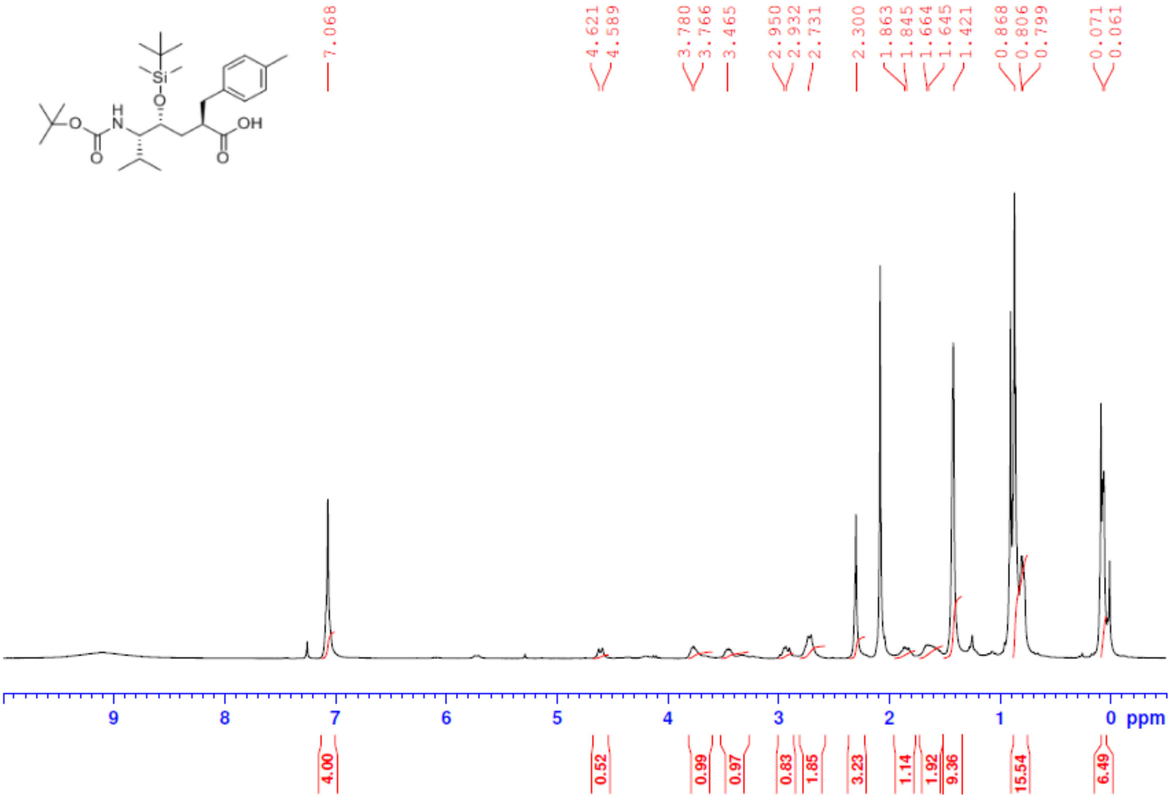
7.068
4.621
4.589
3.780
3.766
3.465
2.950
2.932
2.731
2.300
1.863
1.845
1.664
1.645
1.421
0.868
0.806
0.799
0.071
0.061
9
8
7
6
5
4
3
2
1
0 ppm
4.00
0.52
0.99
0.97
0.83
1.85
3.23
1.14
1.92
9.36
15.54
6.49

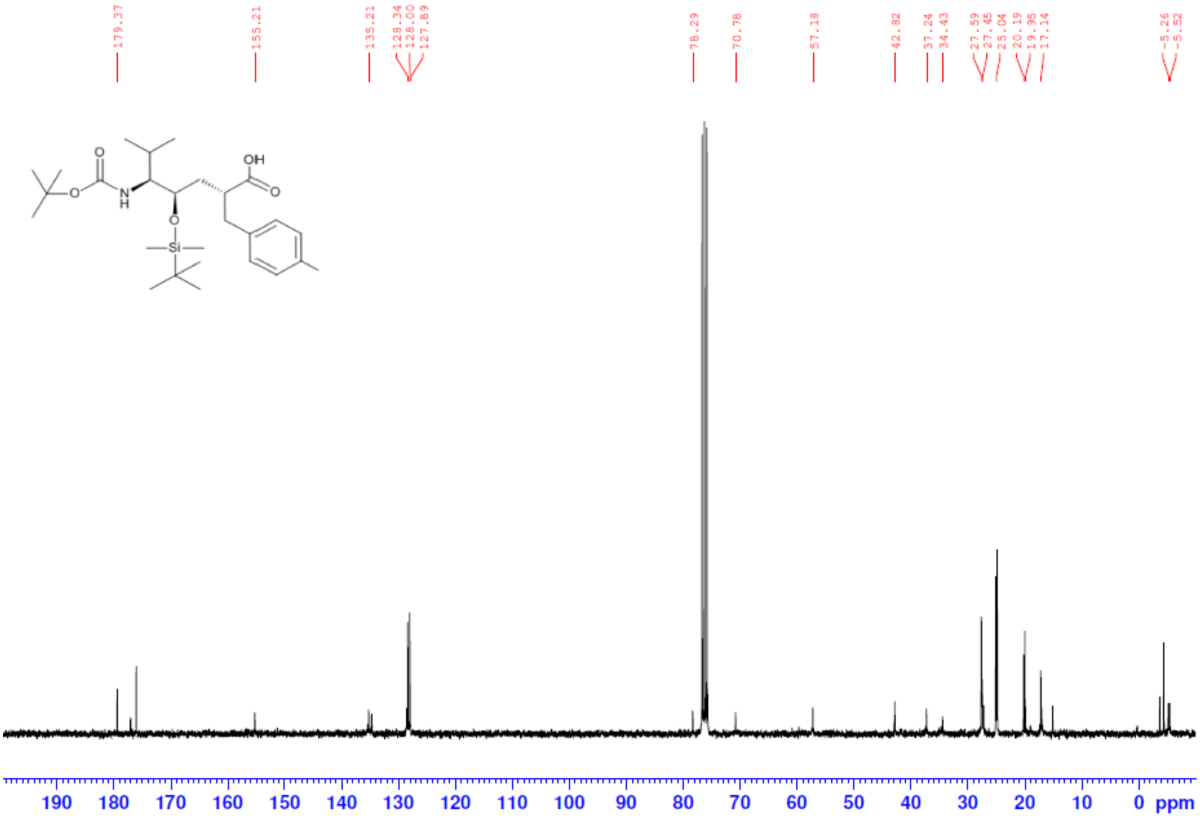
179.37
155.21
135.21
128.34
128.00
127.89
76.29
70.78
57.18
42.82
37.24
34.43
27.59
27.45
25.04
20.19
19.95
17.14
-5.26
-5.52
190
180
170
160
150
140
130
120
110
100
90
80
70
60
50
40
30
20
10
0 ppm

^{1}H and ^{13}C spectra of methyl ((2*R*,4*R*,5*S*)-5-((*tert*-butoxycarbonyl)amino)-4-((*tert*-butyldimethylsilyl)oxy)-6-methyl-2-(4-methylbenzyl)heptanoyl)-L-prolyl-L-tryptophanate (28b)

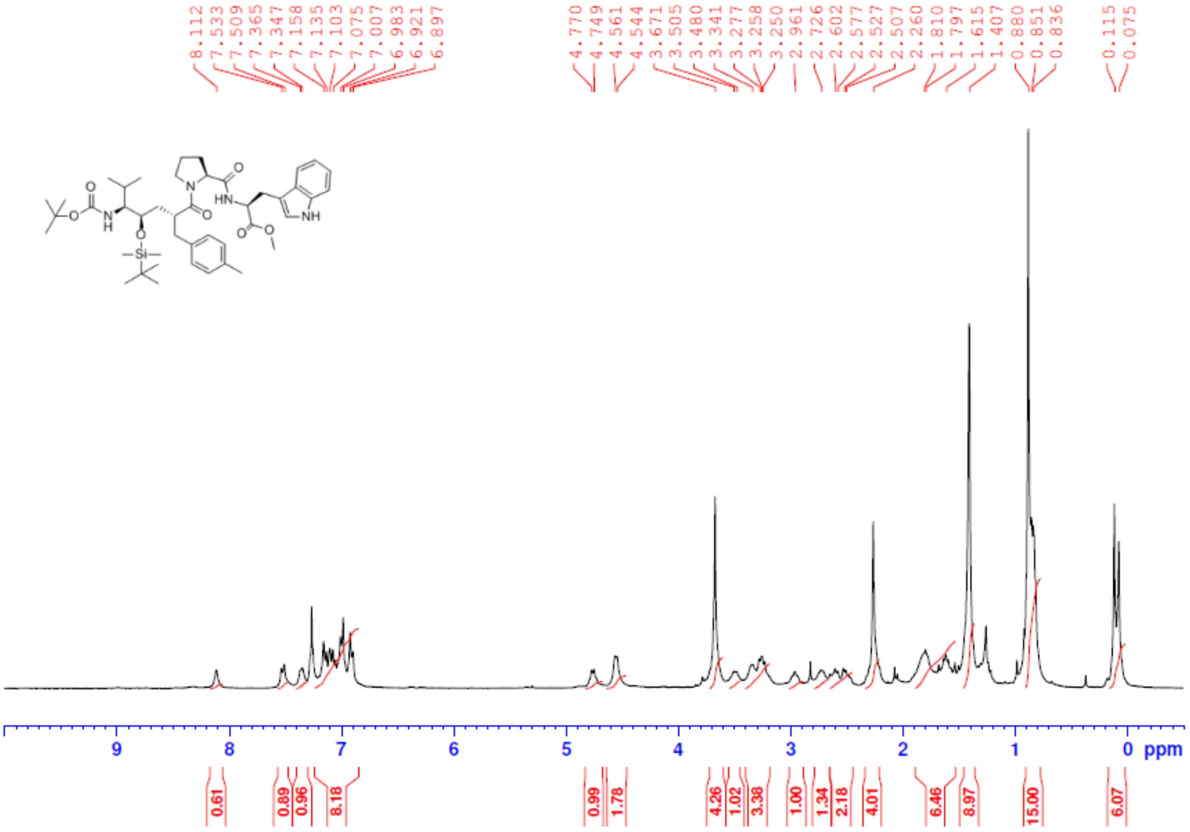

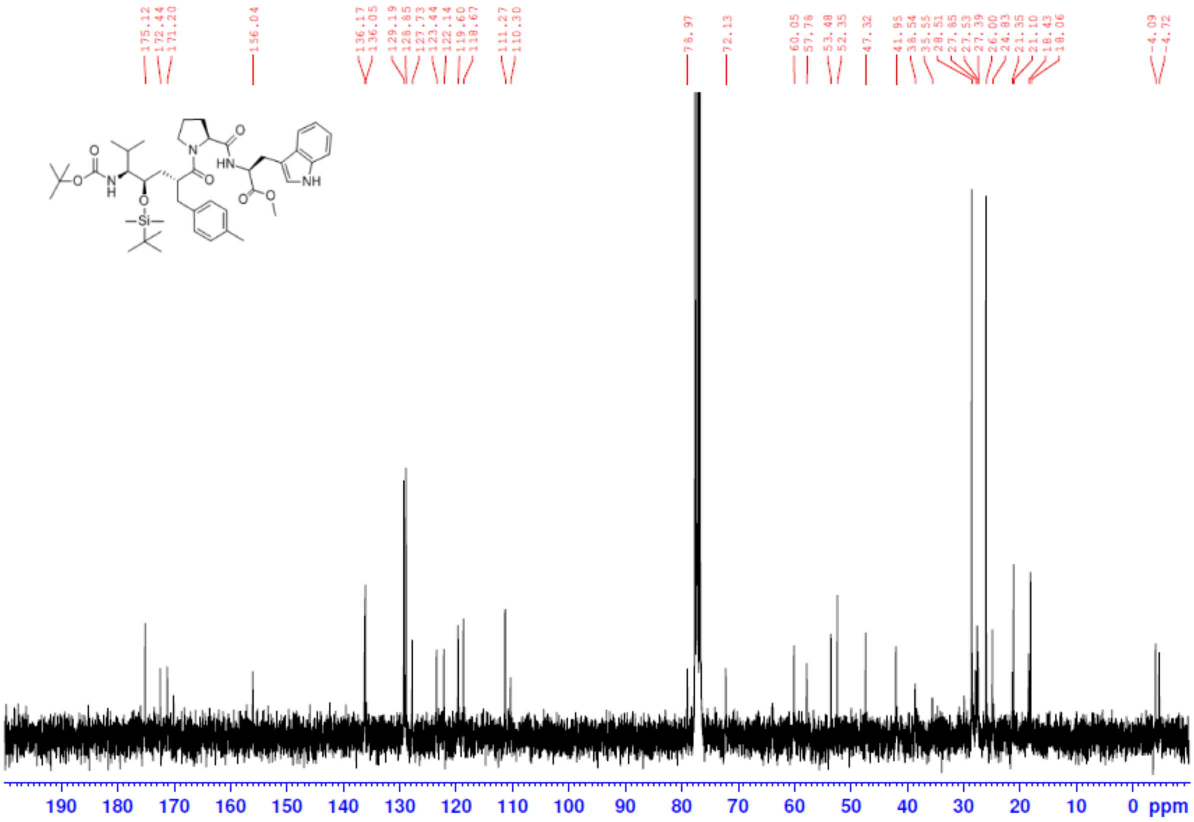

^{1}H and ^{13}C spectra of methyl ((2*R*,4*R*,5*S*)-5-((*S*)-2-((*tert*-butoxycarbonyl)amino)-3-methylbutanamido)-4-hydroxy-6-methyl-2-(4-methylbenzyl)heptanoyl)-L-prolyl-L-tryptophanate (30b)

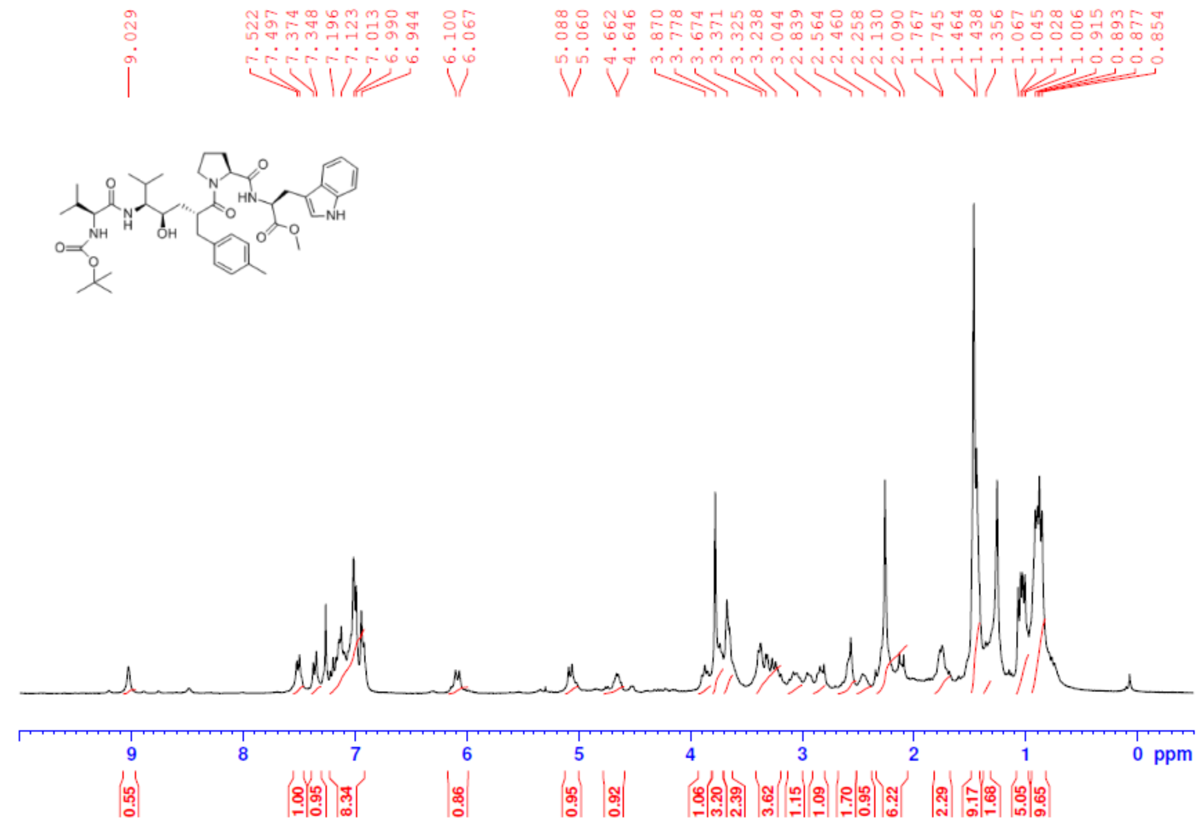

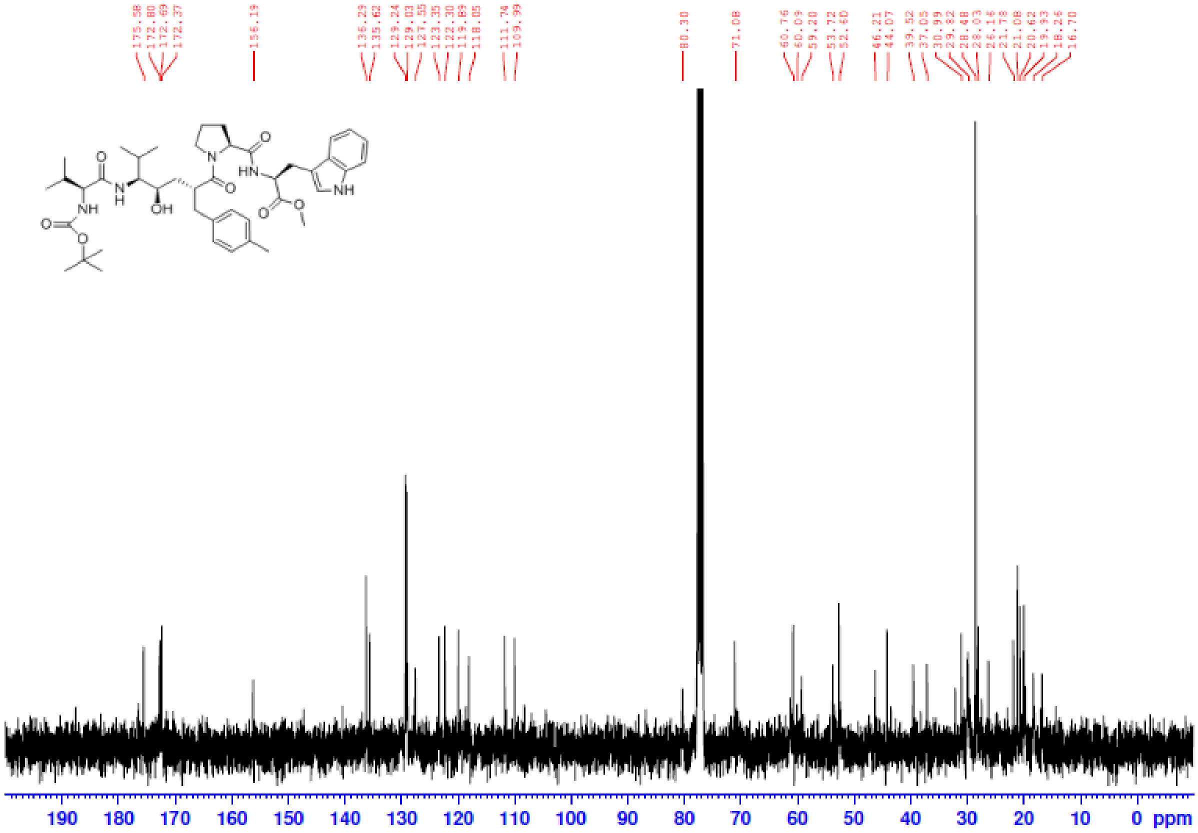

^{1}H and ^{13}C spectra of ((2*R*,4*R*,5*S*)-5-((*S*)-2-ammonio-3-methylbutanamido)-4-hydroxy-6-methyl-2-(4-methylbenzyl)heptanoyl)-L-prolyl-L-tryptophanate (31b)

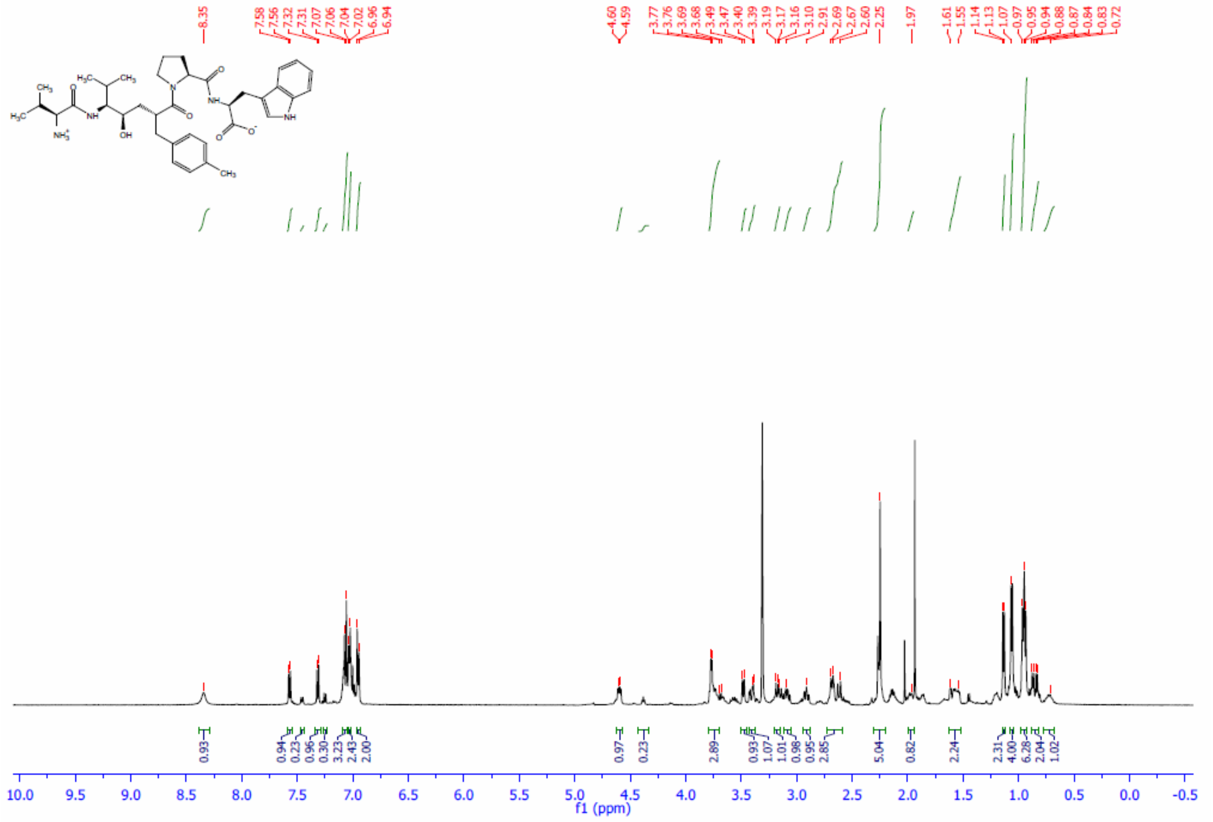

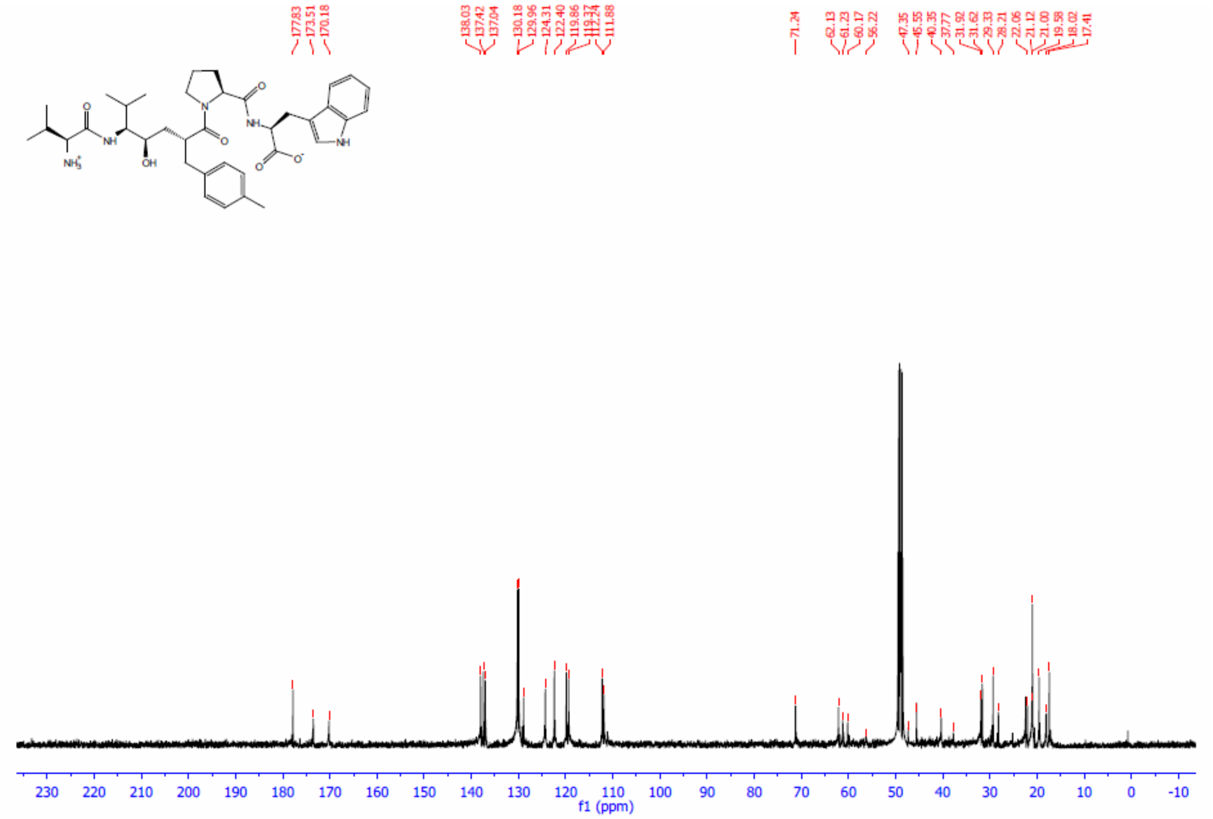

^{1}H and ^{13}C spectra of *tert*-butyl ((1*S*)-1-((2*R*)-4-((4-((*tert*-butyldimethylsilyl)oxy)phenyl)(hydroxy)methyl)-5-oxotetrahydrofuran-2-yl)-2-methylpropyl)carbamate (24c)

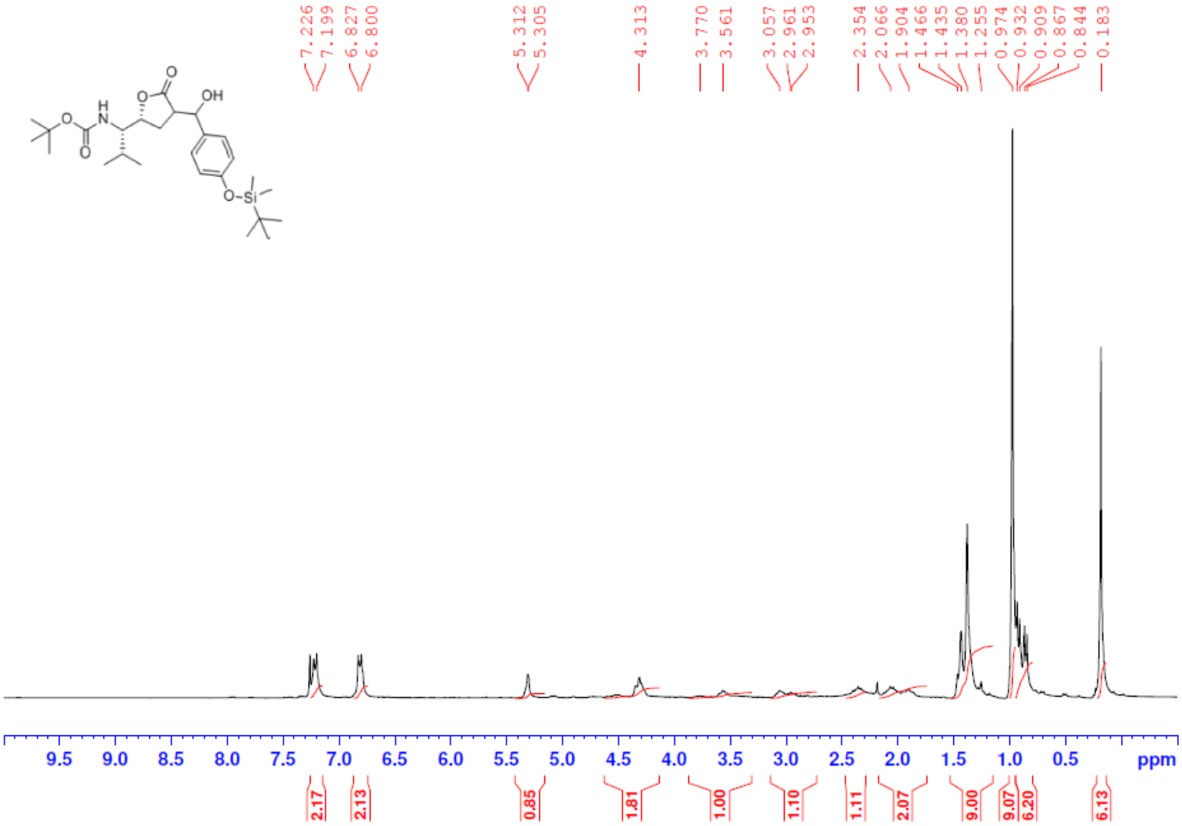

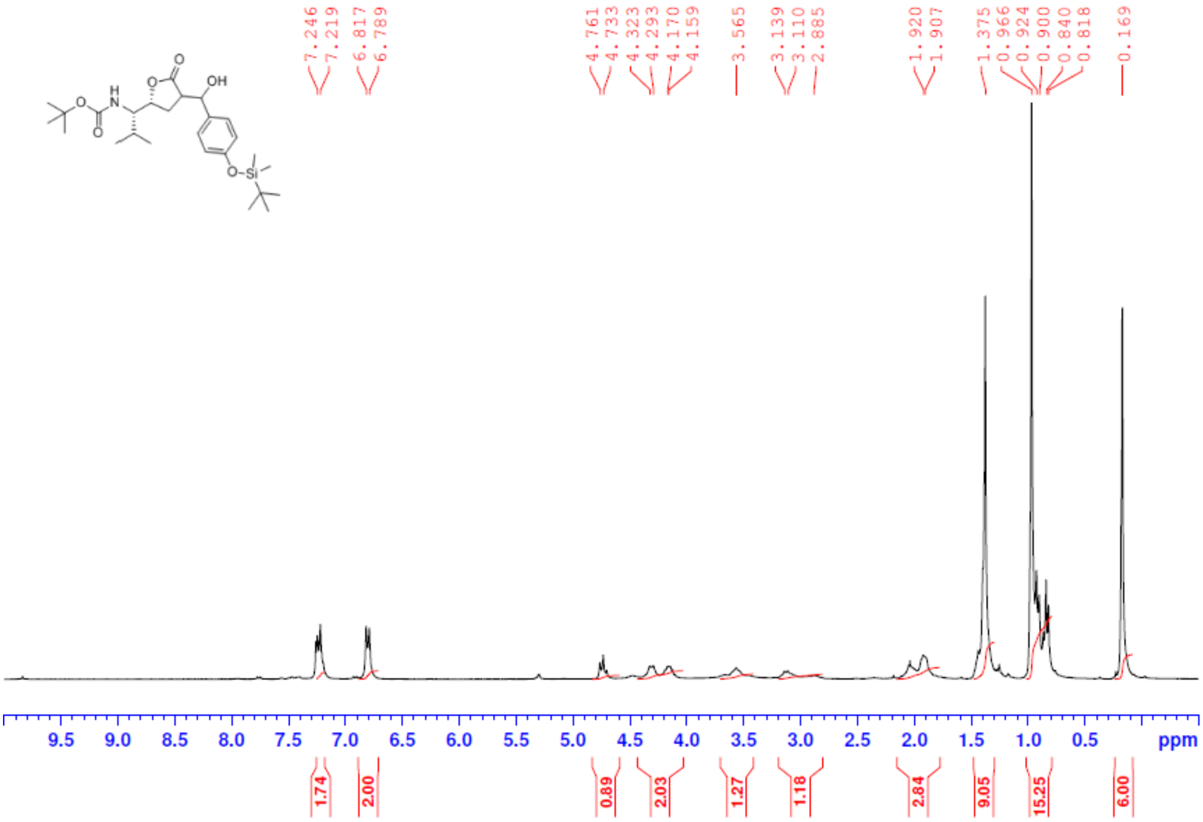

^{1}H and ^{13}C spectra of *tert*-butyl ((*S*)-1-((2*R*,4*R*)-4-(4-((*tert*-butyldimethylsilyl)oxy)benzyl)-5-oxotetrahydrofuran-2-yl)-2-methylpropyl)carbamate (25c)

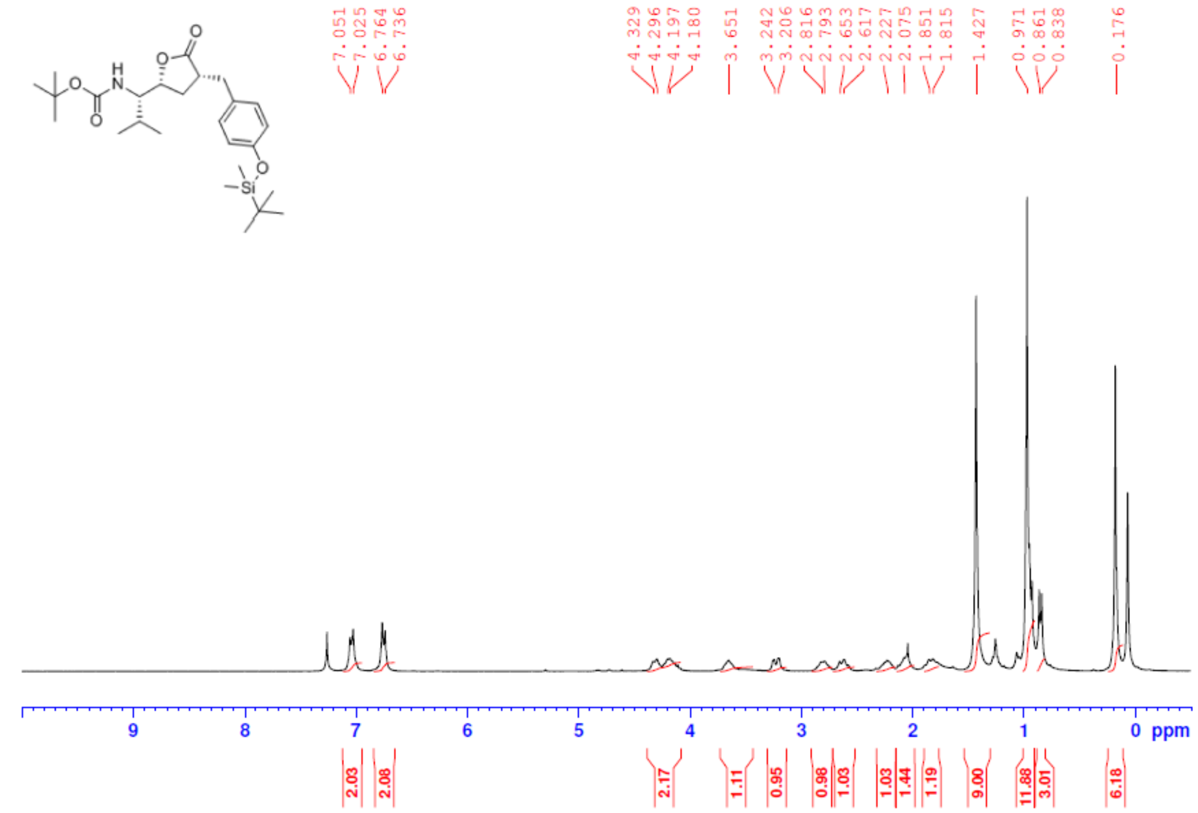
7.051
7.025
6.764
6.736
4.329
4.296
4.197
4.180
3.651
3.242
3.206
2.816
2.793
2.653
2.617
2.227
2.075
1.851
1.815
1.427
0.971
0.861
0.838
0.176
9
8
7
6
5
4
3
2
1
0 ppm
2.03
2.08
2.17
1.11
0.95
0.98
1.03
1.03
1.44
1.19
9.00
11.88
3.01
6.18

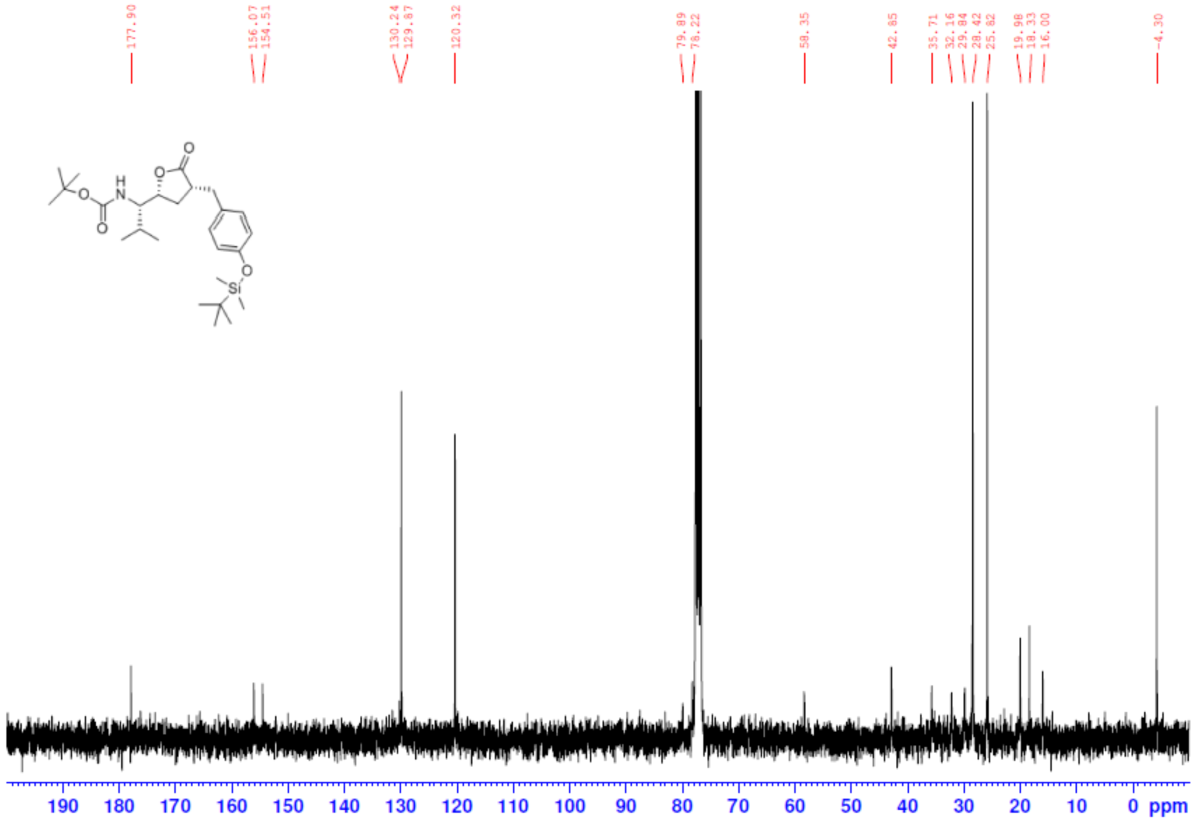
177.90
156.07
154.51
130.24
129.87
120.32
79.89
78.22
58.35
42.85
35.71
32.16
29.94
28.42
25.82
19.98
18.33
16.00
-4.30
190
180
170
160
150
140
130
120
110
100
90
80
70
60
50
40
30
20
10
0 ppm

^{1}H and ^{13}C spectra of (2*R*,4*R*,5*S*)-5-((*tert*-butoxycarbonyl)amino)-4-((*tert*-butyldimethylsilyl)oxy)-2-(4-((*tert*-butyldimethylsilyl)oxy)benzyl)-6-methylheptanoic acid (27c)

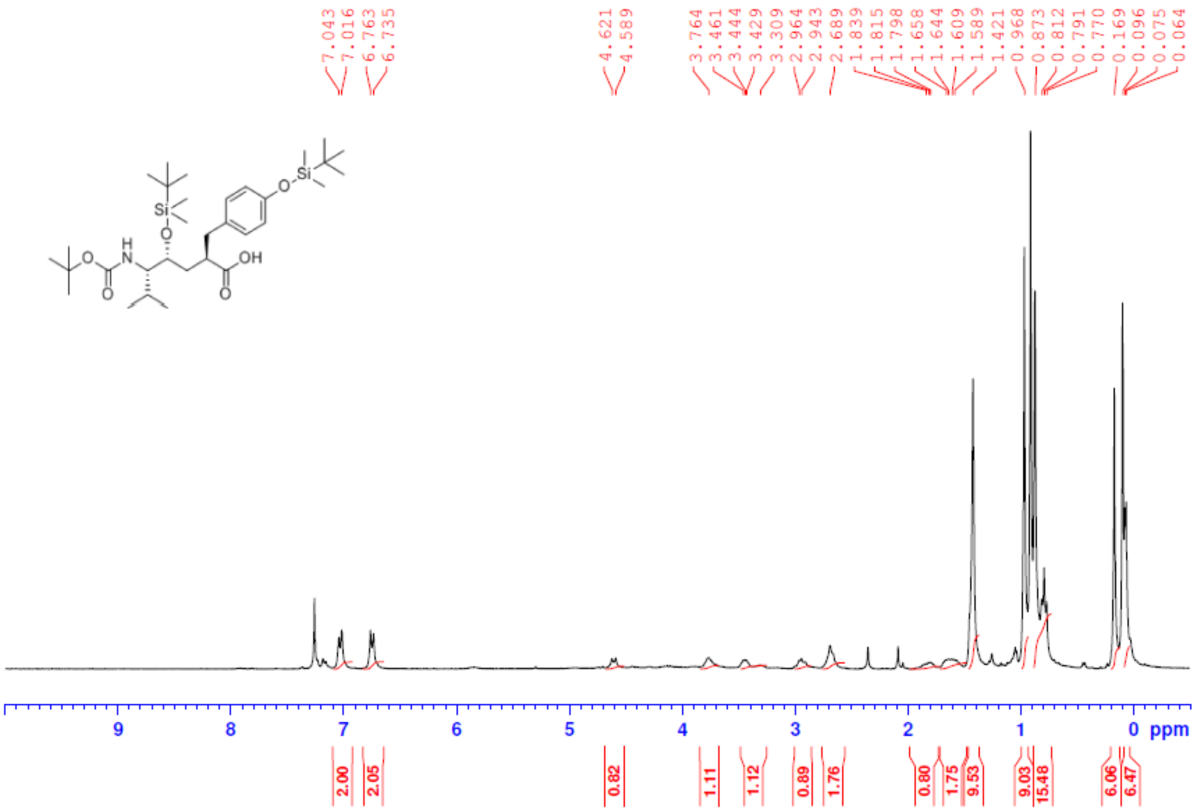

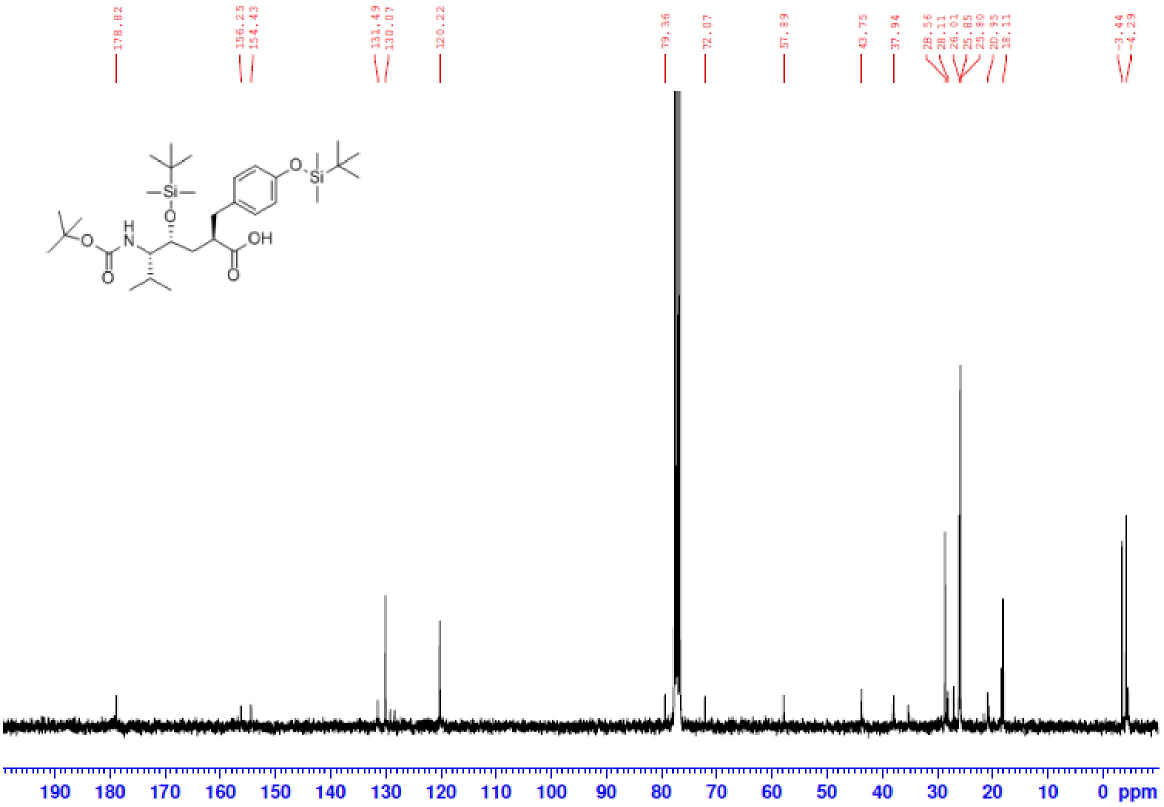

^{1}H and ^{13}C spectra of methyl ((2*R*,4*R*,5*S*)-5-((*tert*-butoxycarbonyl)amino)-4-((*tert*-butyldimethylsilyl)oxy)-2-(4-((*tert*-butyldimethylsilyl)oxy)benzyl)-6-methylheptanoyl)-L-prolyl-L-tryptophanate (28c)

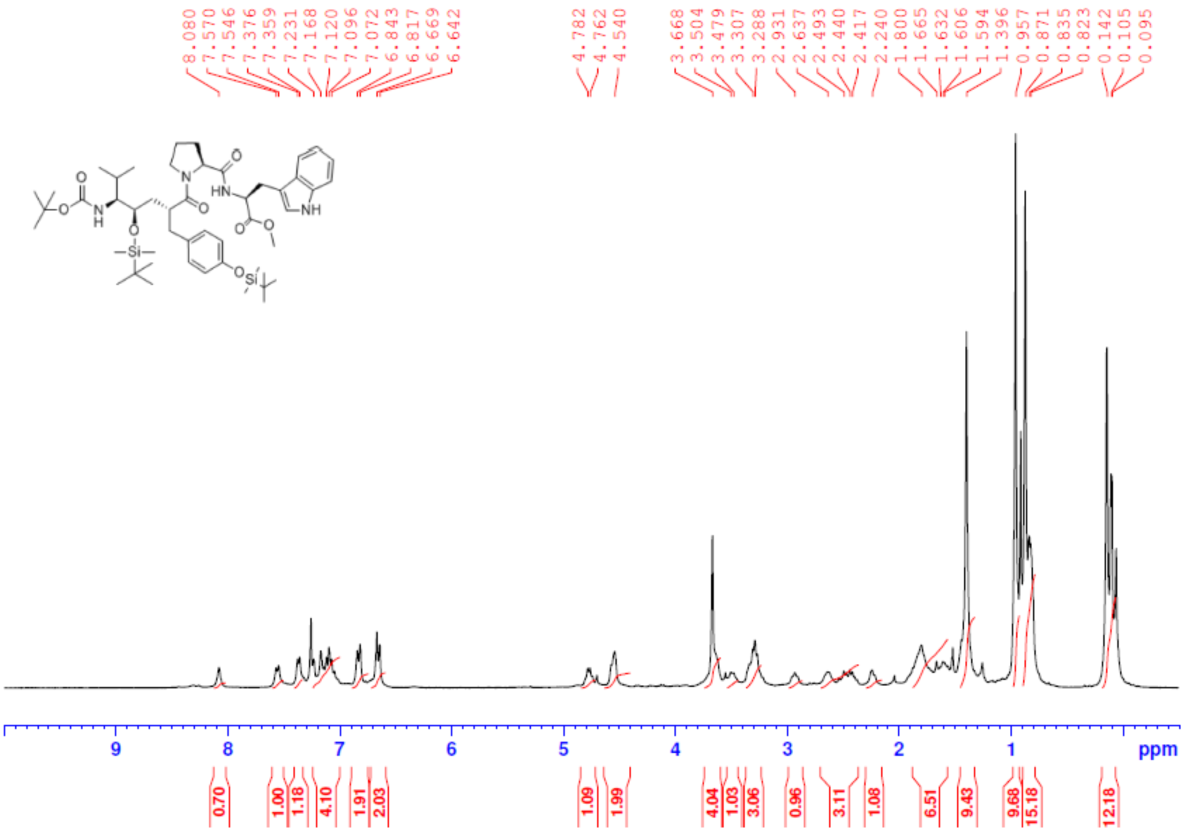

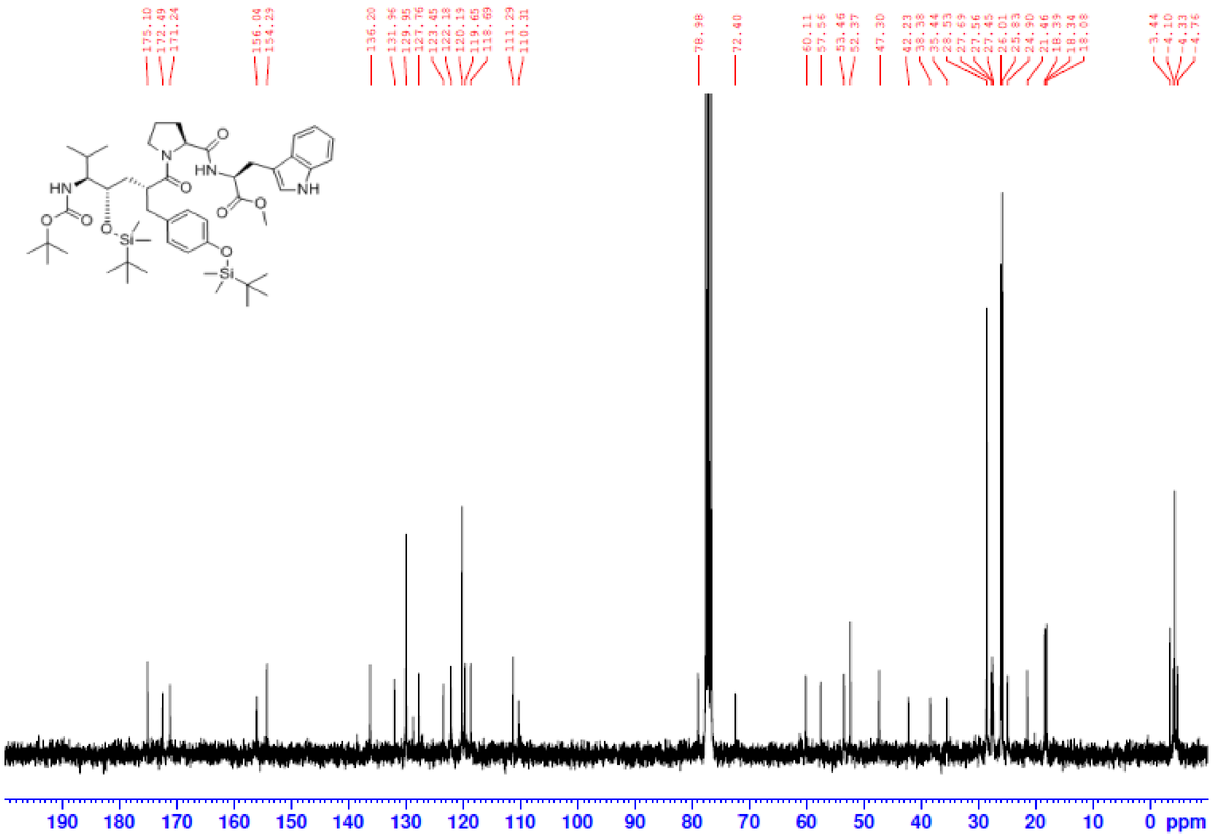

^{1}H and ^{13}C spectra of methyl ((2*R*,4*R*,5*S*)-5-((*S*)-2-((tert-butoxycarbonyl)amino)-3-methylbutanamido)-4-hydroxy-2-(4-hydroxybenzyl)-6-methylheptanoyl)-L-prolyl-L-tryptophanate (28c*)

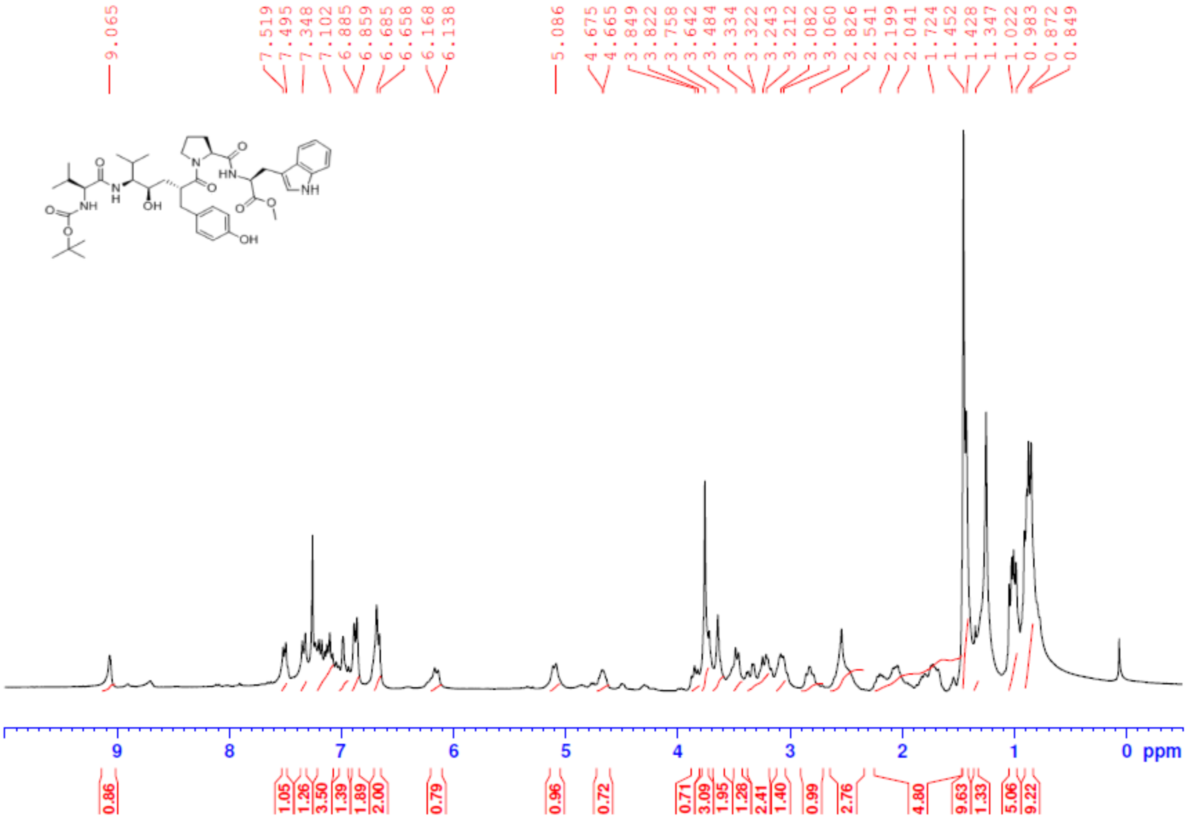
9.065
7.519
7.495
7.348
7.102
6.885
6.859
6.685
6.658
6.168
6.138
5.086
4.675
4.665
3.849
3.822
3.758
3.642
3.484
3.334
3.322
3.243
3.212
3.082
3.060
2.826
2.541
2.199
2.041
1.724
1.452
1.428
1.347
1.022
0.983
0.872
0.849
NH
OH
HN
NH
OH
9 8 7 6 5 4 3 2 1 0 ppm
0.86
1.05
1.26
3.50
1.39
1.89
2.00
0.79
0.96
0.72
0.71
3.09
1.95
1.28
2.41
1.40
0.99
2.76
4.80
9.63
1.33
5.06
9.22

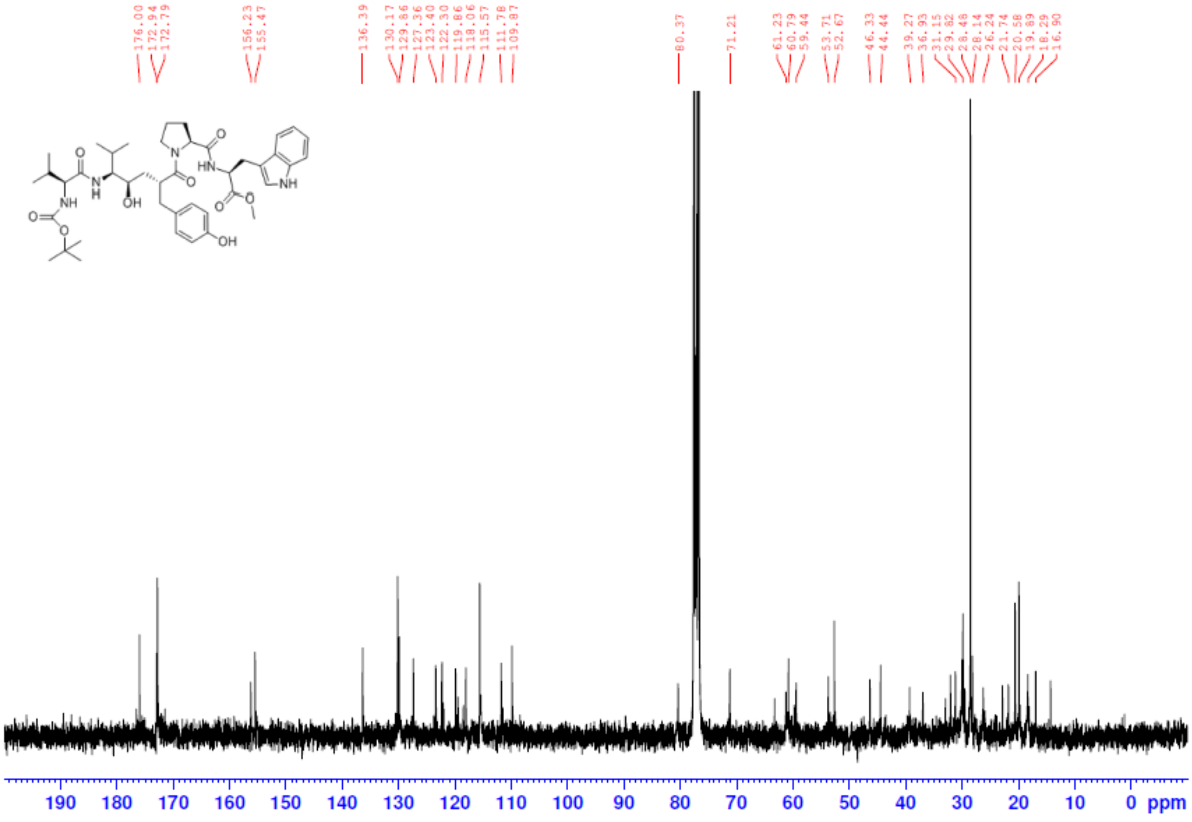
176.00
172.94
172.79
156.23
155.47
136.39
130.17
129.86
127.36
123.40
122.30
119.86
118.06
115.57
111.78
109.87
80.37
71.21
61.23
60.79
59.44
53.71
52.67
46.33
44.44
39.27
36.93
31.15
29.82
28.48
28.14
26.24
21.74
20.58
19.89
18.29
16.90
NH
OH
HN
NH
OH
190 180 170 160 150 140 130 120 110 100 90 80 70 60 50 40 30 20 10 0 ppm

^{1}H and ^{13}C spectra of ((2*R*,4*R*,5*S*)-5-((*S*)-2-ammonio-3-methylbutanamido)-4-hydroxy-2-(4-hydroxybenzyl)-6-methylheptanoyl)-L-prolyl-L-tryptophanate (31c)

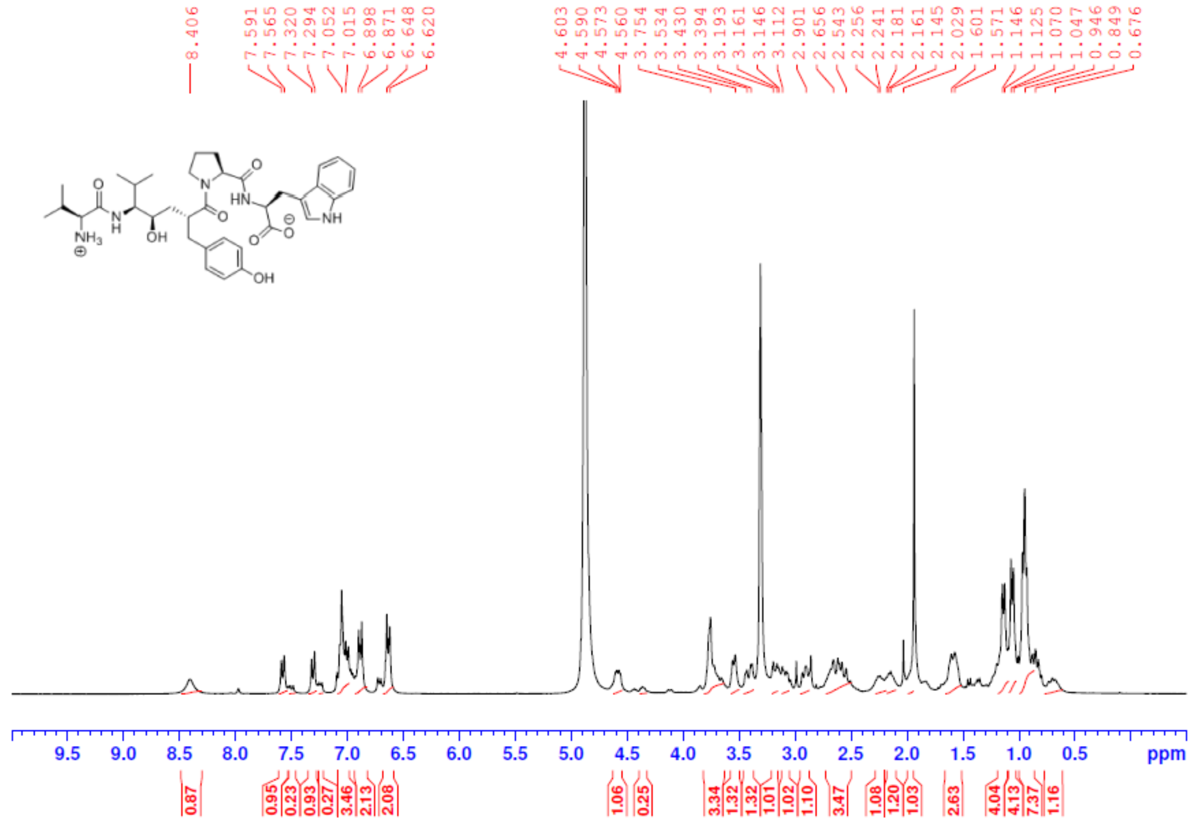

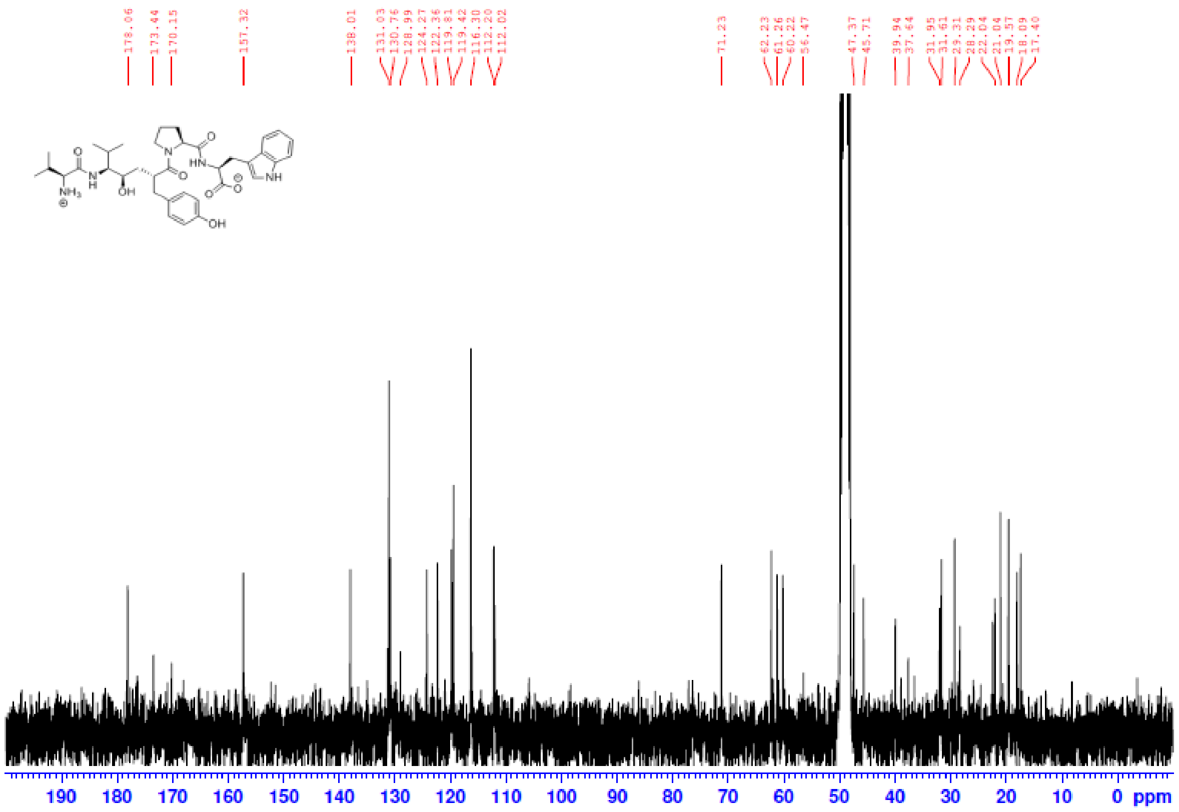

^{1}H and ^{13}C spectra of methyl ((2*R*,4*R*,5*S*)-5-((*S*)-2-amino-3-methylbutanamido)-4-hydroxy-2-(4-hydroxybenzyl)-6-methylheptanoyl)-L-prolyl-L-tryptophanate (32)

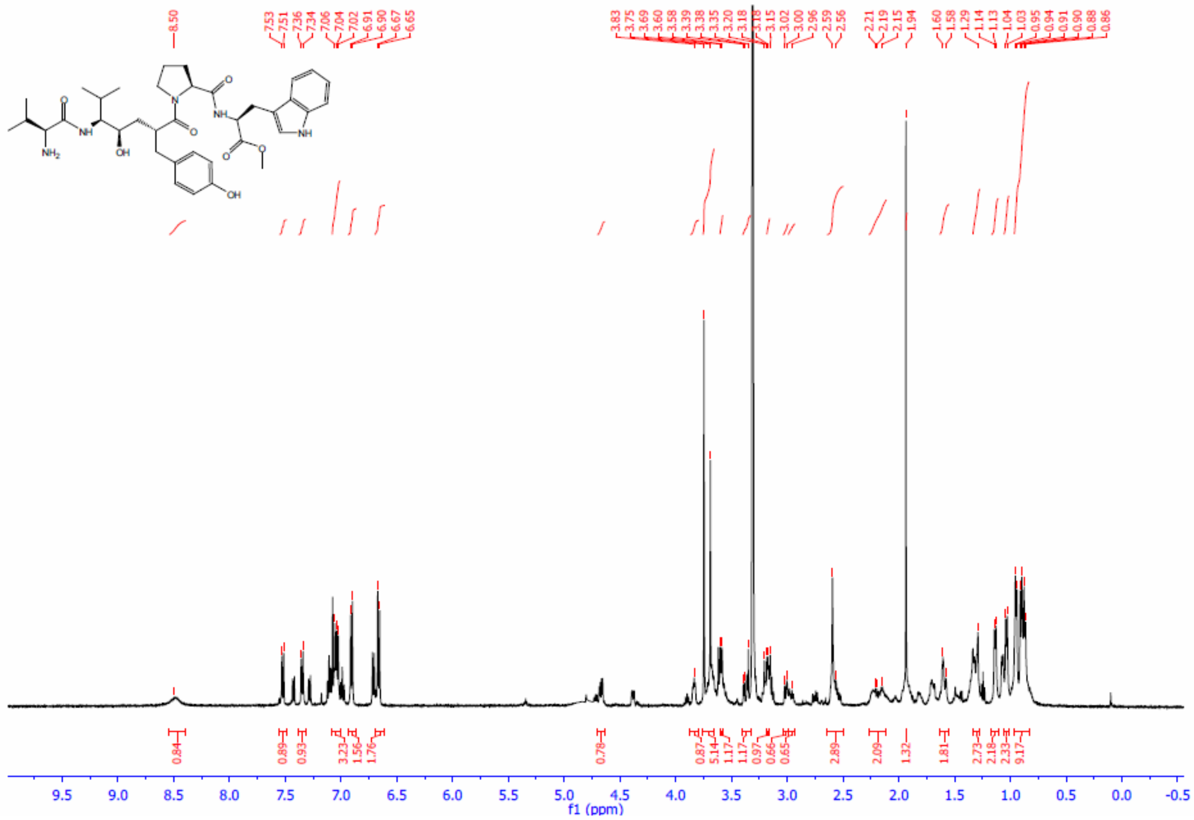

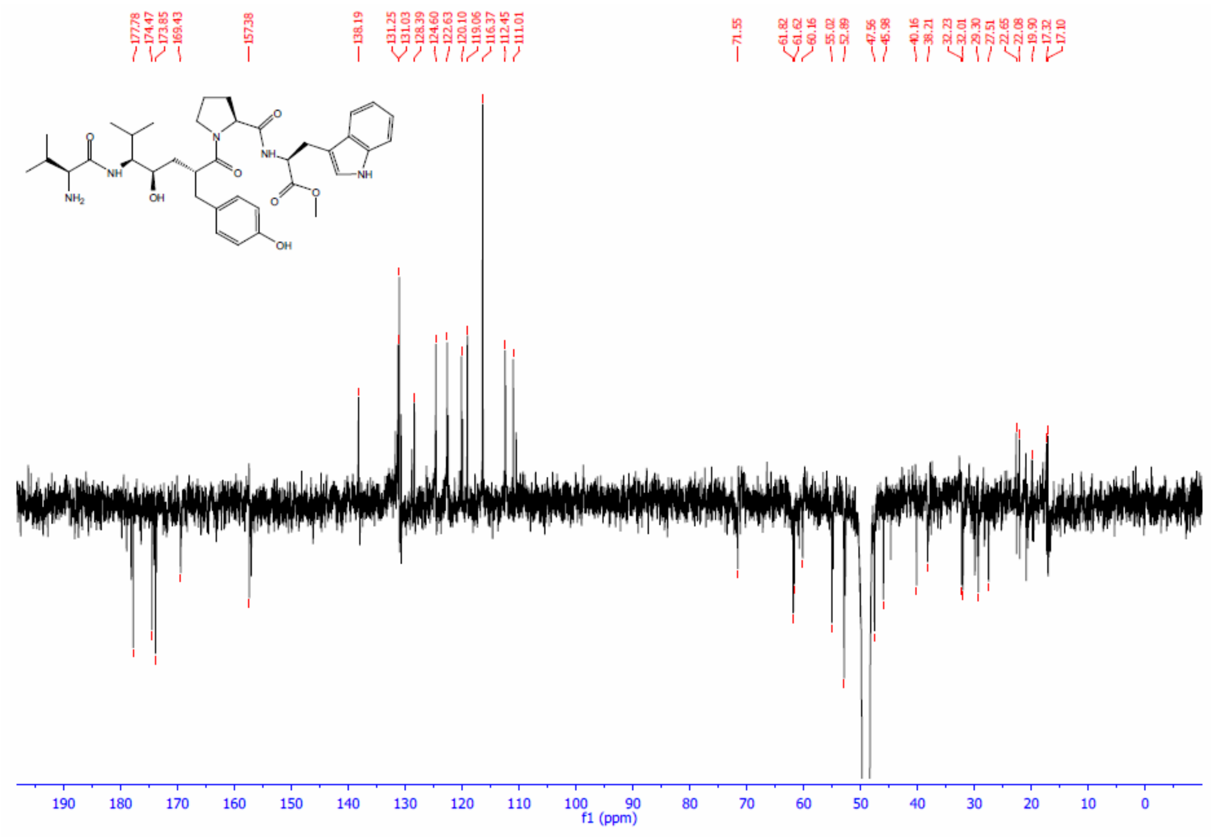

^{1}H and ^{13}C spectra of *tert*-butyl (*S*)-2-(((*S*)-3-(1*H*-indol-3-yl)-1-methoxy-1-oxopropan-2-yl)carbamoyl)piperidine-1-carboxylate

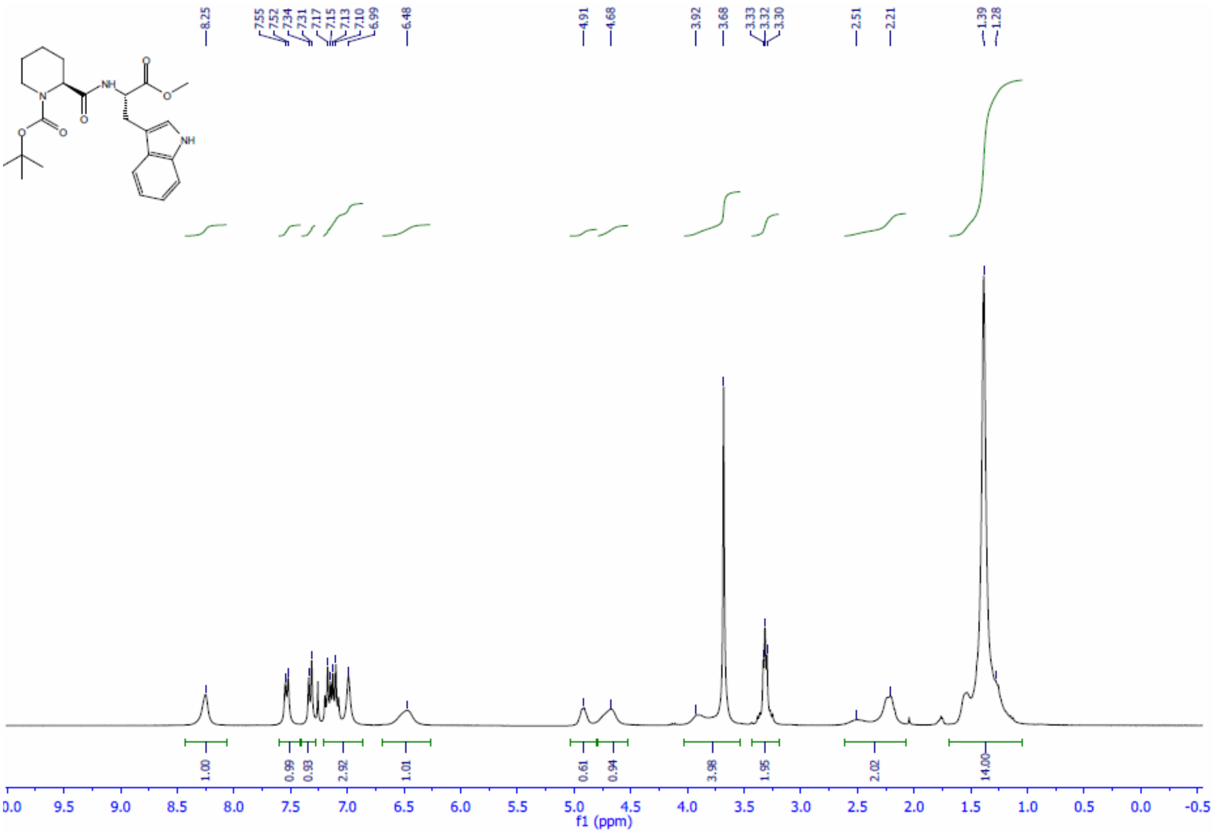
8.25
7.55
7.52
7.34
7.31
7.17
7.15
7.13
7.10
6.99
6.48
4.91
4.68
3.92
3.68
3.33
3.32
3.30
2.51
2.21
1.39
1.28
NH
O
N
1.00
0.99
0.93
2.92
1.01
0.61
0.94
3.98
1.95
2.02
14.00
f1 (ppm)

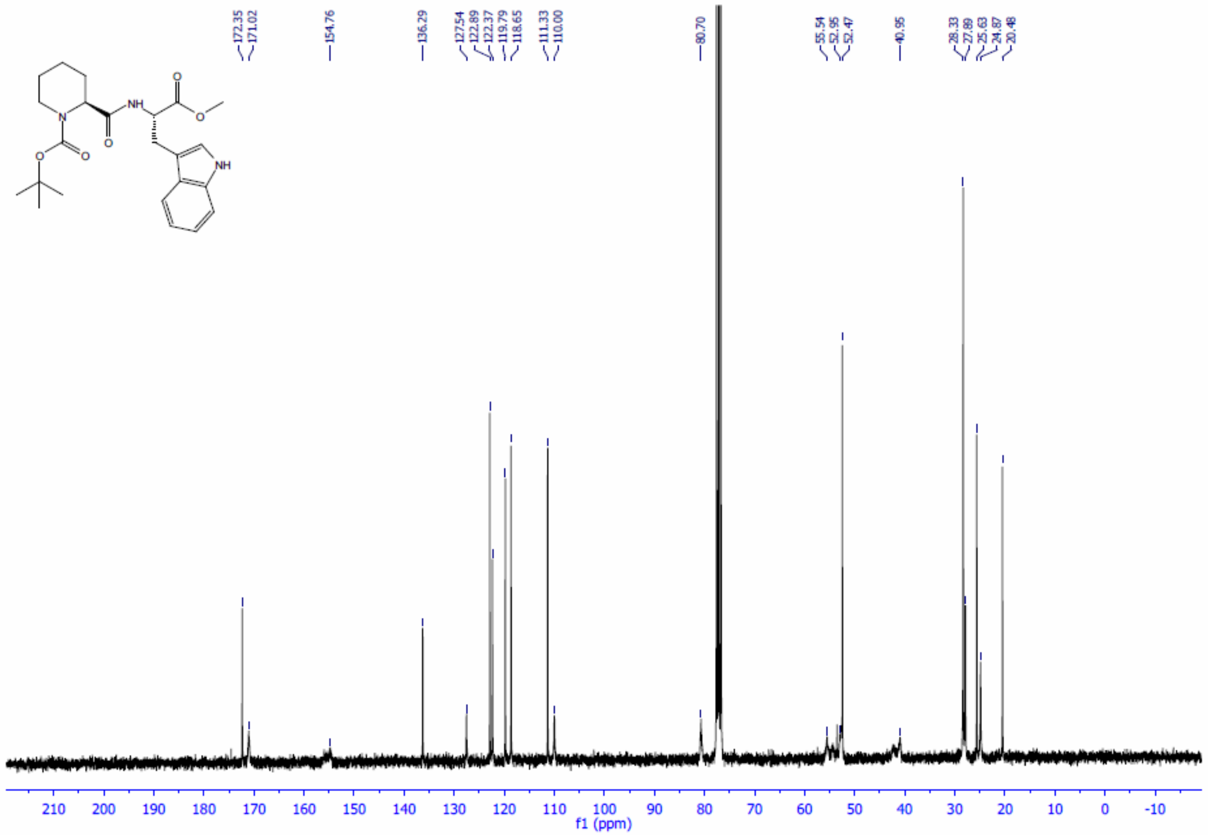
172.35
171.02
154.76
136.29
127.54
122.89
122.37
119.79
118.65
111.33
110.00
80.70
55.54
52.95
52.47
40.95
28.33
27.89
25.63
24.87
20.48
NH
O
N
f1 (ppm)

^{1}H and ^{13}C spectra of methyl ((*S*)-1-((2*R*,4*R*,5*S*)-2-benzyl-5-((*tert*-butoxycarbonyl)amino)-4-((*tert*-butyldimethylsilyl)oxy)-6-methylheptanoyl)piperidine-2-carbonyl)-L-tryptophanate (33)

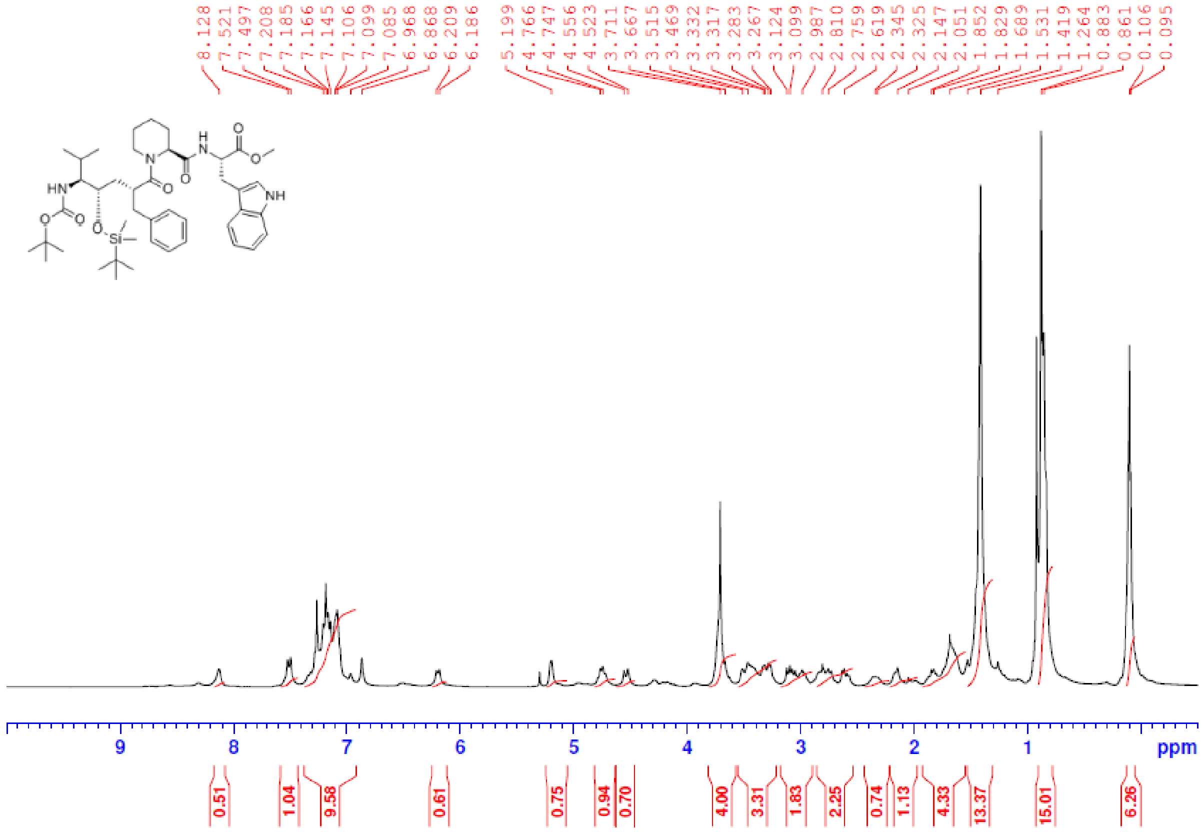

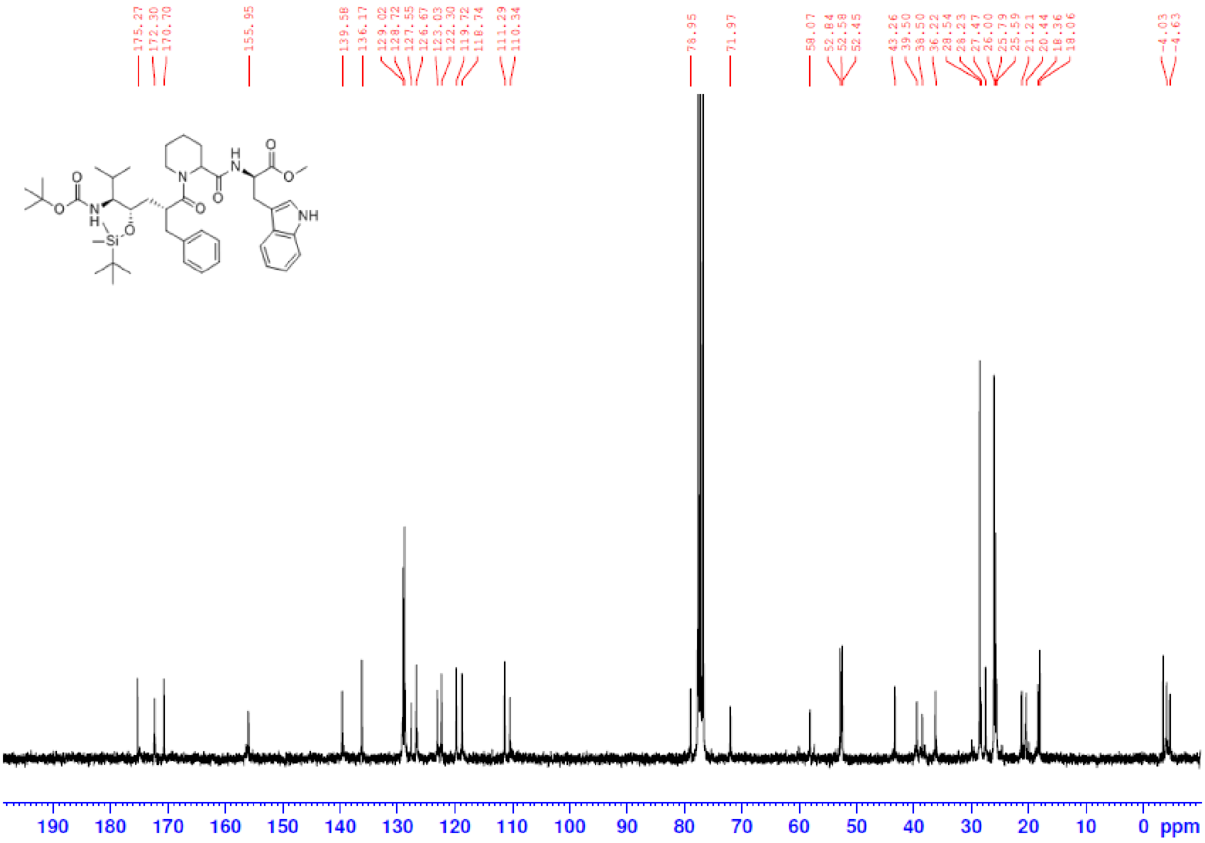

^{1}H and ^{13}C spectra of methyl ((*S*)-1-((2*R*,4*R*,5*S*)-2-benzyl-5-((*S*)-2-((*tert*-butoxycarbonyl)amino)-3-methylbutanamido)-4-hydroxy-6-methylheptanoyl)piperidine-2-carbonyl)-L-tryptophanate (36a)

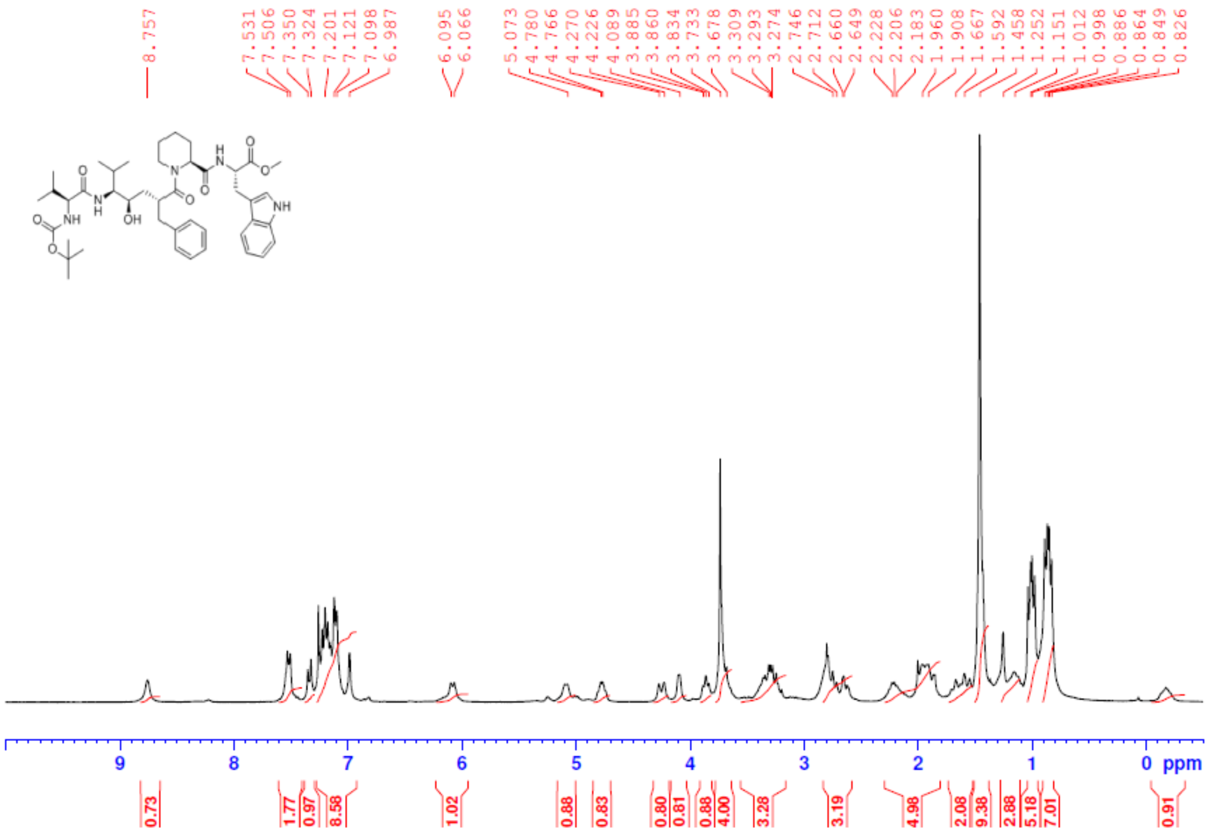

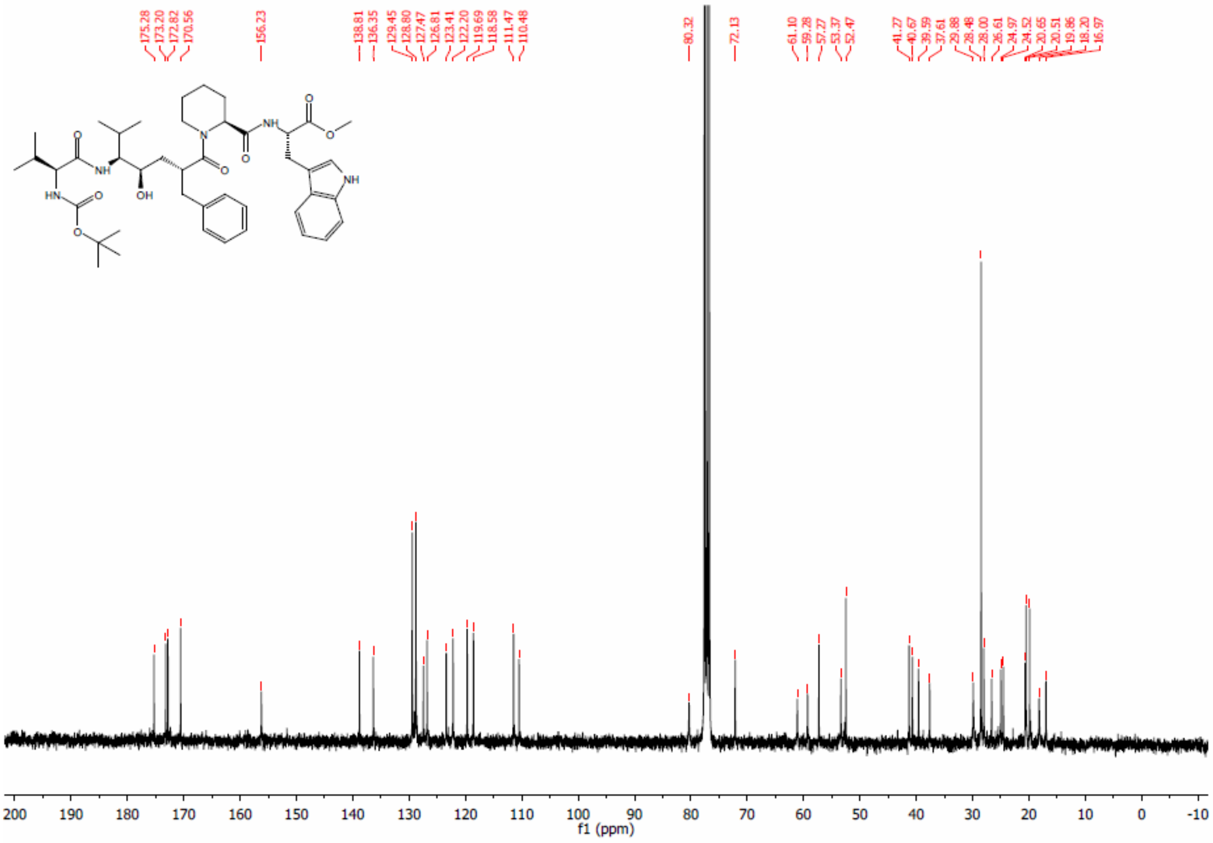

^{1}H and ^{13}C spectra of ((*S*)-1-((2*R*,4*R*,5*S*)-5-((*S*)-2-ammonio-3-methylbutanamido)-2-benzyl-4-hydroxy-6-methylheptanoyl)piperidine-2-carbonyl)-L-tryptophanate (37a)

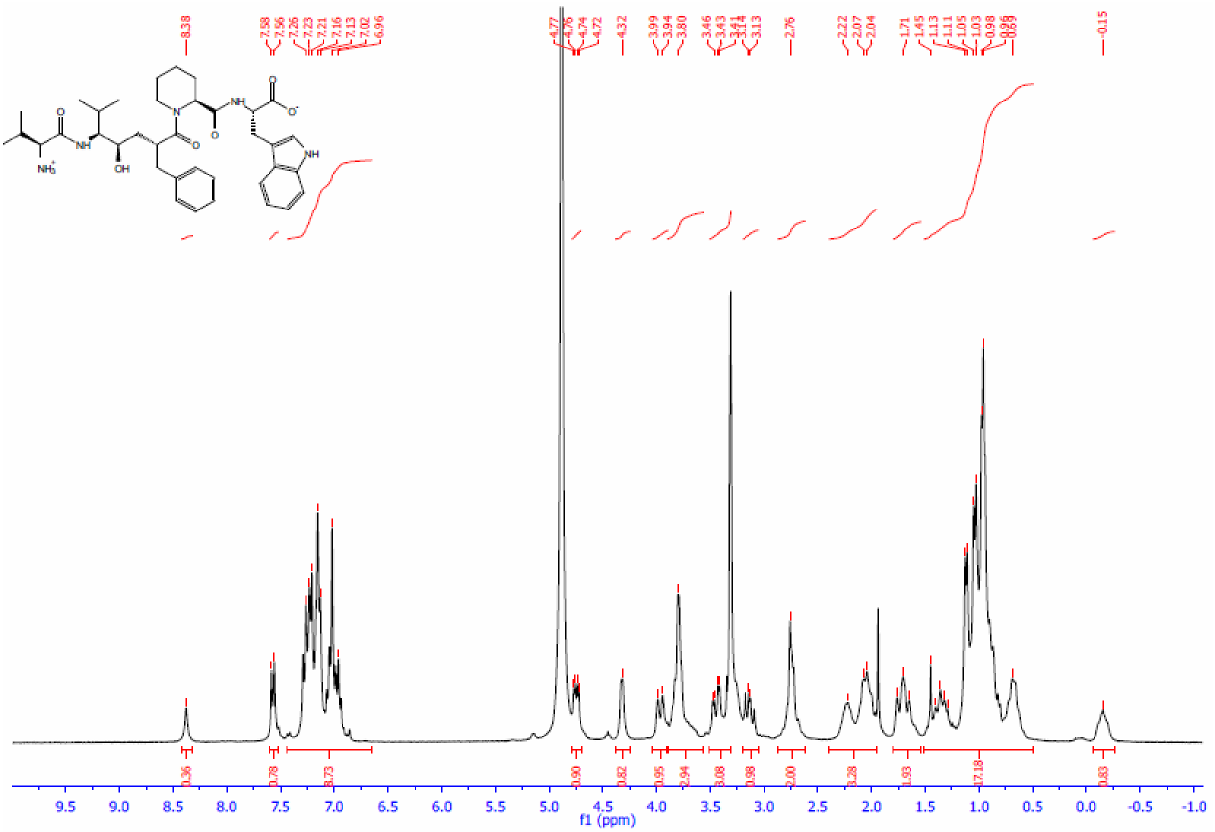

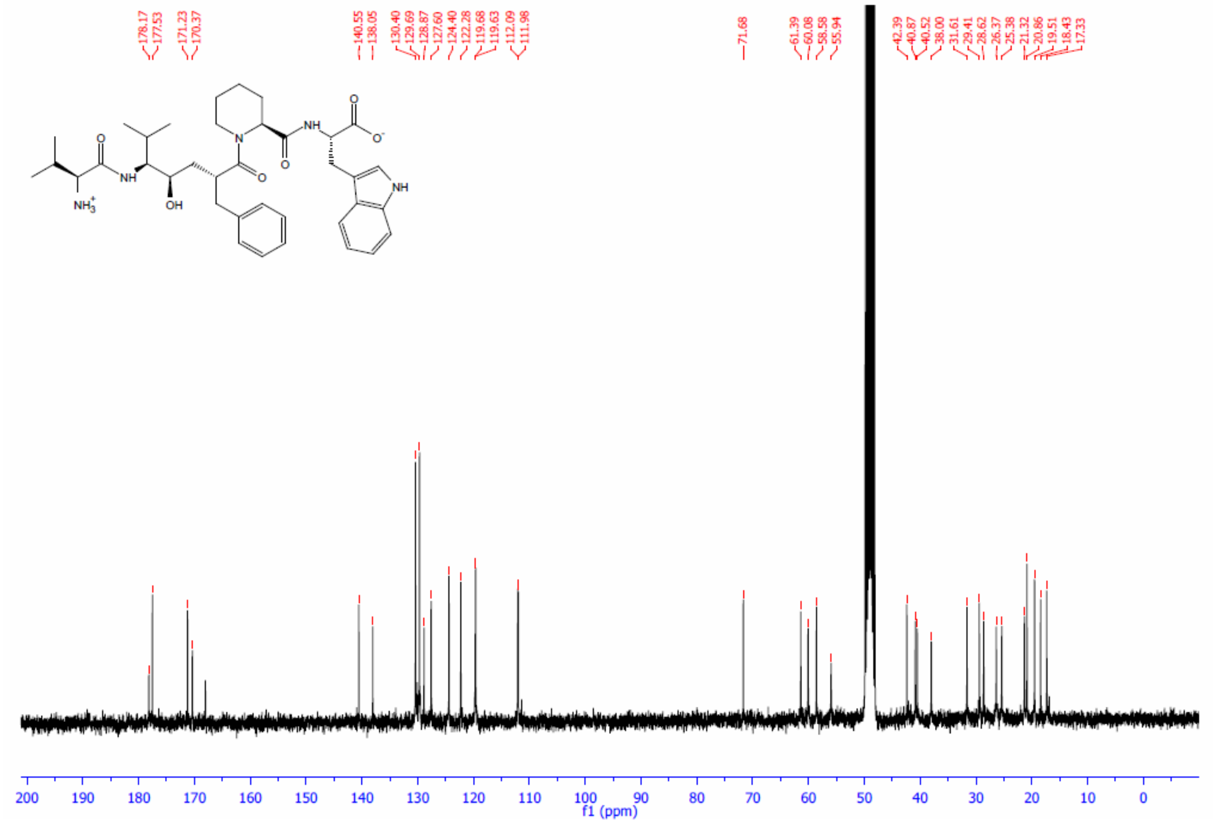

^{1}H and ^{13}C spectra of methyl ((*S*)-1-((2*R*,4*R*,5*S*)-2-benzyl-5-((*S*)-2-((*tert*-butoxycarbonyl)amino)-3,3-dimethylbutanamido)-4-hydroxy-6-methylheptanoyl)piperidine-2-carbonyl)-L-tryptophanate (36b)

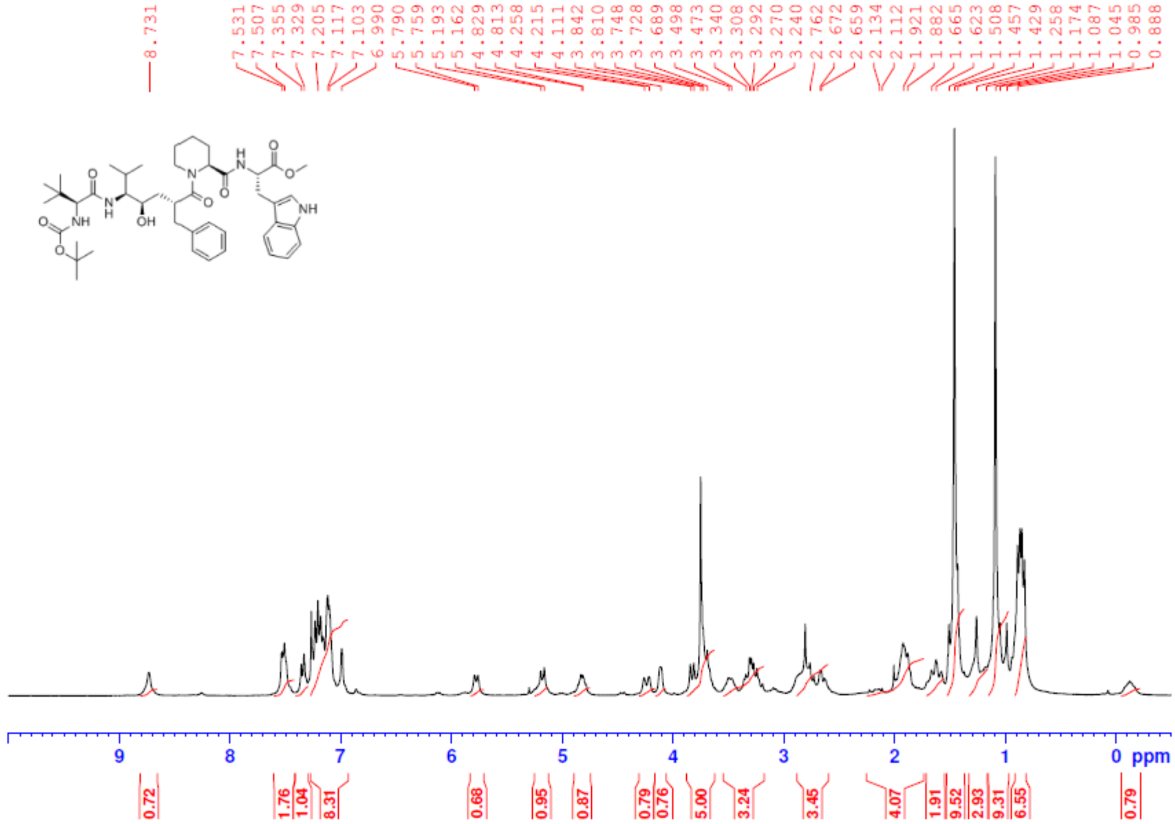
8.731
7.531 7.507 7.355 7.329 7.205 7.117 7.103 6.990 5.790 5.759 5.193 5.162 4.829 4.813 4.258 4.215 4.111 3.842 3.810 3.748 3.728 3.689 3.498 3.473 3.340 3.308 3.292 3.270 3.240 2.762 2.672 2.659 2.134 2.112 1.921 1.882 1.665 1.623 1.508 1.457 1.429 1.258 1.174 1.087 1.045 0.985 0.888
NH
OH
N
O
9 8 7 6 5 4 3 2 1 0 ppm
0.72 1.76 1.04 8.31 0.68 0.95 0.87 0.79 0.76 5.00 3.24 3.45 4.07 1.91 9.52 2.93 9.31 6.55 0.79

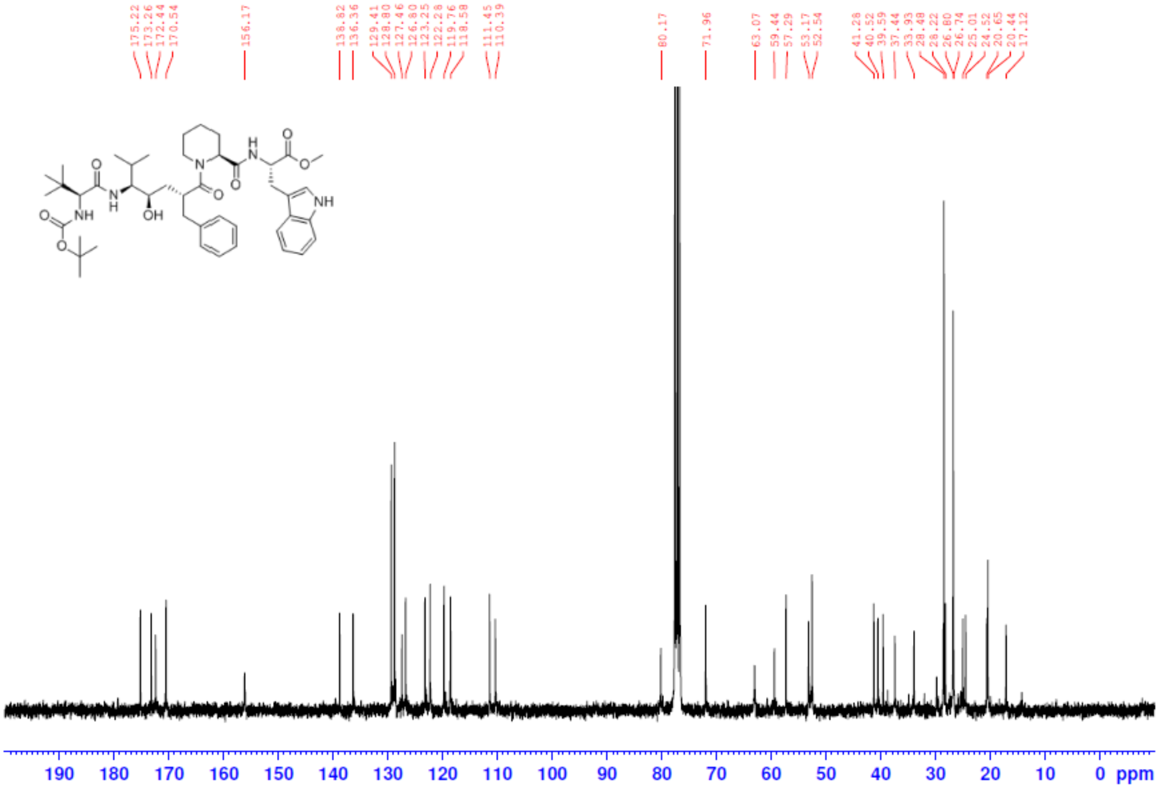
175.22 173.26 172.44 170.54 156.17 138.82 136.36 129.41 128.80 127.46 126.80 123.25 122.28 119.76 118.58 111.45 110.39 80.17 71.96 63.07 59.44 57.29 53.17 52.54 41.28 40.52 39.59 37.44 33.93 28.48 28.22 26.80 26.74 25.01 24.52 20.65 20.44 17.12
NH
OH
N
O
190 180 170 160 150 140 130 120 110 100 90 80 70 60 50 40 30 20 10 0 ppm

^{1}H and ^{13}C spectra of ((*S*)-1-((2*R*,4*R*,5*S*)-5-((*S*)-2-ammonio-3,3-dimethylbutanamido)-2-benzyl-4-hydroxy-6-methylheptanoyl)piperidine-2-carbonyl)-L-tryptophanate (37b)

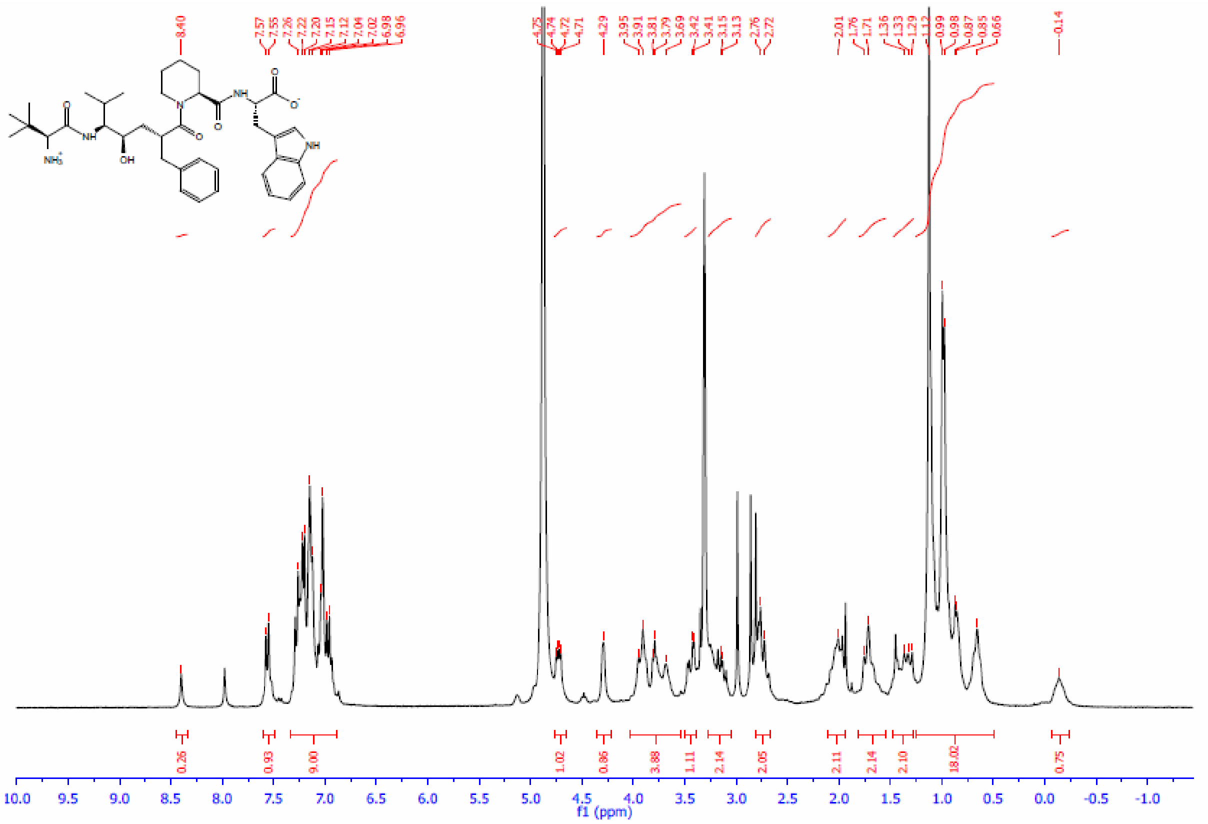

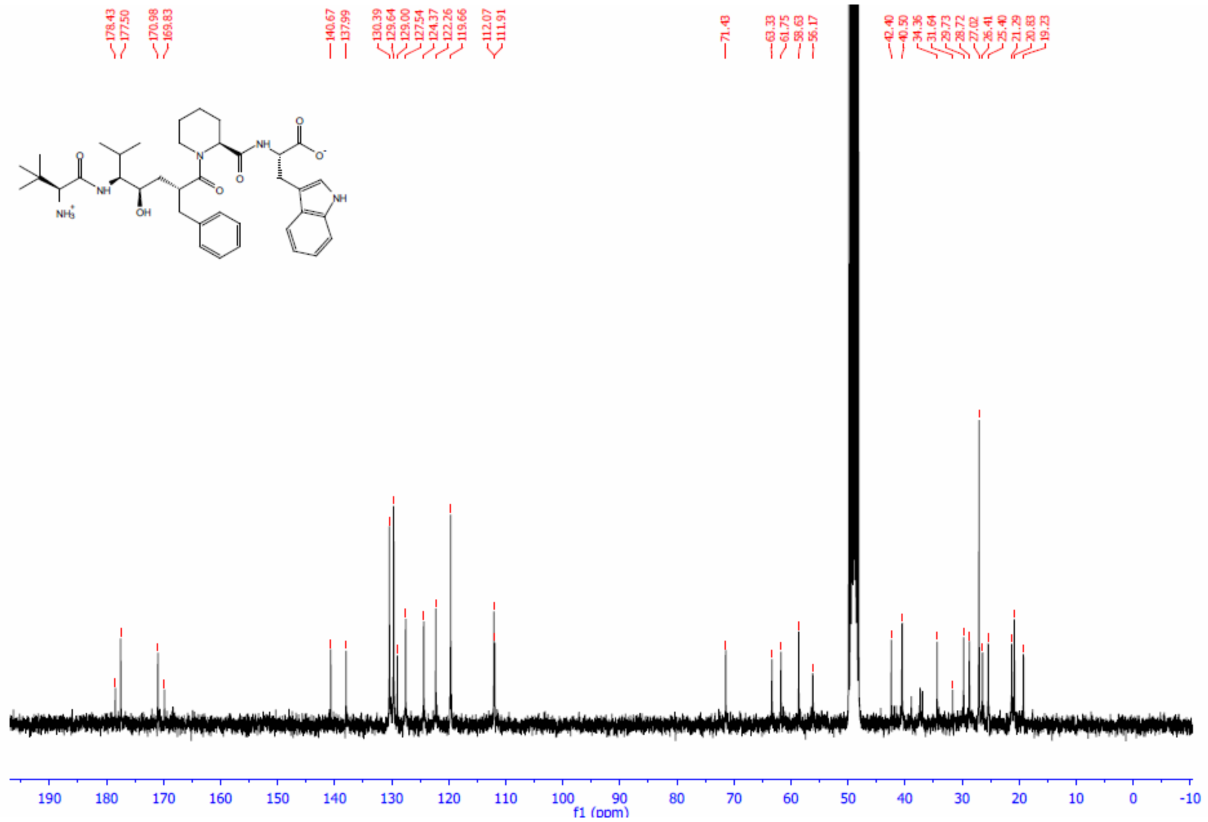

Supplement: Supplementary file 1 — Supporting Information [file CHEM-27-14108-s001.pdf]
